# Metal-Free Aziridination of Unactivated Olefins via Transient *N*-Pyridinium Iminoiodinanes

**DOI:** 10.1021/jacsau.4c00556

**Published:** 2024-10-10

**Authors:** Hao Tan, Phong Thai, Uddalak Sengupta, Isaac R. Deavenport, Cali M. Kucifer, David C. Powers

**Affiliations:** Department of Chemistry, Texas A&M University, College Station, Texas 77843, United States

**Keywords:** olefin aziridination, iminoiodinane, nitrogen-group transfer, C−N coupling, N-aminopyridinium

## Abstract

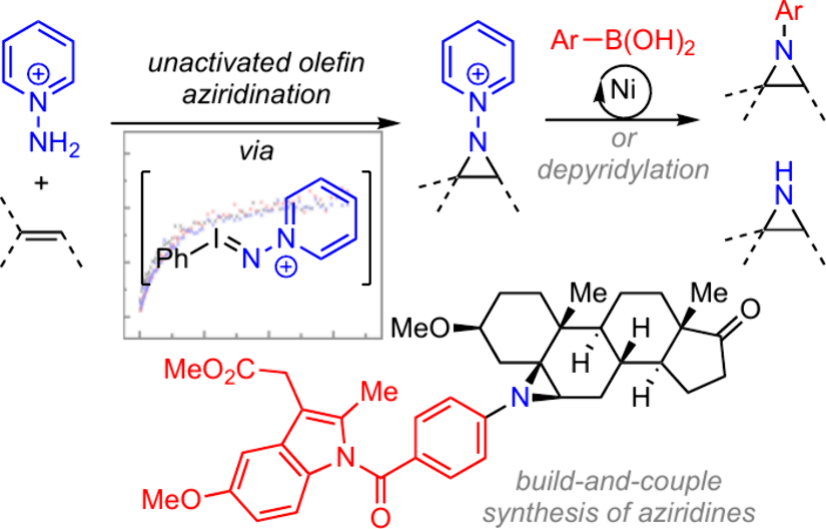

We describe a metal-free aziridination of unactivated olefins to generate *N-*pyridinium aziridines. Subsequent cross-coupling affords *N*-aryl aziridines, and reductive depyridylation affords N–H aziridines. Kinetics experiments, based on a variable time normalization analysis (VTNA), indicate that aziridination proceeds via a highly electrophilic *N*-pyridinium iminoiodinane intermediate. These studies expand *build-and-couple* aziridine synthesis to unactivated olefins and introduce charge-enhanced electrophilicity into the chemistry of iminoiodinanes.

Strained three-membered heterocycles, such as epoxides and aziridines, are frequently encountered in synthetic intermediates and bioactive small molecules due to the combination of predictable reactivity, widespread availability, and benchtop stability.^1^ Epoxides feature prominently in bioactive small molecules due to the facility of epoxide-opening reactions with biological nucleophiles.^2^ Aziridines also display strain-enhanced electrophilicity, however, are much less often encountered in natural products.^2,3^ The dearth of naturally occurring aziridines has evolutionary origins: While robust enzymatic machinery is available for oxygen-atom transfer (OAT) to olefins, the corresponding nitrogen-group transfer (NGT) is not characteristic of natural enzymatic catalysts.^4^

In concept, aziridines are attractive synthetic targets because *N*-functionalization systematically tunes the electrophilicity: In epoxides, divalent oxygen engages in two endocyclic C–O bonds; in aziridines, trivalent nitrogen engages in two endocyclic C–N bonds as well as an exocyclic N–R bond that directly impacts the electrophilicity of the resulting aziridine. In practice, new synthetic methods are needed to realize a general platform for olefin aziridination with respect to olefin scope and *N*-functionalization.^5^

For activated olefins (i.e., styrenes and enol ether derivatives), both metal-catalyzed^3b,6^ and metal-free^7^ aziridination reactions have been developed. Electron withdrawing N-substituents are often required on the nitrene equivalent (i.e., iminoiodinanes, azides, or hydroxylamine derivatives), and thus these methods typically afford *N*-sulfonyl or *N*-acyl aziridines.^8^ Recent progress has begun to broaden the availability of *N*-functionalized^9^ and N–H aziridines.^10^ Despite the synthetic focus on activated olefins, most naturally occurring aziridines are derived from aliphatic olefins (Figure 1a). Classical strategies for unactivated olefin aziridination involve functional group manipulation from the corresponding epoxides.^11^ More recently, metal-catalyzed aziridination reactions of unactivated olefins have been developed, but in general, aliphatic olefins are less reactive than styrenyl olefins.^12^ Of particular note are emerging methods to access N–H aziridines,^10b,13^ as well as photo-^14^ and electrochemical reactions^15^ that can be applied to unactivated olefins.

**Figure 1:**
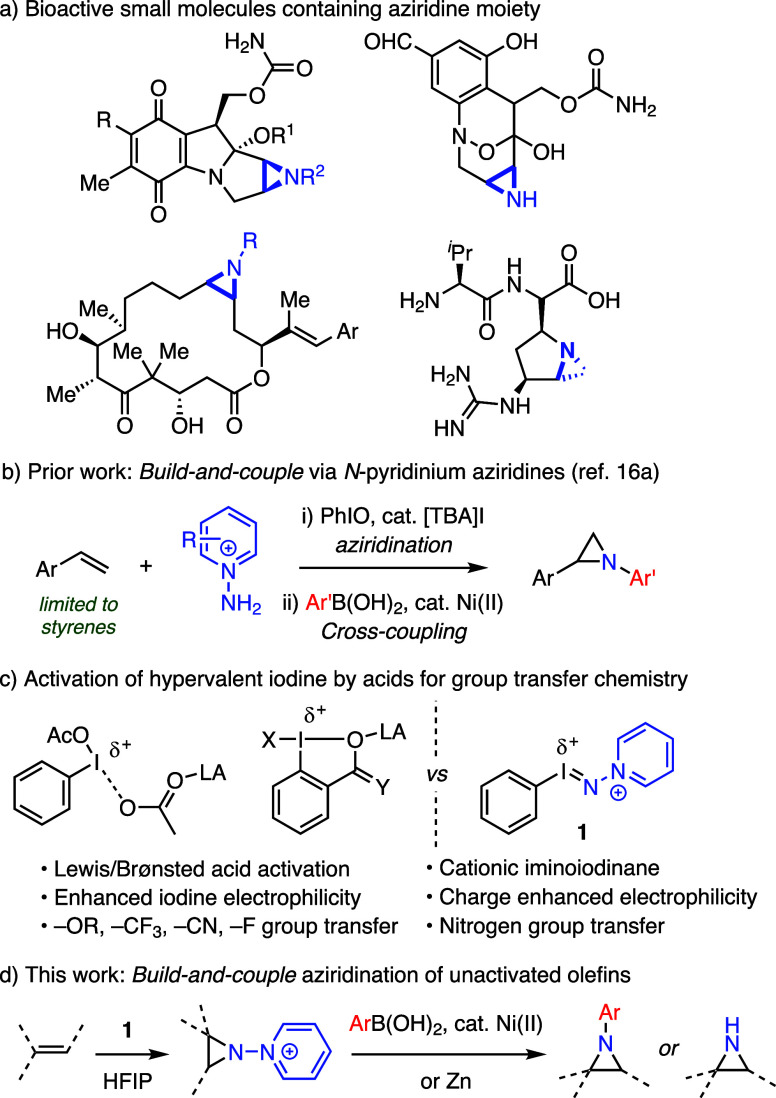
(a) Bioactive aziridines derived from unactivated olefins. (b) Previously, build-and-couple aziridination has been limited to styrenyl olefins. (c) Acid activation can transiently enhance hypervalent iodine electrophilicity. Here charge-enhanced electrophilicity enables nitrene-transfer catalysis to unactivated olefins. (d) Charge-enhanced hypervalent iodine activity underpins build-and-couple aziridination of unactivated olefins.

Previously, we developed a *build-and-couple* synthesis of *N*-arylaziridines via sequential olefin *N*-aminopyridylation followed by Ni-catalyzed C–N coupling with aryl boronic acids (Figure 1b).^16^ While this approach enabled efficient diversification of the exocyclic nitrogen valence of aziridines, the original iodide-catalyzed reaction failed to effect the aziridination of unactivated olefins in synthetically useful yields. We envisioned *build-and-couple* aziridination of unactivated olefins could be achieved if a highly electrophilic source of *N*-pyridinium nitrene equivalents were available. To this end, we were motivated by reports of acid-activated hypervalent iodine reagents (Figure 1c).^17^ In this paradigm, reversible acid activation enhances the electrophilicity of hypervalent iodine reagents. We reasoned that persistently activated iminoiodinanes would be generated if the hypervalent iodine center featured a cationic N*-*substituent.

Here we describe aziridination of unactivated olefins via the intermediacy of highly electrophilic *N*-pyridinium iminoiodinane **1** (Figure 1d), generated by the condensation of an *N*-aminopyridinium salt with iodosylbenzene. By virtue of the proximity of the cationic pyridinium residue to the electrophilic hypervalent iodine center, iminoiodinane **1** is exceptionally electrophilic. This approach provides a platform for olefin *N*-aminopyridylation that avoids the radical intermediates involved in previously described iodide-catalyzed reactions and thus enables aziridination of unactivated olefins including complex molecules. These results expand the synthetic chemistry of *N*-functionalized aziridines to include unactivated olefins and demonstrate charge labeling as a new strategy in hypervalent iodine activation.

Using cyclohexene (**2a**) as an archetypal unactivated olefin, we identified conditions for highly efficient aziridination: Combination of **2a** with *N*-aminopyridinium triflate (**3**), iodosylbenzene (**4**), and 4 Å molecular sieves in HFIP afforded the corresponding *N-*pyridinium aziridine (**5a**) in 99% NMR yield (eq 1).^18^ Excess **4** is crucial to accomplish efficient aziridination using cyclohexene as the limiting reagent and the reaction is highly solvent dependent, with HFIP being optimal (see Supporting Information for optimization details regarding stoichiometry, concentration, and solvents).^19^ Of significance, metal salts that are often utilized in aziridination catalysis, such as AgOTf, Cu(I), Cu(II), Rh_2_(tfacam)_4_, and Mn(TPP)Cl negatively impacted aziridination efficiency.
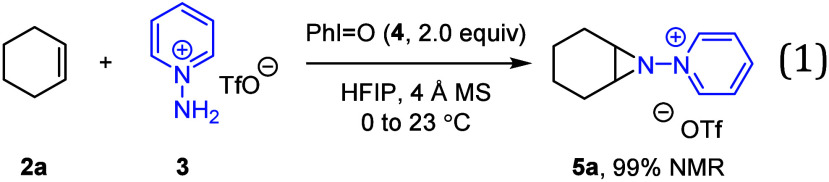
1

Figure 2 depicts aliphatic olefins that participate in efficient aziridination.^20^ Compounds **5a**, **5b**, **5d**, and **5e** are formed with exclusively *cis*-stereochemistry, while **5c** afforded a mixture of diastereomers (73% yield, *c-***5c**: *t*-**5c** = 6.25:1.00 assigned based on ^1^H coupling constants), likely as a result of the larger ring size. Similarly, aziridination of 1,5-cyclooctadiene (**2f**), *cis*-2-hexene (*c*-**2g**), and *t*-**2g** afforded 1.0:1.0 (69% yield, Figure S2), 2.0:1.0 (68% yield), and 5.0:6.0 (77% yield) *cis*-/*trans*-mixtures, respectively. The stereochemistry of *t*-**5f** was confirmed by single-crystal X-ray diffraction (SCXRD). Norbornene undergoes *exo*-aziridination to afford **5e** in 69% yield; the stereochemistry was confirmed by SCXRD following cross coupling (*vide infra*). Terminal alkenes featuring halide, ester, nitrile, and azide substituents underwent efficient aziridination (**5h**–**5q**). Aziridination of 4-vinyl homoallylbenzene **2r** proceeded exclusively at the styrenyl C=C bond to afford **5r** in 65% yield, and *N*-heterocycles were also tolerated (i.e., **2s**).

**Figure 2:**
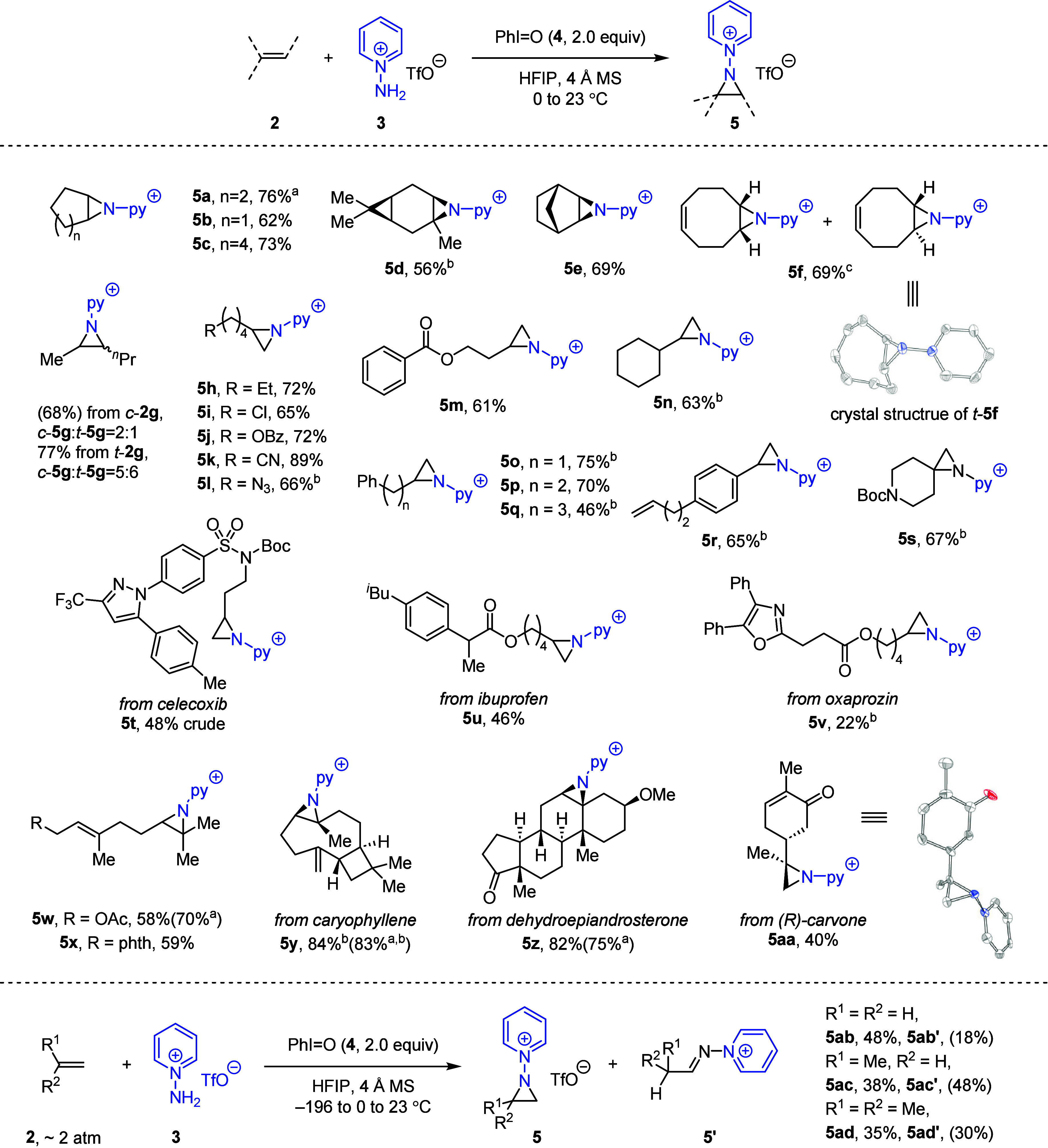
Synthesis of *N*-pyridinium aziridines from unactivated olefins. Conditions: **2** (1.0 equiv, 0.30 mmol), **3** (1.0 equiv, 0.1 M), PhI = O (**4**, 2.0 equiv). Isolated yield (NMR yield). ^a^**2** (1.0 mmol); ^b^CH_3_CN was used as solvent because the substrate was not stable in HFIP; ^c^Isolated as a *cis-*/*trans-*mixture.

Olefin-containing pharmaceutical derivatives **2t** (celecoxib-derived), **2u** (ibuprofen), and **2v** (oxaprozin) participate in *N*-aminopyridiylation to afford aziridines **5t**, **5u**, and **5v**, respectively. Similarly, natural product derivatives of geraniol (**2w** and **2x**), β-caryophyllene (**2y**), dehydroepiandrosterone (**2z**), and *R*-carvone (**2aa**) undergo aziridination in 58, 59, 84, 82, and 40% yields, respectively. For geraniol derivatives **5w** and **5x**, aziridination occurred selectively at the π-bond furthest from the electron-withdrawing substituent. Aziridination occurred at the endocyclic π-bond of **2y** to afford **5y**, and at the 1,2-disubstituted olefin in **2aa** in preference to the α,β-unsaturated site to afford **5aa**. Consistent with the hypothesized electrophilic iminoiodinane intermediate **1**, the aziridination favored electron-rich C=C bonds, as exemplified in compounds **5r**, **5w**, **5x**, **5y**, and **5aa**.

In addition to aziridination products, some substrates (*e.g.,* carvone and celecoxib derivatives) afforded imine byproducts (*vide infra*); for carvone, the imine byproduct (**5aa’**) was isolated in 18% yield. Regarding mass balance, triturating the crude mixture with Et_2_O, instead of purifying via column chromatography, can improve material recovery: For example, **5a** was isolated in 92% yield, **5p** in 90% yield, and 5**aa** (with **5aa’**) in 98% yield. Substrates that did not engage in efficient aziridination are summarized in Figure S1.

Finally, gaseous olefins can be converted to the corresponding *N*-pyridinium aziridines.^21^ Aziridination of ethylene (2 atm, excess with respect to **3**) affords **5ab** in 48% yield (Figure S3); aziridination of propylene and isobutylene afford the corresponding aziridines **5ac** and **5ad** in 38 and 35% yields, respectively. Aziridination of these gaseous olefins is accompanied by the formation of the corresponding imine byproducts, which together account for ∼90% of the mass balance.

From a mechanistic perspective, the *N-*aminopyridylation of unactivated olefins was developed based on the hypothesis that aziridination proceeded via highly electrophilic, cationic iminoiodinane **1**. Consistent with this hypothesis, putative iminoiodinane was observed by ESI+ mass spectrometry of mixtures of **3** and **4**. Attempts to isolate (or spectroscopically observe) intermediate **1** were unsuccessful, likely due to the inherent reactivity of **1**.

To evaluate the intermediacy of **1** and the potential for charge-enhanced hypervalent iodine reactivity, we carried out variable time normalization analysis (VTNA) of the aziridination of cyclohexene. In VTNA, the *x*-axis represents reaction time normalized by concentration raised to its reaction order.^22^ The reaction profile pictured in Figure 3a displays good agreement with the zeroth-order kinetic dependence on cyclohexene. Similar analyses for *N*-aminopyridinium salt **3** and PhIO (**4**) indicate first-order dependence on each of these components (Figure 3b and c, respectively). The rate constant (*k*_*obs*_) was determined to be 0.0118 M^–1^ s^–1^ by plotting the concentration of aziridine **5a** versus the normalized time abscissas for all reaction components (Figure 3d). The experimentally derived rate law, *r* = *k*_obs_[**2a**]^0^[**3**]^1^[**4**]^1^, suggests the rate-determining reaction of **3** with PhIO to generate iminoiodinane **1**, which was then quickly trapped by the olefin substrate. See Figures S4–S7 for additional VTNA plots.

**Figure 3:**
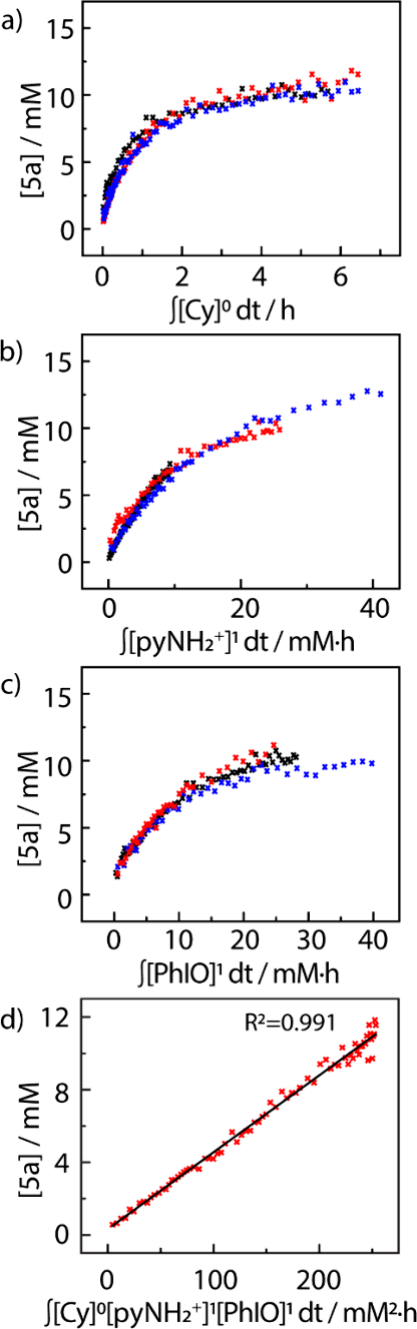
VTNA indicates aziridination is (a) 0th order in cyclohexene ([cyclohexene]: (black ×) 15 mM, (red ×) 30 mM, and (blue ×) 45 mM), (b) 1st order in *N*-aminopyridinium triflate ([pyNH_2_OTf]: (black ×) 10 mM, (red ×) 20 mM, and (blue ×) 30 mM), and (c) 1st order in PhIO ([PhIO]: (black ×) 20 mM, (red ×) 30 mM, and (blue ×) 40 mM). (d) Determination of rate constant; *k*_obs_= 0.0118 M^–1^ s^–1^.

Hammett analyses were carried out via VTNA to define the impact of both *N*-aminopyridinium and iodosylbenzene substitution on the rate of aziridination.^23^ The Hammett plot based on aziridination with *para*-substituted iodosylbenzene derivatives (Figure S8) displayed linear correlation (R^*2*^ = 0.835) with ρ = −0.3 (Figure S9) and the Hammett plot derived from substituted *N*-aminopyridinium salts (Figures S10 and S11) displayed linear correlation with ρ = −0.4 (R^2^ = 0.947, Figure S12). These data are consistent with accumulation of positive charge in the iodosylbenzene and loss of negative charge in the *N*-aminopyridinium as would be expected for the formation of *N*-pyridinium iminoiodinane **1**.

Charge-labeled iminoiodinane **1** is more reactive than PhINTs: An aziridination reaction using a mixture of **3** and TsNH_2_, which has similar p*K*_a_ with **3** and also engages in PhIO-promoted aziridination chemistry,^24^ afforded a 1.00:0.53 mixture of **5a** and **6** (Figure 4a). To further evaluate the aziridination mechanism, we carried out aziridination of **2a** in the presence of α-phenyl-*N*-*t*-butyl nitrone (PBN), a common radical trap, and obtained aziridine **5a** in 87% NMR yield (Figure 4b). In contrast, the addition of PBN completely suppressed the previously reported iodide-catalyzed chemistry. Finally, aziridination of radical clock **2ae** resulted in aziridine **7** in 83% yield with minimal byproduct formation (Figure 4c).^25^ Together, these experiments suggest that radical intermediates are unlikely in the described aziridination chemistry.

**Figure 4:**
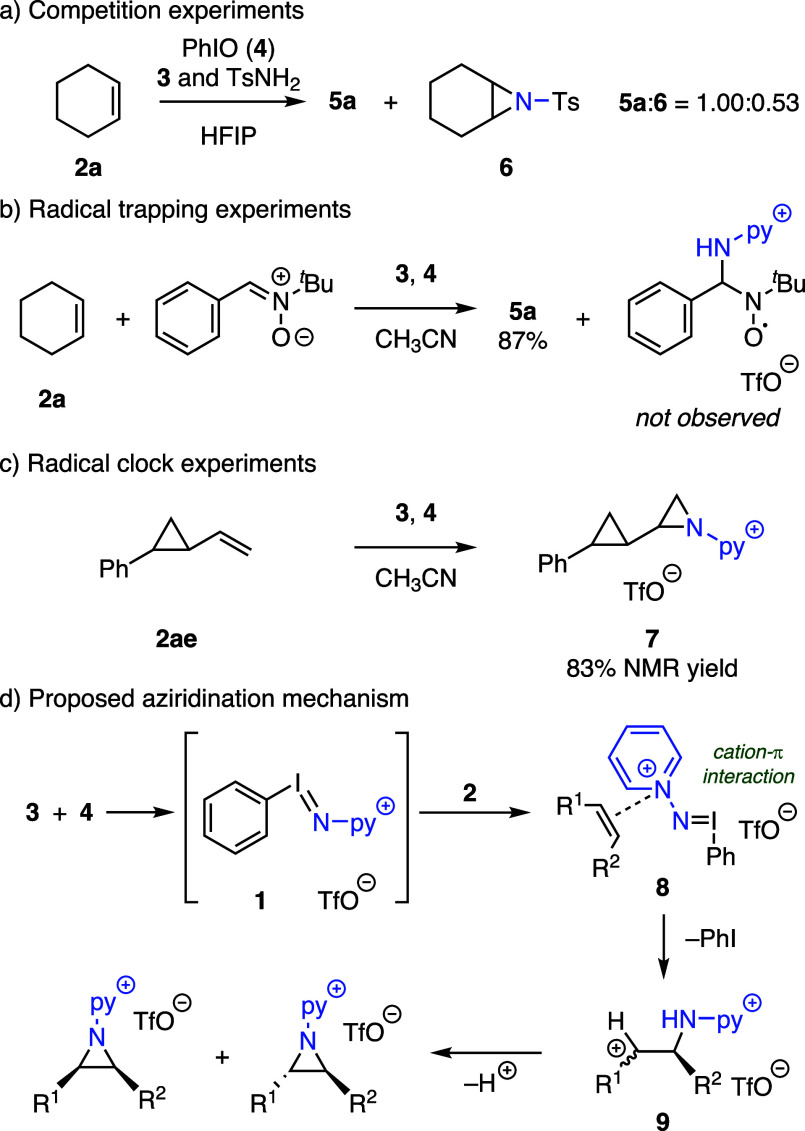
a) **1** is more reactive than PhINTs via competition experiments. b) Radical trapping experiments and c) radical clock experiments are inconsistent with a radical mechanism. d) Proposed reaction mechanism.

Together, the experimentally determined rate law, competition reactions, and radical trapping experiments indicate that the rate-determining reaction of PhIO with **3** generates highly reactive *N*-pyridinium iminoiodinane **1**. We speculate that **1** could subsequently combine with olefin **2** to form a cation-π-complex **8**.^26^ Formal reductive elimination of **8** would afford carbocation **9**, which would undergo rapid ring closure to the aziridine. The observed diastereomeric mixtures obtained from aziridination of *cis*-olefins **2c**, **2f**, and **2g** (*vide supra*) are consistent with the carbocation intermediate (i.e., **9**), which can undergo bond rotation affording *cis-*/*trans-* mixtures (Figure 4d). The observation of incomplete stereoablation in the aziridination of **2g** suggests either rapid ring closure without full rotational equilibration or competition between nonstereospecific mechanism and a stereospecific mechanism, such as concerted nitrene transfer from **1**. The formation of imine byproducts during the aziridination of some substrates pictured in Figure 2 further suggests a pinacol-type side reaction.

**Figure 5:**
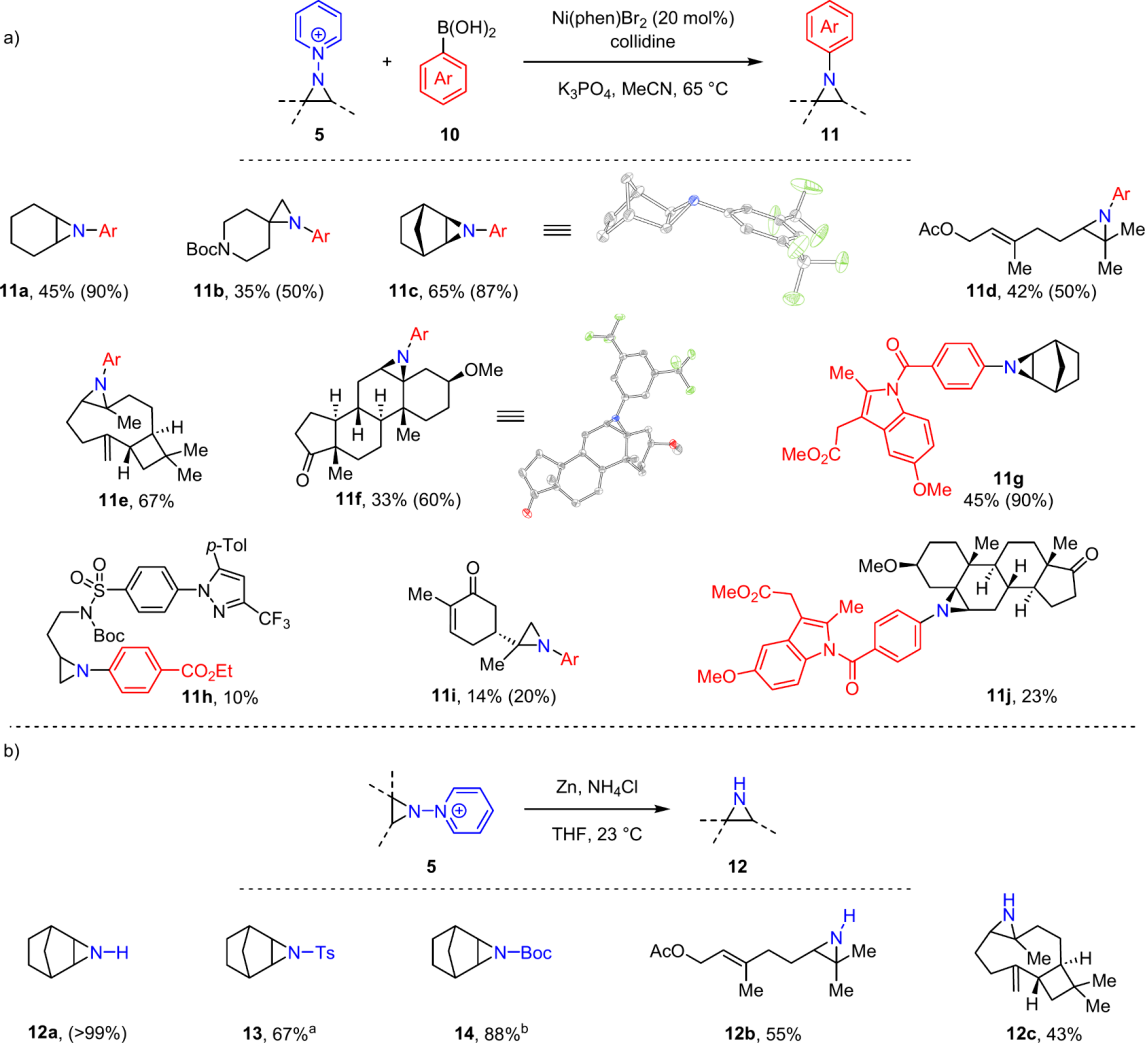
(a) Cross-coupling and (b) depyridylation of *N*-pyridinium aziridines. Conditions: **5** (1.0 equiv), **10** (2.4 equiv), Ni(phen)Br_2_ (20 mol %), 2,4,6-collidine (1.0 equiv). Depyridylation: **5** (1.0 equiv), I_2_ (5.0 mol %), Zn (10 equiv), NH_4_Cl (10 equiv). ^a^TsCl (1.50 equiv), K_2_CO_3_ (2.00 equiv), DMAP (10 mol %). ^b^Boc_2_O (2.00 equiv), Et_3_N (3.00 equiv). Ar = 3,5-Bis(trifluoromethyl)phenyl. Isolated yield (NMR yield).

With access to a large family of *N*-pyridinium aziridines (Figure 2), we sought to functionalize the exocyclic *N*-valence without an initial deprotection (Figure 5). Ni-catalyzed cross-coupling of *N*-pyridinium aziridines with aryl boronic acids^16a,27^ provides entry to *N*-arylaziridines. Simple pyridinium aziridines underwent cross-coupling with 3,5-bis(trifluoromethyl)phenyl boronic acid (**10a**) to afford *N*-aryl aziridines **11a** and **11b** in 90% and 50% yields (45 and 35% isolated yields, respectively). Norbornene-derived aziridine cross-coupled with **10a** to give *N*-aryl aziridine **11c** in 87% yield (65% isolated); SCXRD confirmed *exo*-stereochemistry. Pharmaceutical and natural product derivatives of pyridinium aziridines **5** engaged in cross-coupling: Pyridinium aziridines derived from geranyl acetate, β-caryophyllene, and dehydroepiandrosterone were cross-coupled with **10a** to afford *N*-aryl aziridines (**11d** to **11f**) in moderate to good yields. The stereochemistry of **11f** was confirmed by SCXRD. Indomethacin derived boronic acid **10b** engaged in coupling with **5e** in 90% yield (45% isolated yield, **11g**). Coupling efficiency is substrate dependent: Celecoxib and carvone derivatives **5t** and **5aa** afford *N*-aryl aziridines **11h** and **11i** in low yield. Coupling of dehydroepiandrosterone-derived aziridine **5z** and indomethacin-derived boronic acid **10b** afforded *N*-aryl aziridine conjugate **11j** in 23% yield.

In addition to C–N cross-coupling, depyridylation of **5** provides N–H aziridines (**12**) under mild conditions. For example, depyridylation of **5e** with Zn in the presence of NH_4_Cl afforded aziridine **12a** in quantitative NMR yield. For isolation, **12a** was treated with either TsCl or Boc_2_O to afford protected aziridines **13** and **14**, respectively. Pyridinium aziridines **5w** and **5y** smoothly underwent deprotection to afford the corresponding aziridines **12b** and **12c** in moderate isolated yields.

In summary, the aziridination of unactivated alkenes with *N*-aminopyridinium salts is demonstrated. Kinetics studies indicate that aziridination proceeds via a highly electrophilic, charge-labeled *N*-pyridinium iminoiodinane. The obtained *N*-pyridinium aziridines can be derivatized to afford both *N*-aryl and N–H aziridines. These studies advance new methods for the aziridination of unactivated olefins, broaden the scope of *build-and-couple* aziridine synthesis via *N*-pyridinium aziridine intermediates, and introduce charge-enhanced electrophilicity into iminoiodinane chemistry.

## Data Availability

The data underlying this study are available in the published article and its Supporting Information.^28^

## Supporting Information Available

Experimental methods, optimization data, spectral data and experimental spectra, diffraction data (PDF)
Crystllographic data (CIF)

## Supplementary Material

au4c00556_si_001.pdf

au4c00556_si_002.cif
