# Supplementary material for: Metal-Free Aziridination of Unactivated Olefins via Transient N-Pyridinium Iminoiodinanes
Source: JACS Au. 2024 Oct 10;4(11):4187–93. doi: 10.1021/jacsau.4c00556 (PMC11600189; doi:10.1021/jacsau.4c00556)
Supplement: Supplementary file 1 — au4c00556_si_001.pdf [file au4c00556_si_001.pdf]

Supporting Information

Metal-Free Aziridination of Unactivated Olefins via Transient *N*-Pyridinium Iminoiodinanes

Hao Tan, Phong Thai, Uddalak Sengupta, Isaac R. Deavenport, Cali M. Kucifer, and David C. Powers*

[a]*Department of Chemistry, Texas A&M University, College Station, TX 77843, USA*

Email: powers@chem.tamu.edu

Table of Contents

A. General Considerations **S3**

A.1 Materials S3

A.2 Characterization Details S3

B. Synthesis and Characterization **S5**

B.1 Preparation of Aliphatic Olefins S5

B.2 Synthesis of *N*-Aminopyridinium Salts S8

B.3 Synthesis of Iodosylbenzene Derivatives S12

B.4 Aziridination of Aliphatic Olefins S13

B.5 Cross-Coupling of Pyridinium Aziridines S25

B.6 Deprotection of Pyridinium Aziridines S29

C. Reaction Optimization Studies **S31**

C.1 Optimization of Aliphatic Olefin Aziridination S31

C.2 Problematic Olefin Substrates S36

C.3 Aziridination of Cyclooctadiene S37

C.4 Aziridination of Ethylene Gas S38

D. Kinetics Analysis of Mechanism **S39**

E. Hammett Analyses **S47**

F. Chemical Analysis of Mechanism **S52**

F.1 Mass Spectrometry Evidence of Iminoiodinane S52

F.2 Competition Aziridination Altering Nitrogen Sources S53

F.3 Aziridination with Kinetic Probe Substrates S54

F.4 Decomposition Kinetics of PhIO and *N*-Aminopyridinium Triflate S58

F.5 Alternative Aziridination Mechanism S60

G. X-Ray Diffraction Data **S61**

H. NMR Spectra for New Compounds **S73**

I. Supplementary References **S164**

A. General Considerations

A.1 Materials All chemicals and solvents were obtained as ACS reagent grade and used as received. Hydroxylamine-*O*-sulfonic acid, dehydroepiandrosterone, and 4-ethoxylcarbonylphenylboronic acid (**10c**) were purchased from Matrix. Pyridine, potassium carbonate, methyl *tert*-butyl ether, and vinylcyclohexane (**2n**) were purchased from BTC. Potassium phosphate, trifluoroacetic acid, cyclohexene (**2a**), 1-octene (**2h**), allylbenzene (**2o**), but-3-en-1-ylbenzene (**2p**), iodomethane, ethylene (**2ab**), propylene (**2ac**), 1,3,5-trimethoxybenzene, and triethyl benzene-1,3,5-tricarboxylate were purchased from Sigma Aldrich. Trifluoromethanesulfonic aicd, mesitylsulfonyl chloride, *tert*-butyl-*N*-hydroxycarbamate, 4-bromo-1-butene, 6-bromo-1-hexene, ibuprofen, celecoxib, oxaprozin, benzoic acid, 6-chloro-1-hexene (**2i**), di-*tert*-butyl decarbonate, and isobutylene (**2ad**) were purchased from Oakwood. Geraniol, cyclooctene (**2c**), and norbornene (**2e**) were purchased from Alfa Aesar. Cyclopentene (**2b**), (+)-δ3-carene (**2d**), and *cis*-cyclooctadiene (**2f**) were purchased from TCI. *tert*-Butyl 4-methylenepiperidine-1-carboxylate (**2s**), β-caryophyllene (**2y**), (R)-carvone (**2aa**), and 3,5-bis(trifluoromethyl)benzeneboronic acid (**10a**) were purchased from Ambeed. 1,1,1,3,3,3-Hexafluoro-2-propanol (HFIP) was refluxed for 12 h under N_2 over 3 Å molecular sieves, distilled, and stored in a Straus flask.[1] Dry dichloromethane and acetonitrile (purchased from Fisher scientific, HPLC grade) were obtained from a drying column and stored over activated 4 Å molecular sieves.[2] NMR solvents were purchased from Cambridge Isotope Laboratories and were used as received. All reactions were carried out under an ambient atmosphere unless otherwise noted.

Hex-5-en-1-yl benzoate (**2j**),[3] 6-azidohex-1-ene (**2l**),[4] pent-4-en-1-ylbenzene (**2q**),[5] 1-(but-3-en-1-yl)-4-vinylbenzene (**2r**),[6] (*E*)-3,7-dimethylocta-2,6-dien-1-yl acetate (**2w**),[7] (*E*)-2-(3,7-dimethylocta-2,6-dien-1-yl)-3a,7a-dihydro-1*H*-isoindole-1,3(2*H*)-dione (**2x**),[7] *tert*-butyl ((4-(5-(*p*-tolyl)-3-(trifluoromethyl)-1H-pyrazol-1-yl)phenyl)sulfonyl)carbamate (**S4**), [8] (4-bromophenyl)- λ³-iodanediyl diacetate (**S5a**),[9] (4-cyanophenyl)- λ³-iodanediyl diacetate (**S5b**),[9] (4-nitrophenyl)- λ³-iodanediyl diacetate (**S5c**),[9] and iodosyl-4-methylbenzene (**S6d**)[10] were synthesized according to literature methods. was prepared from celecoxib according to a literature procedure. Iodosyl-4-methoxybenzene (**S6e**) was synthesized according to a reported procedure[10] from (4-methoxyphenyl)-λ³-iodanediyl diacetate, which was prepared via a literature method.[11]

A.2 Characterization Details ^{1}H and ^{13}C NMR spectra were recorded on an Inova 500 FT NMR (Varian), an Acsend™ 400 NMR (Bruker), or an Acsend™ 400 NMR (Bruker) and were referenced against residual proteo solvent signals: $CDCl_3$ (7.26 ppm, ^{1}H; 77.16 ppm, ^{13}C), CD_3OD (3.31 ppm, ^{1}H; 49.00 ppm, ^{13}C), $(CD_3)_2SO$ (2.50 ppm, ^{1}H), and acetonitrile-d_3 (1.94 ppm, ^{1}H; 1.32 ppm, ^{13}C).[12] ^{1}H NMR data are reported as follows: chemical shift (δ, ppm), (multiplicity: s (singlet), d (doublet), t (triplet), m (multiplet), br (broad), integration). ^{13}C NMR data are reported as follows: chemical shift (δ, ppm). Mass spectrometry data were recorded on either Orbitrap Fusion™ Tribrid™ Mass Spectrometer or Q Exactive™ Focus Hybrid Quadrupole-Orbitrap™ Mass Spectrometer from ThermoFisher Scientific.

For single-crystal X-ray diffraction analysis of *t*-**5f**, **5aa**, **11c**, and **11f**, a Bruker APEX 2 Duo X-ray (three-circle) diffractometer was used for crystal screening, unit cell determination,

and data collection. A crystal suitable for X-ray diffraction was mounted on a MiTeGen dual-thickness micro-mount and placed under a cold N_2 stream (Oxford). The X-ray radiation employed was generated from a Mo sealed X-ray tube (K_α = 0.70173 Å with a potential of 40 kV and a current of 40 mA). Bruker AXS APEX II software was used for data collection and reduction. Absorption corrections were applied using SADABS. A solution was obtained using XT/XS in APEX2 and refined in Olex2.[13-15] Hydrogen atoms were placed in idealized positions and were set riding on the respective parent atoms. All non-hydrogen atoms were refined with anisotropic thermal parameters. The structure was refined (weighted least squares refinement on F^2) to convergence.[15]

B. Synthesis and Characterization

B.1 Preparation of Aliphatic Olefins

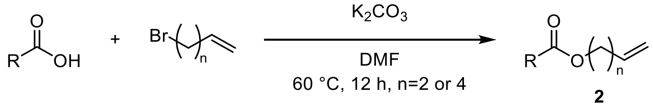

2

General Procedure

A 20-mL scintillation vial was charged with the appropriate carboxylic acid (1.50 mmol, 1.00 equiv.), K_2CO_3 (311 mg, 2.25 mmol, 1.50 equiv.), and DMF (10.0 mL). The appropriate bromoolefin (1.65 mmol, 1.10 equiv.) was added and the reaction was stirred at 60 °C for 12 h. The reaction mixture was cooled to 23 °C and water (15 mL) was added. The mixture was extracted with EtOAc (3 × 10 mL). The combined organic layers were washed with H_2O (4 × 10 mL), washed with brine (30 mL), dried over Na_2SO_4, and concentrated under reduced pressure. The crude mixture was purified by silica gel flash chromatography to afford the corresponding aliphatic olefin (**2**).

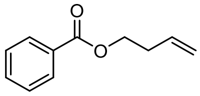

2m

*But-3-en-1-yl benzoate (**2m**)*. Prepared via the general procedure from benzoic acid (800 mg, 6.55 mmol), and obtained as a colorless oil (1.13 g, 6.41 mmol, 98%). ^{1}H NMR (400 MHz, $CDCl_3$) δ 8.04 (d, *J* = 7.0 Hz, 2H), 7.56 (t, *J* = 7.4 Hz, 1H), 7.44 (t, *J* = 7.6 Hz, 2H), 5.88 (ddt, *J* = 17.0, 10.2, 6.7 Hz, 1H), 5.30 – 4.95 (m, 2H), 4.38 (t, *J* = 6.7 Hz, 2H), 2.53 (qt, *J* = 6.7, 1.4 Hz, 2H). These spectral data are well-matched to those reported in the literature.[21]

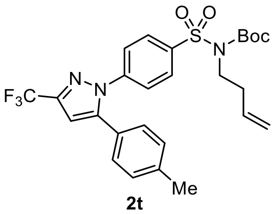

2t

*tert-Butyl but-3-en-1-yl((4-(5-(p-tolyl)-3-(trifluoromethyl)-1H-pyrazol-1-yl)phenyl)sulfonyl) carbamate (**2t**)*. Prepared via the general procedure from *N*-Boc-celecoxib **S4** (1.50 mmol) and obtained as a white solid (743 mg, 1.39 mmol, 92%). ^{1}H NMR (400 MHz, $CDCl_3$) δ 7.90 (d, *J* = 8.8 Hz, 2H), 7.46 (d, *J* = 8.8 Hz, 2H), 7.17 (d, *J* = 7.9 Hz, 2H), 7.10 (d, *J* = 8.2 Hz, 2H), 6.74 (s, 1H), 5.79 (ddt, *J* = 17.1, 10.2, 7.0 Hz, 1H), 5.32–4.96 (m, 2H), 3.96–3.79 (m, 2H), 2.48 (q, *J* = 7.2 Hz, 2H), 2.38 (s, 3H), 1.36 (s, 9H). ^{19}F NMR (377 MHz, $CDCl_3$) δ –62.5. ^{13}C NMR (126 MHz, $CDCl_3$) δ 150.8, 145.4, 144.3 (q, *J* = 38.6 Hz), 143.0, 139.9, 139.7, 134.3, 129.9, 129.1,

128.9, 125.9, 125.0, 121.2 (q, J = 269.2 Hz), 117.8, 106.6, 84.7, 46.7, 34.7, 28.0, 21.4. HRMS-ESI$^+$ (m/z): [M+1]$^+$ calcd. for $C_{26}H_{29}F_3N_3O_4S^+$, 536.1825; found, 536.1817.

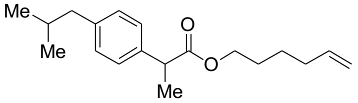

2u

*Hex-5-en-1-yl 2-(4-isobutylphenyl)propanoate (**2u**)*. Prepared via the general procedure from ibuprofen (1.00 g, 4.85 mmol), and obtained as a colorless oil (1.30 g, 4.51 mmol, 93%). ^{1}H NMR (400 MHz, $CDCl_3$) δ 7.20 (d, J = 8.1 Hz, 2H), 7.09 (d, J = 8.1 Hz, 2H), 5.74 (ddt, J = 16.9, 10.2, 6.7 Hz, 1H), 5.12–4.73 (m, 2H), 4.06 (t, J = 6.6 Hz, 2H), 3.68 (q, J = 7.2 Hz, 1H), 2.44 (d, J = 7.2 Hz, 2H), 2.08–1.95 (m, 2H), 1.84 (dp, J = 13.6, 6.8 Hz, 1H), 1.59 (dq, J = 8.6, 6.6 Hz, 2H), 1.49 (d, J = 7.2 Hz, 3H), 1.35 (tt, J = 9.8, 6.4 Hz, 2H), 0.90 (d, J = 6.6 Hz, 6H). These spectral data are well-matched to those reported in the literature.[3]

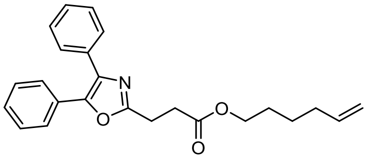

2v

*Hex-5-en-1-yl 3-(4,5-diphenyloxazol-2-yl)propanoate (**2v**)*. Prepared via the general procedure from oxaprozin (587 mg, 2.00 mmol), and obtained as a colorless oil (727 mg, 1.94 mmol, 97%). ^{1}H NMR (400 MHz, $CDCl_3$) δ 7.60 (ddd, J = 23.9, 8.2, 1.6 Hz, 4H), 7.41–7.27 (m, 6H), 5.76 (ddt, J = 16.9, 10.2, 6.7 Hz, 1H), 5.14–4.83 (m, 2H), 4.13 (t, J = 6.6 Hz, 2H), 3.19 (t, J = 7.5 Hz, 2H), 2.91 (t, J = 7.5 Hz, 2H), 2.11–2.00 (m, 2H), 1.72–1.59 (m, 2H), 1.45 (tt, J = 10.1, 6.3 Hz, 2H). These spectral data are well-matched to those reported in the literature.[22]

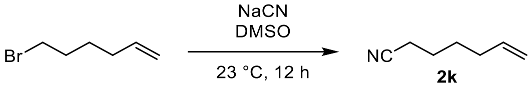

2k

*Hept-6-enenitrile (**2k**).* To NaCN (150 mg, 3.06 mmol, 1.02 equiv.) in a 50-mL round bottom flask was added DMSO (10.0 mL), followed by 6-bromohex-1-ene (489 mg, 3.00 mmol, 1.00 equiv.). The mixture was stirred for 12 h, after which TLC monitoring indicated completion of the reaction (100% hexanes, visualized by permanganate stain). Dilute aqueous NaOH (10 mL) was added. The mixture was extracted with CH_2Cl_2 (3 × 10 mL). The combined organic layers were washed with H_2O (4 × 10 mL), followed by brine (20 mL), dried over Na_2SO_4 and concentrated under reduced pressure. The crude mixture was filtered through a silica plug to afford the title compound as a colorless oil (270 mg, 2.46 mmol, 82%). ^{1}H NMR (400 MHz, $CDCl_3$) δ 5.77 (ddt, J = 16.9, 10.2, 6.7 Hz, 1H), 5.06–5.01 (m, 1H), 5.01–4.97 (m, 1H), 2.35 (t, J = 7.0 Hz, 2H), 2.17–2.02 (m, 2H), 1.73–1.61 (m, 2H), 1.60–1.50 (m, 2H). These spectral data are well-matched to those reported in the literature.[23]

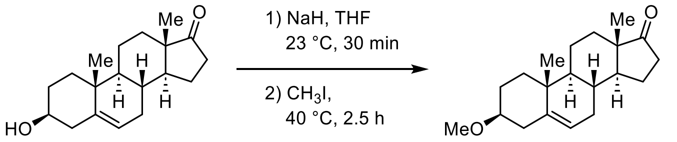

2z

*O-Methyl dehydroepiandrosterone (**2z**).*[24] In an N_2-filled glovebox, NaH dispersed in mineral oil was washed with pentane and dried. A 200-mL Schlenk flask was charged with dehydroepiandrosterone (2.32 g, 8.04 mmol, 1.00 equiv.), and anhydrous THF (50 mL). NaH (220 mg, 9.16 mmol, 1.14 equiv.) was added and the resulting mixture was stirred under N_2 at 23 °C for 30 min to afford an orange-colored mixture. The reaction vessel was removed from the glovebox. Iodomethane (5.69 g, 2.50 mL, 40.1 mmol, 5.00 equiv.) was added dropwise and the resulting mixture was heated to 40 °C and stirred for 2.5 h. The reaction was cooled to 23 °C, water (150 mL) was added, and the mixture was extracted with EtOAc (3 × 50 mL). The combined organic layer was washed with brine, dried over Na_2SO_4, and concentrated under reduced pressure. The crude mixture was purified by silica gel flash chromatography using EtOAc : hexanes (1 : 10) as the eluent to afford the title compound as a white solid (3.58 g, 3.61 mmol, 45% yield). ^1H NMR (500 MHz, $CDCl_3$) δ 5.39 (d, J = 5.4 Hz, 1H), 3.36 (s, 3H), 3.07 (tt, J = 11.3, 4.5 Hz, 1H), 2.54–2.32 (m, 2H), 2.23–2.02 (m, 3H), 2.01–1.79 (m, 4H), 1.73–1.60 (m, 3H), 1.61–1.37 (m, 3H), 1.34–1.23 (m, 2H), 1.11–0.96 (m, 5H), 0.89 (s, 3H). These spectral data are well-matched to those reported in the literature.[25]

B.2 Synthesis of *N*-Aminopyridinium Salts

Synthesis of *N*-Aminopyridinium Triflate (3)

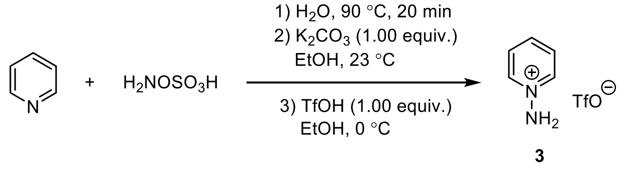

N-Aminopyridinium triflate (**3**) was prepared according to the following modification of literature methods.[16] A 250-mL round-bottom flask was charged with a freshly prepared solution of hydroxylamine-*O*-sulfonic acid (11.3 g, 100. mmol, 1.00 equiv.) in H_2O (64 mL). Pyridine (24.0 mL, 300. mmol, 1.00 equiv.) was added. The reaction was heated at 90 °C for 20 min before being cooled to 23 °C. With vigorous stirring, potassium carbonate (13.8 g, 100. mmol, 1.00 equiv.) was added to the reaction vessel, and the mixture was stirred for 5 min. Volatiles were removed under reduced pressure and 120 mL of ethanol was added. Solids were removed by filtration. The filtrate was cooled to 0 °C and TfOH (15.0 g, 8.80 mL, 100. mmol, 1.00 equiv.) was added dropwise. The solution was concentrated under reduced pressure until about 20 mL was left in the flask. The concentrated solution was cooled to –20 °C to induce crystallization. The precipitate was filtered and dried under vacuum to give the title compound **3** as an off-white solid (16.5 g, 67.6 mmol, 67% yield). ^1H NMR (400 MHz, CD_3CN) δ 8.57 (d, J = 6.2 Hz, 2H), 8.28 (t, J = 7.7 Hz, 1H), 7.92 (t, J = 7.3 Hz, 2H), 7.11 (s, 2H). ^{19}F NMR (377 MHz, CD_3CN) δ –79.1. These spectral data are well-matched to those reported in the literature.[16]

Synthesis of *O-(mesitylsulfonyl)hydroxylamine* (S2)

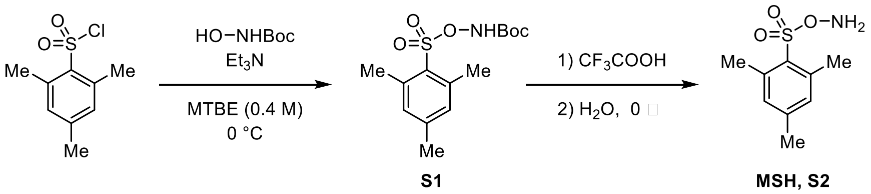

tert-Butyl-((mesitylsulfonyl)oxy)carbamate (**S1**) was prepared according to the following modification of literature methods.[17] A 100-mL round-bottom flask charged with mesitylenesulfonyl chloride (2.19 g, 10.0 mmol, 1.00 equiv.), *tert*-butyl-*N*-hydroxycarbamate (1.33 g, 10.0 mmol, 1.00 equiv.), and methyl *tert*-butyl ether (MTBE, 25 mL). The mixture was sparged with N_2 and cooled to 0 °C. Triethylamine (1.39 mL, 10.0 mmol, 1.00 equiv.) was added dropwise with stirring after which the reaction was stirred for 2 h.[a] The mixture was filtered, the obtained solids were washed with MTBE, and the filtrate was concentrated under reduced pressure. Hexanes were then added, and the resulting precipitate was collected by filtration. The obtained solid was dried under vacuum at 23 °C to afford compound **S1** as a white solid (2.70 g, 8.60 mmol, 86%). ^1H NMR (400 MHz, $CDCl_3$) δ 7.51 (s,

[a] Reaction progress was monitored by thin-layer chromatography (TLC) using a 7 : 3 hexanes : ethyl acetate mobile phase.

1H), 6.99 (s, 2H), 2.68 (s, 6H), 2.32 (s, 3H), 1.32 (s, 9H). These spectral data are well-matched to those reported in the literature.[17]

A 100-mL round-bottom flask was charged with trifluoroacetic acid (7.9 mL, 0.10 mol, 12 equiv.), and was cooled to 0 °C. *tert*-Butyl ((mesitylsulfonyl)oxy)carbamate (**S1**, 2.70 g, 8.57 mmol, 1.00 equiv.) was added to the reaction vessel in 3 portions over 20 min. The reaction was stirred at 0 °C for 90 min.[b] Crushed ice was added followed by water (15 mL). After 15 min, the obtained precipitate was collected by filtration, washed with water until the pH of the wash measured 7 by litmus, and dried to afford **MSH** (**S2**) as a white solid (3.14 g, 146%)[c] and stored below 0 °C. This was used without further drying or purification. ^{1}H NMR (400 MHz, DMSO-d_6) δ 6.75 (s, 2H), 2.49 (s, 6H), 2.17 (s, 3H). These spectral data are well-matched to those reported in the literature.[17]

Synthesis of *N*-aminopyridinium 2,4,6-trimethylbenzenesulfonates (S3)

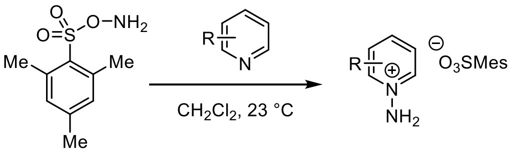

MSH, S2 **S3**

N-Aminopyridinium 2,4,6-trimethylbenzenesulfonate (**S3**) was prepared according to the following modification of literature methods.[18] A 20-mL scintillation vial was charged with MSH (**S2**, 0.60 mmol, 1.0 equiv.) and CH_2Cl_2 (4.0 mL). The appropriate pyridine derivative (0.60 mmol, 1.0 equiv.) was added. The mixture was stirred for 30 min at 23 °C and concentrated under reduced pressure. The desired *N*-aminopyridinium salts (**S3**) were purified by crystallization from MeOH/Et_2O. Characterization data are collected below.

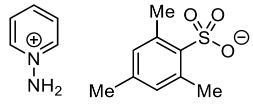

S3a

*1-Aminopyridin-1-ium 2,4,6-trimethylbenzenesulfonate (**S3a**).* Prepared from pyridine (0.600 mmol), and obtained as a white solid (34.6 mg, 0.120 mmol, 20%). ^{1}H NMR (400 MHz, DMSO) δ 8.77 (d, *J* = 6.2 Hz, 2H), 8.47 (s, 2H), 8.27 (t, *J* = 7.9 Hz, 1H), 8.01 (t, *J* = 7.1 Hz, 2H), 6.74 (s, 2H), 2.49 (s, 6H), 2.17 (s, 3H). These spectral data are well-matched to those reported in the literature.[19]

---

[b] Reaction progress was monitored by thin-layer chromatography (TLC) using an 8 : 2 hexanes : ethyl acetate mobile phase.

[c] **CAUTION!! Caution is needed for handling MSH. MSH has been reported to be unstable and potentially explosive when dry. The >100% apparent yield is due to the water content of the wet MSH. CAUTION!!**

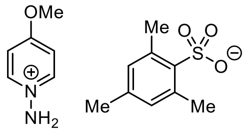

S3b

*1-Amino-4-methoxypyridin-1-ium 2,4,6-trimethylbenzenesulfonate (**S3b**)*. Prepared from 4-methoxypyridine (0.400 mmol), and obtained as a white solid (65.0 mg, 0.200 mmol, 50%). ^{1}H NMR (500 MHz, $CDCl_3$) δ 8.86 (d, *J* = 7.6 Hz, 2H), 8.14 (s, 2H), 7.06 (d, *J* = 7.6 Hz, 2H), 6.80 (s, 2H), 3.95 (s, 3H), 2.61 (s, 6H), 2.22 (s, 3H). These spectral data are well-matched to those reported in the literature.[20]

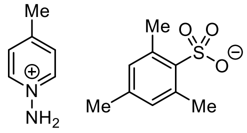

S3c

*1-Amino-4-methylpyridin-1-ium 2,4,6-trimethylbenzenesulfonate (**S3c**)*. Prepared from 4-methylpyridine (0.600 mmol), and obtained as an off-white solid (67.5 mg, 0.216 mmol, 36%). ^{1}H NMR (400 MHz, DMSO) δ 8.64 (d, *J* = 6.8 Hz, 2H), 8.22 (s, 2H), 7.83 (d, *J* = 6.5 Hz, 2H), 6.74 (s, 2H), 2.52 (s, 3H), 2.49 (s, 6H), 2.17 (s, 3H). These spectral data are well-matched to those reported in the literature.[19]

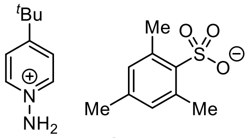

S3d

*1-Amino-4-(tert-butyl)pyridin-1-ium 2,4,6-trimethylbenzenesulfonate (**S3d**)*. Prepared from 4-(tert-butyl)pyridine (0.600 mmol), and obtained as a colorless oil (130 mg, 0.372 mmol, 62%). ^{1}H NMR (400 MHz, DMSO) δ 8.68 (d, *J* = 7.1 Hz, 2H), 8.28 (s, 2H), 8.02 (d, *J* = 7.1 Hz, 2H), 6.74 (s, 2H), 2.49 (s, 6H), 2.17 (s, 3H), 1.33 (s, 9H). ^{13}C NMR (101 MHz, DMSO) δ 164.1, 142.8, 138.2, 136.2, 135.9, 129.8, 125.0, 35.7, 29.6, 22.7, 20.3. HRMS-ESI^{+} (m/z): [M]$^{+}$ calcd. for $C_9H_{15}N_2^{+}$, 151.1230; found, 151.1227.

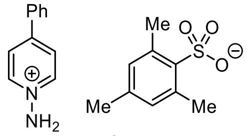

S3e

*1-Amino-4-phenylpyridin-1-ium 2,4,6-trimethylbenzenesulfonate (**S3e**)*. Prepared from 4-phenylpyridine (0.600 mmol), and obtained as a yellow solid (128 mg, 0.348 mmol, 58%). ^{1}H NMR (400 MHz, DMSO) δ 8.81 (d, *J* = 7.1 Hz, 2H), 8.48 (s, 2H), 8.36 (d, *J* = 7.1 Hz, 2H), 8.07–7.85 (m, 2H), 7.69–7.33 (m, 3H), 6.74 (s, 2H), 2.51 (s, 6H), 2.15 (s, 3H). ^{13}C NMR (101 MHz, DMSO) δ 149.4, 142.6, 138.5, 136.4, 135.9, 133.7, 131.3, 129.9, 129.6, 127.6, 124.6, 22.7, 20.3. HRMS-ESI^{+} (m/z): [M]$^{+}$ calcd. for $C_{11}H_{11}N_2^{+}$, 171.0917; found, 171.0914.

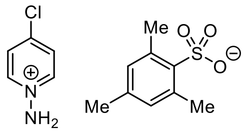

S3f

*1-Amino-4-chloropyridin-1-ium 2,4,6-trimethylbenzenesulfonate (**S3f**)*. Prepared from 4-chloropyridine hydrochloric acid[d] (0.600 mmol), and obtained as a yellow solid (29.7 mg, 0.0900 mmol, 15%). ^{1}H NMR (400 MHz, DMSO) δ 8.76 (d, *J* = 7.3 Hz, 2H), 8.52 (s, 2H), 8.17 (d, *J* = 7.2 Hz, 2H), 6.74 (s, 2H), 2.49 (s, 6H), 2.17 (s, 3H). These spectral data are well-matched to those reported in the literature.[19]

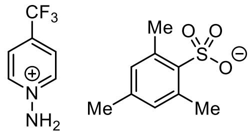

S3g

*1-Amino-4-(trifluoromethyl)pyridin-1-ium 2,4,6-trimethylbenzenesulfonate (**S3g**)*. Prepared from 4-(trifluoromethyl)pyridine (0.600 mmol) and obtained as a white solid (131 mg, 0.360 mmol, 60%).^{1}H NMR (500 MHz, DMSO) δ 8.88 (s, 2H), 8.69 (d, *J* = 6.5 Hz, 2H), 8.14 (d, *J* = 6.7 Hz, 2H), 6.48 (s, 2H), 2.23 (s, 6H), 1.90 (s, 3H). ^{19}F NMR (377 MHz, DMSO) δ –63.3. ^{13}C NMR (126 MHz, DMSO) δ 142.5, 138.0, 136.4, 136.1 (q, *J* = 35.5 Hz), 135.9, 129.9, 124.9 (q, *J* = 3.7 Hz), 121.6 (q, *J* = 273.8 Hz), 22.7, 20.3. HRMS-ESI^{+} (m/z): [M]$^{+}$ calcd. for $C_6H_6F_3N_2^+$, 163.0478; found, 163.0474.

---

[d] NaOH (1.0 M, 5 mL) was added to 4-chloropyridine hydrochloric acid (90.1 mg), and the mixture was stirred for 5 min, which was then extracted with CH_2Cl_2 (3 × 3 mL). The obtained solution was used in the reaction without further purification.

B.3 Synthesis of Iodosylbenzene Derivatives

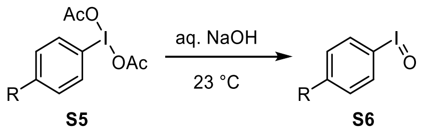

A 2-dram vial was charged with iodobenzene diacetate derivative (**S5**, 0.500 mmol, 1.00 equiv.), NaOH (600 mg, 15.0 mmol, 30.0 equiv.), and water (5.0 mL) at 23 °C. The vial was capped and shaken vigorously for 5 min. The resulting solid was isolated by filtration, washed with ether (2 × 1.5 mL) and water (6 × 1.5 mL), dried *in vacuo*, and stored in the dark at 9 °C.

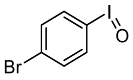

S6a

*1-Bromo-4-iodosylbenzene (**S6a**).* Prepared from (4-bromophenyl)-λ^3-iodanediyl diacetate (**S5a**, 149 mg) and obtained as a pale-yellow solid (120 mg, 0.401 mmol, 80% yield). ^{1}H NMR (δ, 23 °C, 400 MHz, CD_3OD): 7.94 (d, *J* = 8.2 Hz, 2H), 7.73 (d, *J* = 8.7 Hz, 2H). The spectroscopic data is in good agreement with that reported in the literature.[30]

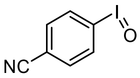

S6b

*4-Iodosylbenzonitrile (**S6b**).* Prepared from (4-cyanophenyl)-λ^3-iodanediyl diacetate (**S5b**, 174 mg) and obtained as a pale-yellow solid (111 mg, 0.453 mmol, 91% yield). ^{1}H NMR (δ, 23 °C, 400 MHz, CD_3OD): 8.19 (d, *J* = 8.7 Hz, 2H), 7.93 (d, *J* = 8.8 Hz, 2H). The spectroscopic data is in good agreement with that reported in the literature.[30]

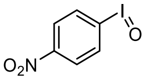

S6c

*1-Iodosyl-4-nitrobenzene (**S6c**).* Prepared from (4-cyanophenyl)-λ^3-iodanediyl diacetate (**S5c**, 184 mg) and obtained as an orange solid (110 mg, 0.415 mmol, 83% yield). ^{1}H NMR (δ, 23 °C, 400 MHz, CD_3OD): 8.40 (d, *J* = 9.2 Hz, 2H), 8.26 (d, *J* = 9.1 Hz, 2H). The spectroscopic data is in good agreement with that reported in the literature.[31]

B.4 Aziridination of Aliphatic Olefins

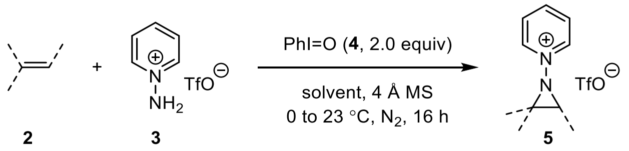

Procedure A

A 25-mL Schlenk tube was charged with *N*-aminopyridinium triflate (**3**, 0.300 mmol, 73.2 mg, 1.00 equiv.), the appropriate olefin (**2**, 0.300 mmol, 1.00 equiv.), and 4 Å molecular sieves. Dry HFIP (1.0 mL) was added under N_2 and the mixture was cooled to 0 °C. A 20-mL scintillation vial was charged with iodosylbenzene (**4**, 0.600 mmol, 132 mg, 2.00 equiv.) and HFIP (2.0 mL) under N_2. The HFIP solution of iodosylbenzene was then added dropwise to the Schlenk tube at 0 °C. The resulting mixture was allowed to warm to 23 °C and stirred for 12 h. The reaction was filtered, the obtained solids washed with CH_2Cl_2 (3 × 1.0 mL), and the combined filtrate was concentrated under reduced pressure. The crude was purified by silica gel flash chromatography, using EtOAc, CH_2Cl_2, 3% (v/v) MeOH/CH_2Cl_2, then 5% (v/v) MeOH/CH_2Cl_2 as the eluent to afford the title compound.

Procedure B

A 25-mL Schlenk tube was charged with iodosylbenzene (**4**, 0.600 mmol, 132 mg, 2.00 equiv.), 4 Å molecular sieves, and dry CH_3CN (1.0 mL) under N_2 and the mixture was cooled to 0 °C. A 20-mL scintillation vial was charged with *N*-aminopyridinium triflate (**3**, 0.300 mmol, 73.2 mg, 1.00 equiv.), the corresponding olefin (**2**, 0.300 mmol, 1.00 equiv.), and dry CH_3CN (2.0 mL) under N_2. This solution was then added dropwise to the Schlenk tube, and the mixture was allowed to warm to 23 °C and stirred for 12 h. The reaction was filtered, the obtained solids washed with CH_3CN (3 × 1.0 mL), and the combined filtrate was concentrated under reduced pressure. The crude was purified by silica gel flash chromatography using EtOAc, CH_2Cl_2, 3% (v/v) MeOH/CH_2Cl_2, then 5% (v/v) MeOH/CH_2Cl_2 as the eluent to afford the title compound.

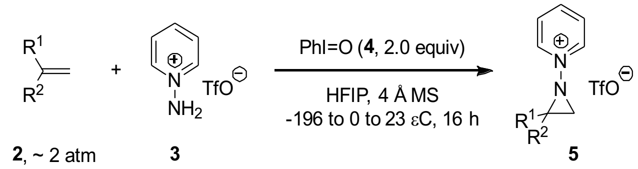

Procedure C (aziridination of gaseous olefins)

A 50-mL Schlenk flask with a rubber septum was evacuated and the head space filled with the appropriate gaseous olefin (**2**), supplied from an inflated balloon.

A 25-mL Schlenk tube was charged with *N*-aminopyridinium triflate (**3**, 0.200 mmol, 44.8 mg, 1.00 equiv.), 4 Å molecular sieves, and dry HFIP (1.0 mL) under N_2. The mixture was cooled to –78 °C. At this temperature, an HFIP solution (1.0 mL) of iodosylbenzene (**4**, 0.400 mmol, 88.0 mg, 2.00 equiv.) was slowly added. The Schlenk tube was then frozen ($N_{2(l)}$ cooling bath) and the head space was evacuated. The gaseous olefin was transferred to the reaction flask (maintained at –196 °C) via a rubber hose connection. The mixture was warmed to 23 °C, at which temperature it was stirred for 16 h. The reaction was filtered, the obtained solids were

washed with CH_3CN (3 × 1.0 mL), and the combined filtrate was concentrated under reduced pressure. The crude was purified by silica gel flash chromatography, using EtOAc, CH_2Cl_2, 3% (v/v) MeOH/CH_2Cl_2, then 5% (v/v) MeOH/CH_2Cl_2 as the eluent to afford the title compound.

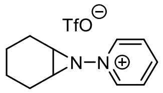

5a

*1-(7-Azabicyclo[4.1.0]heptan-7-yl)pyridin-1-ium trifluoromethanesulfonate (**5a**)*. The title compound was prepared via Procedure A in a 50-mL Schlenk flask from cyclohexene (**2a**, 2.00 mmol), was purified by crystallization from CH_3CN/Et_2O, and was obtained as a yellow solid (494 mg, 1.52 mmol, 76%). Alternatively, trituration of the crude mixture resulted in 92% isolated yields (starting from 0.500 mmol of **2a**) with 94% purity (^{1}H NMR). ^{1}H NMR (400 MHz, CD_3CN) δ 8.80 (d, *J* = 5.6 Hz, 2H), 8.29 (t, *J* = 7.8 Hz, 1H), 7.92 (t, *J* = 7.3 Hz, 2H), 3.39–3.27 (m, 2H), 2.25–2.17 (m, 2H), 2.07–2.03 (m, 2H), 1.45–1.28 (m, 4H). These spectral data are well-matched to those reported in the literature.[26]

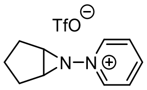

5b

*1-(6-Azabicyclo[3.1.0]hexan-6-yl)pyridin-1-ium trifluoromethanesulfonate (**5b**).* Prepared via Procedure A from cyclopentene (**2b**) and obtained as a yellow powder (57.9 mg, 0.187 mmol, 62%). ^{1}H NMR (400 MHz, CD_3CN) δ 8.83 (d, *J* = 5.6 Hz, 2H), 8.29 (t, *J* = 7.8 Hz, 1H), 7.93 (t, *J* = 7.3 Hz, 2H), 3.75 (m, 2H), 2.34–2.29 (m, 2H), 1.88 (ddt, *J* = 13.6, 8.9, 1.7 Hz, 2H), 1.73 (dt, *J* = 13.0, 8.8 Hz, 1H), 1.35 (dtt, *J* = 13.0, 10.8, 8.2 Hz, 1H). ^{19}F NMR (377 MHz, CD_3CN) δ –79.3. ^{13}C NMR (101 MHz, CD_3CN) δ 143.6, 142.0, 129.3, 121.9 (q, *J* = 320.4 Hz), 54.7, 28.3, 21.0. HRMS-ESI$^+$ (m/z): [M]$^+$ calcd. for $C_{10}H_{13}N_2^+$, 161.1073; found, 161.1071.

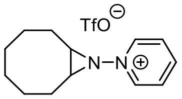

5c

*1-(9-Azabicyclo[6.1.0]nonan-9-yl)pyridin-1-ium trifluoromethanesulfonate (c-**5c**).* Prepared via Procedure A from cyclooctene (**2c**, 0.400 mmol) and obtained as a yellow oil (102 mg, 0.289 mmol, 73%). ^{1}H NMR (400 MHz, CD_3CN) δ 8.82 (dd, *J* = 6.9, 1.3 Hz, 2H), 8.34–8.26 (m, 1H), 7.98–7.86 (m, 2H), 3.25–3.13 (m, 2H), 2.56–2.45 (m, 2H), 1.75–1.64 (m, 2H), 1.57–1.39 (m, 8H). ^{19}F NMR (377 MHz, CD_3CN) δ –79.4. ^{13}C NMR (101 MHz, CD_3CN) δ 143.7, 141.0, 129.2, 122.1 (q, *J* = 320.8 Hz), 51.3, 26.7, 26.5, 25.8. HRMS-ESI$^+$ (m/z): [M]$^+$ calcd. for $C_{13}H_{19}N_2^+$, 203.1543; found, 203.1543.

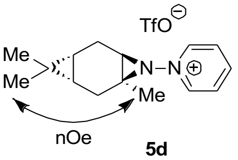

5d

*1-((1S,3S,5R,7R)-3,8,8-Trimethyl-4-azatricyclo[5.1.0.0^{3,5}]octan-4-yl)pyridin-1-ium trifluoromethanesulfonate (**5d**)*. Prepared via Procedure B from 3-carene (**2d**, 0.200 mmol) and obtained as a yellow oil (42.2 mg, 0.112 mmol, 56%). ^{1}H NMR (500 MHz, CD_3CN) δ 8.67 (d, *J* = 5.8 Hz, 2H), 8.32 (t, *J* = 7.8 Hz, 1H), 7.96 (t, *J* = 7.2 Hz, 2H), 3.15 (s, 1H), 2.72–2.58 (m, 1H), 2.50 (dd, *J* = 16.2, 9.3 Hz, 1H), 1.73 (dt, *J* = 16.2, 3.3 Hz, 1H), 1.54–1.47 (m, 1H), 1.06 (s, 3H), 1.04 (s, 3H), 0.79 (s, 3H), 0.59 (dtd, *J* = 33.4, 9.3, 3.2 Hz, 2H). ^{19}F NMR (377 MHz, CD_3CN) δ –79.4. ^{13}C NMR (126 MHz, CD_3CN) δ 143.3, 141.3, 129.3, 122.0 (q, *J* = 320.7 Hz), 50.8, 50.7, 27.8, 24.1, 18.9, 17.8, 17.7, 16.9, 15.3, 15.0. HRMS-ESI$^+$ (m/z): [M]$^+$ calcd. for $C_{15}H_{21}N_2^+$, 229.1699; found, 229.1695.

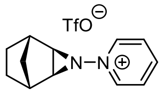

5e

*1-(3-Azatricyclo[3.2.1.0^{2,4}]octan-3-yl)pyridin-1-ium trifluoromethanesulfonate (**5e**).* Prepared via Procedure A from norbornene (**2e**) and obtained as a yellow powder (69.7 mg, 0.207 mmol, 69%). ^{1}H NMR (400 MHz, CD_3CN) δ 8.82 (dd, *J* = 7.0, 1.3 Hz, 2H), 8.27 (t, *J* = 7.8 Hz, 1H), 8.00–7.83 (m, 2H), 3.41 (s, 2H), 2.81 (s, 2H), 1.62–1.52 (m, 2H), 1.49 (dt, *J* = 10.4, 2.2 Hz, 1H), 1.36–1.24 (m, 2H), 1.09–0.95 (m, 1H). ^{19}F NMR (376 MHz, CD_3CN) δ –79.3. ^{13}C NMR (101 MHz, CD_3CN) δ 143.4, 142.0, 129.3, 121.9 (q, *J* = 320.6 Hz), 49.7, 37.3, 29.3, 25.8. HRMS-ESI$^+$ (m/z): [M]$^+$ calcd. for $C_{12}H_{15}N_2^+$, 187.1230; found, 187.1228.

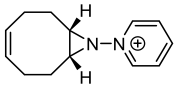

c-**5f**

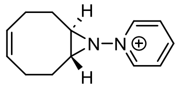

t-**5f**

*(Z)-1-(9-Azabicyclo[6.1.0]non-4-en-9-yl)pyridin-1-ium trifluoromethanesulfonate (**5f**).* Prepared via Procedure A from cyclooctadiene (**2f**) and obtained as a yellow oil (72.5 mg, 0.207 mmol, 69%), which is a mixture of *cis*- and *trans*- isomer (*c*:*t* = 1.4:1).

^{1}H NMR (400 MHz, CD_3CN) *c*-**5f**: δ 8.82 (d, *J* = 5.7 Hz, 2H), 8.36–8.26 (m, 1H), 7.99–7.89 (m, 2H), 5.66–5.56 (m, 2H), 3.32–3.20 (m, 2H), 2.53–2.38 (m, 4H), 2.40–2.28 (m, 2H), 2.16–2.08 (m, 2H). *t*-**5f**: δ 8.67 (d, *J* = 6.3 Hz, 2H), 8.29 (t, *J* = 7.8 Hz, 1H), 7.93 (t, *J* = 7.2 Hz, 2H), 5.82–5.65 (m, 2H), 3.07–2.92 (m, 1H), 2.85–2.72 (m, 1H), 2.53–2.21 (m, 4H), 2.09–2.06 (m, 2H), 1.43–1.26 (m, 1H), 0.90–0.79 (m, 1H). ^{19}F NMR (376 MHz, $CDCl_3$) δ –78.3.[e] ^{13}C NMR (101 MHz, $CDCl_3$), mixture of *c*-**5f** and *t*-**5f**: δ 142.6, 142.2, 140.6, 139.9, 131.8, 130.7, 129.9, 129.1, 128.8,

[e] ^{1}H of *t*-**5f** was taken from sample with enriched purity via preparative HPLC. ^{1}H of *c*-**5f** was obtained by comparison of the mixture against the purified sample. The amount of pure *t*-**5f** sample was not suitable for ^{13}C acquisition (See Figure S2).

120.8 (q, *J* = 320.4 Hz), 54.3, 51.4, 50.8, 50.6, 28.6, 27.6, 25.1, 24.6, 23.6. HRMS-ESI+ (m/z): $[M]^+$ calcd. for $C_{13}H_{17}N_2^+$, 201.1386; found, 201.1386.

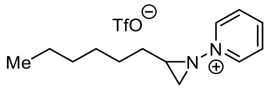

5h

*1-(2-Hexylaziridin-1-yl)pyridin-1-ium trifluoromethanesulfonate (**5h**).* Prepared via Procedure A from 1-octene (**2h**, 0.600 mmol) and obtained as a yellow oil (153 mg, 0.432 mmol, 72%). ^{1}H NMR (400 MHz, CD_3CN) δ 8.76 (d, *J* = 6.8 Hz, 2H), 8.32 (t, *J* = 7.8 Hz, 1H), 7.94 (t, *J* = 7.3 Hz, 2H), 3.17–3.06 (m, 1H), 3.03 (dd, *J* = 8.4, 2.6 Hz, 1H), 2.75 (dd, *J* = 5.7, 2.6 Hz, 1H), 1.82–1.70 (m, 1H), 1.73–1.63 (m, 1H), 1.65–1.52 (m, 2H), 1.46–1.37 (m, 1H), 1.40–1.30 (m, 4H), 0.95–0.87 (m, 3H). ^{19}F NMR (376 MHz, $CDCl_3$) δ –78.4. ^{13}C NMR (101 MHz, $CDCl_3$) δ 142.7, 140.1, 128.8, 47.7, 40.6, 31.7, 31.3, 29.1, 26.6, 22.6, 14.2. HRMS-ESI+ (m/z): $[M]^+$ calcd. for $C_{13}H_{21}N_2^+$, 205.1699; found, 205.1700.

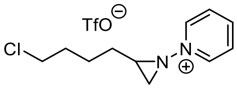

5i

*1-(2-(4-Chlorobutyl)aziridin-1-yl)pyridin-1-ium trifluoromethanesulfonate (**5i**).* Prepared via Procedure A from 6-chloro-1-hexene (**2i**, 0.200 mmol) and obtained as a yellow oil (46.9 mg, 0.130 mmol, 65%). ^{1}H NMR (400 MHz, CD_3CN) δ 8.99–8.87 (m, 2H), 8.44 (t, *J* = 7.8 Hz, 1H), 8.14–7.96 (m, 2H), 3.76 (t, *J* = 6.5 Hz, 2H), 3.27 (dq, *J* = 8.5, 5.8 Hz, 1H), 3.17 (dd, *J* = 8.5, 2.7 Hz, 1H), 2.87 (dd, *J* = 5.6, 2.7 Hz, 1H), 2.03–1.89 (m, 3H), 1.88–1.72 (m, 3H). ^{19}F NMR (377 MHz, CD_3CN) δ –79.3. ^{13}C NMR (101 MHz, CD_3CN) δ 144.0, 141.2, 129.3, 122.0 (q, *J* = 320.7 Hz), 47.5, 45.8, 41.0, 32.8, 30.8, 24.3. HRMS-ESI+ (m/z): $[M]^+$ calcd. for $C_{11}H_{16}ClN_2^+$, 211.0997, 213.0967; found, 211.0994, 213.0963.

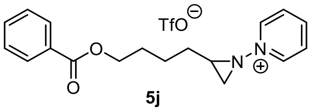

5j

*1-(2-(4-(Benzoyloxy)butyl)aziridin-1-yl)pyridin-1-ium trifluoromethanesulfonate (**5j**).* Prepared via Procedure A from hex-5-en-1-yl benzoate (**2j**, 0.200 mmol) and obtained as a yellow oil (64.3 mg, 0.144 mmol, 72%). ^{1}H NMR (500 MHz, CD_3CN) δ 8.78 (d, *J* = 6.2 Hz, 2H), 8.32 (t, *J* = 7.8 Hz, 1H), 8.03 (d, *J* = 8.4 Hz, 2H), 7.94 (t, *J* = 7.3 Hz, 2H), 7.63 (t, *J* = 7.4 Hz, 1H), 7.50 (t, *J* = 7.8 Hz, 2H), 4.35 (t, *J* = 6.4 Hz, 2H), 3.21–3.12 (m, 1H), 3.05 (dd, *J* = 8.4, 2.7 Hz, 1H), 2.78 (dd, *J* = 5.7, 2.7 Hz, 1H), 1.90–1.82 (m, 3H), 1.81–1.69 (m, 3H). ^{19}F NMR (377 MHz, CD_3CN) δ –79.3. ^{13}C NMR (126 MHz, CD_3CN) δ 167.2, 144.0, 141.2, 134.0, 131.4, 130.2, 129.5, 129.3, 122.0 (q, *J* = 320.5 Hz), 65.4, 47.7, 41.0, 31.2, 29.0, 23.6. HRMS-ESI+ (m/z): $[M]^+$ calcd. for $C_{18}H_{21}N_2O_2^+$, 297.1598; found, 297.1591.

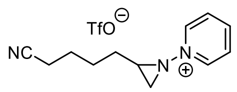

5k

*1-(2-(4-Cyanobutyl)aziridin-1-yl)pyridin-1-ium trifluoromethanesulfonate (**5k**)*. Prepared via Procedure A from hept-6-enenitrile (**2k**, 0.300 mmol) and obtained as a yellow solid (93.8 mg, 0.267 mmol, 89%). ^{1}H NMR (400 MHz, CD_3CN) δ 8.79 (d, *J* = 5.3 Hz, 2H), 8.33 (t, *J* = 7.8 Hz, 1H), 7.96 (t, *J* = 7.1 Hz, 2H), 3.16 (dq, *J* = 10.6, 5.7, 4.7 Hz, 1H), 3.05 (dd, *J* = 8.5, 2.6 Hz, 1H), 2.77 (dd, *J* = 5.8, 2.6 Hz, 1H), 2.45 (t, *J* = 6.4 Hz, 2H), 1.87–1.77 (m, 1H), 1.79–1.62 (m, 5H). ^{19}F NMR (376 MHz, CD_3CN) δ –79.3. ^{13}C NMR (101 MHz, CD_3CN) δ 144.1, 141.2, 129.3, 122.0 (q, *J* = 320.5 Hz), 121.1, 47.3, 41.0, 30.7, 26.0, 25.7, 17.3. HRMS-ESI$^+$ (m/z): [M]$^+$ calcd. for $C_{12}H_{16}N_3^+$, 202.1339; found, 202.1334.

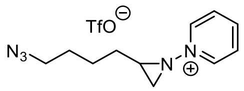

5l

*1-(2-(4-Azidobutyl)aziridin-1-yl)pyridin-1-ium trifluoromethanesulfonate (**5l**)*. Prepared via Procedure B from 6-azidohex-1-ene (**2l**, 0.200 mmol) and obtained as a yellow oil (48.5 mg, 0.132 mmol, 66%). ^{1}H NMR (500 MHz, CD_3CN) δ 8.84–8.73 (m, 2H), 8.32 (t, *J* = 7.8 Hz, 1H), 7.95 (t, *J* = 7.2 Hz, 2H), 3.37 (t, *J* = 6.0 Hz, 2H), 3.13 (dq, *J* = 7.8, 6.1 Hz, 1H), 3.03 (dd, *J* = 8.4, 2.7 Hz, 1H), 2.77 (dd, *J* = 5.7, 2.7 Hz, 1H), 1.80 (p, *J* = 6.7 Hz, 1H), 1.75–1.60 (m, 5H). ^{19}F NMR (376 MHz, CD_3CN) δ –79.3. ^{13}C NMR (126 MHz, CD_3CN) δ 144.1, 141.2, 129.3, 51.8, 47.6, 41.0, 31.1, 29.1, 24.2. HRMS-ESI$^+$ (m/z): [M]$^+$ calcd. for $C_{11}H_{16}N_5^+$, 218.1400; found, 218.1397.

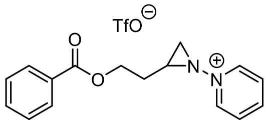

5m

*1-(2-(2-(Benzoyloxy)ethyl)aziridin-1-yl)pyridin-1-ium trifluoromethanesulfonate (**5m**).* Prepared via Procedure A from but-3-en-1-yl benzoate (**2m**, 0.200 mmol) and obtained as a colorless oil (50.8 mg, 0.122 mmol, 61%). ^{1}H NMR (500 MHz, $CDCl_3$) δ 8.94 (d, *J* = 5.6 Hz, 2H), 8.25 (t, *J* = 7.8 Hz, 1H), 8.03 (d, *J* = 6.9 Hz, 2H), 7.92 (t, *J* = 7.3 Hz, 2H), 7.57 (t, *J* = 7.4 Hz, 1H), 7.44 (t, *J* = 7.8 Hz, 2H), 4.69 (ddd, *J* = 11.9, 7.7, 4.4 Hz, 1H), 4.55 (ddd, *J* = 11.4, 6.7, 4.7 Hz, 1H), 3.61 (tt, *J* = 8.1, 5.2 Hz, 1H), 3.48 (dd, *J* = 8.5, 2.7 Hz, 1H), 2.78 (dd, *J* = 5.6, 2.7 Hz, 1H), 2.29 (ddt, *J* = 15.3, 7.9, 4.8 Hz, 1H), 2.13 (dtd, *J* = 14.9, 7.0, 4.4 Hz, 1H). ^{19}F NMR (377 MHz, $CDCl_3$) δ –78.4. ^{13}C NMR (126 MHz, $CDCl_3$) δ 166.8, 142.9, 140.4, 133.6, 129.8, 129.7, 128.8, 128.7, 120.7 (q, *J* = 319.9 Hz), 62.1, 44.7, 40.2, 30.9. HRMS-ESI$^+$ (m/z): [M]$^+$ calcd. for $C_{16}H_{17}N_2O_2^+$, 269.1285; found, 269.12853.

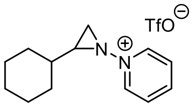

5n

*1-(2-Cyclohexylaziridin-1-yl)pyridin-1-ium trifluoromethanesulfonate (**5n**)*. Prepared via Procedure B from vinylcyclohexene (**2n**, 0.200 mmol) and obtained as a yellow oil (44.3 mg, 0.126 mmol, 63%). ^{1}H NMR (400 MHz, CD_3CN) δ 8.76 (d, *J* = 5.5 Hz, 2H), 8.32 (t, *J* = 7.8 Hz, 1H), 7.95 (t, *J* = 7.3 Hz, 2H), 3.03 (dd, *J* = 8.5, 2.5 Hz, 1H), 2.97 (td, *J* = 8.1, 7.6, 5.4 Hz, 1H), 2.82 (dd, *J* = 5.5, 2.5 Hz, 1H), 2.05–1.98 (m, 1H), 1.86–1.75 (m, 3H), 1.75–1.66 (m, 1H), 1.45–1.11 (m, 6H). ^{19}F NMR (376 MHz, CD_3CN) δ –79.3. ^{13}C NMR (126 MHz, CD_3CN) δ 144.0, 141.2, 129.4, 122.0 (q, *J* = 320.8 Hz), 52.3, 40.0, 39.7, 30.9, 29.6, 26.7, 26.2. HRMS-ESI$^+$ (m/z): [M]$^+$ calcd. for $C_{13}H_{19}N_2^+$, 203.1543; found, 203.1540.

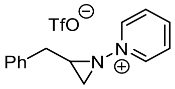

5o

*1-(2-Benzylaziridin-1-yl)pyridin-1-ium trifluoromethanesulfonate (**5o**)*. Prepared via Procedure B from allylbenzene (**2o**, 0.300 mmol) and obtained as a yellow oil (54.6 mg, 0.150 mmol, 50%). ^{1}H NMR (400 MHz, CD_3CN) δ 8.60 (d, *J* = 6.0 Hz, 2H), 8.29 (t, *J* = 7.8 Hz, 1H), 7.97–7.81 (m, 2H), 7.48–7.21 (m, 5H), 3.42–3.33 (m, 1H), 3.14–3.03 (m, 3H), 2.87 (dd, *J* = 5.7, 2.9 Hz, 1H). These spectral data are well-matched to those reported in the literature.[26]

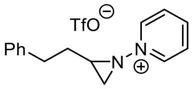

5p

*1-(2-Phenethylaziridin-1-yl)pyridin-1-ium trifluoromethanesulfonate (**5p**)*. Prepared via Procedure A from but-3-en-1-ylbenzene (**2p**, 0.3 mmol) and obtained as a yellow solid (81.6 mg, 0.219 mmol, 73%). Alternatively, trituration of the crude mixture resulted in 90% isolated yield (starting from 0.500 mmol of **2p**) of 80% NMR purity. ^{1}H NMR (500 MHz, $CDCl_3$) δ 8.48 (d, *J* = 6.5 Hz, 2H), 8.14 (t, *J* = 7.8 Hz, 1H), 7.80 (t, *J* = 7.3 Hz, 2H), 7.33–7.03 (m, 2H), 3.31–3.11 (m, 2H), 3.03–2.78 (m, 2H), 2.61–2.53 (m, 1H), 2.16–1.69 (m, 2H). ^{19}F NMR (377 MHz, $CDCl_3$) δ –78.3. ^{13}C NMR (126 MHz, $CDCl_3$) δ 142.7, 140.2, 139.3, 128.9, 128.8, 128.7, 126.7, 120.7 (q, *J* = 319.5 Hz), 46.9, 40.2, 32.7, 32.4. HRMS-ESI$^+$ (m/z): [M]$^+$ calcd. for $C_{15}H_{17}N_2^+$, 225.1386; found, 225.1385.

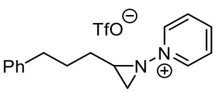

5q

*1-(2-(3-Phenylpropyl)aziridin-1-yl)pyridin-1-ium trifluoromethanesulfonate (**5q**)*. Prepared via Procedure B from pent-4-en-1-ylbenzene (**2q**, 0.200 mmol) and obtained as a yellow oil (35.5 mg, 0.0920 mmol, 46%). ^{1}H NMR (500 MHz, $CDCl_3$) δ 8.55 (d, *J* = 6.5 Hz, 2H), 8.21 (t, *J* = 7.8 Hz, 1H), 7.87 (t, *J* = 7.3 Hz, 2H), 7.40–7.10 (m, 5H), 3.38–3.18 (m, 2H), 3.10–2.85 (m, 2H), 2.67–2.60 (m, 1H), 2.23–1.76 (m, 2H). ^{19}F NMR (377 MHz, CD_3CN) δ –79.3. ^{13}C NMR (126

MHz, CD_3CN) δ 144.0, 143.0, 141.2, 129.4, 129.4, 129.3, 126.9, 122.1 (q, *J* = 320.7 Hz), 47.7, 41.1, 36.0, 31.3, 29.0. HRMS-ESI$^+$ (m/z): $[M]^+$ calcd. for $C_{16}H_{19}N_2^+$, 239.1543; found, 239.1530.

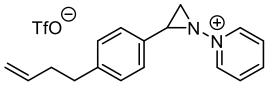

5r

*1-(2-(4-(But-3-en-1-yl)phenyl)aziridin-1-yl)pyridin-1-ium trifluoromethanesulfonate trifluoromethanesulfonate (**5r**)*. Prepared via Procedure B from 1-(but-3-en-1-yl)-4-vinylbenzene (**2r**, 0.300 mmol) and obtained as a yellow oil (78.1 mg, 0.195 mmol, 65%). ^{1}H NMR (500 MHz, CD_3CN) δ 8.91 (dd, *J* = 5.2, 1.9 Hz, 2H), 8.38 (t, *J* = 7.8 Hz, 1H), 8.00 (t, *J* = 7.3 Hz, 2H), 7.37 (d, *J* = 8.2 Hz, 2H), 7.30 (d, *J* = 7.6 Hz, 2H), 5.88 (ddt, *J* = 16.9, 10.2, 6.6 Hz, 1H), 5.04 (dq, *J* = 17.2, 1.8 Hz, 1H), 4.97 (dq, *J* = 10.3, 1.5 Hz, 1H), 4.20 (ddd, *J* = 8.1, 5.7, 2.2 Hz, 1H), 3.49 (ddd, *J* = 8.9, 3.2, 1.3 Hz, 1H), 3.20 (dd, *J* = 5.8, 3.1 Hz, 1H), 2.74 (t, *J* = 7.8 Hz, 2H), 2.46–2.31 (m, 2H). ^{19}F NMR (377 MHz, CD_3CN) δ –79.2. ^{13}C NMR (126 MHz, CD_3CN) δ 144.4, 144.0, 141.2, 139.0, 132.7, 129.8, 129.4, 127.9, 122.0 (d, *J* = 320.6 Hz), 115.5, 48.2, 42.8, 35.9, 35.4. HRMS-ESI$^+$ (m/z): $[M]^+$ calcd. for $C_{17}H_{19}N_2^+$, 251.1543; found, 251.1536.

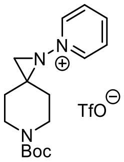

5s

*1-(6-(tert-Butoxycarbonyl)-1,6-diazaspiro[2.5]octan-1-yl)pyridin-1-ium trifluoromethanesulfonate (**5s**)*. Prepared via Procedure B from *tert*-butyl 4-methylenepiperidine-1-carboxylate (**2s**, 0.200 mmol) and obtained as a yellow oil (58.9 mg, 0.134 mmol, 67%). ^{1}H NMR (500 MHz, CD_3CN) δ 8.72 (d, *J* = 5.5 Hz, 2H), 8.36 (t, *J* = 7.8 Hz, 1H), 7.99 (t, *J* = 7.3 Hz, 2H), 4.13 (t, *J* = 15.4 Hz, 2H), 3.09 (dd, *J* = 3.5, 1.1 Hz, 1H), 3.01 (dd, *J* = 3.5, 1.9 Hz, 1H), 2.20 (td, *J* = 12.7, 4.6 Hz, 1H), 2.13 (d, *J* = 16.4 Hz, 1H), 1.80 (tdd, *J* = 11.8, 4.8, 2.0 Hz, 1H), 1.45 (s, 11H), 0.73 (dd, *J* = 12.5, 2.7 Hz, 1H). ^{19}F NMR (376 MHz, CD_3CN) δ –79.3. ^{13}C NMR (126 MHz, CD_3CN) δ 155.2, 144.0, 141.4, 129.4, 122.1 (q, *J* = 320.9 Hz), 80.4, 53.2, 45.1, 30.1, 28.5, 28.5. HRMS-ESI$^+$ (m/z): $[M]^+$ calcd. for $C_{16}H_{24}N_3O_2^+$, 290.1863; found, 290.1855.

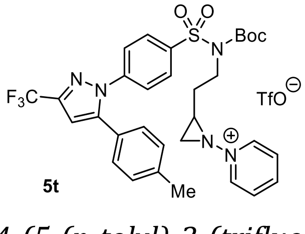

5t

*1-(2-(2-((N-(tert-Butoxycarbonyl)-4-(5-(p-tolyl)-3-(trifluoromethyl)-1H-pyrazol-1-yl)phenyl)sulfonamido)ethyl)aziridin-1-yl)pyridin-1-ium trifluoromethanesulfonate (**5t**)*. Prepared via Procedure A from **2t** (0.300 mmol) and obtained as a yellow solid (113 mg, 0.144 mmol, 48%) containing 15% of the corresponding imine byproduct **5t'**.[f] ^{1}H NMR (500

[f] Resonances at 8.74, 8.53, 8.36, and 8.11 ppm indicate the presence of the imine byproduct **5t'** (Figure S67).

MHz, CD_3CN) δ 8.86 (d, *J* = 5.7 Hz, 2H), 8.34 (t, *J* = 7.8 Hz, 1H), 7.97 (t, *J* = 7.3 Hz, 2H), 7.92 (d, *J* = 8.8 Hz, 2H), 7.53 (d, *J* = 8.7 Hz, 2H), 7.31–7.14 (m, 4H), 6.93 (s, 1H), 4.21–4.05 (m, 2H), 3.28 (dq, *J* = 8.5, 6.3 Hz, 1H), 3.11 (dd, *J* = 8.4, 2.8 Hz, 1H), 2.83 (dd, *J* = 5.6, 2.8 Hz, 1H), 2.34 (s, 3H), 2.26 (dq, *J* = 13.7, 7.2 Hz, 1H), 2.08 (dq, *J* = 14.1, 7.1 Hz, 1H), 1.30 (s, 9H). ^{19}F NMR (376 MHz, CD_3CN) δ –62.8, –79.2. ^{13}C NMR (126 MHz, CD_3CN) δ 178.4, 151.6, 146.7, 144.3, 144.1, 144.0, 141.4, 140.8, 140.4, 130.4, 129.9, 129.6, 129.3, 126.7, 122.4 (q, *J* = 268.0 Hz), 122.1 (q, *J* = 320.7 Hz), 107.2, 86.1, 45.3, 45.2, 40.9, 32.7, 28.0, 21.2. HRMS-ESI$^+$ (m/z): [M]$^+$ calcd. for $C_{31}H_{33}F_3N_5O_4S^+$, 628.2200; found, 628.2190.

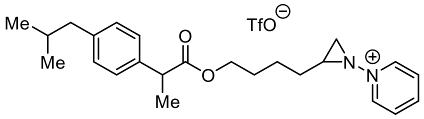

5u

*1-(2-(4-((2-(4-Isobutylphenyl)propanoyl)oxy)butyl)aziridin-1-yl)pyridin-1-ium trifluoromethanesulfonate (**5u**)*. Prepared via Procedure A from **2u** (0.200 mmol) and obtained as a colorless oil (49.0 mg, 0.0920 mmol, 46%). ^{1}H NMR (500 MHz, $CDCl_3$) δ 8.78 (dd, *J* = 5.2, 1.6 Hz, 2H), 8.27 (t, *J* = 7.8 Hz, 1H), 7.97 (t, *J* = 7.3 Hz, 2H), 7.19 (d, *J* = 8.0 Hz, 2H), 7.08 (d, *J* = 6.5 Hz, 2H), 4.11 (td, *J* = 6.5, 1.9 Hz, 2H), 3.70 (q, *J* = 7.1 Hz, 1H), 3.41 (s, 2H), 2.64 (dd, *J* = 4.8, 3.2 Hz, 1H), 2.42 (dd, *J* = 7.2, 4.6 Hz, 2H), 1.83 (dtt, *J* = 13.6, 6.8, 3.6 Hz, 1H), 1.76–1.65 (m, 4H), 1.60–1.52 (m, 2H), 1.49 (d, *J* = 7.1 Hz, 3H), 0.88 (dd, *J* = 6.6, 1.7 Hz, 6H). ^{19}F NMR (377 MHz, $CDCl_3$) δ –78.4. ^{13}C NMR (126 MHz, $CDCl_3$) δ 175.0, 142.7, 140.7, 140.2, 137.9, 129.5, 128.8, 127.3, 120.7 (q, *J* = 320.0 Hz), 64.2, 47.2, 45.3, 45.2, 40.4, 30.7, 30.3, 28.3, 23.0, 22.5, 18.7. HRMS-ESI$^+$ (m/z): [M]$^+$ calcd. for $C_{24}H_{33}N_2O_2^+$, 381.2537; found, 381.2533.

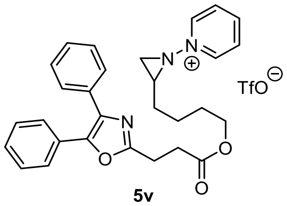

5v

*1-(2-(4-((3-(4,5-Diphenyloxazol-2-yl)propanoyl)oxy)butyl)aziridin-1-yl)pyridin-1-ium trifluoromethanesulfonate (**5v**)*. Prepared via Procedure B from **2v** (0.300 mmol) and obtained as a colorless oil (40.9 mg, 0.0660 mmol, 22%). ^{1}H NMR (500 MHz, CD_3CN) δ 8.76–8.68 (m, 2H), 8.36–8.21 (m, 1H), 7.99–7.85 (m, 2H), 7.62–7.49 (m, 4H), 7.48–7.26 (m, 6H), 4.14 (t, *J* = 6.3 Hz, 2H), 3.13 (t, *J* = 7.0 Hz, 2H), 3.07–2.99 (m, 1H), 2.95 (ddd, *J* = 8.4, 5.3, 2.6 Hz, 1H), 2.88 (t, *J* = 7.1 Hz, 2H), 2.69 (dd, *J* = 5.7, 2.6 Hz, 1H), 1.81–1.67 (m, 3H), 1.66–1.53 (m, 3H). ^{19}F NMR (377 MHz, CD_3CN) δ –79.3. ^{13}C NMR (126 MHz, CD_3CN) δ 173.0, 163.4, 146.2, 144.0, 141.2, 135.7, 133.5, 129.9, 129.8, 129.7, 129.5, 129.3, 129.1, 128.5, 127.4, 122.1 (q, *J* = 321.2 Hz), 65.0, 47.6, 40.9, 31.5, 31.1, 28.9, 24.1, 23.5. HRMS-ESI$^+$ (m/z): [M]$^+$ calcd. for $C_{29}H_{30}N_3O_3^+$, 468.2282; found, 468.2276.

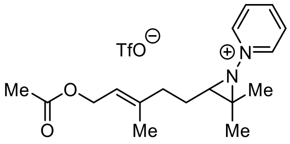

5w

*(E)-1-(3-(5-Acetoxy-3-methylpent-3-en-1-yl)-2,2-dimethylaziridin-1-yl)pyridin-1-ium trifluoromethanesulfonate (**5w**).* Prepared via Procedure A from geranyl acetate (**2w**, 0.200 mmol) and obtained as a yellow oil (50.5 mg, 0.116 mmol, 58%). ^{1}H NMR (400 MHz, CD_3CN) δ 8.64 (d, *J* = 6.6 Hz, 2H), 8.34 (t, *J* = 7.8 Hz, 1H), 7.98 (t, *J* = 7.2 Hz, 2H), 5.57–5.24 (m, 1H), 4.67–4.43 (m, 2H), 3.02 (t, *J* = 6.8 Hz, 1H), 2.36 (t, *J* = 7.5 Hz, 2H), 1.97 (s, 4H), 1.91–1.82 (m, 1H), 1.76 (s, 3H), 1.45 (s, 3H), 1.10 (s, 3H). ^{19}F NMR (376 MHz, CD_3CN) δ –79.3. ^{13}C NMR (101 MHz, CD_3CN) δ 171.6, 143.6, 141.5, 141.1, 129.3, 122.0 (q, *J* = 320.7 Hz), 121.0, 61.7, 56.5, 53.8, 37.1, 25.9, 21.1, 20.6, 19.6, 16.3. HRMS-ESI$^+$ (m/z): [M]$^+$ calcd. for $C_{17}H_{25}N_2O_2^+$, 289.1911; found, 289.1906.

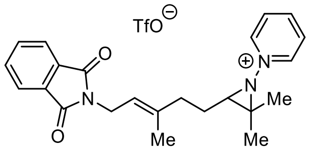

5x

*(E)-1-(3-(5-(1,3-Dioxoisoindolin-2-yl)-3-methylpent-3-en-1-yl)-2,2-dimethylaziridin-1-yl)pyridin-1-ium trifluoromethanesulfonate (**5x**).* Prepare via Procedure A from (*E*)-2-(3,7-dimethylocta-2,6-dien-1-yl)isoindoline-1,3-dione (**2x**, 0.200 mmol) and obtained as a white solid (60.1 mg, 0.114 mmol, 57%). ^{1}H NMR (400 MHz, CD_3CN) δ 8.65–8.55 (m, 2H), 8.28 (t, *J* = 7.6 Hz, 1H), 7.94 (t, *J* = 7.3 Hz, 2H), 7.77 (s, 4H), 5.40 (ddq, *J* = 6.7, 5.4, 1.4 Hz, 1H), 4.23 (dt, *J* = 9.5, 4.8 Hz, 2H), 3.00 (t, *J* = 6.8 Hz, 1H), 2.46–2.27 (m, 2H), 2.19 (s, 1H), 1.84 (m, 4H), 1.43 (s, 3H), 1.04 (s, 3H). ^{19}F NMR (377 MHz, CD_3CN) δ –79.3. ^{13}C NMR (126 MHz, CD_3CN) δ 169.0, 143.5, 141.0, 139.6, 135.1, 133.1, 129.2, 123.8, 122.0 (d, *J* = 320.7 Hz), 120.9, 56.4, 53.6, 37.0, 36.4, 25.6, 20.5, 19.6, 16.3. HRMS-ESI$^+$ (m/z): [M]$^+$ calcd. for $C_{23}H_{26}N_3O_2^+$, 376.2020; found, 376.2016.

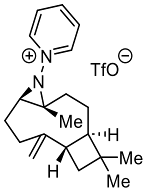

5y

*1-((1R,4R,6R,10S)-4,12,12-Trimethyl-9-methylene-5-azatricyclo[8.2.0.0^{4,6}]dodecan-5-yl)pyridin-1-ium trifluoromethanesulfonate (**5y**).* Prepared via Procedure B from β-caryophyllene (**2y**, 0.200 mmol) and obtained as a yellow solid (74.6 mg, 0.168 mmol, 84%). This was also scaled up using 1.00 mmol of **2y**, affording 369 mg (0.830 mmol) of the product in 83% yield. ^{1}H NMR (400 MHz, CD_3CN) δ 8.66 (d, *J* = 5.8 Hz, 2H), 8.32 (t, *J* = 7.8 Hz, 1H), 7.95 (t, *J* = 7.3 Hz, 2H), 5.04 (d, *J* = 1.7 Hz, 1H), 4.93 (d, *J* = 1.8 Hz, 1H), 3.20 (dd, *J* = 11.3, 4.1 Hz, 1H), 2.75 (q, *J* = 9.6 Hz, 1H), 2.64 (ddt, *J* = 13.4, 7.9, 4.1 Hz, 1H), 2.44 (ddd, *J* = 13.1, 8.8, 4.1 Hz, 1H), 2.24–2.14 (m, 1H), 2.00 (dt, *J* = 12.4, 3.7 Hz, 1H), 1.76 (td, *J* = 9.6, 1.6 Hz, 1H), 1.68–1.59 (m, 4H), 1.56–1.49 (m, 1H), 1.40 (s, 3H), 1.00 (s, 3H), 0.92 (s, 3H), 0.29 (td, *J* = 12.3, 5.4 Hz, 1H). ^{19}F NMR (376 MHz, CD_3CN) δ –79.3. ^{13}C NMR (101 MHz, CD_3CN) δ 152.3, 143.4, 141.2, 129.2, 122.1 (q, *J* = 320.9 Hz), 113.8, 57.4, 55.3, 49.9, 49.0, 40.0, 35.2, 35.0, 30.3, 30.1, 29.7, 26.9, 21.6, 18.3. HRMS-ESI$^+$ (m/z): [M]$^+$ calcd. for $C_{20}H_{29}N_2^+$, 297.2325; found, 297.2320.

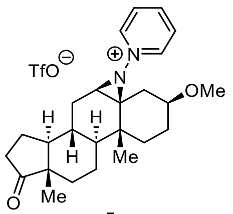

5z

*1-((3S,4aS,5aR,6aR,6bS,9aS,11aS,11bR)-3-Methoxy-9a,11b-dimethyl-9-oxohexadecahydro-5H-cyclopenta[1,2]phenanthro[8a,9-b]azirin-5-yl)pyridin-1-ium trifluoromethanesulfonate (**5z**)*. Prepared via Procedure A from *O*-methyl dehydroepiandrosterone (**2z**, 0.300 mmol) and obtained as a yellow powder (133 mg, 0.246 mmol, 82%). ^{1}H NMR (500 MHz, $CDCl_3$) δ 8.88 (s, 2H), 8.33 (t, *J* = 8.1 Hz, 1H), 8.07 (t, *J* = 7.2 Hz, 2H), 3.95 (s, 1H), 3.52–3.43 (m, 1H), 3.18 (s, 3H), 2.64–2.52 (m, 1H), 2.50–2.36 (m, 1H), 2.15–1.97 (m, 2H), 1.94–1.73 (m, 3H), 1.61 (dt, *J* = 13.0, 9.5 Hz, 3H), 1.55–1.40 (m, 5H), 1.31–1.13 (m, 5H), 0.94–0.75 (m, 5H). ^{19}F NMR (377 MHz, $CDCl_3$) δ –78.3. ^{13}C NMR (126 MHz, $CDCl_3$) δ 220.3, 142.4, 140.5, 128.8, 120.9 (q, *J* = 320.4 Hz), 59.0, 56.1, 52.9, 51.4, 49.1, 47.4, 36.2, 35.8, 34.9, 33.6, 31.5, 30.5, 28.9, 26.0, 21.7, 21.6, 20.4, 13.6. HRMS-ESI$^+$ (m/z): $[M]^+$ calcd. for $C_{25}H_{35}N_2O_2^+$, 395.2693; found, 395.2687.

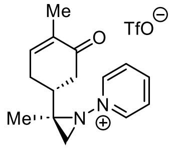

5aa

*1-((R)-2-methyl-2-((R)-4-methyl-5-oxocyclohex-3-en-1-yl)aziridin-1-yl)pyridin-1-iumtrifluoromethanesulfonate (**5aa**)*. Prepare via Procedure A from (*R*)-carvone (**2aa**, 0.300 mmol) and obtained as a yellow oil (47.1 mg, 0.120 mmol, 40%). Alternatively, trituration of the crude mixture resulted in 98% overall isolated yield (starting from 2.00 mmol of **2aa**) as a mixture of aziridine **5aa** and imine **5aa'** (65% and 33% respectively). ^{1}H NMR (500 MHz, CD_3CN) δ 8.67–8.58 (m, 2H), 8.44–8.33 (m, 1H), 8.12–7.91 (m, 2H), 6.94–6.61 (m, 1H), 3.06 (d, *J* = 3.7 Hz, 1H), 2.97 (dd, *J* = 3.8, 2.3 Hz, 1H), 2.61 (dddd, *J* = 27.5, 15.5, 3.6, 1.7 Hz, 1H), 2.55–2.38 (m, 2H), 2.38–2.21 (m, 2H), 1.79–1.71 (m, 3H), 1.10 (s, 3H). ^{19}F NMR (377 MHz, CD_3CN) δ –79.3. ^{13}C NMR (126 MHz, CD_3CN) δ 198.6, 198.5, 146.4, 144.8, 144.7, 143.9, 143.0, 141.4, 141.4, 140.2, 136.1, 136.0, 129.5, 129.5, 122.0 (q, *J* = 321.0 Hz), 54.0, 53.9, 44.6, 42.1, 42.0, 40.5, 40.2, 28.5, 28.2, 15.7, 15.7, 14.9, 14.8. HRMS-ESI$^+$ (m/z): $[M]^+$ calcd. for $C_{15}H_{19}N_2O^+$, 243.1492; found, 243.1490.

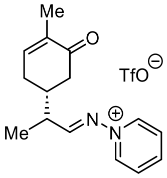

5aa'

*(R)-1-((2-(4-Methyl-5-oxocyclohex-3-en-1-yl)propylidene)amino)pyridin-1-ium trifluoromethanesulfonate (**5aa'**)*. Prepared via Procedure A from (*R*)-carvone (0.300 mmol)

and obtained as a yellow oil (21.6 mg, 0.0540 mmol, 18%). ^{1}H NMR (400 MHz, $CDCl_3$) δ 8.94 (d, *J* = 6.2 Hz, 2H), 8.77 (d, *J* = 5.8 Hz, 1H), 8.52 (t, *J* = 7.6 Hz, 1H), 8.13 (t, *J* = 7.0 Hz, 2H), 6.76 (s, 1H), 3.03–2.78 (m, 1H), 2.57 (dd, *J* = 21.6, 15.9 Hz, 3H), 2.32 (qd, *J* = 11.3, 5.1 Hz, 2H), 1.75 (s, 3H), 1.32 (t, *J* = 6.6 Hz, 3H). ^{19}F NMR (376 MHz, $CDCl_3$) δ –78.5. ^{13}C NMR (126 MHz, $CDCl_3$) δ 198.9, 180.3, 145.2, 144.8, 139.4, 135.8, 129.0, 120.6 (q, *J* = 320.0 Hz), 42.0, 41.3, 37.9, 30.1, 15.7, 13.5. HRMS-ESI$^+$ (m/z): $[M]^+$ calcd. for $C_{15}H_{19}N_2O^+$, 243.1492; found, 243.1490.

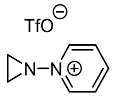

5ab

*1-(Aziridin-1-yl)pyridin-1-ium trifluoromethanesulfonate (**5ab**)*. Prepared via Procedure C from ethylene and **3** (0.600 mmol), and obtained as a yellow oil (78.0 mg, 0.288 mmol, 48%). ^{1}H NMR (400 MHz, CD_3CN) δ 9.01 (d, *J* = 6.0 Hz, 2H), 8.35 (t, *J* = 7.8 Hz, 1H), 8.09–7.75 (m, 2H), 3.03–2.96 (m, 2H), 2.88–2.80 (m, 2H). ^{19}F NMR (377 MHz, CD_3CN) δ –79.3. ^{13}C NMR (101 MHz, CD_3CN) δ 144.1, 141.6, 129.3, 35.0. HRMS-ESI^{+} (m/z): $[M]^{+}$ calcd. for $C_7H_9N_2^{+}$, 121.0760; found, 121.0762.

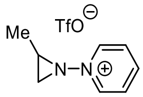

5ac

*1-(2-Methylaziridin-1-yl)pyridin-1-ium trifluoromethanesulfonate (**5ac**)*. Prepared via Procedure C from propylene and **3** (0.200 mmol), and obtained as a yellow oil (21.7 mg, 0.0760 mmol, 38%) containing 13% of the imine byproduct **5ac'**. ^{1}H NMR (400 MHz, CD_3OD) δ 9.13 (d, *J* = 5.7 Hz, 2H), 8.40 (t, *J* = 7.8 Hz, 1H), 8.10–7.87 (m, 2H), 3.30–3.22 (m, 1H), 3.15 (dd, *J* = 8.4, 2.6 Hz, 1H), 2.74 (dd, *J* = 5.8, 2.5 Hz, 1H), 1.53 (d, *J* = 5.7 Hz, 3H). ^{19}F NMR (377 MHz, CD_3OD) δ –80.1. ^{13}C NMR (101 MHz, CD_3OD) δ 144.1, 141.7, 129.5, 121.8 (q, *J* = 318.5 Hz), 44.0, 41.9, 16.9. HRMS-ESI^{+} (m/z): $[M]^{+}$ calcd. for $C_8H_{11}N_2^{+}$, 135.0917; found, 135.0915.

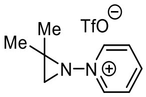

5ad

*1-(2,2-Dimethylaziridin-1-yl)pyridin-1-ium trifluoromethanesulfonate (**5ad**)*. Prepared via Procedure C from isobutylene and **3** (0.200 mmol), and obtained as a yellow oil (20.7 mg, 0.0700 mmol, 35%) containing 33% of imine byproduct **5ad'**. ^{1}H NMR (400 MHz, CD_3CN) δ 8.71 (d, *J* = 5.9 Hz, 2H), 8.35 (t, *J* = 7.8 Hz, 1H), 7.99 (t, *J* = 7.3 Hz, 2H), 2.97 (d, *J* = 3.5 Hz, 1H), 2.91 (d, *J* = 3.7 Hz, 1H), 1.50 (s, 3H), 1.13 (s, 3H). ^{19}F NMR (376 MHz, CD_3CN) δ –79.3. ^{13}C NMR (101 MHz, CD_3CN) δ 182.5, 146.1, 143.5, 141.4, 140.1, 129.5, 129.3, 121.9 (q, *J* = 320.5 Hz), 49.6, 46.5, 33.4, 24.1, 19.1, 18.4. (Extra signals are due to the imine byproduct.) HRMS-ESI^{+} (m/z): $[M]^{+}$ calcd. for $C_9H_{13}N_2^{+}$, 149.1073; found, 149.1071.

B.5 Cross-Coupling of Pyridinium Aziridines

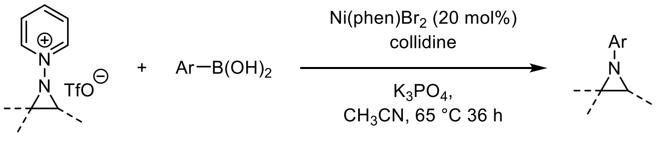

5 **10** **11**

A 20-mL scintillation vial was charged with Ni(phen)Br_2 (20 mol%), potassium phosphate (2.8 equiv.), aryl boronic acid (**10**, 2.4 equiv.), and pyridinium aziridine (**5**, 1.0 equiv.). In an N_2 filled dry box, a solution of 2,4,6-collidine in acetonitrile (0.10 M, 1.0 equiv.) was added to the reaction vial. With stirring, the reaction mixture was heated to 65 °C for 36 h. After cooling to 23 °C, the reaction mixture was transferred to a centrifuge tube and centrifuged at 4000 rpm for 4 min. The supernatant was decanted. The residue was washed with CH_2Cl_2 and the combined supernatants were concentrated under reduced pressure and the crude mixture was purified as indicated below to afford the indicated compounds.

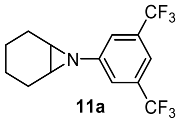

7-(3,5-Bis(trifluoromethyl)phenyl)-7-azabicyclo[4.1.0]heptane (***11a***). Prepared from **5a** (0.200 mmol), purified on deactivated silica gel column[g] (1% to 10% Et_2O in hexanes), and obtained as a white solid (31.1 mg, 0.100 mmol, 50%). ^{1}H NMR (499 MHz, $CDCl_3$) δ 7.40 (s, 1H), 7.33 (s, 2H), 2.46–2.38 (m, 2H), 2.07 (dt, J = 13.3, 6.5 Hz, 2H), 1.99–1.89 (m, 2H), 1.55–1.44 (m, 2H), 1.38–1.28 (m, 2H). ^{19}F NMR (470 MHz, $CDCl_3$) δ –63.0. ^{13}C NMR (126 MHz, $CDCl_3$) δ 157.2, 132.3 (q, J = 33.0 Hz), 123.5 (q, J = 272.6 Hz), 120.6, 115.3, 39.7, 24.5, 20.2. HRMS-ESI$^+$ (m/z): $[M+1]^+$ calcd. for $C_{14}H_{14}F_6N^+$, 310.1025; found, 310.1020.

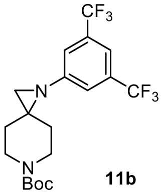

11b

tert-Butyl 1-(3,5-bis(trifluoromethyl)phenyl)-1,6-diazaspiro[2.5]octane-6-carboxylate (***11b***). Prepared from **5s** (0.100 mmol), purified on deactivated silica gel column (10% EtOAc in hexanes), and obtained as a white solid (14.7 mg, 0.0350 mmol, 35%). ^{1}H NMR (500 MHz, $CDCl_3$) δ 7.45 (s, 1H), 7.28 (s, 2H), 4.12 (s, 2H), 2.99 (t, J = 12.2 Hz, 2H), 1.91 (td, J = 12.2, 4.2 Hz, 2H), 1.48 (s, 9H), 1.12 (d, J = 13.2 Hz, 2H). ^{19}F NMR (376 MHz, $CDCl_3$) δ –63.0. ^{13}C NMR (126 MHz, $CDCl_3$) δ 154.8, 151.9, 132.4 (q, J = 33.1 Hz), 123.4 (q, J = 272.8 Hz), 120.8, 115.7, 80.2, 44.9, 39.2, 33.3, 28.6. HRMS-ESI$^+$ (m/z): $[M+1]^+$ calcd. for $C_{19}H_{23}F_6N_2O_2^+$, 425.1658; found, 425.1647.

---

[g] Silica gel (20 mL) was wet-packed with hexanes. 1% Et_3N in hexanes (20 mL) was passed through the column, followed by 100% hexanes (20 mL). The crude mixture dried in neutral alumina was dry-loaded on the column.

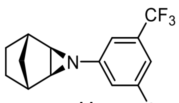

11c

*exo-3-(3,5-Bis(trifluoromethyl)phenyl)-3-azatricyclo[3.2.1.0^{2,4}]octane (**11c**)*. Prepared from **5e** (0.150 mmol), purified on deactivated silica gel column (1% to 10% Et_2O in hexanes), and obtained as a white solid (31.4 mg, 0.0975 mmol, 65%). ^{1}H NMR (400 MHz, $CDCl_3$) δ 7.35 (s, 1H), 7.32 (s, 2H), 2.60 (s, 2H), 2.40 (s, 2H), 1.61–1.44 (m, 3H), 1.28–1.17 (m, 2H), 0.90 (d, *J* = 10.2 Hz, 1H). ^{19}F NMR (376 MHz, $CDCl_3$) δ –63.1. ^{13}C NMR (126 MHz, $CDCl_3$) δ 154.7, 132.3 (q, *J* = 33.1 Hz), 123.5 (q, *J* = 272.7 Hz), 121.0, 42.1, 36.4, 29.1, 26.2. HRMS-ESI$^+$ (m/z): $[M+1]^+$ calcd. for $C_{15}H_{14}F_6N^+$, 322.1025; found, 322.1020. The spectroscopic data didn't match those reported in literature, but XRD analysis unambiguously characterized the structure of **11c** (see Figure S24).

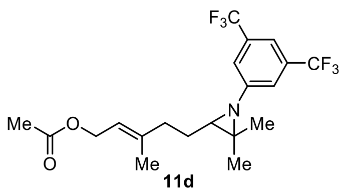

11d

*(E)-5-(1-(3,5-Bis(trifluoromethyl)phenyl)-3,3-dimethylaziridin-2-yl)-3-methylpent-2-en-1-yl acetate (**11d**)*. Prepared from **5w** (0.200 mmol), purified on neutral alumina column (0% to 5% EtOAc in hexanes), and obtained as a colorless oil (35.9 mg, 0.0840 mmol, 42%). ^{1}H NMR (400 MHz, $CDCl_3$) δ 7.41 (s, 1H), 7.18 (s, 2H), 5.47 (t, *J* = 6.7 Hz, 1H), 4.61 (d, *J* = 7.0 Hz, 2H), 2.31 (td, *J* = 7.6, 3.4 Hz, 2H), 2.03 (s, 3H), 2.00 (dd, *J* = 7.4, 5.7 Hz, 1H), 1.86–1.70 (m, 5H), 1.37 (s, 3H), 1.00 (s, 3H). ^{19}F NMR (377 MHz, $CDCl_3$) δ –63.0. ^{13}C NMR (101 MHz, $CDCl_3$) δ 171.2, 153.0, 141.2, 132.2 (q, *J* = 33.1 Hz), 123.5 (q, *J* = 272.7 Hz), 120.4, 119.5, 115.1, 61.3, 50.6, 44.1, 37.7, 27.1, 22.0, 21.1,[h] 16.6. HRMS-ESI$^+$ (m/z): $[M+1]^+$ calcd. for $C_{20}H_{24}F_6NO_2^+$, 424.1706; found, 424.1697.

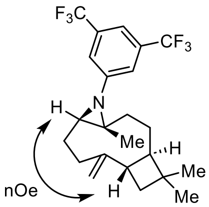

11e

*(1R,4R,6R,10S)-5-(3,5-Bis(trifluoromethyl)phenyl)-4,12,12-trimethyl-9-methylene-5-azatricyclo[8.2.0.0^{4,6}]dodecane (**11e**)*. Prepared from **5y** (0.200 mmol), purified on neutral alumina column (100% hexanes), and obtained as a colorless oil (57.8 mg, 0.134 mmol, 67%). ^{1}H NMR (500 MHz, $CDCl_3$) δ 7.39 (s, 1H), 7.17 (s, 2H), 5.02 (d, *J* = 1.7 Hz, 1H), 4.93 (d, *J* = 1.6 Hz, 1H), 2.66 (dd, *J* = 19.4, 8.6 Hz, 1H), 2.44 (ddt, *J* = 11.5, 6.7, 3.8 Hz, 2H), 2.24–2.12 (m, 2H), 1.92 (dt, *J* = 13.1, 3.6 Hz, 1H), 1.70–1.58 (m, 5H), 1.55–1.46 (m, 1H), 1.32 (s, 3H), 1.00 (s, 3H), 0.93 (s, 3H), 0.36 (td, *J* = 12.8, 4.8 Hz, 1H). ^{19}F NMR (376 MHz, $CDCl_3$) δ –63.0. ^{13}C NMR (126 MHz, $CDCl_3$) δ 132.2 (q, *J* = 32.9 Hz), 123.5 (q, *J* = 272.6 Hz), 120.3, 114.9, 112.7, 51.3, 51.1,

[h] Two resonances for methyl carbons overlap at 21.1, which is confirmed by HSQC analysis.

49.0, 45.7, 39.5, 37.0, 34.3, 31.3, 31.0, 30.0, 27.2, 21.7, 19.2. HRMS-ESI+ (m/z): [M+1]+ calcd. for $C_{23}H_{28}F_6N^+$, 432.2120; found, 432.2105.

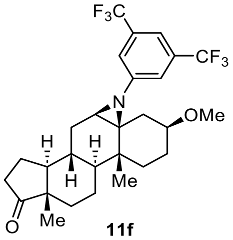

11f

*(3S,4aS,5aR,6aR,6bS,9aS,11aS,11bR)-5-(3,5-Bis(trifluoromethyl)phenyl)-3-methoxy-9a,11b-dimethylhexadecahydro-9H-cyclopenta[1,2]phenanthro[8a,9-b]azirin-9-one (**11f**)*. Prepared from **5z** (0.150 mmol), purified on neutral alumina column (10% EtOAc in hexanes), and obtained as a white solid (26.5 mg, 0.0495 mmol, 33%). ^{1}H NMR (400 MHz, $CDCl_3$) δ 7.41 (s, 1H), 7.21 (s, 2H), 3.39 (dq, *J* = 10.6, 5.8 Hz, 1H), 3.23 (s, 3H), 2.54–2.41 (m, 2H), 2.34 (dt, *J* = 13.6, 3.1 Hz, 1H), 2.16–1.93 (m, 2H), 1.92–1.71 (m, 5H), 1.65–1.33 (m, 7H), 1.27–1.15 (m, 5H), 0.84 (s, 4H), 0.65 (dd, *J* = 13.2, 5.7 Hz, 1H). ^{19}F NMR (377 MHz, $CDCl_3$) δ –63.0. ^{13}C NMR (101 MHz, $CDCl_3$) δ 220.8, 151.9, 132.3 (q, *J* = 33.0 Hz), 123.5 (q, *J* = 272.7 Hz), 120.1, 115.1, 77.4, 55.9, 51.7, 50.2, 49.6, 47.7, 47.3, 36.0, 35.9, 35.0, 31.8, 31.0, 30.5, 26.2, 21.9, 21.8, 21.0, 13.7. (The signal at 77.4 ppm overlapped with $CDCl_3$ signal and was confirmed by HSQC.) HRMS-ESI+ (m/z): [M+1]+ calcd. for $C_{28}H_{34}F_6NO_2^+$, 530.2488; found, 530.2483.

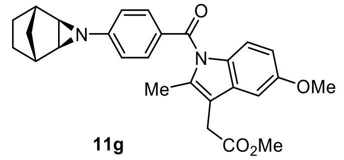

11g

*Methyl exo-2-(1-(4-(3-azatricyclo[3.2.1.0^{2,4}]octan-3-yl)benzoyl)-5-methoxy-2-methyl-1H-indol-3-yl)acetate (**11g**)*. Prepared from **5e** (0.150 mmol), purified on neutral alumina column (10% EtOAc in hexanes), and obtained as a yellow solid (29.8 mg, 0.0675 mmol, 45%). ^{1}H NMR (400 MHz, $CDCl_3$) δ 7.59 (d, *J* = 8.6 Hz, 2H), 7.01–6.93 (m, 3H), 6.89 (d, *J* = 9.0 Hz, 1H), 6.63 (dd, *J* = 9.0, 2.6 Hz, 1H), 3.84 (s, 3H), 3.70 (s, 3H), 3.67 (s, 2H), 2.58 (s, 2H), 2.42–2.38 (m, 5H), 1.64–1.43 (m, 3H), 1.24 (dd, *J* = 7.6, 2.4 Hz, 2H), 0.90 (d, *J* = 9.9 Hz, 1H). ^{13}C NMR (101 MHz, $CDCl_3$) δ 171.7, 169.1, 158.3, 155.8, 136.3, 131.8, 131.3, 130.4, 127.9, 120.8, 115.0, 111.5, 101.1, 55.9, 52.2, 41.8, 36.5, 30.4, 29.2, 26.3, 13.2. HRMS-ESI+ (m/z): [M+1]+ calcd. for $C_{27}H_{29}N_2O_4^+$, 445.2122; found, 445.2118.

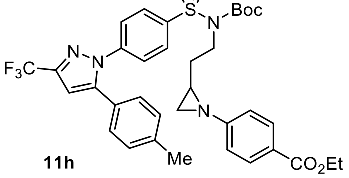

11h

*Ethyl 4-(2-(2-((N-(tert-butoxycarbonyl)-4-(5-(p-tolyl)-3-(trifluoromethyl)-1H-pyrazol-1-yl)phenyl)sulfonamido)ethyl)aziridin-1-yl)benzoate (**11h**)*. Prepared from **5t** (0.107 mmol), purified on deactivated silica gel column (17% to 25% EtOAc in hexanes), and obtained as a white solid (7.3 mg, 0.0107 mmol, 10%). ^{1}H NMR (400 MHz, $CDCl_3$) δ 7.92 (d, *J* = 8.6 Hz, 2H), 7.89 (d, *J* = 8.8 Hz, 2H), 7.48 (d, *J* = 8.8 Hz, 2H), 7.17 (d, *J* = 7.9 Hz, 2H), 7.10 (d, *J* = 8.2 Hz, 2H),

6.99 (d, *J* = 8.6 Hz, 2H), 6.74 (s, 1H), 4.34 (q, *J* = 7.1 Hz, 2H), 4.07 (t, *J* = 7.4 Hz, 2H), 2.38 (s, 3H), 2.28–2.12 (m, 4H), 1.99 (dq, *J* = 13.9, 7.5 Hz, 1H), 1.42–1.33 (m, 12H). ^{19}F NMR (377 MHz, $CDCl_3$) δ –62.5. ^{13}C NMR (126 MHz, $CDCl_3$) δ 166.5, 158.9, 150.7, 145.5, 144.4 (q, *J* = 38.8 Hz), 143.1, 140.0, 139.6, 131.0, 130.0, 129.0, 128.9, 126.0, 125.1, 124.7, 121.2 (q, *J* = 269.0 Hz), 120.5, 106.7, 85.1, 60.8, 45.3, 37.5, 33.8, 33.7, 28.1, 21.5, 14.5. HRMS-ESI$^+$ (m/z): [M+1]$^+$ calcd. for $C_{35}H_{38}F_3N_4O_6S^+$, 699.2459; found, 699.2442.

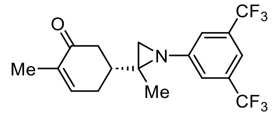

11i

*(R)-5-(1-(3,5-Bis(trifluoromethyl)phenyl)-2-methylaziridin-2-yl)-2-methylcyclohex-2-en-1-one (**11i**)*. Prepared from **5aa** (0.150 mmol), purified on neutral alumina column (0% to 10% EtOAc in hexanes), and obtained as a colorless oil (8.0 mg, 0.021 mmol, 14%). ^{1}H NMR (400 MHz, $CDCl_3$) δ 7.46 (s, 1H), 7.20 (s, 2H), 6.79 (s, 1H), 2.74–2.60 (m, 1H), 2.59–2.26 (m, 4H), 2.16 (m, 1H), 2.00–1.92 (m, 1H), 1.80 (s, 3H), 1.02 (s, 3H). ^{19}F NMR (376 MHz, $CDCl_3$) δ –63.0. ^{13}C NMR (126 MHz, $CDCl_3$) δ 199.0, 198.9, 152.0, 151.9, 144.1, 143.8, 136.1, 135.9, 132.5 (q, *J* = 36.5 Hz), 120.6, 120.1 (q, *J* = 272.8 Hz), 115.6, 45.2, 45.0, 42.5, 42.2, 40.9, 40.6, 39.0, 38.5, 28.7, 28.4, 16.4, 16.0, 15.9.[i] HRMS-ESI$^+$ (m/z): [M+1]$^+$ calcd. for $C_{18}H_{18}F_6NO^+$, 378.1287; found, 378.1279.

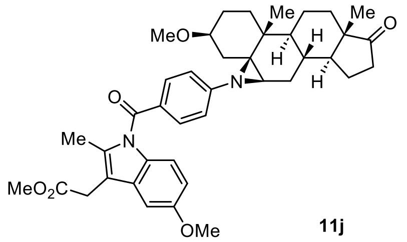

11j

*Methyl 2-(5-methoxy-1-(4-((3S,4aS,5aR,6aR,6bS,9aS,11aS,11bR)-3-methoxy-9a,11b-dimethyl-9-oxohexadecahydro-5H-cyclopenta[1,2]phenanthro[8a,9-b]azirin-5-yl)benzoyl)-2-methyl-1H-indol-3-yl)acetate (**11j**)*. Prepared from **5z** (0.100 mmol), purified on deactivated silica gel column (10% to 25% EtOAc in hexanes), and obtained as a yellow solid (14.9 mg, 0.0230 mmol, 23%). ^{1}H NMR (400 MHz, $CDCl_3$) δ 7.63 (d, *J* = 8.5 Hz, 2H), 7.01–6.86 (m, 4H), 6.65 (dd, *J* = 9.0, 2.6 Hz, 1H), 3.84 (s, 3H), 3.70 (s, 3H), 3.68 (s, 2H), 3.39 (dt, *J* = 10.5, 5.3 Hz, 1H), 3.22 (s, 3H), 2.53–2.41 (m, 2H), 2.40 (s, 3H), 2.37–2.29 (m, 1H), 2.16–1.94 (m, 2H), 1.83 (dt, *J* = 13.6, 5.7 Hz, 5H), 1.66–1.31 (m, 6H), 1.29–1.20 (m, 2H), 1.18 (s, 3H), 0.87 (s, 3H), 0.82 (dd, *J* = 11.5, 4.0 Hz, 1H), 0.72 (dd, *J* = 13.3, 5.7 Hz, 1H). ^{13}C NMR (126 MHz, $CDCl_3$) δ 220.8, 171.7, 169.2, 155.9, 155.6, 136.3, 131.7, 131.4, 130.4, 128.3, 120.2, 115.0, 111.6, 111.5, 101.2, 77.4, 55.9, 52.2, 51.9, 49.9, 49.3, 47.7, 47.1, 36.1, 36.0, 35.8, 34.8, 31.8, 31.3, 30.5, 30.4, 26.6, 21.9, 21.8, 21.1, 13.9, 13.2. HRMS-ESI$^+$ (m/z): [M+1]$^+$ calcd. for $C_{40}H_{49}N_2O_6^+$, 653.3585; found, 653.3576.

[i] Excess ^{13}C NMR resonances are attributed to an inseparable mixture of diastereomers.

B.6 Deprotection of Pyridinium Aziridines

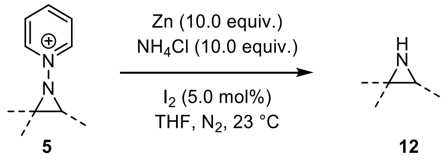

A 20-mL scintillation vial charged with zinc dust (130 mg, 2.00 mmol 10.0 equiv.) and ammonium chloride (107 mg, 2.00 mmol, 10.0 equiv.) was heated under vacuum at 65 °C for 30 min, which was then taken to an N_2 filled dry box. Tetrahydrofuran (0.5 mL) was added, followed by a solution of I_2 in tetrahydrofuran (0.10 M, 0.10 mL, 5.0 mol%). The mixture was vigorously stirred until the color of the liquid phase disappeared. Pyridinium aziridine (**5**, 0.200 mmol, 1.00 equiv.) was dissolved in tetrahydrofuran (0.5 mL), and added to the suspension, which was then stirred for 12 h at 23 °C. The mixture was filtered through celite, and the filtrate was concentrated under reduced pressure. The crude mixture was purified as indicated below to afford the indicated compounds.

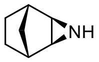

12a

*3-Azatricyclo[3.2.1.0^{2,4}]octane (**12a**)*. Prepared from **5e**. NMR analysis of the reaction mixture at 4 h indicated quantitative conversion. Without further purification, **12a** was transformed to **13** and **14** for isolation.

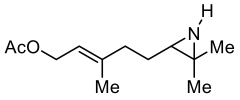

12b

*(E)-5-(3,3-Dimethylaziridin-2-yl)-3-methylpent-2-en-1-yl acetate (**12b**)*. Prepared from **5w**, purified on silica gel flash chromatography (hexanes/acetone = 4:1 to 1:1, with 0.1% Et_3N), and obtained as a yellow oil (23.2 mg, 0.110 mmol, 55%). ^1H NMR (400 MHz, $CDCl_3$) δ 5.37 (td, J = 7.1, 1.3 Hz, 1H), 4.58 (d, J = 7.1 Hz, 2H), 2.26–2.15 (m, 1H), 2.19–2.08 (m, 1H), 2.04 (s, 3H), 1.93 (t, J = 6.7 Hz, 1H), 1.72 (s, 3H), 1.68–1.54 (m, 2H), 1.31 (s, 3H), 1.22 (s, 3H). HRMS-ESI$^+$ (m/z): $[M+1]^+$ calcd. for $C_{12}H_{22}NO_2^+$, 212.1645; found, 212.1637. These spectral data are well-matched to those reported in the literature.[27]

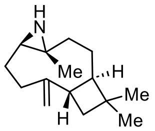

12c

*(1R,4R,6R,10S)-4,12,12-Trimethyl-9-methylene-5-azatricyclo[8.2.0.0^{4,6}]dodecane (**12c**)*. Prepared from **5y**, purified on silica gel flash chromatography (hexanes/acetone = 4:1 to 1:1, with 0.1% Et_3N), and obtained as a colorless oil (18.7 mg, 0.0860 mmol, 43%). ^1H NMR (400

MHz, $CDCl_3$) δ 4.95 (d, *J* = 1.7 Hz, 1H), 4.82 (d, *J* = 1.7 Hz, 1H), 2.59 (q, *J* = 9.3 Hz, 1H), 2.40–2.28 (m, 1H), 2.23–2.09 (m, 2H), 2.00 (ddd, *J* = 13.1, 8.7, 4.6 Hz, 1H), 1.90 (dd, *J* = 11.0, 4.2 Hz, 1H), 1.73 (t, *J* = 9.6 Hz, 1H), 1.69–1.60 (m, 2H), 1.56–1.52 (m, 1H), 1.51–1.45 (m, 1H), 1.21–1.15 (m, 1H), 1.08 (s, 3H), 0.98 (s, 3H), 0.95 (s, 3H), 0.64 (td, *J* = 12.9, 4.8 Hz, 1H). HRMS-ESI$^+$ (m/z): $[M+1]^+$ calcd. for $C_{15}H_{26}N^+$, 220.2060; found, 220.2059. These spectral data are well-matched to those reported in the literature.[27]

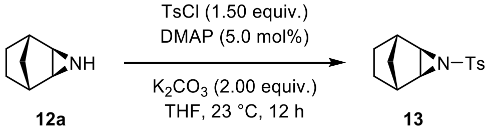

12a **13**

*exo-N-Tosyl-3-azatricyclo[3.2.1.0^{2,4}]octane (**13**).* The above mentioned reaction mixture of **12a** was filtered through celite to a 20-mL scintillation vial containing *p*-methylbenzenesulfonyl chloride (57.2 mg, 0.300 mmol, 1.50 equiv.), *N*,*N*-dimethylpyridin-4-amine (1.2 mg, 0.0010 mmol, 5.0 mol%), and potassium carbonate (55.3 mg, 0.400 mmol, 2.00 equiv.). The mixture was stirred at 23 °C for 12 h, and filtered through celite. The filtrate was concentrated under reduced pressure, and the crude mixture was purified by silica gel flash chromatography (10% EtOAc in hexanes). **13** was obtained as a white solid (35.0 mg, 0.134 mmol, 67%). ^{1}H NMR (400 MHz, $CDCl_3$) δ 7.79 (d, *J* = 6.6 Hz, 2H), 7.31 (d, *J* = 7.9 Hz, 2H), 2.90 (s, 2H), 2.43 (s, 5H), 1.56–1.39 (m, 3H), 1.29–1.17 (m, 2H), 0.74 (d, *J* = 10.1 Hz, 1H). ^{13}C NMR (101 MHz, $CDCl_3$) δ 144.2, 136.0, 129.7, 127.7, 42.0, 35.9, 28.3, 25.7, 21.7. These spectral data are well-matched to those reported in the literature.[28]

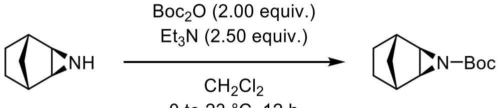

13a **14**

*exo-tert-Butyl 3-azatricyclo[3.2.1.0^{2,4}]octane-N-carboxylate (**14**).* The above mentioned reaction mixture of **13a** was filtered to a 50-mL round bottom flask, and the filtrate was concentrated under reduce pressure. To the resulting yellow oil was added dichloromethane (1.0 mL), triethylamine (50.6 mg, 0.500 mmol, 2.50 equiv.), and the mixture was cooled to 0 °C. A solution of di-*tert*-butyl dicarbonate (87.3 mg, 0.400 mmol, 2.00 equiv.) in dichloromethane (1.0 mL) was added. The reaction was stirred for 12 h and allowed to warm to 23 °C. The reaction mixture was concentrated under reduced pressure, and the crude mixture was filtered through a silica gel plug (17% EtOAc in hexanes). The filtrate was concentrated and dried under vacuum to afford **14** as a pale yellow solid (36.8 mg, 0.176 mmol, 88%). ^{1}H NMR (400 MHz, $CDCl_3$) δ 2.50 (s, 2H), 2.46 (s, 2H), 1.47–1.39 (m, 11H), 1.39–1.29 (m, 1H), 1.23–1.12 (m, 2H), 0.75 (d, *J* = 10.0 Hz, 1H). ^{13}C NMR (101 MHz, $CDCl_3$) δ 161.5, 80.7, 39.1, 36.0, 28.1, 28.1, 26.0. HRMS-ESI$^+$ (m/z): $[M+Na]^+$ calcd. for $C_{12}H_{19}NO_2Na^+$, 232.1308; found, 232.1304. These spectral data are well-matched to those reported in the literature.[29]

C. Reaction Optimization Studies

C.1 Optimization of Aliphatic Olefin Aziridination

Table S1. Evaluation of metal-catalyzed intermolecular aziridination. Yields were determined by ^1H NMR analysis of the crude mixture with 1,3,5-trimethoxybenzene as the internal standard.

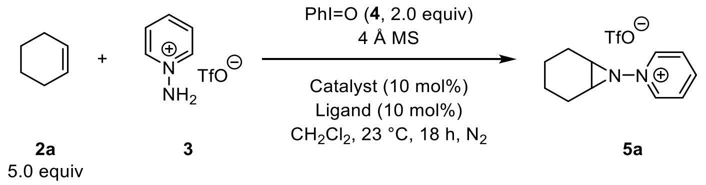

Entry	Solvent	Catalyst	Ligand	NMR yield
1	CH_2Cl_2	AgOTf	TPA	29%
2	CH_2Cl_2	AgOTf	Tp*Br	26%
3	CH_2Cl_2	AgOTf	Tp	13%
4	CH_3CN	AgOTf	^{t}Bu$_3$terpy	62%
5	CH_3CN	CuI	phen	12%
6	CH_3CN	Cu(^{t}Bu$_2$bpy)(OTf)$_2$		33%
7	CH_3CN	Rh$_2$(tfacam)$_4$ (5 mol%)		50%
8	CH_3CN	Mn(TPP)Cl		5%
9	HFIP	AgOTf	^{t}Bu$_3$terpy	92%
10	HFIP	CuI	phen	88%
11	HFIP	Cu(^{t}Bu$_2$bpy)(OTf)$_2$		64%
12	HFIP	Rh$_2$(tfacam)$_4$ (5 mol%)		91%
13	HFIP	Mn(TPP)Cl		63%

Table S2. Evaluation of the impact of reaction stoichiometry on metal-free aziridination. Yields were determined by ^{1}H NMR analysis of the crude mixture with 1,3,5-trimethoxybenzene as the internal standard.

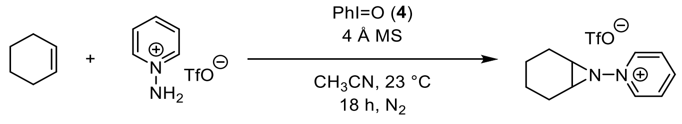

Entry	Cyclohexene	py–NH$_2$	PhI=O	NMR yield
1	1.0 equiv.	1.0 equiv.	1.0 equiv.	33%
2	1.0 equiv.	1.0 equiv.	2.0 equiv.	46%
3	1.0 equiv.	1.2 equiv.	2.4 equiv.	34%
4	1.0 equiv.	1.6 equiv.	3.0 equiv.	20%
5	5.0 equiv.	1.0 equiv.	2.0 equiv.	70%
6	5.0 equiv.	1.0 equiv.	3.0 equiv.	67%
**7**	**3.0 equiv.**	**1.0 equiv.**	**2.0 equiv.**	**68%**

Table S3. Evaluation of the impact of CH_3CN/HFIP ratio on olefin aziridination. Yields were determined by ^1H NMR analysis of the crude mixture with 1,3,5-trimethoxybenzene as the internal standard.

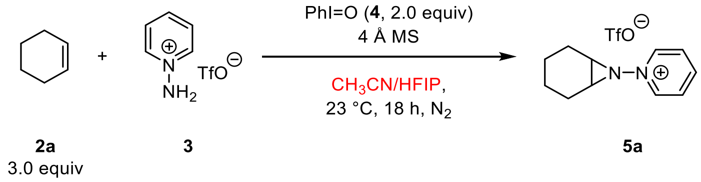

Entry	HFIP% (volume)	NMR yield
**1**	**100%**	**71%**
**2***	**100%**	**Quant.**
3	50%	61%
4	30%	56%
5	15%	63%
6	2.0 equiv	68%

* Using 1.0 equiv. of cyclohexene (**2a**).

Table S4. Control reactions varying concentrations, solvents, iodine(III) reagents, etc. Yields were determined by ^{1}H NMR analysis of the crude mixture with 1,3,5-trimethoxybenzene as the internal standard.

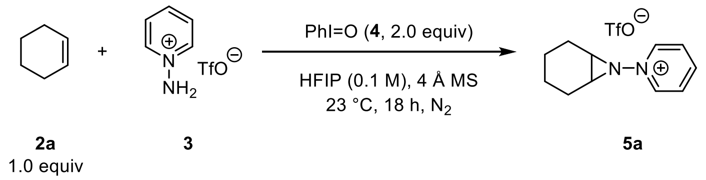

Entry	Variation	NMR yields
1	Non	99%
2	w/o 4 Å MS	85%
3	Precaution free (non-distilled HFIP under air)	73%
4	0.05 M	86%
5	0.2 M	99%
6	0.4 M	99%
7	Using TFE instead of HFIP	90%
8	Using DCE instead of HFIP	0%
9	Using MeOH instead of HFIP	0%
10	Using DMA instead of HFIP	0%
11	Using DCE instead of HFIP	0%
12	Using 1-(tert-butylsulfonyl)-2-iodosylbenzene (Protasiewicz's reagent) instead of **4**	0%
13	Using bis(trifluoroacetoxy)iodobenzene (PIFA) instead of **4**	0%
14	Using iodobenzene diacetate (PIDA) instead of **4**	66%

Table S5. Comparison of effects of MeCN and iodide catalyst in the efficiency of unactivated olefin aziridination. Reactions were carried out at 0.2 mmol scale (with respect to **2**) according to the general procedure. Condition A: PhIO (**4**, 2.0 equiv.), HFIP, 4 Å MS, 0 to 23 °C, N_2; Condition B: PhIO (**4**, 1.0 equiv.), MeCN, 4 Å MS, 23 °C, N_2; Condition C: PhIO (**4**, 1.0 equiv.), TBAI (5.0 mol%), MeCN, 4 Å MS, 23 °C, N_2. *NMR yields using 1,3,5-trimethoxybenzene as the internal standard.

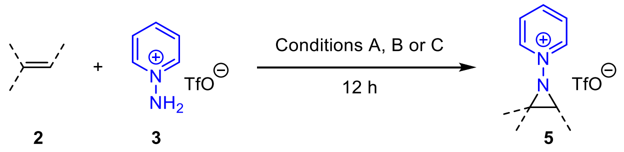

Entry	Alkene	Condition A	Condition B	Condition C
1	**2a** (cyclohexene)	76%	33%	33%
2	**2b** (cyclopentene)	62%	0%*	0%*
3	**2e** (norbornene)	69%	29%*	24%*
4	**2h** (1-octene)	72%	13%*	17%*
5	**2o** (allylbenzene)	75%	16%	16%
6	**2p** (4-phenylbutene)	70%	10%*	11%*
7	**2w** (geranyl acetate)	70%*	27%*	27%*

C.2 Problematic Olefin Substrates

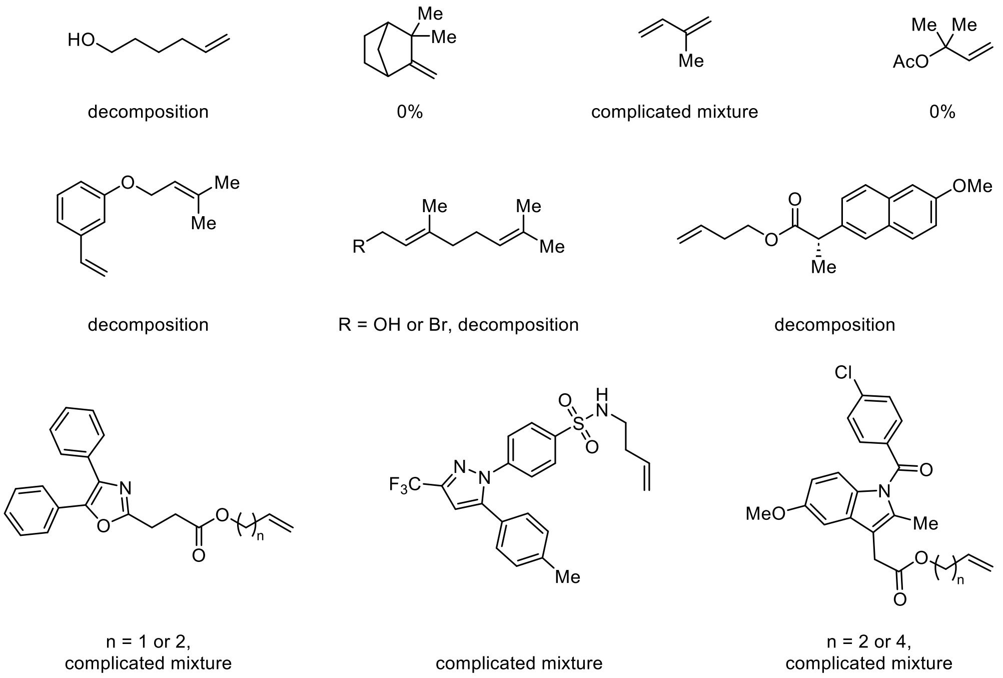

Figure S1. Olefin substrates with free OH, free NH, 1,3-dienes, or allyl esters suffer from low yields or decomposition during aziridination.

C.3 Aziridination of Cyclooctadiene

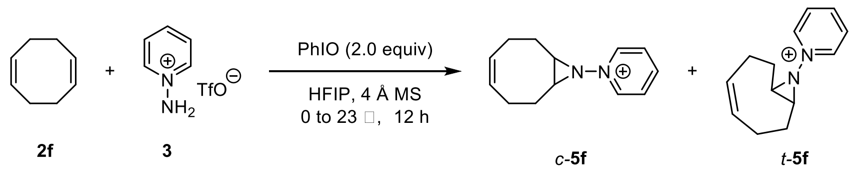

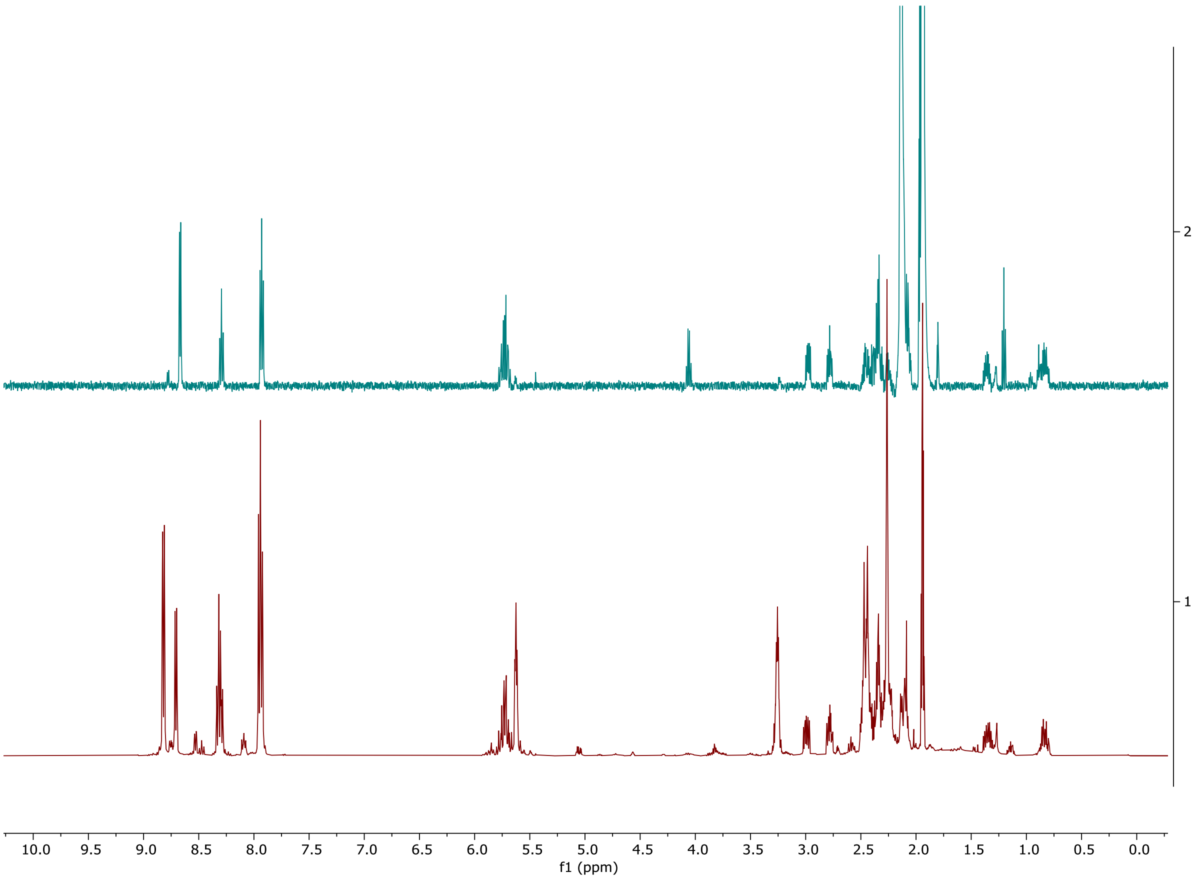

Figure S2. Aziridination with cyclooctadiene afforded a mixture of *cis*- and *trans*-aziridines. Top spectrum: ^{1}H NMR spectrum of the *trans*-aziridine (*t*-**5f**) after purification by preparative HPLC separation. Bottom spectrum: ^{1}H NMR spectrum of the mixture (*c*-**5f** and *t*-**5f**). Integrations of multiplets at δ 5.72 and 5.63 ppm indicate the *trans*- : *cis*- ratio to be 1:1.4.

C.4 Aziridination of Ethylene Gas

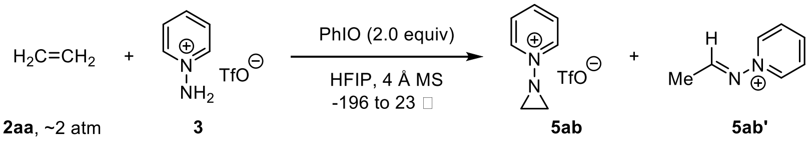

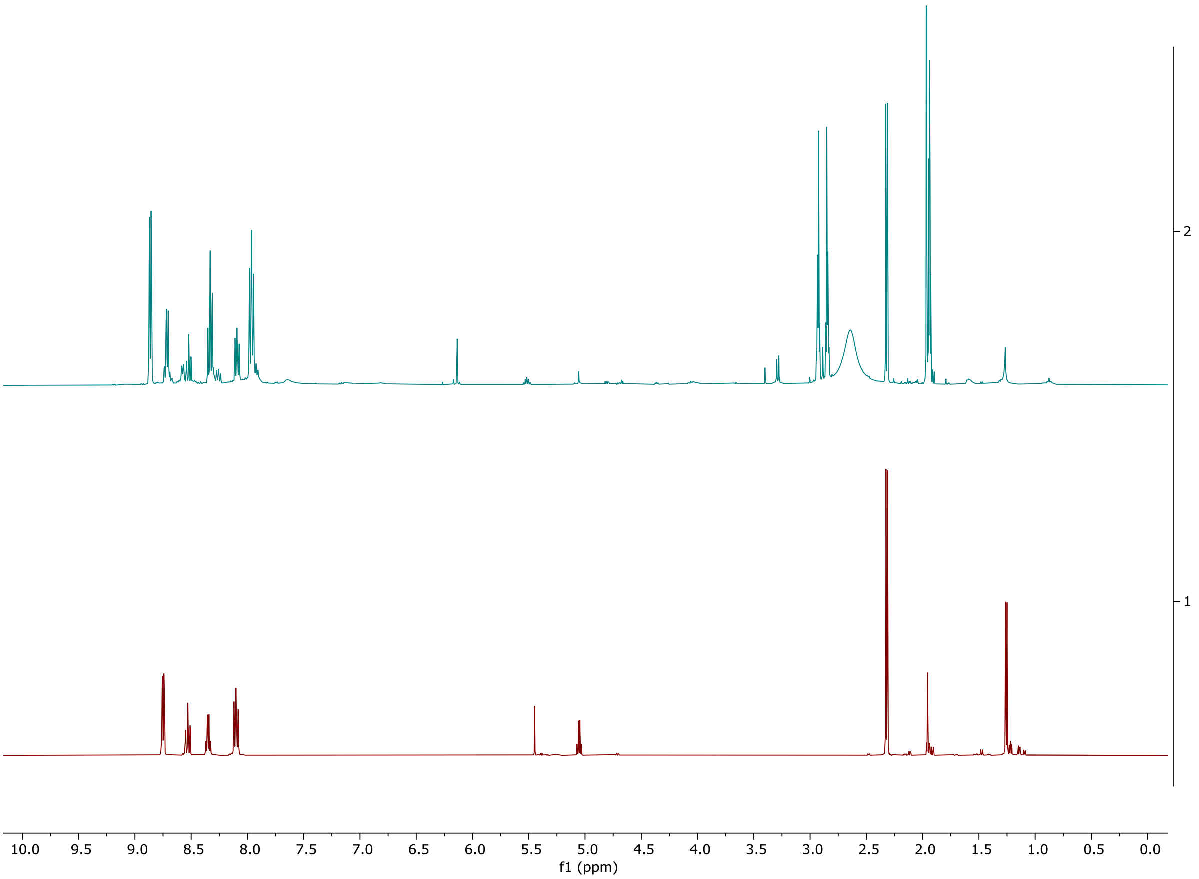

Figure S3. Ethylene gas treated according to Procedure C afforded a mixture of aziridine (5ab) and imine (5ab'). Top spectrum: ^{1}H NMR spectrum of the reaction mixture after a quick silica plug. Separation via column chromatography afforded the aziridine (**5aa**) in enriched purity (see Figure S87). Bottom spectrum: ^{1}H NMR spectrum of separately prepared imine (**5ab'**) in CD_3CN (400 MHz) at 23 °C. A mixture of **3** (73.2 mg, 0.300 mmol), acetaldehyde (100 μL), and Na_2SO_4 (100.0 mg) were stirred in CH_2Cl_2 at 0 °C for 4 h. The mixture was filtered, and the filtrate was concentrated under reduced pressure to afford a sample containing the imine (**5ab'**) in CD_3CN (400 MHz) at 23 °C. The quartet at 8.35 ppm and the doublet at 2.32 ppm indicate the presence of imine (**5ab'**), with a coupling constant J = 5.4 Hz.

D. Kinetics Analysis of Mechanism

Determination of the Reaction Order in Cyclohexene

The following analysis follows the kinetic analysis reported in the literature.[32]

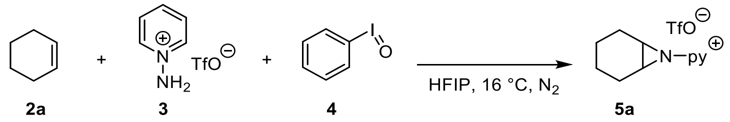

To determine the order of cyclohexene in the rate law, three reactions were carried out in which the cyclohexene concentration was varied, and the kinetic profile of aziridine formation was analyzed via a series of plots. In these plots, the x-axis is the reaction time integrated with the concentration of cyclohexene raised to reaction order α (Eqn. S1, left side), which is approximated using the trapezoid rule (Eqn. S1, right side). Meanwhile, the y-axis is the concentration profile of **5a**, and the α value was then varied to construct a set of plot. When the α value is chosen correctly for the x-axis, the different kinetic profiles of different cyclohexene concentrations will overlap.

$$\int_{t=0}^{t=n} [\mathrm{Cy}]^{\alpha} dt = \sum_{i=1}^{n} \left(\frac{[\mathrm{Cy}]_i + [\mathrm{Cy}]_{i-1}}{2}\right)^{\alpha} (t_i - t_{i-1}) \qquad \text{(S1)}$$

where:

[Cy] is the concentration of cyclohexene (mM); and

α is the reaction order of cyclohexene.

Procedure A 5-mm NMR tube was charged with cyclohexene (15, 30, or 45 mM), **3** (20 mM), and PhIO (20 mM) in dry HFIP (0.5 mL) under a nitrogen atmosphere. To this NMR tube was added a capillary containing a stock solution of 1,1,2,2-tetrachloroethane in CD_3CN. The NMR tube was allowed to equilibrate within the NMR cavity maintained at 16 °C, and the concentration of **5a** was monitored via NMR until the reaction progress exceeded four half-lives. The data is then processed, and the inverse first, zeroth, first, and second order plots are depicted in Figure S4. Figure S4b shows the best overlap, suggesting that cyclohexene is zeroth order in the rate law.

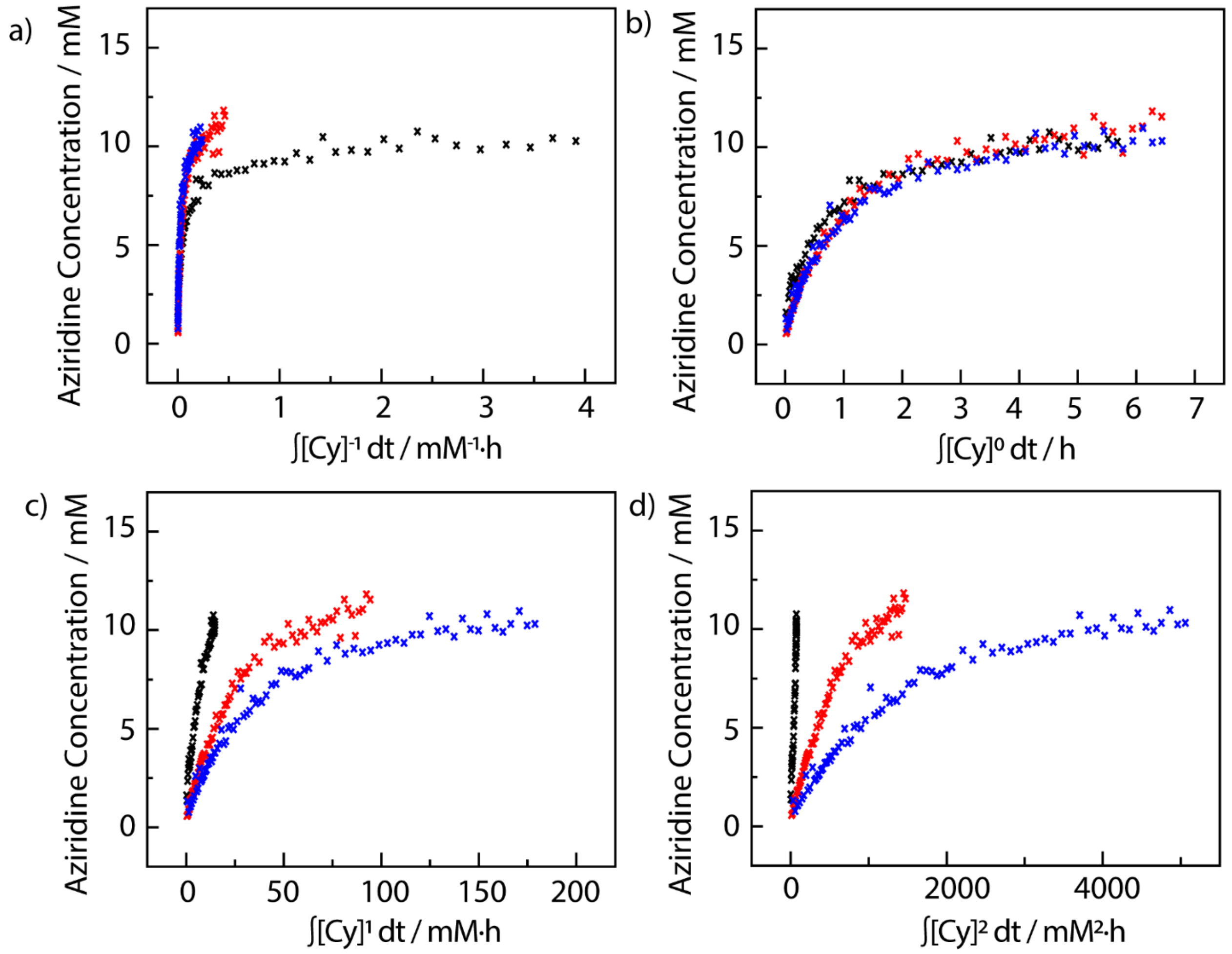

Figure S4. VTNA plots to assess the reaction order with respect to cyclohexene. Kinetic profiles of aziridine formation plotted *vs.* the time integral of cyclohexene concentrations ((×) 15 mM, (×) 30 mM, or (×) 45 mM) raised to a) inverse first, b) zeroth, c) first, and d) second order.

*Determination of the Reaction Order in N-Aminopyridinium Triflate **3***

To determine the order of **3** in the rate law, three reactions were carried out in which the concentration of **3** was varied, and the kinetic profile of aziridine formation was analyzed via a series of plots. In these plots, the x-axis is the reaction time integrated with the concentration of **3** raised to reaction order β (Eqn. S2, left side), which is approximated using the trapezoid rule (Eqn. S2, right side). Meanwhile, the y-axis is the concentration profile of **5a**, and the β value was then varied to construct a set of plot. When the β value is chosen correctly for the x-axis, the different kinetic profiles of different *N*-aminopyridinium concentrations will overlap.

$$\int_{t=0}^{t=n}[\mathrm{pyNH_2^+}]^{\beta}dt = \sum_{i=1}^{n}\left(\frac{[\mathrm{pyNH_2^+}]_i+[\mathrm{pyNH_2^+}]_{i-1}}{2}\right)^{\beta}(t_i - t_{i-1}) \qquad \text{(S2)}$$

where:

$[\mathrm{pyNH_2^+}]$ is the concentration of **3** (mM); and

β is the reaction order of **3**.

Procedure An NMR tube was charged with cyclohexene (15 mM), **3** (10, 20, or 30 mM), and PhIO (20 mM) in dry HFIP (0.5 mL) under a nitrogen atmosphere. To this NMR tube was added a capillary containing a stock solution of 1,1,2,2-tetrachloroethane in CD_3CN. The NMR tube was allowed to equilibrate within the NMR cavity maintained at 16 °C, and the concentration of **5a** was monitored via NMR until the reaction exceeded four half-lives. The data is then processed, and the inverse first, zeroth, first, and second order plot are depicted in Figure S5. Figure S5c shows the best overlap, suggesting that **3** is first order in the rate law.

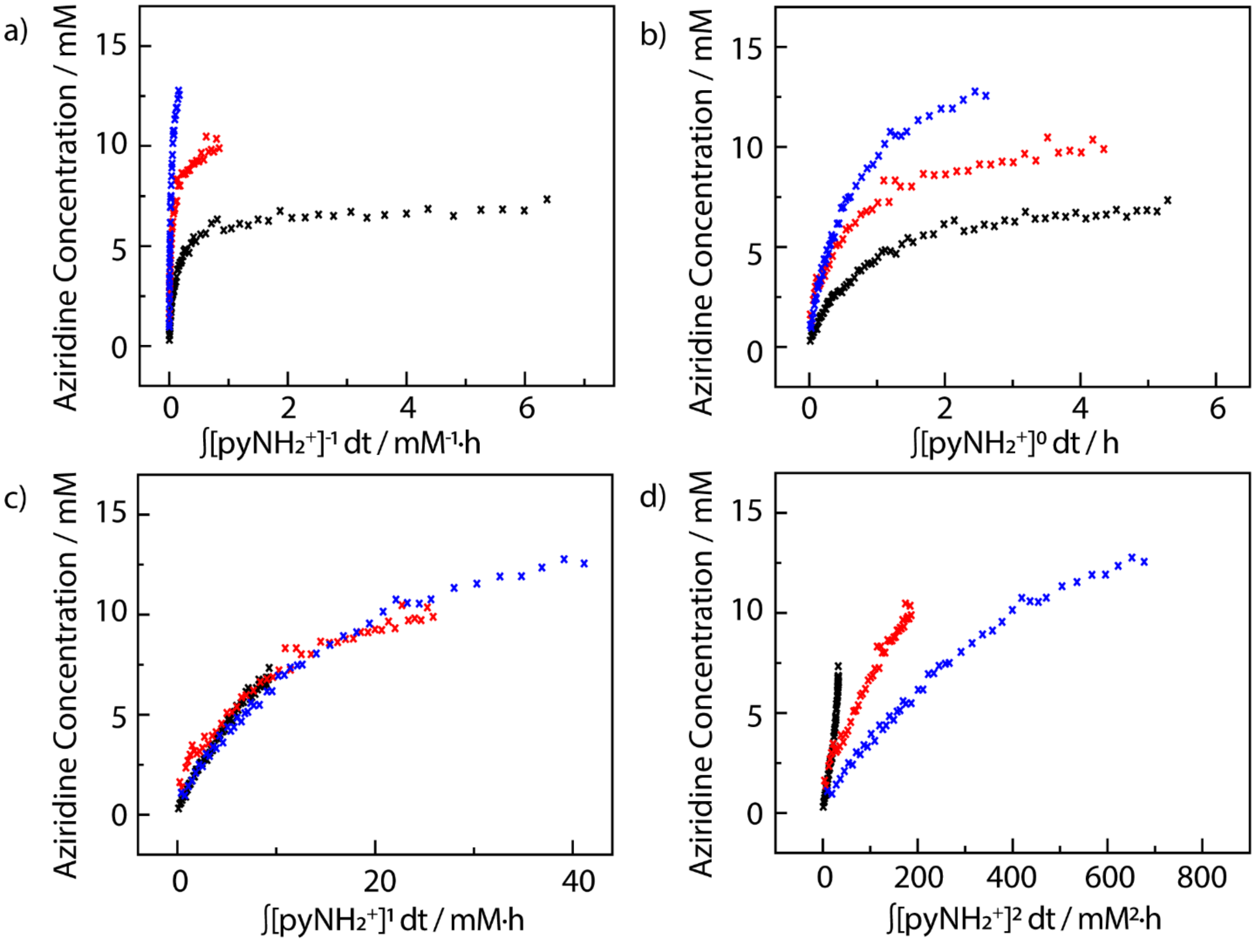

Figure S5. VTNA plots to assess the reaction order with respect to *N*-aminopyridinium triflate (3). Kinetic profiles of aziridine formation plotted *vs.* the time integral of *N*-aminopyridinium concentrations ((×) 10 mM, (×) 20 mM, or (×) 30 mM) raised to a) inverse first, b) zeroth, c) first, and d) second order.

Determination of the Reaction Order in Iodosylbenzene

To determine the order of PhIO in the rate law, three reactions were carried out in which the concentration of iodosylbenzene was varied, and the kinetic profile of aziridine formation was analyzed via a series of plots. In these plots, the x-axis is the reaction time integrated with the concentration of PhIO raised to reaction order δ (Eqn. S3, left side), which is approximated using the trapezoid rule (Eqn. S3, right side). Meanwhile, the y-axis is the concentration profile of **5a**, and the δ value was then varied to construct a set of plots. When the δ value is chosen correctly for the x-axis, the different kinetic profiles of different PhIO concentrations will overlap.

$$\int_{t=0}^{t=n} [\mathrm{PhIO}]^{\delta} dt = \sum_{i=1}^{n} \left(\frac{[\mathrm{PhIO}]_i + [\mathrm{PhIO}]_{i-1}}{2}\right)^{\delta} (t_i - t_{i-1}) \qquad \text{(S3)}$$

where:

[PhIO] is the concentration of PhIO (mM); and

δ is the reaction order of PhIO.

Procedures. an NMR tube was charged with cyclohexene (15 mM), **3** (20 mM), and PhIO (20, 30, or 40 mM) in dry HFIP (0.5 mL) under a nitrogen atmosphere. To this NMR tube was added a capillary containing a stock solution of 1,1,2,2-tetrachloroethane in CD_3CN. The NMR tube was allowed to equilibrate within the NMR cavity maintained at 16 °C, and the concentration of **5a** was monitored via NMR until the reaction exceeded four half-lives. The data is then processed, and the inverse first, zeroth, first, and second order plots are depicted in Figure S6. Figure S6c shows the best overlap, suggesting that PhIO is first order in the rate law.

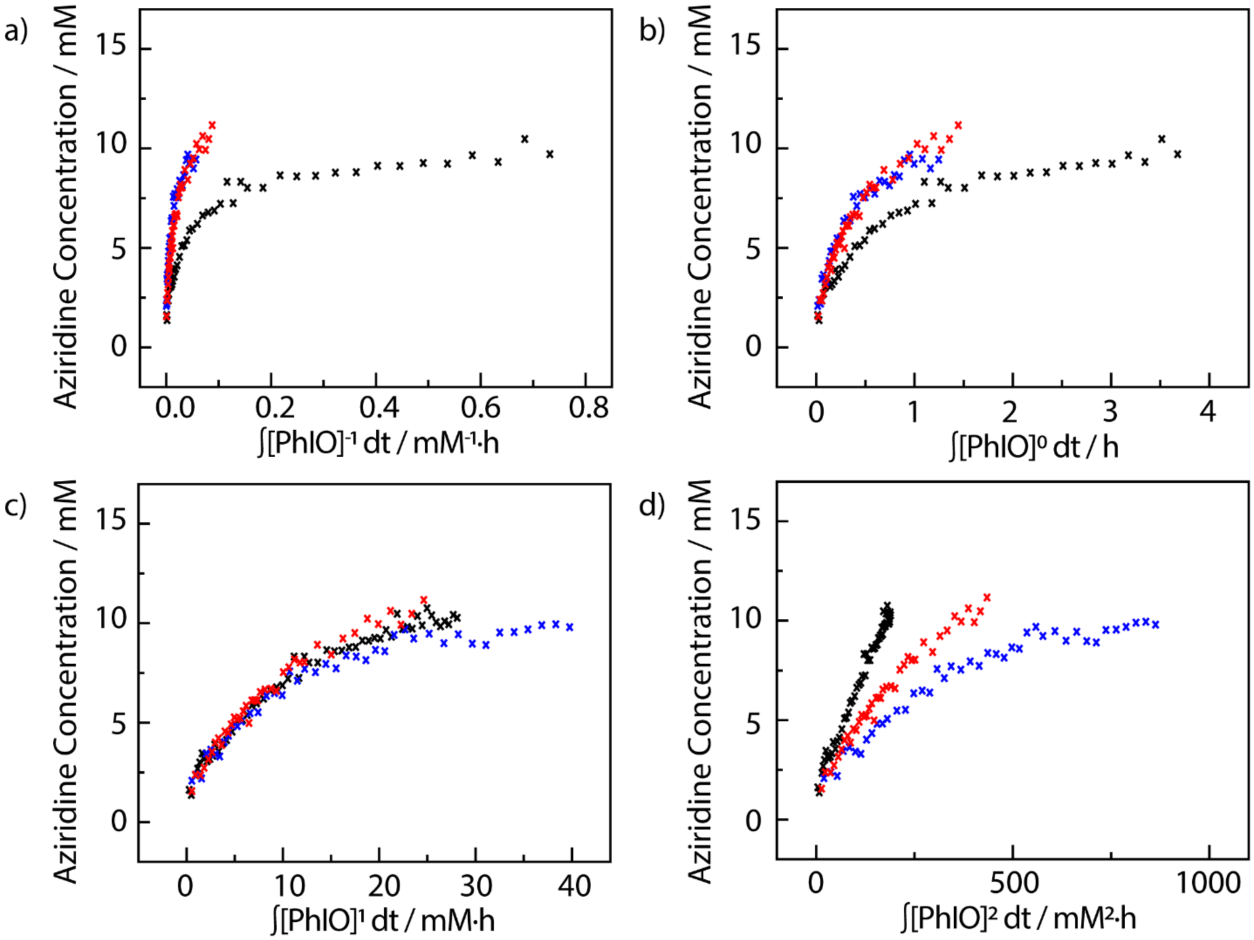

Figure S6. VTNA plots to assess the reaction order with respect to PhIO. Kinetic profiles of aziridine formation plotted *vs.* the time integral of PhIO concentrations ((×) 20 mM, (×) 30 mM, or (×) 40 mM) raised to a) inverse first, b) zeroth, c) first, and d) second order.

Determination of the Reaction Rate Constant

To determine the reaction rate constant in the rate law, an experiment was carried out in which cyclohexene was used in slight excess, and the kinetic profile of product formation were plotted versus the time integral of the concentration of all reactants raised to the corresponding orders, which was approximate by the trapezoid rule (Eqn. S4). The slope of the linear fit provides the reaction rate constant k_{obs}.

$$\int_{t=0}^{t=n} [\mathrm{Cy}]^{\alpha}[\mathrm{pyNH_2^+}]^{\beta}[\mathrm{PhIO}]^{\delta} dt =$$

$$\sum_{i=1}^{n} \left(\frac{[Cy]_i+[Cy]_{i-1}}{2}\right)^{\alpha} \left(\frac{[\mathrm{pyNH_2^+}]_i+[\mathrm{pyNH_2^+}]_{i-1}}{2}\right)^{\beta} \left(\frac{[\mathrm{PhIO}]_i+[\mathrm{PhIO}]_{i-1}}{2}\right)^{\delta} (t_i - t_{i-1}) \quad \text{(S4)}$$

where:

[Cy] is the concentration of cyclohexene (mM).

[$pyNH_2^+$] is the concentration of **3** (mM).

[PhIO] is the concentration of PhIO (mM).

α=0 is the reaction order of cyclohexene.

β=1 is the reaction order of **3**.

δ=1 is the reaction order of PhIO.

Procedure An NMR tube was charged with cyclohexene (30 mM), **3** (20 mM), and PhIO (20 mM) in dry HFIP (0.5 mL) under a nitrogen atmosphere. To this NMR tube was added a capillary containing a stock solution of 1,1,2,2-tetrachloroethane in CD_3CN. The NMR tube was allowed to equilibrate within the NMR cavity maintained at 16 °C, and the concentration of each component (cyclohexene, **3**, PhI, **5a**, and PhIO) were monitored via NMR until the reaction exceeded four half-lives. The data was then processed, and the plot of concentration of aziridine product *vs.* the time integral is depicted in Figure S7, affording a linear fit (R^2=0.991) with a slope of 0.0424 $mM^{-1}h^{-1}$or 0.0118 $M^{-1}s^{-1}$.

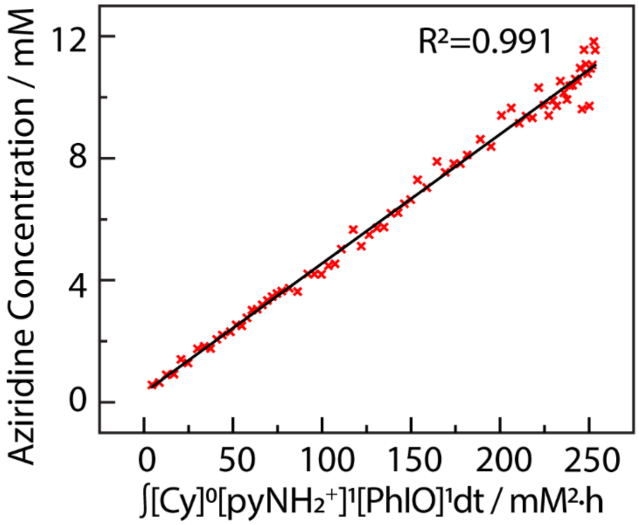

Figure S7. VTNA plot of aziridine concentration (×) *vs.* the time integral to determine the reaction rate constant in the aziridination of cyclohexene using 3 and PhIO.

E. Hammett Analyses

Hammett Analysis of para-Substituted Iodosylarene in the Aziridination of Cyclohexene

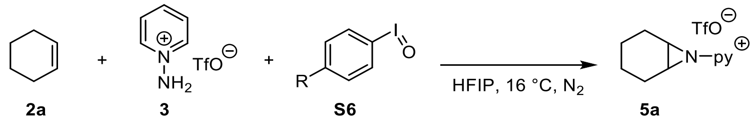

The rate constant for the aziridination of cyclohexene using *para*-substituted iodosylarene (**S6**) and **3** was obtained similarly to the aziridination of cyclohexene using **3** and PhIO using Eqn. S4 (*vide infra*). The plots of concentration of aziridine product *vs.* the time integral is depicted in Figure S8.

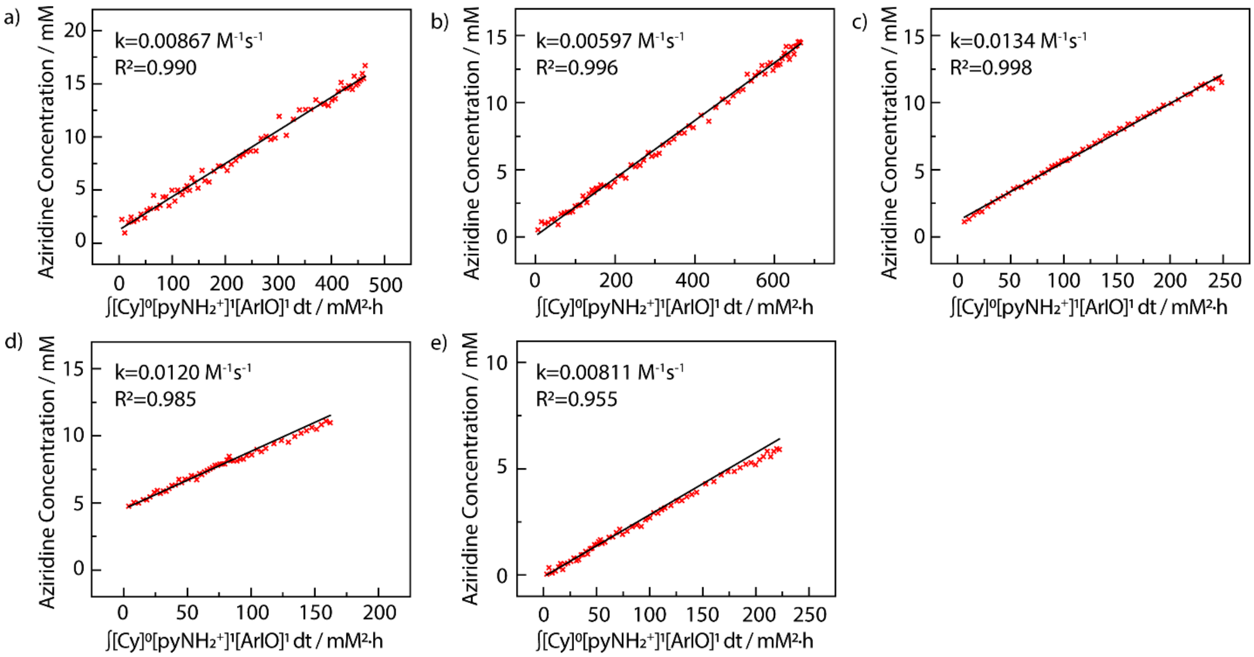

Figure S8. VTNA plot of aziridine concentration (×) *vs.* the time integral to determine the reaction rate constant in the aziridination of cyclohexene using 3 and substituted iodosylarene S6. Substitution at the *para*-position R= (a) CN, (b) NO_2, (c) OMe, (d) Me, and (e) Br. Reaction conditions: cyclohexene (30 mM), **3** (20 mM), and **S6** (20 mM) in dry HFIP (0.5 mL) under a nitrogen atmosphere in an NMR tube charged with a capillary containing a stock solution of triethyl benzene-1,3,5-tricarboxylate in CD_3CN at 16 °C. Note: for R=Br, the concentration of **S6a** was 10 mM due to the solubility limitations.

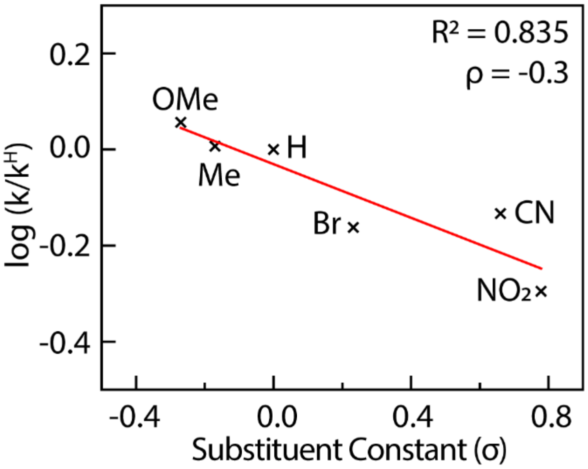

Figure S9. Hammett plot depicting the effect of *para*-substituent of iodosylbenzene on the observed rate constant in the aziridination of cyclohexene.

Determination of the Reaction Rate Constant in the Aziridination of Cyclohexene using N-Aminopyridinium Mesitylsulfonate

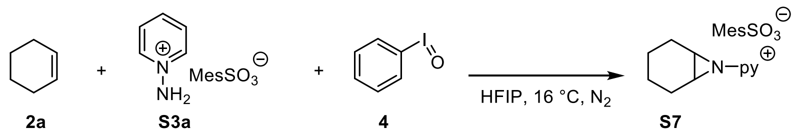

The rate constant for the aziridination of cyclohexene using *N*-aminopyridinium mesitylsulfonate (**S3a**) and PhIO was obtained similarly to the aziridination of cyclohexene using *N*-aminopyridinium triflate (**3**) and PhIO using Eqn. S4 (*vide infra*). The plot of concentration of aziridine product *vs.* the time integral is depicted in Figure S10, affording a linear fit (R^2=0.999) with a slope of 0.0507 $mM^{-1}h^{-1}$or 0.0141 $M^{-1}s^{-1}$.

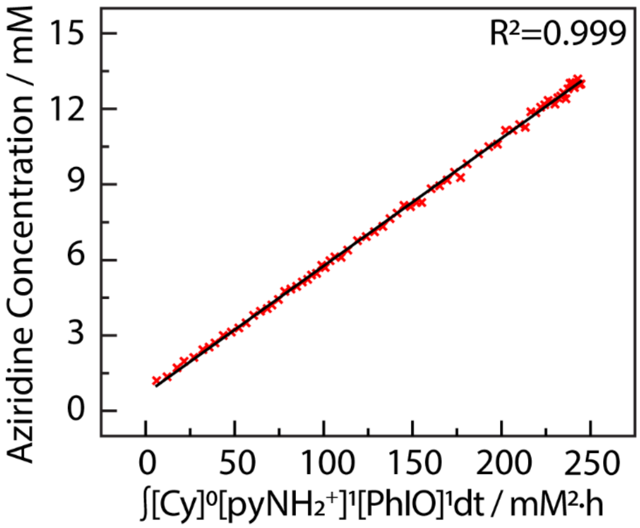

Figure S10. VTNA plot of aziridine concentration (×) *vs.* the time integral to determine the reaction rate constant in the aziridination of cyclohexene using *N*-aminopyridinium mesitylsulfonate S3a and PhIO. Reaction conditions: cyclohexene (30 mM), **S3a** (20 mM), and PhIO (20 mM) in dry HFIP (0.5 mL) under a nitrogen atmosphere in an NMR tube charged with a capillary containing a stock solution of triethyl benzene-1,3,5-tricarboxylate in CD_3CN at 16 °C.

Hammett Analysis of para-Substituted N-Aminopyridinium Mesitylsulfonate in the Aziridination of Cyclohexene

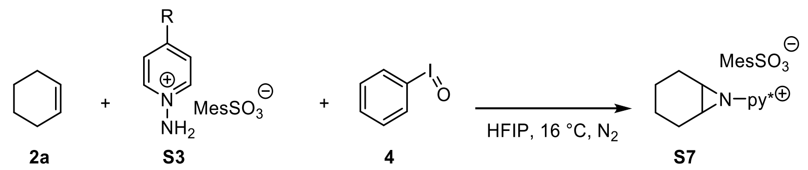

The rate constant for the aziridination of cyclohexene using *para*-substituted *N*-aminopyridinium mesitylsulfonate salts (**S3**) and PhIO was obtained similarly to that using *N*-aminopyridinium triflate (**3**) and PhIO using Eqn. S4 (*vide infra*). The plots of concentration of aziridine product *vs.* the time integral is depicted in Figure S11.

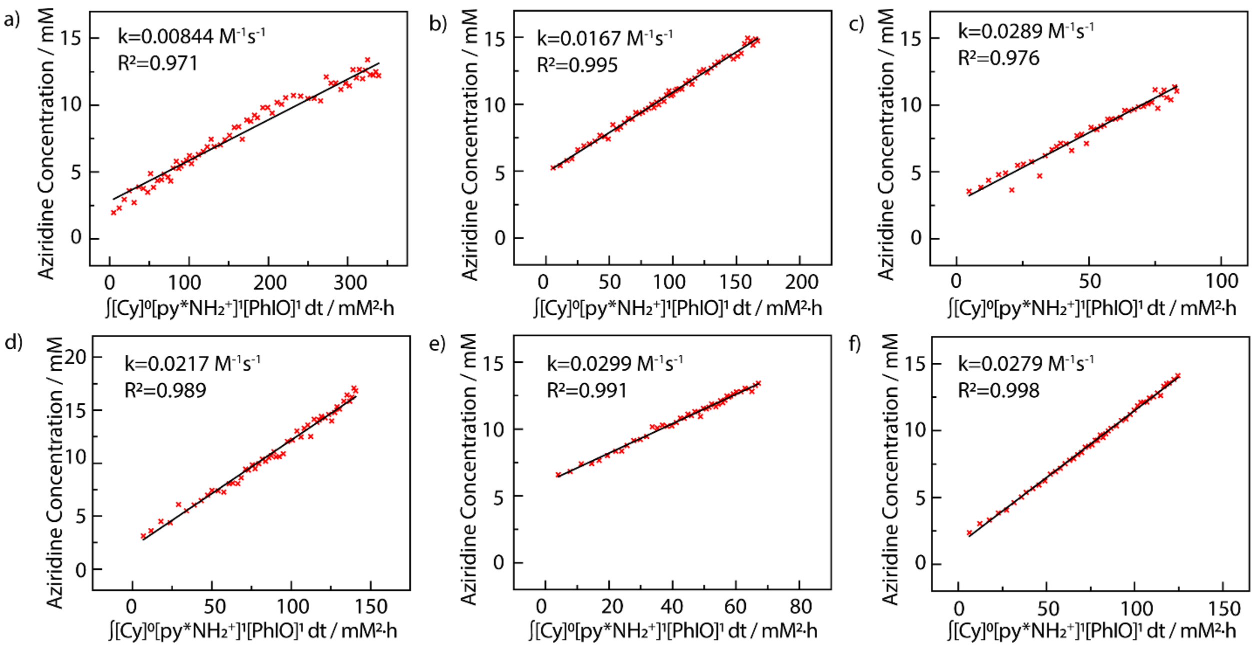

Figure S11. VTNA plot of aziridine concentration (×) *vs.* the time integral to determine the reaction rate constant in the aziridination of cyclohexene using substituted *N*-aminopyridinium mesitylsulfonate S3 and PhIO. Substitution at the *para*-position R= (a) CF_3, (b) Me, (c) OMe, (d) Cl, (e) Ph, and (f) ^{t}Bu. Reaction conditions: cyclohexene (30 mM), **S3** (20 mM), and PhIO (20 mM) in dry HFIP (0.5 mL) under a nitrogen atmosphere in an NMR tube charged with a capillary containing a stock solution of triethyl benzene-1,3,5-tricarboxylate in CD_3CN at 16 °C. Figure 4 in the main text describes the Hammett plot including only non-conjugative substituents. Figure S12 depicts the Hammett plot for all substituents. Note: *N*-aminopyridinium salts bearing *para*-substitution R=NO_2, CN, and Me_2N were also subjected to the aforementioned conditions, but these reactions afforded a complex mixture of products.

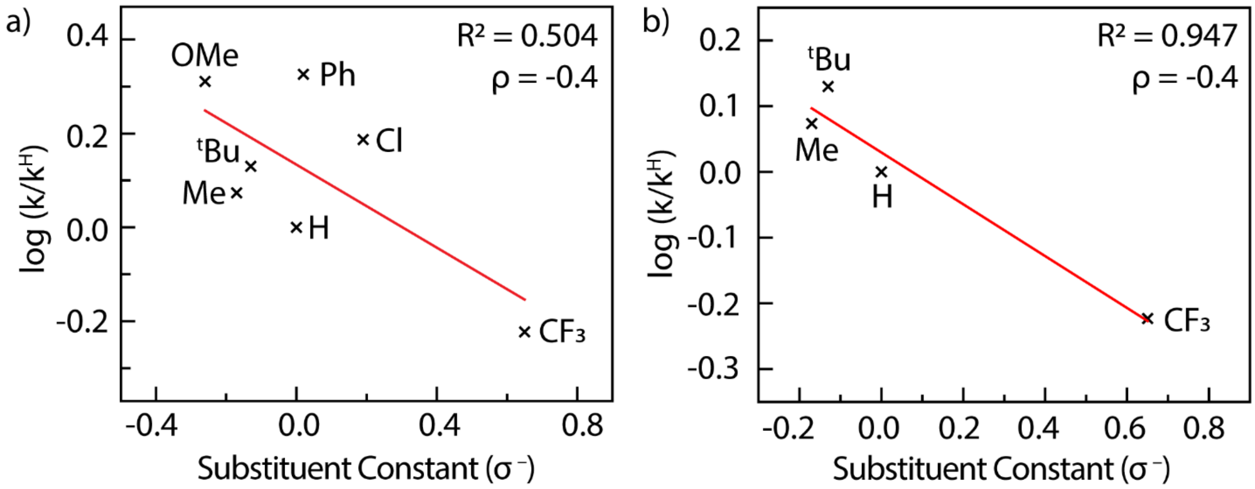

Figure S12. Hammett plot depicting the effect of *para*-substituent of *N*-aminopyridinium mesitylsulfonate on the observed rate constant in the aziridination of cyclohexene. a) Analysis with conjugated and non-conjugated substituents, and b) analysis with non-conjugated substituents. Because the amino group of 3 has a strong π-donating effect,[33] conjugated *para*-substituents, especially π-acceptors, afforded poor correlation (Figure S12a). A linear Hammett correlation was obtained with $\rho = -0.4$ ($R^2 = 0.947$, Figure 4b) when σ^- values were used (S12b).

F. Chemical Analysis of Mechanism

F.1 Mass Spectrometry Evidence of Iminoiodinane

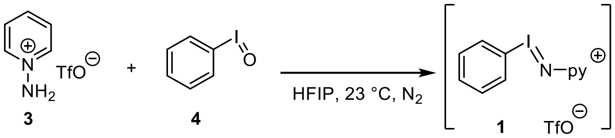

A test tube was charged with *N*-aminopyridinium triflate (**3**, 9.8 mg, 0.040 mmol, 1.0 equiv.) in dry HFIP (1.0 mL). A second test tube was charged with PhIO (8.8 mg, 0.040 mmol, 1.0 equiv.) in dry HFIP (1.0 mL). The contents of the two test tubes were mixed, and an aliquot the resulting mixture was analyzed by ESI(+) mass spectrometry.

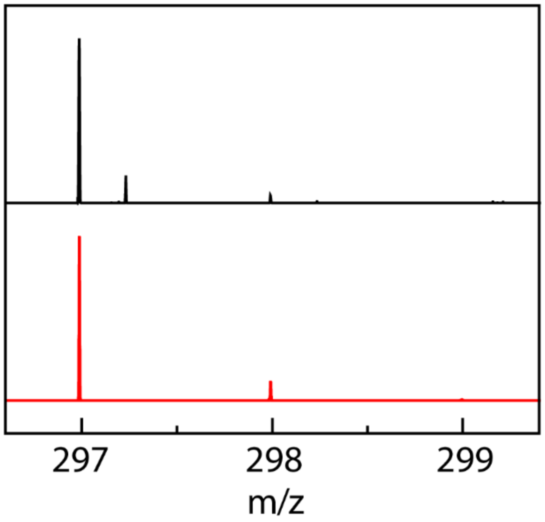

Figure S13. Mass Spectrometric Analysis of a Mixture of PhIO and 3. HRMS-ESI: measured mass $[M]^+$ = 296.9877 (—) and simulated mass $[M]^+$ = 296.9883 (—). *The peak observed at 297.2392 is consistent with the empirical formula $C_{44}H_{40}I_4N_{8}{}^{4+}$.

F.2 Competition Aziridination Altering Nitrogen Sources

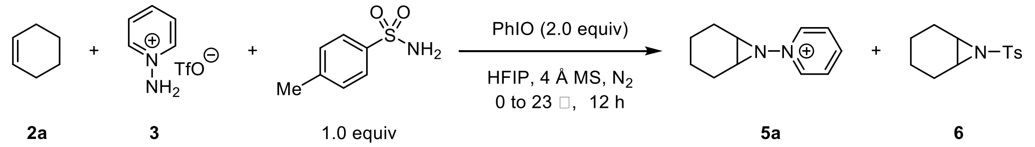

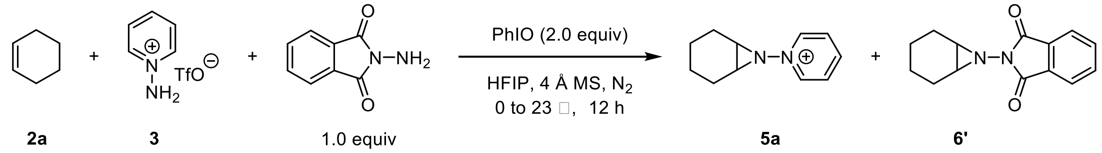

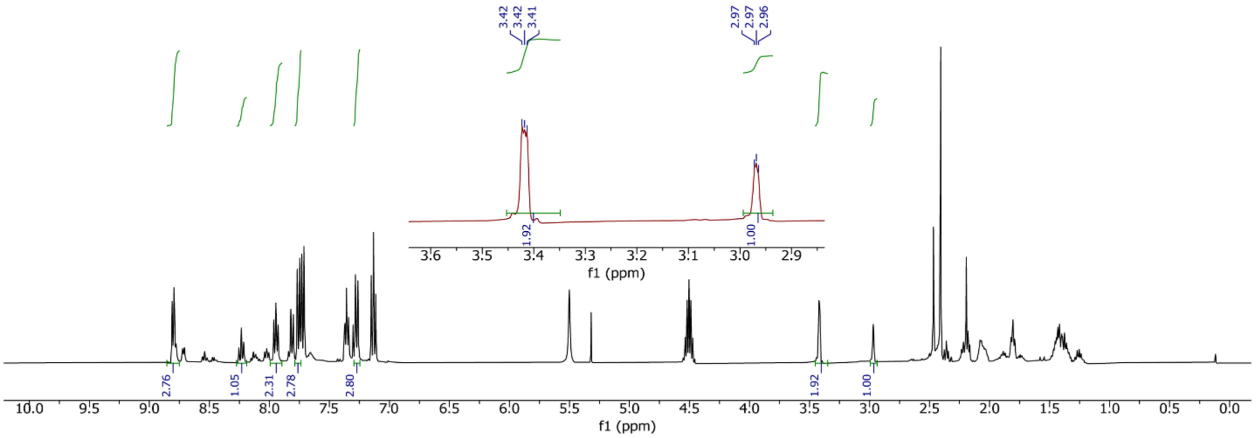

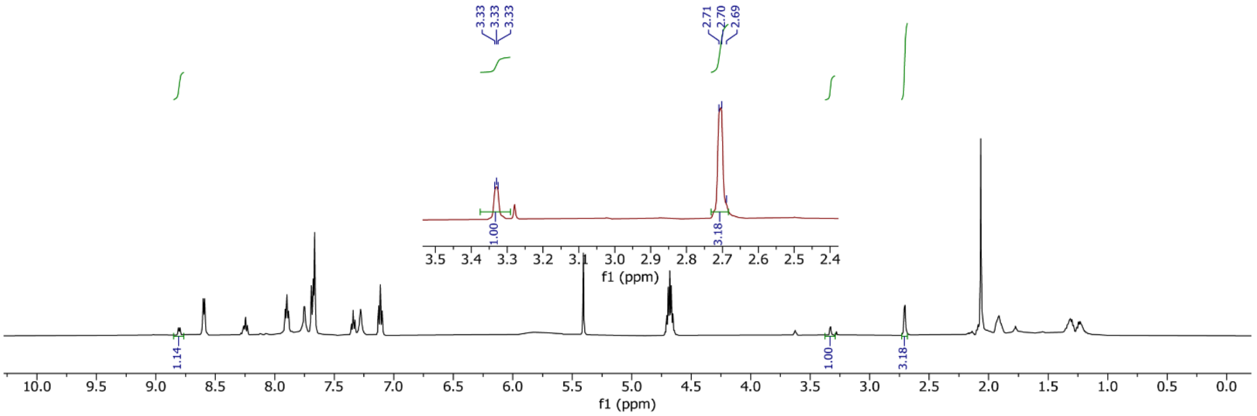

Figure S14. Competition between $TsNH_2$, 3, and $PhthNH_2$ in the aziridination of cyclohexene revealed the relative reactivity to be 0.53 : 1.00 : 3.18 ($TsNH_2$: 3 : $PhthNH_2$). The aziridination with competing nitrogen sources was carried out according to the general procedure, using cyclohexene (0.200 mmol, 1.00 equiv.), each of the nitrogen sources (0.200 mmol, 1.00 equiv.), and PhIO (0.400 mmol, 2.00 equiv.). After workup, an aliquot of the crude mixture was taken and analyzed by ^{1}H NMR, and the product ratio was determined by the ratio of the integration of the aziridine C–H bonds.

F.3 Aziridination with Kinetic Probe Substrates

Aziridination of trans-(2-vinylcyclopropyl)benzene

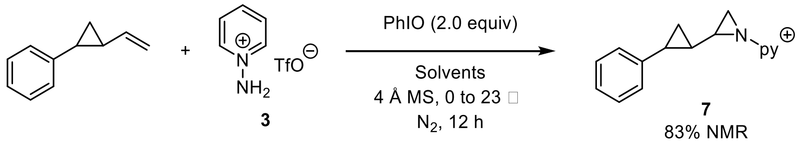

Trans-(2-vinylcyclopropyl)benzene oligomerized in HFIP. The aziridination was carried out in trifluoroethanol (TFE) instead, resulting in complicated mixture, where ring-opening was observed. Similar results were observed when carried out in CH_3CN. Similarly complex mixtures have been reported in previous studies.[34]

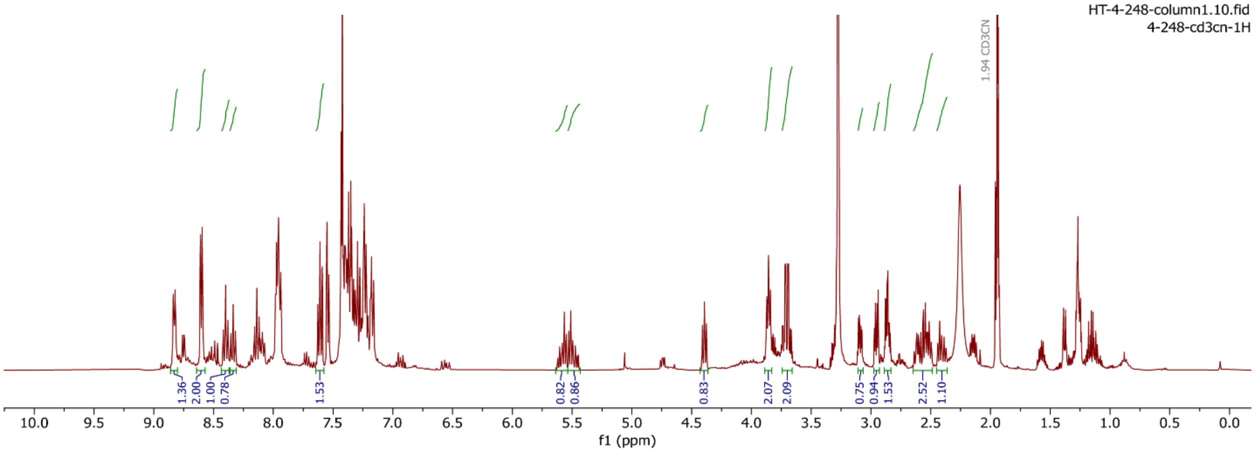

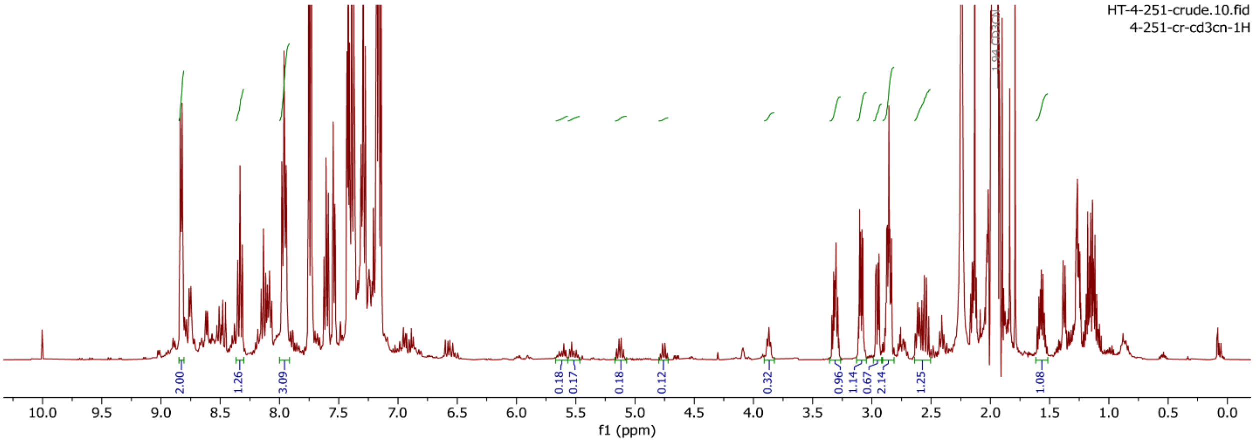

Figure S15. Aziridination of *trans*-(2-vinylcyclopropyl)benzene. Aziridination was carried out according to the general procedure using 0.200 mmol of *trans*-(2-vinylcyclopropyl)benzene. Top spectrum: crude NMR of the reaction in TFE. Bottom spectrum: crude NMR of the reaction in CH_3CN.

Aziridination with cis-2-hexene

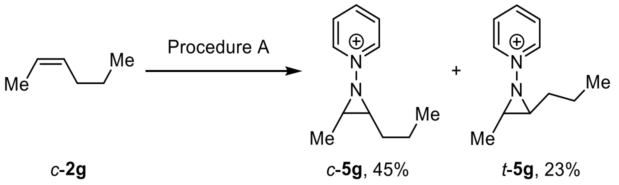

H	δ (ppm)	range	integration	multiplicity	*J* (Hz)
D (q)	2.95	2.99 .. 2.91	0.51	q	5.94, 5.94, 6.09
C (p)	2.82	2.87 .. 2.78	0.53	p	5.92, 5.92, 5.90, 5.90
B (dt)	3.10	3.14 .. 3.07	0.94	dt	6.66, 6.66, 8.74
A (dq)	3.20	3.26 .. 3.16	1.00	dq	5.94, 5.94, 5.91, 8.71

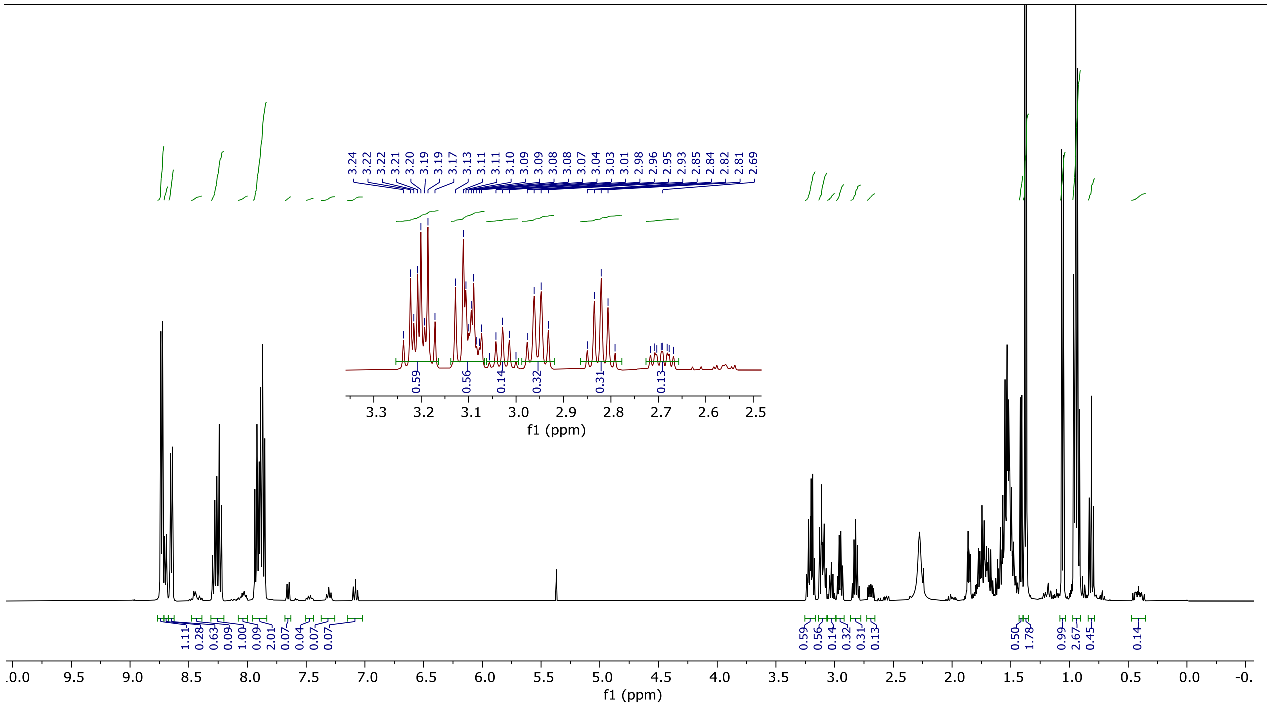

Figure S16. Aziridination was carried out according to the general procedure using 0.300 mmol of *cis*-2-hexene. The mixture was purified by silica gel flash chromatography and afforded a mixture of *cis*- and *trans*-aziridines in a 2:1 ratio. The *cis*- and *trans*- isomers were differentiated by the coupling constants of the C–H bonds in the aziridine ring. The *cis*-isomer has greater $^3J_{H-H}$ than that of the *trans*-isomer.

Aziridination with trans-2-hexene

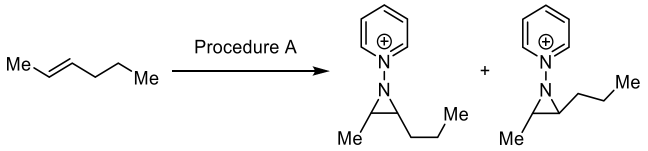

t-**2g** *c*-**5g**, 35% *t*-**5g**, 42%

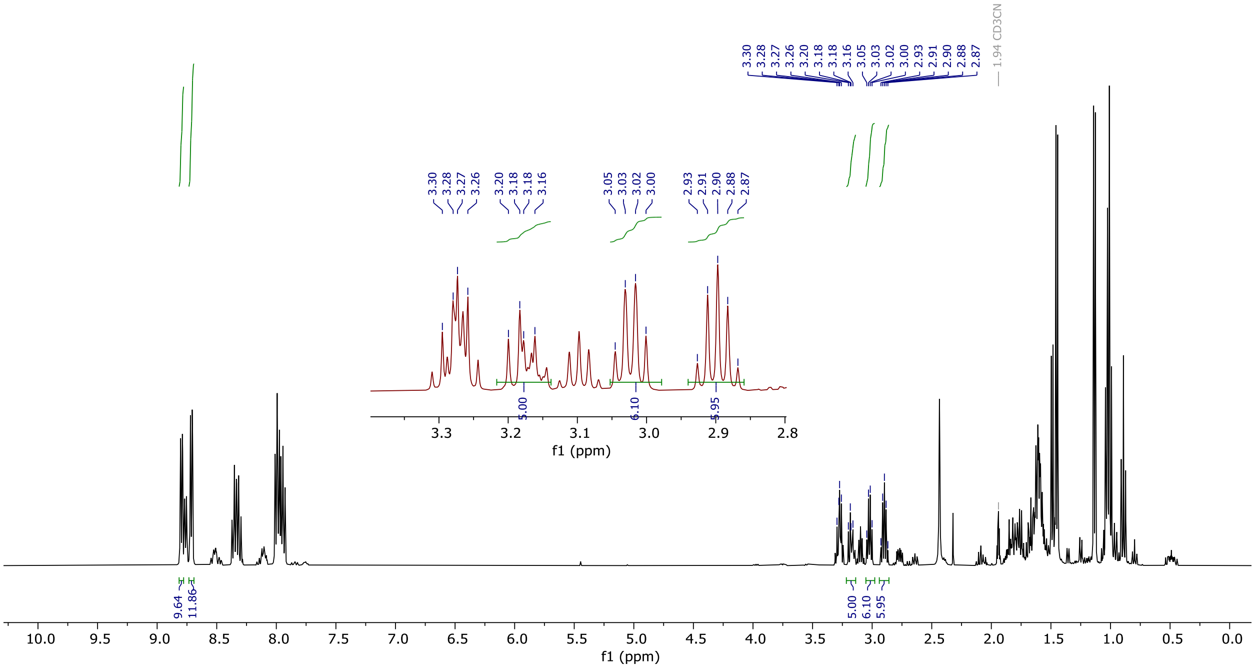

Figure S17. Aziridination was carried out according to the general procedure using 0.300 mmol of *cis*-2-hexene. The mixture was purified by silica gel flash chromatography and afforded a mixture of *cis*- and *trans*-aziridines in a 5:6 ratio. The *cis*- and *trans*- isomers were differentiated by the coupling constants of the C–H bonds in the aziridine ring. The *cis*-isomer has greater $^3J_{H-H}$ than that of the *trans*-isomer.

Aziridination of Cyclohexene in the Presence of PBN

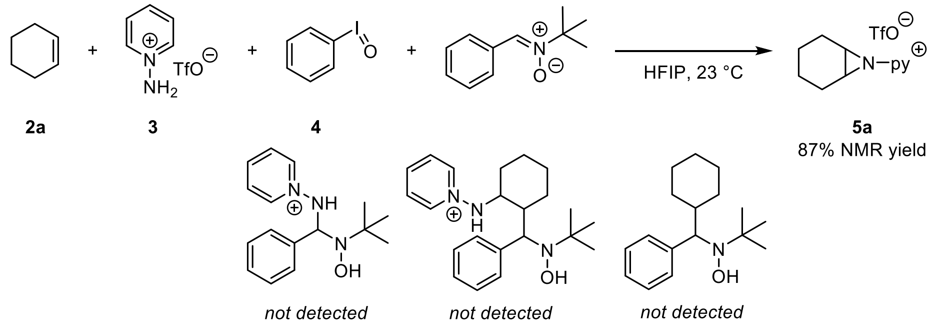

Figure S18. Aziridination of Cyclohexene in the Presence of PBN. A 20-mL scintillation vial was charged with *N*-aminopyridinium triflate **3** (24.4 mg, 0.100 mmol, 1.00 equiv.), cyclohexene (15.0 µL, 0.148 mmol, 1.48 equiv.), PBN (17.7 mg, 0.100 mmol, 1.00 equiv.), and dry HFIP (1.0 mL). To this vial was added a solution of PhIO (44.0 mg, 0.200 mmol, 2.00 equiv.) dissolved in dry HFIP (1.0 mL), and the reaction mixture was stirred for 5h at 23 °C. An aliquot (0.1 mL) was taken, diluted with 0.5 mL CD_3CN, and subjected to ^1H NMR analysis. The aziridine product was formed in 87% NMR yield along with quantitative unreacted PBN. A different aliquot was taken and subjected to mass spectrometry, and no trapped radical species was found.

Iodide Catalyzed Aziridination of Styrene in the Presence of PBN

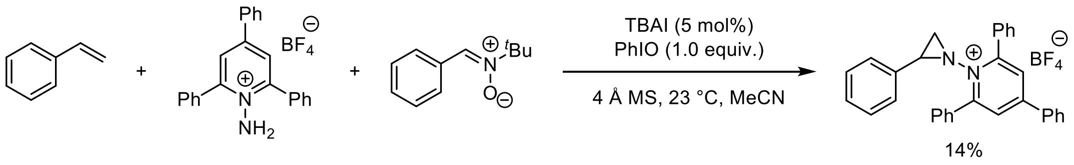

Figure S19. Aziridination of Styrene in the Presence of PBN. A 20-mL scintillation vial was charged with TBAI (3.7 mg, 0.010 mmol 5.0 mol%), 1-amino-2,4,6-triphenylpyridinium tetrafluoroborate (82.0 mg, 0.200 mmol, 1.00 equiv.), styrene (21.0 mg, 0.200 mmol, 1.00 equiv.), PBN (35.4 mg, 0.200 mmol, 1.00 equiv.), and dry CH_3CN (1.0 mL). To this vial was added PhIO (44.0 mg, 0.200 mmol, 1.00 equiv.), and the reaction mixuture was stirred for 12 h at 23 °C. An aliquot (0.1 mL) was taken, diluted with 0.5 mL of CD_3CN, and subjected to ^1H NMR analysis. The aziridine product was formed in 14 % NMR yield.

F.4 Decomposition Kinetics of PhIO and *N*-Aminopyridinium Triflate

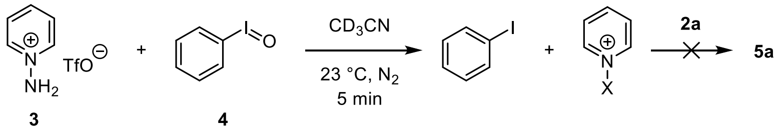

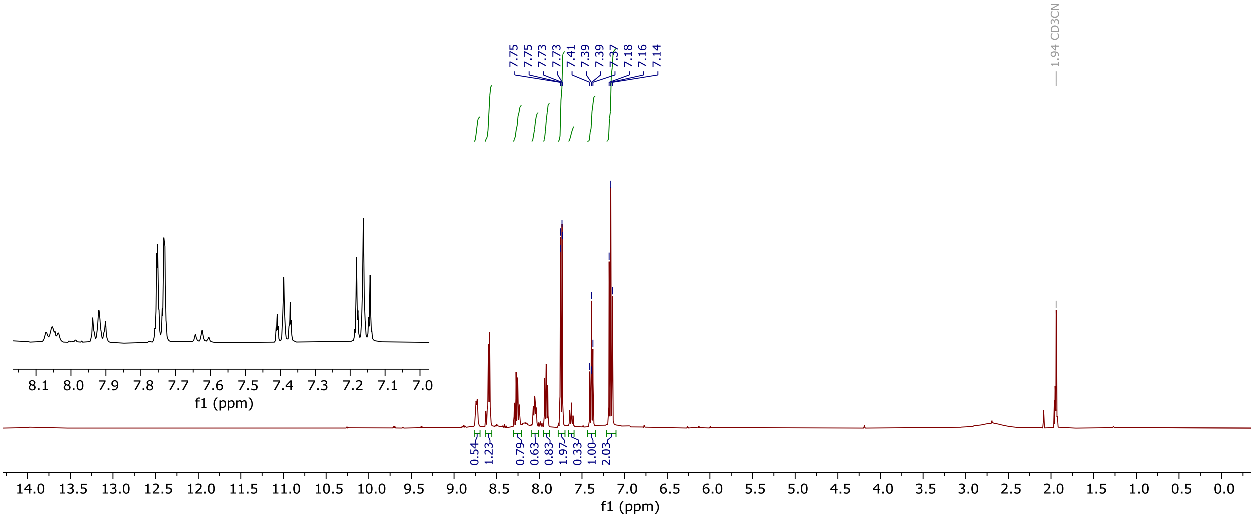

Figure S20. An NMR experiment showed quick decomposition upon mixing 3 and 4 in CD_3CN. In an N_2 filled glovebox, aminopyridinium triflate (**3**, 0.10 mmol) in CD_3CN (0.60 mL) was added to iodosylbenzene (**4**, 0.10 mmol) in a 20-mL scintillation vial. The mixture was kept settled before transferring to an NMR tube, and a ^1H spectrum was acquired. To a separately prepared mixture was added cyclohexene (**2a**, 0.10 mmol), where no aziridine product **5a** was observed.

Procedures: A test tube was charged with *N*-aminopyridinium triflate (**3**, 4.9 mg, 0.020 mmol, 1.0 equiv.) in dry HFIP (1.0 mL). A second test tube was charged with PhIO (4.4 mg, 0.020 mmol, 1.0 equiv.) in dry HFIP (1.0 mL). The contents of the two test tubes were mixed, and a 0.50 mL aliquot the resulting mixture was transferred to an NMR tube. To this NMR tube was added a capillary containing a stock solution of 1,1,2,2-tetrachloroethane in CD_3CN. The NMR tube was allowed to equilibrate within the NMR cavity maintained at 16 °C, and the concentration of both **3** and PhIO were monitored. The data is detailed in Figure S21. A similar experiment was conducted using CD_3CN as the solvent instead of HFIP; however, the data was complicated due to the decomposition completing within 5 min and the low solubility of PhIO in CD_3CN.

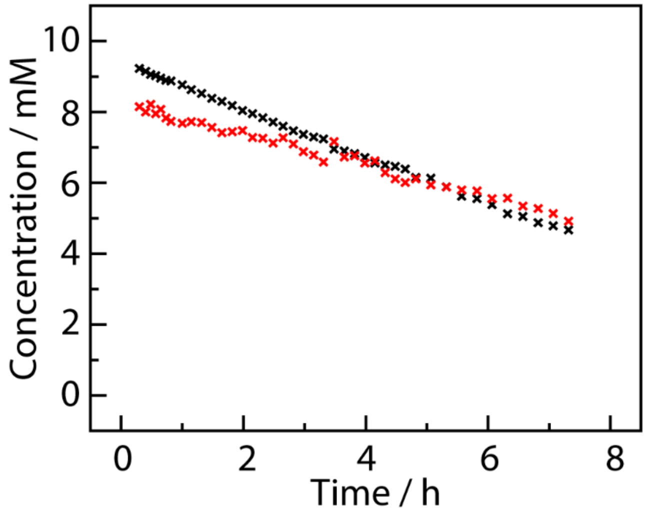

Figure S21. Plot of [PhIO] (×) and [3] (×) in HFIP, showing their slow decomposition in HFIP over time.

F.5 Alternative Aziridination Mechanism

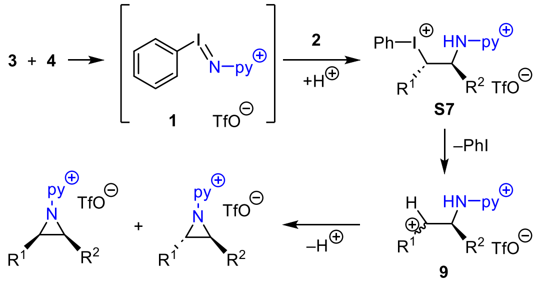

Figure S22. Alternative reaction mechanism for aziridination via 1. Rate-determining reaction of PhIO with **3** generates highly reactive *N*-pyridinium iminoiodinane **1**, which subsequently reacts with olefin **2**. Olefin addition to **1** could proceed via either a pinacol-type iodonium intermediate **S7** or via concerted nitrene transfer.

G. X-Ray Diffraction Data

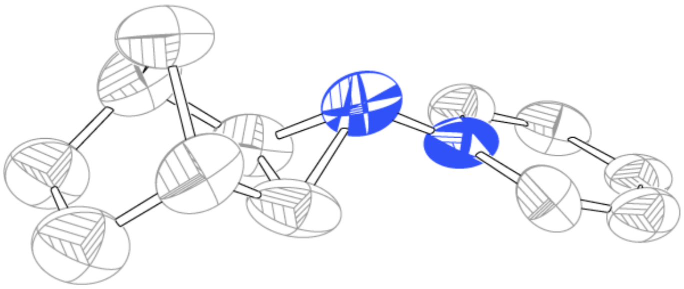

Figure S23. Thermal ellipsoid plot of **5e** plotted at 50% probability. H-atoms and TfO$^-$ counterion are removed for clarity. The crystalline sample used for the diffraction experiment was obtained via slow evaporation of a CH_2Cl_2 solution at −20 °C.

Table S6. X-ray experimental details for **5e** (CCDC 2347026).

Crystal data	
Chemical formula	$CHF_3O_3S{\cdot}CF_3O_3S{\cdot}2(C_{12}H_{15}N_2)$
M_r	673.67
Crystal system, space group	Triclinic, $P\bar{1}$
Temperature (K)	100
a, *b*, *c* (Å)	9.684(1), 9.871(1), 15.803(3)
α, β, γ (°)	78.84(1), 79.73(2), 85.28(1)
V (Å^3)	1456.5(4)
Z	2
Radiation type	Cu *K*α
μ (mm^{-1})	2.44
Crystal size (mm)	0.2 × 0.1 × 0.1
Data collection	
Diffractometer	XtaLAB Synergy, Dualflex, HyPix
Absorption correction	Multi-scan<br>*CrysAlis PRO* 1.171.43.107a (Rigaku Oxford Diffraction, 2024) Empirical absorption correction using spherical harmonics, implemented in SCALE3 ABSPACK scaling algorithm.
T_{min}, T_{max}	0.658, 1.000
No. of measured, independent and observed [$I > 2\sigma(I)$] reflections	10259, 4418, 1676
R_{int}	0.205
$(\sin \theta/\lambda)_{max}$ (Å^{-1})	0.580
Refinement	
$R[F^2 > 2\sigma(F^2)]$, $wR(F^2)$, S	0.157, 0.460, 1.15
No. of reflections	4418
No. of parameters	398
H-atom treatment	H-atom parameters constrained
$\Delta\rho_{max}$, $\Delta\rho_{min}$ (e Å^{-3})	1.08, −0.48

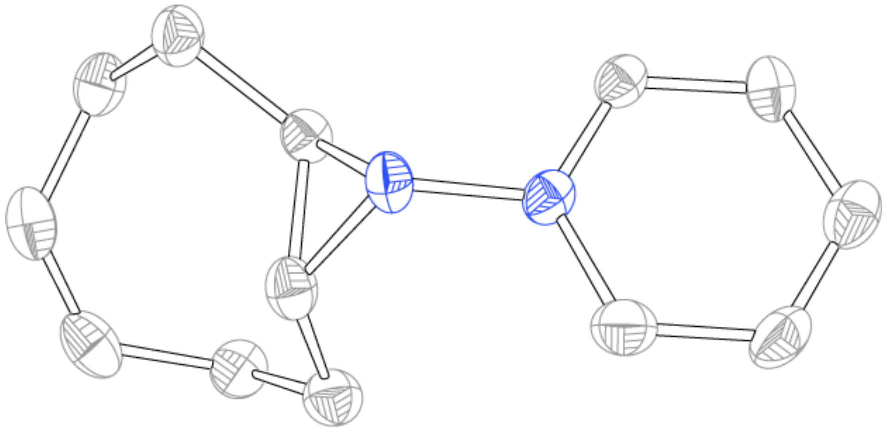

Figure S24. Thermal ellipsoid plot of *t*-**5f** plotted at 50% probability. H-atoms and TfO^- counterion are removed for clarity. The crystalline sample used for the diffraction experiment was obtained via Et_2O layering to an CH_3CN solution.

Table S7. X-ray experimental details for *t*-**5f** (CCDC 2313619).

Crystal data	
Chemical formula	$CF_3O_3S{\cdot}C_{13}H_{17}N_2$
M_r	350.36
Crystal system, space group	Triclinic, *P*–1
Temperature (K)	110
a, *b*, *c* (Å)	8.850(2), 9.284(2), 10.970(3)
α, β, γ (°)	107.639(7), 94.720(7), 109.337(6)
V (Å^3)	793.6(4)
Z	2
Radiation type	Mo *K*α
μ (mm^{-1})	0.25
Crystal size (mm)	0.30 × 0.20 × 0.15
Data collection	
Diffractometer	Bruker Quest (PHOTON III) diffractometer
Absorption correction	Multi-scan *SADABS2016*/2 (Bruker,2016/2) was used for absorption correction. wR2(int) was 0.0899 before and 0.0617 after correction. The Ratio of minimum to maximum transmission is 0.8476. The λ/2 correction factor is Not present.
T_{min}, T_{max}	0.632, 0.745
No. of measured, independent and found [$I \geqslant 2u(I)$] reflections	10252, 3019, 2070
R_{int}	0.072
$(\sin \theta/\lambda)_{max}$ (Å^{-1})	0.612
Refinement	
$R[F^2 > 2\sigma(F^2)]$, $wR(F^2)$, *S*	0.068, 0.143, 1.10
No. of reflections	3019
No. of parameters	208
H-atom treatment	H-atom parameters constrained
$\Delta\rho_{max}$, $\Delta\rho_{min}$ (e Å^{-3})	0.56, –0.74

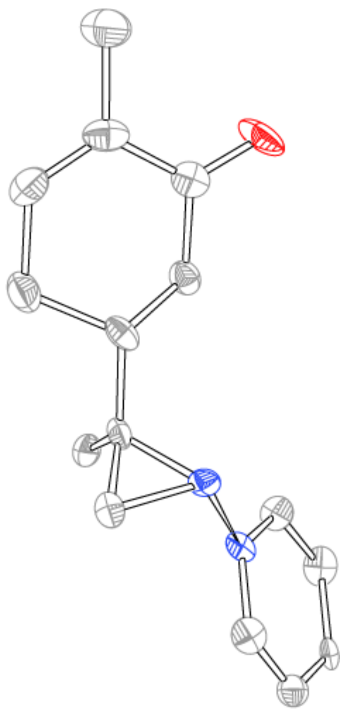

Figure S25. Thermal ellipsoid plot of **5aa** plotted at 50% probability. H-atoms and TfO^- counterion are removed for clarity. The crystalline sample used for the diffraction experiment was obtained via Et_2O diffusion to a CH_2Cl_2 solution at –20 °C.

Table S8. X-ray experimental details for **5aa** (CCDC 2314753).

Crystal data	
Chemical formula	$CF_3O_3S·CH_2Cl_2·C_{15}H_{19}N_2O$
M_r	477.32
Crystal system, space group	Monoclinic, *C*2
Temperature (K)	110
a, *b*, *c* (Å)	25.219(4), 6.996(1), 13.319(2)
β (°)	115.734(4)
V (Å³)	2116.9(6)
Z	4
Radiation type	Mo *K*α
μ (mm⁻¹)	0.46
Crystal size (mm)	0.3 × 0.2 × 0.2
Data collection	
Diffractometer	Bruker *APEX*-II CCD
Absorption correction	Multi-scan *SADABS2016*/2 (Bruker,2016/2) was used for absorption correction. wR2(int) was 0.1567 before and 0.0626 after correction. The Ratio of minimum to maximum transmission is 0.8536. The λ/2 correction factor is Not present.
T_{min}, T_{max}	0.636, 0.745
No. of measured, independent and found [$I > 2\sigma(I)$] reflections	13321, 3795, 3313
R_{int}	0.060
$(\sin \theta/\lambda)_{max}$ (Å⁻¹)	0.603
Refinement	
$R[F^2 > 2\sigma(F^2)]$, $wR(F^2)$, S	0.090, 0.180, 1.18
No. of reflections	3795
No. of parameters	264
No. of restraints	1
H-atom treatment	H-atom parameters constrained
	$w = 1/[\sigma^2(F_o^2) + (0.0472P)^2 + 13.0548P]$ where $P = (F_o^2 + 2F_c^2)/3$
$\Delta\rho_{max}$, $\Delta\rho_{min}$ (e Å⁻³)	0.46, −0.42
Absolute structure	Flack x determined using 1067 quotients [(I+)-(I-)]/[(I+)+(I-)] (Parsons, Flack and Wagner, Acta Cryst. B69 (2013) 249-259).
Absolute structure parameter	0.03(5)

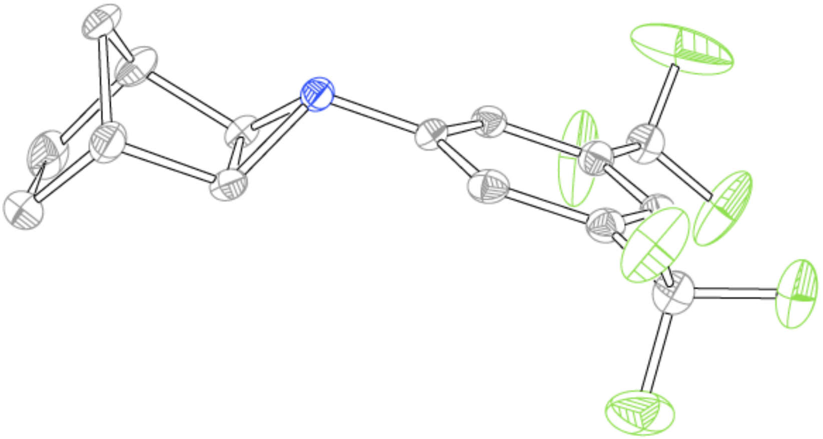

Figure S26. Thermal ellipsoid plot of **11c** plotted at 50% probability. H-atoms are removed for clarity. The crystalline sample used for the diffraction experiment was obtained via slow evaporation of a CH_2Cl_2 solution at –20 °C.

Table S9. X-ray experimental details for **11c** (CCDC 2314754).

Crystal data	
Chemical formula	$C_{15}H_{13}F_6N$
M_r	321.26
Crystal system, space group	Monoclinic, $P2_1/n$
Temperature (K)	100
a, b, c (Å)	8.6496(1), 11.4405(2), 13.9339(2)
β (°)	89.994(1)
V (Å^3)	1378.84(4)
Z	4
Radiation type	Cu $K\alpha$
μ (mm^{-1})	1.31
Crystal size (mm)	0.2 × 0.1 × 0.1
Data collection	
Diffractometer	XtaLAB Synergy, Dualflex, HyPix
Absorption correction	Multi-scan<br>*CrysAlis PRO* 1.171.42.101a (Rigaku Oxford Diffraction, 2023) Empirical absorption correction using spherical harmonics, implemented in SCALE3 ABSPACK scaling algorithm.
T_{min}, T_{max}	0.497, 1.000
No. of measured, independent and found [$I > 2\sigma(I)$] reflections	47832, 2962, 2880
R_{int}	0.077
$(\sin \theta/\lambda)_{max}$ (Å^{-1})	0.639
Refinement	
$R[F^2 > 2\sigma(F^2)]$, $wR(F^2)$, S	0.111, 0.245, 1.19
No. of reflections	2962
No. of parameters	199
H-atom treatment	H-atom parameters constrained
	$w = 1/[\sigma^2(F_o^2) + 13.6162P]$<br>where $P = (F_o^2 + 2F_c^2)/3$
$\Delta\rho_{max}$, $\Delta\rho_{min}$ (e Å^{-3})	0.74, −0.57

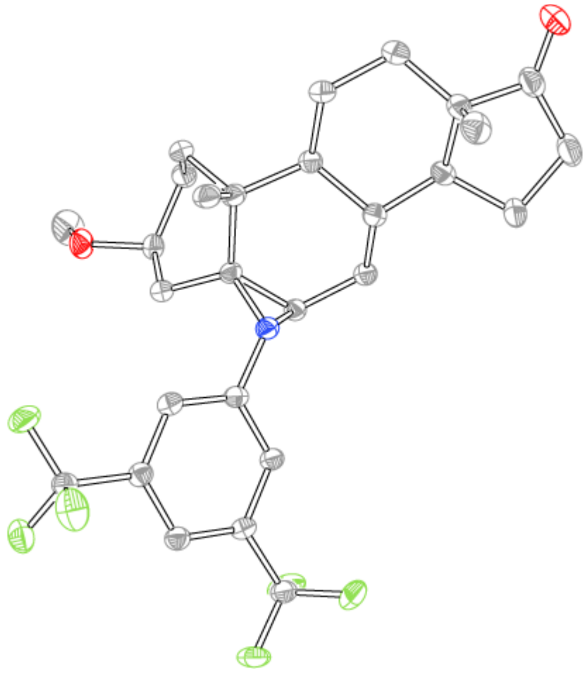

Figure S27. Thermal ellipsoid plot of **11f** plotted at 50% probability. H-atoms are removed for clarity. The crystalline sample used for the diffraction experiment was obtained via slow evaporation of a CH_2Cl_2 solution at –20 °C.

Table S10. X-ray experimental details for **11f** (CCDC 2314755).

Crystal data	
Chemical formula	$CH_2Cl_2 \cdot C_{28}H_{33}F_6NO_2$
M_r	614.48
Crystal system, space group	Monoclinic, $P2_1$
Temperature (K)	100
a, b, c (Å)	8.3042(1), 17.0509(3), 10.9991(2)
β (°)	110.024(2)
V (Å^3)	1463.26(4)
Z	2
Radiation type	Cu $K\alpha$
μ (mm^{-1})	2.58
Crystal size (mm)	0.2 × 0.2 × 0.1
Data collection	
Diffractometer	XtaLAB Synergy, Dualflex, HyPix
Absorption correction	Multi-scan<br>*CrysAlis PRO* 1.171.42.101a (Rigaku Oxford Diffraction, 2023) Empirical absorption correction using spherical harmonics, implemented in SCALE3 ABSPACK scaling algorithm.
T_{min}, T_{max}	0.586, 1.000
No. of measured, independent and found [$I > 2\sigma(I)$] reflections	31868, 6338, 6173
R_{int}	0.039
$(\sin \theta/\lambda)_{max}$ (Å^{-1})	0.639
Refinement	
$R[F^2 > 2\sigma(F^2)]$, $wR(F^2)$, S	0.036, 0.094, 1.07
No. of reflections	6338
No. of parameters	364
No. of restraints	1
H-atom treatment	H-atom parameters constrained
$\Delta\rho_{max}$, $\Delta\rho_{min}$ (e Å^{-3})	0.75, −0.79
Absolute structure	Flack x determined using 2871 quotients [(I+)-(I-)]/[(I+)+(I-)] (Parsons, Flack and Wagner, Acta Cryst. B69 (2013) 249-259).
Absolute structure parameter	0.036(6)

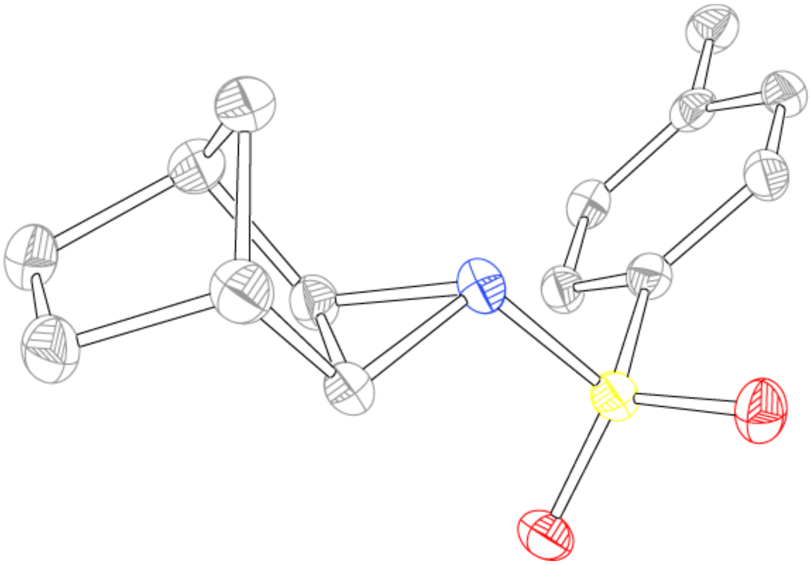

Figure S28. Thermal ellipsoid plot of **13** plotted at 50% probability. H-atoms are removed for clarity. The crystalline sample used for the diffraction experiment was obtained via slow evaporation of a $CHCl_3$ solution at 23 °C.

Table S11. X-ray experimental details for **13** (CCDC 2347027).

Crystal data	
Chemical formula	$C_{14}H_{17}NO_2S$
M_r	263.34
Crystal system, space group	Monoclinic, $P2_1/n$
Temperature (K)	110
a, b, c (Å)	6.0612(8), 18.291(3), 11.642(2)
β (°)	97.956(4)
V (Å^3)	1278.3(3)
Z	4
Radiation type	Mo $K\alpha$
μ (mm^{-1})	0.25
Crystal size (mm)	0.4 × 0.2 × 0.2
Data collection	
Diffractometer	Bruker *APEX*-II CCD
Absorption correction	Multi-scan *SADABS2016*/2 (Bruker,2016/2) was used for absorption correction. wR2(int) was 0.1323 before and 0.0634 after correction. The Ratio of minimum to maximum transmission is 0.9136. The λ/2 correction factor is Not present.
T_{min}, T_{max}	0.681, 0.745
No. of measured, independent and observed [$I > 2\sigma(I)$] reflections	36925, 2648, 2144
R_{int}	0.060
$(\sin \theta/\lambda)_{max}$ (Å^{-1})	0.627
Refinement	
$R[F^2 > 2\sigma(F^2)]$, $wR(F^2)$, S	0.039, 0.104, 1.10
No. of reflections	2648
No. of parameters	164
H-atom treatment	H-atom parameters constrained
$\Delta\rho_{max}$, $\Delta\rho_{min}$ (e Å^{-3})	0.30, −0.44

H. NMR Spectra for New Compounds

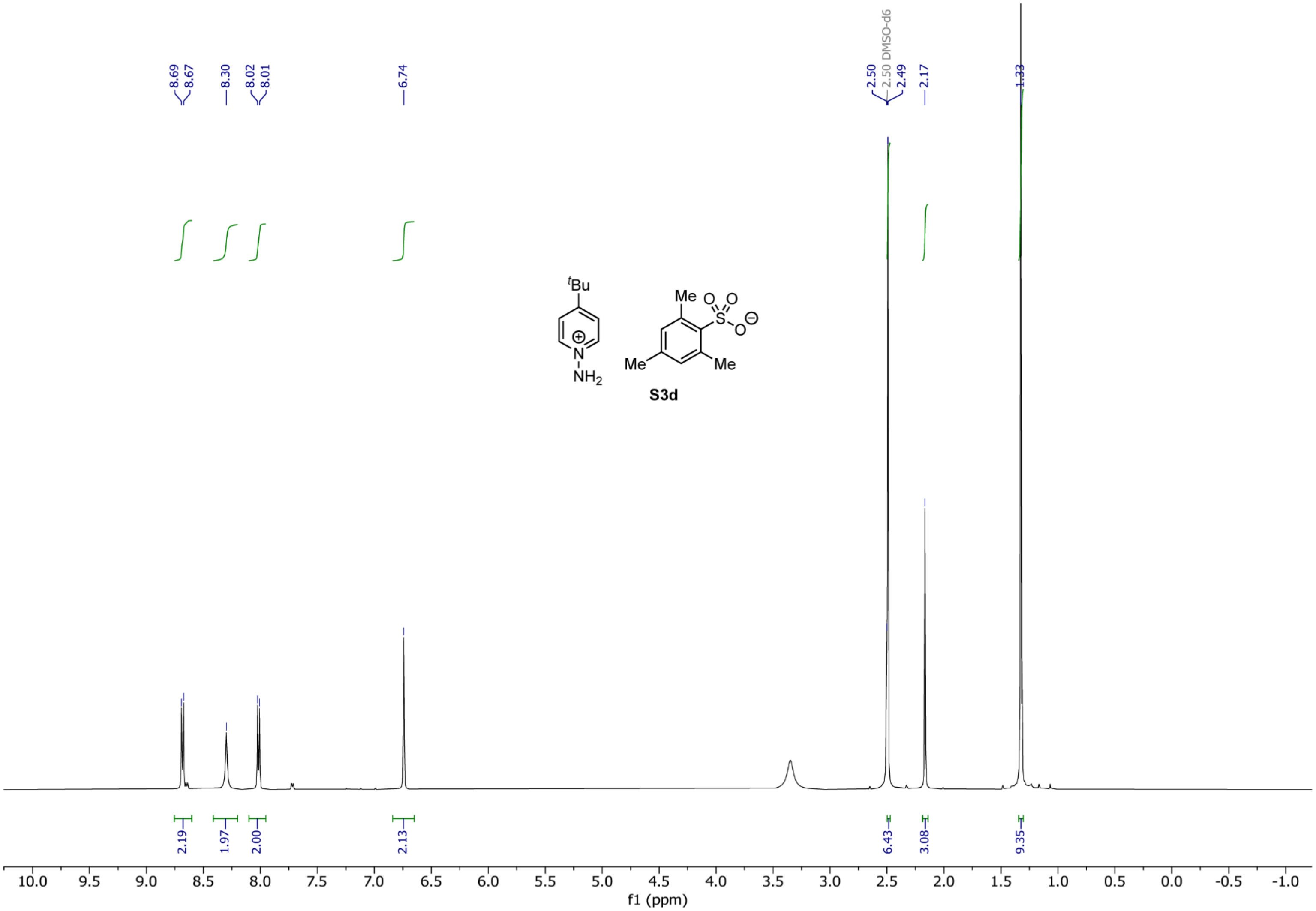

Figure S29. ^{1}H NMR spectrum of 1-amino-4-(*tert*-butyl)pyridin-1-ium 2,4,6-trimethylbenzenesulfonate (**S3d**) in DMSO (400 MHz) at 23 °C.

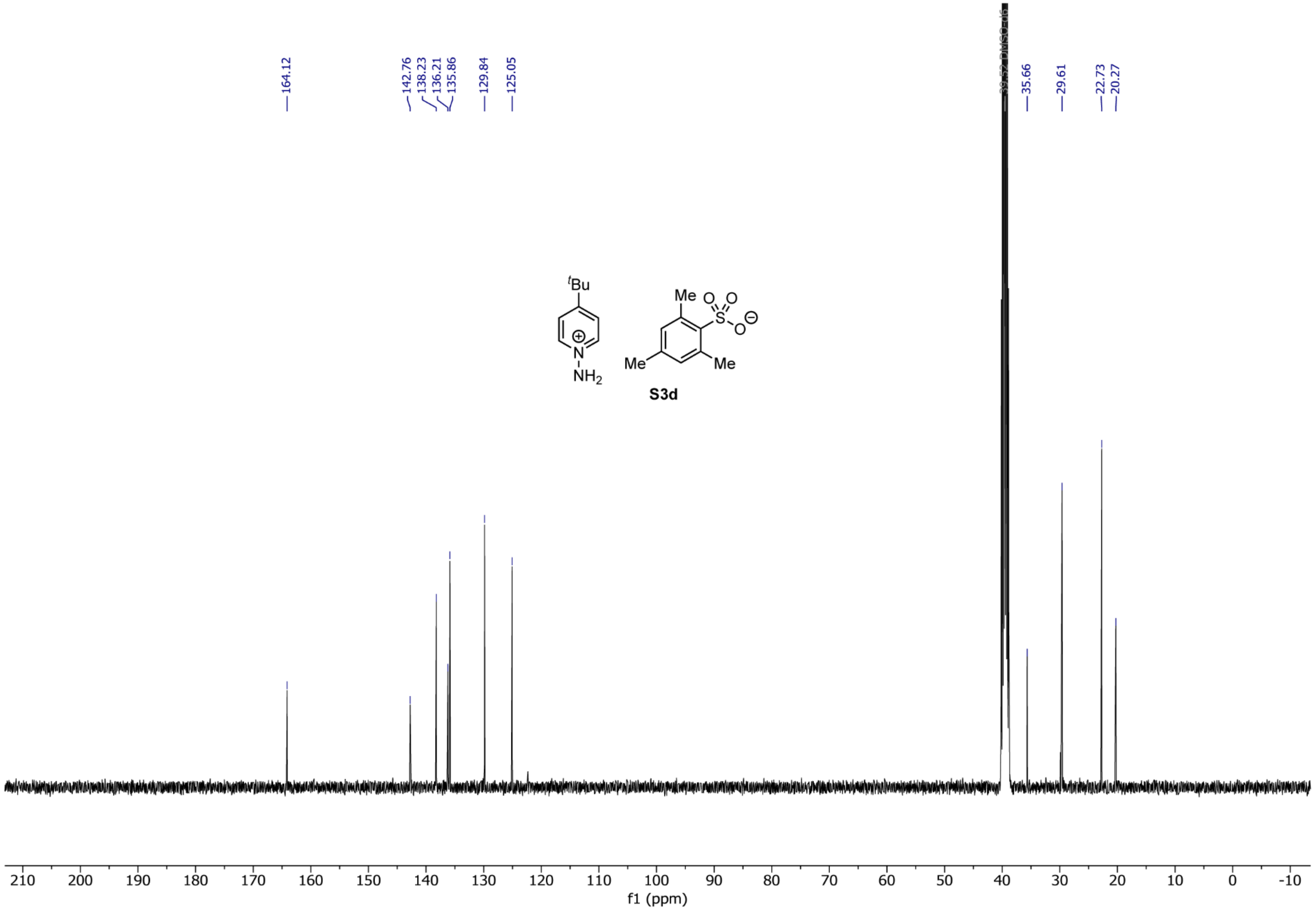

Figure S30. ^{13}C NMR spectrum of 1-amino-4-(*tert*-butyl)pyridin-1-ium 2,4,6-trimethylbenzenesulfonate (**S3d**) in DMSO (101 MHz) at 23 °C.

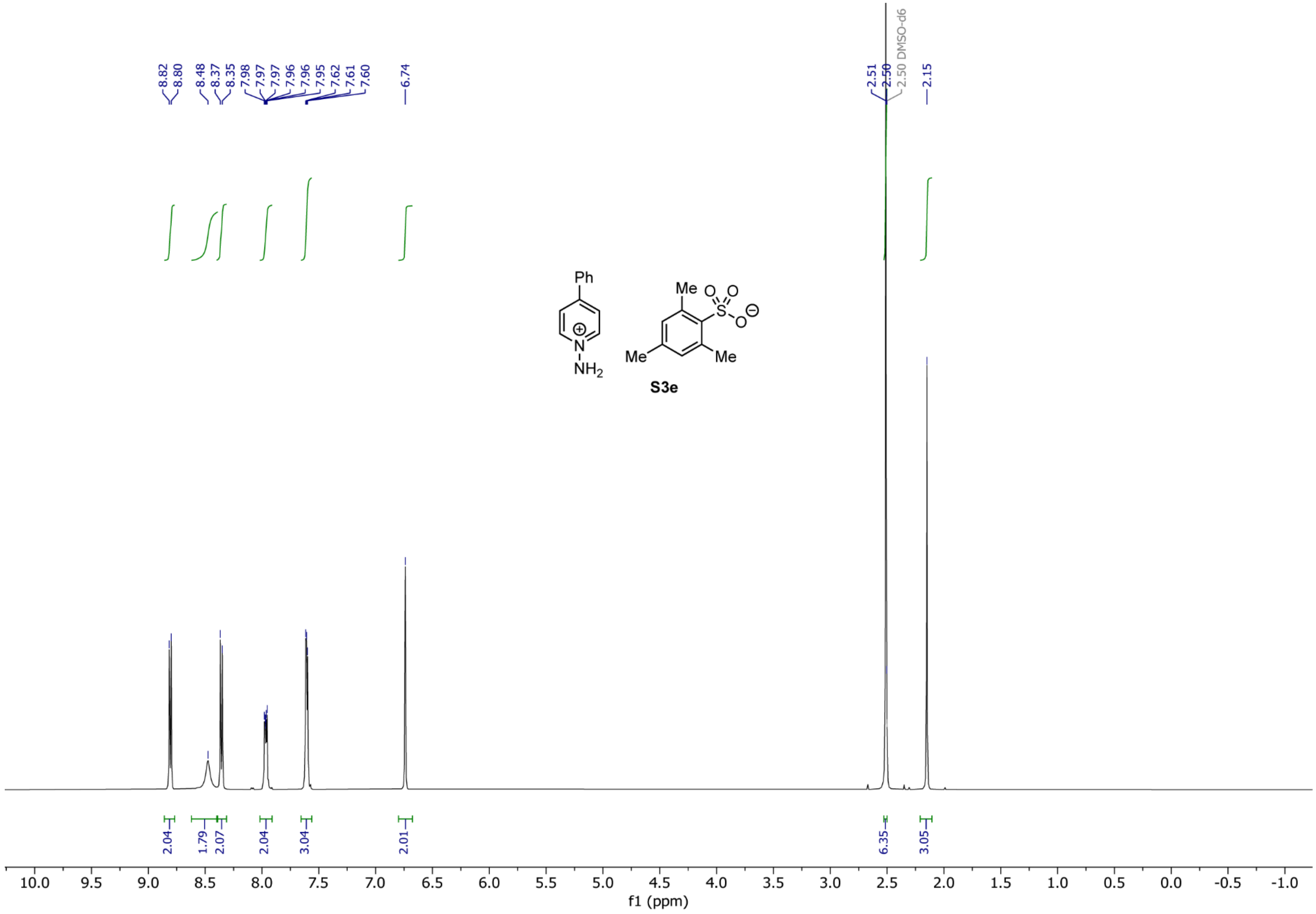

Figure S31. ^{1}H NMR spectrum of 1-amino-4-phenylpyridin-1-ium 2,4,6-trimethylbenzenesulfonate (**S3e**) in DMSO (400 MHz) at 23 °C.

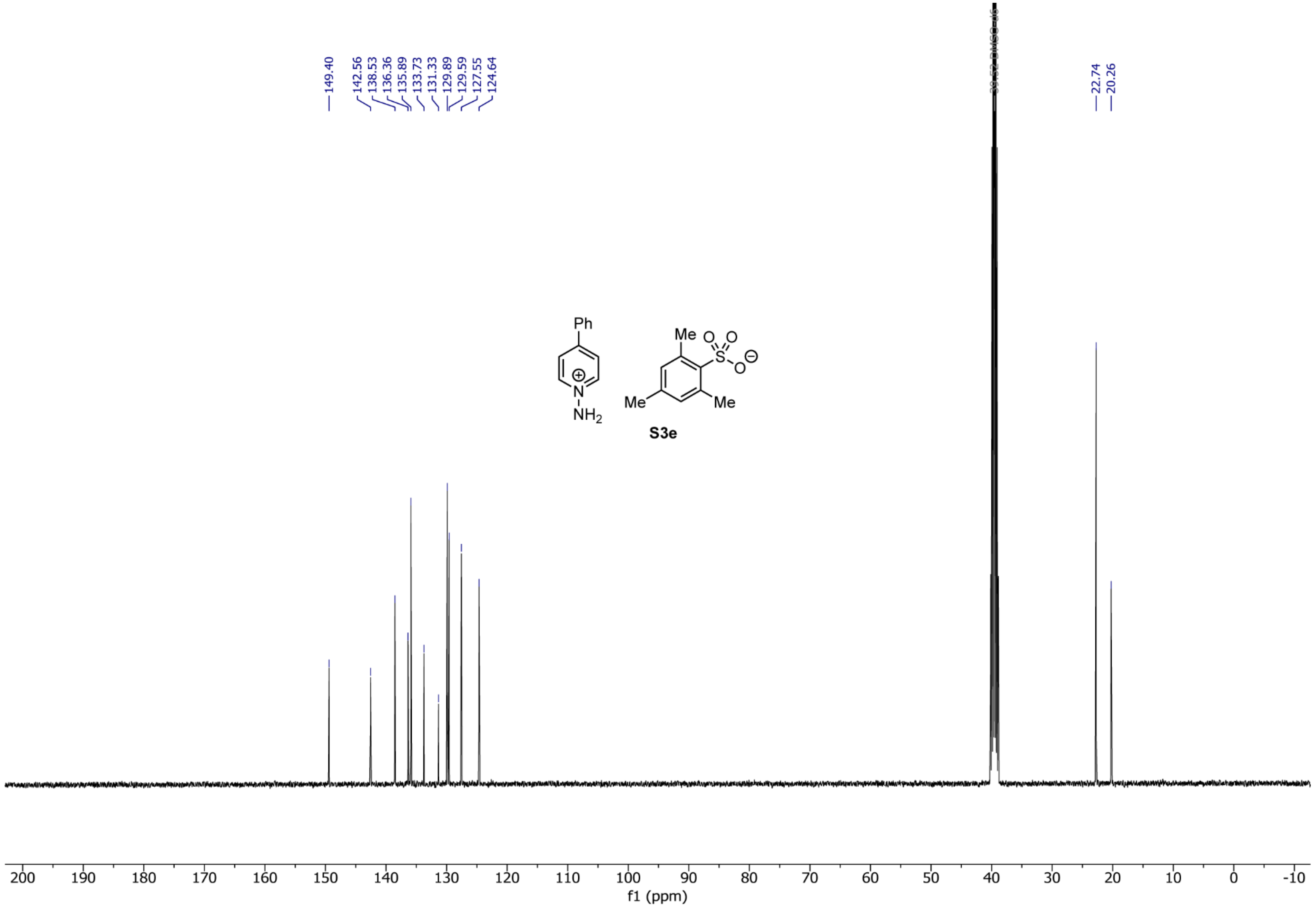

Figure S32. ^{13}C NMR spectrum of 1-amino-4-phenylpyridin-1-ium 2,4,6-trimethylbenzenesulfonate (**S3e**) in DMSO (101 MHz) at 23 °C.

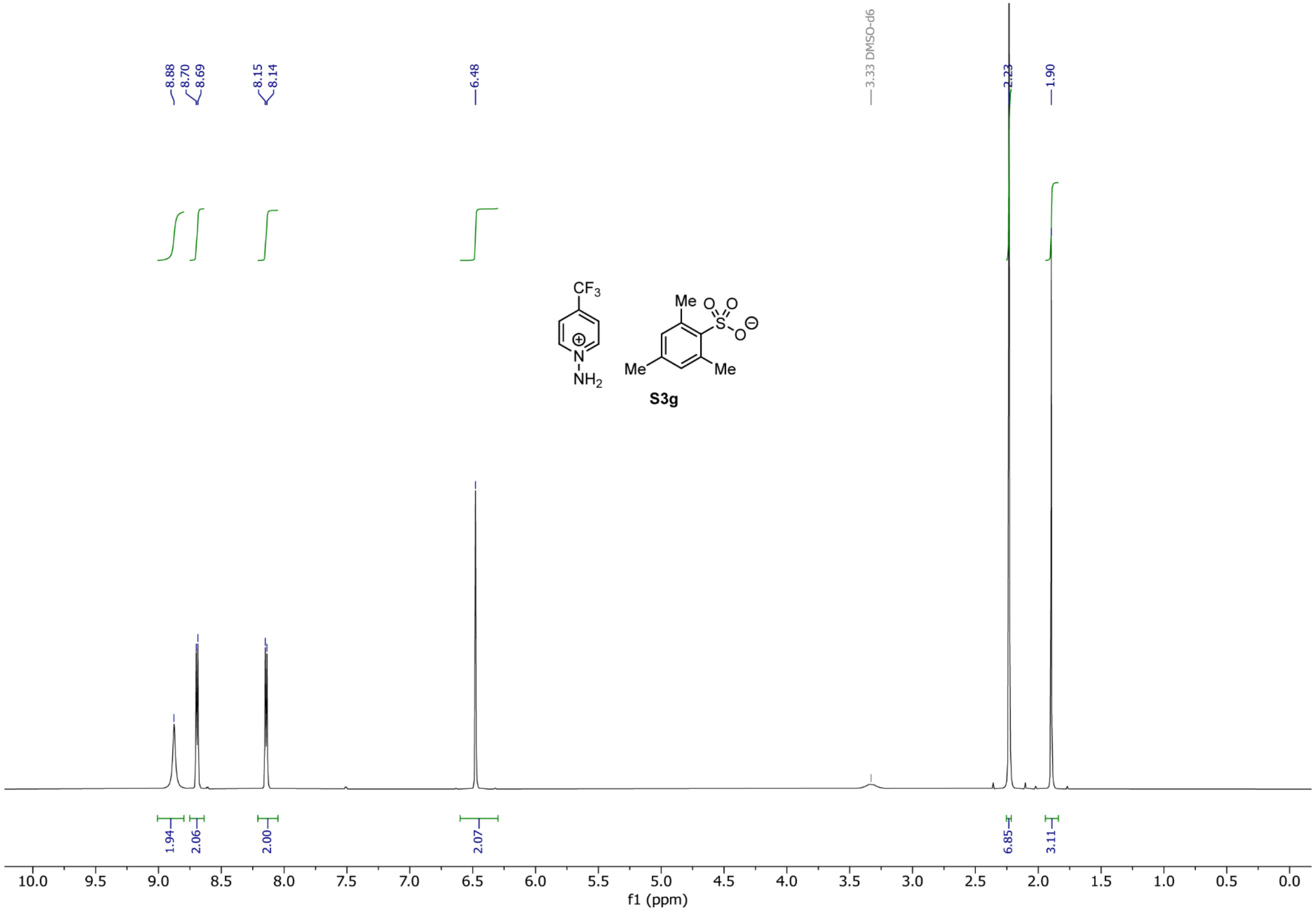

Figure S33. ^{1}H NMR spectrum of 1-amino-4-(trifluoromethyl)pyridin-1-ium 2,4,6-trimethylbenzenesulfonate (**S3g**) in DMSO (500 MHz) at 23 °C.

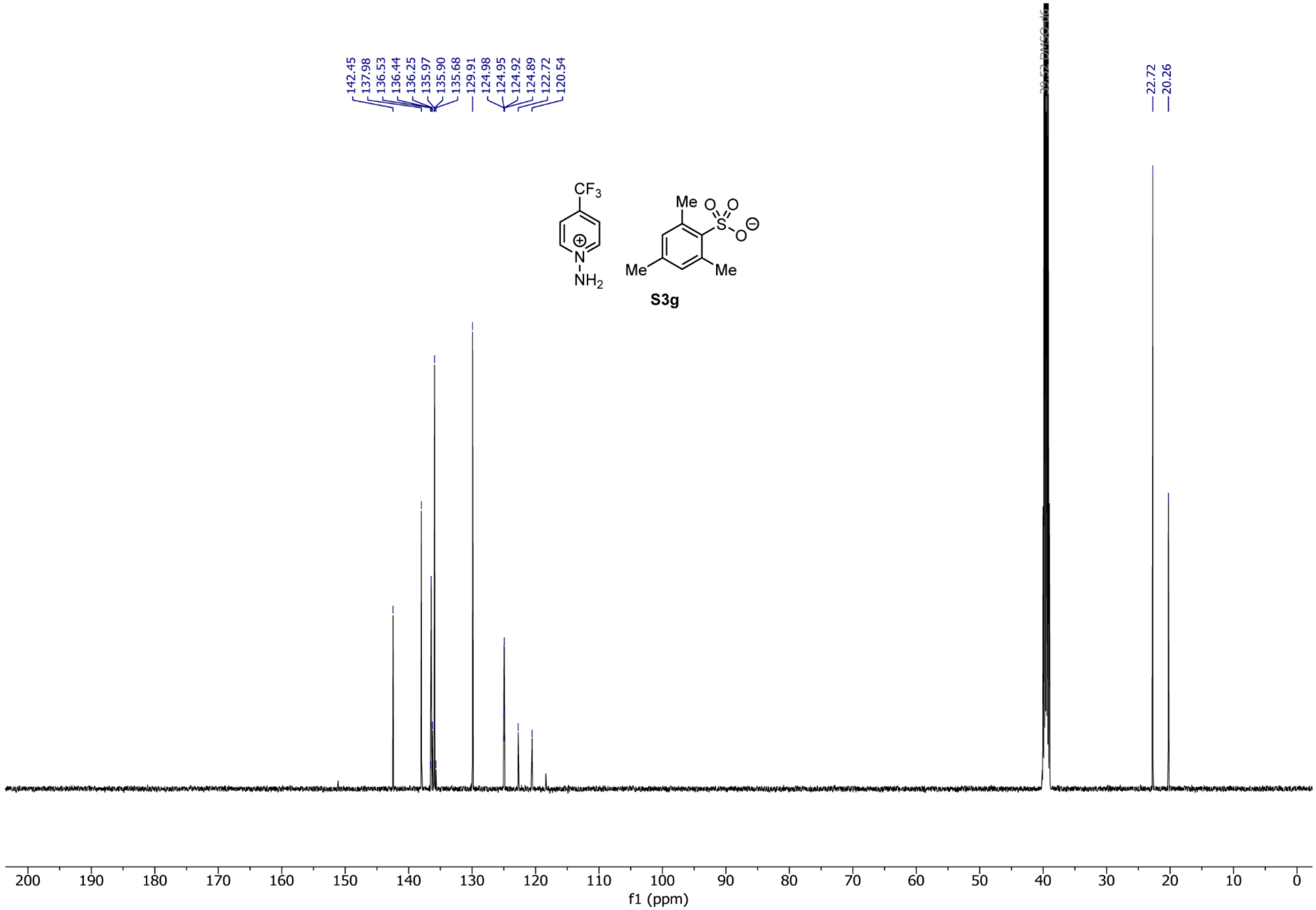

Figure S34. ^{13}C NMR spectrum of 1-amino-4-(trifluoromethyl)pyridin-1-ium 2,4,6-trimethylbenzenesulfonate (**S3g**) in DMSO (126 MHz) at 23 °C.

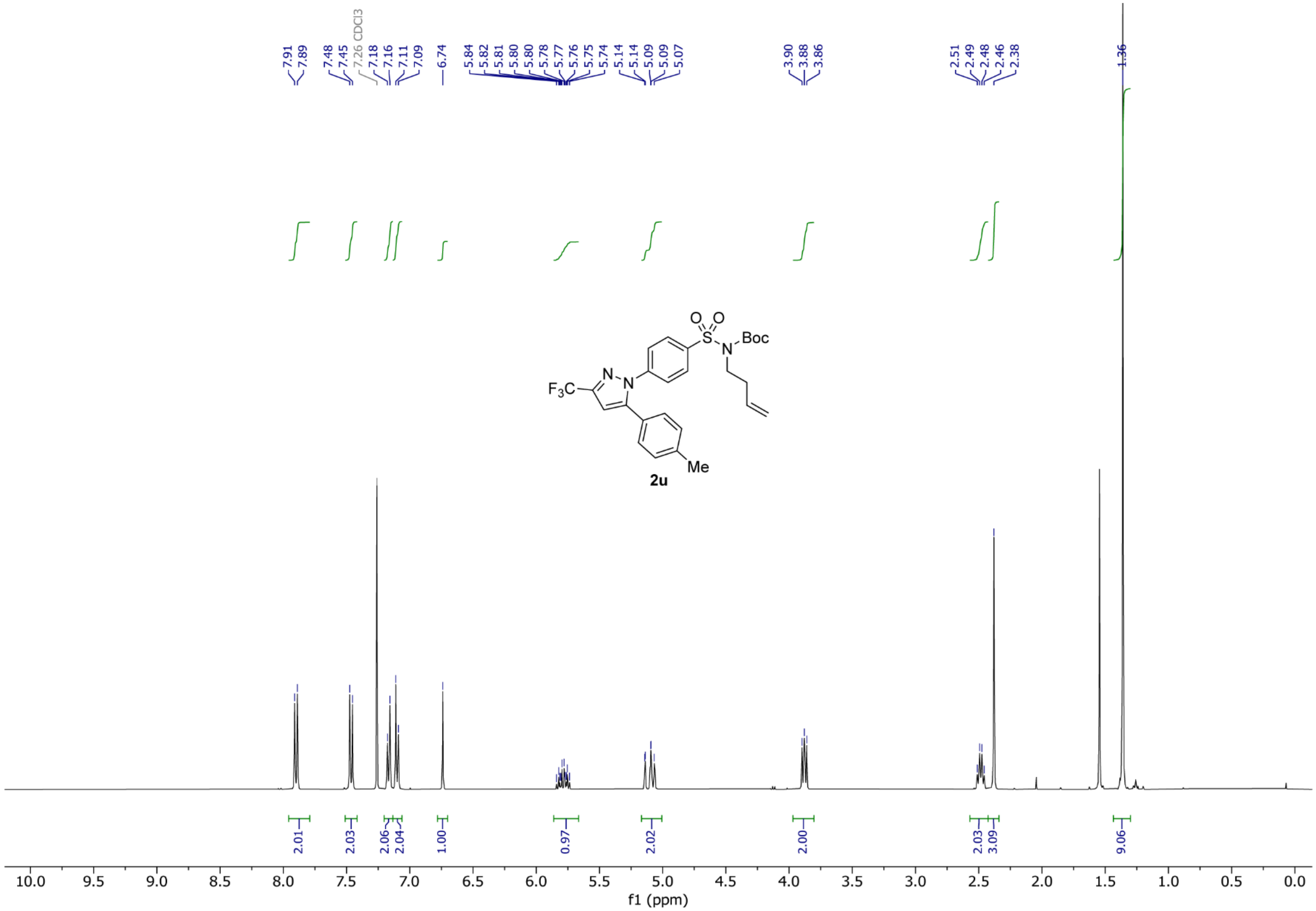

Figure S35. ^{1}H NMR spectrum of *tert*-butyl but-3-en-1-yl((4-(5-(*p*-tolyl)-3-(trifluoromethyl)-1*H*-pyrazol-1-yl)phenyl)sulfonyl) carbamate (**2u**) in $CDCl_3$ (400 MHz) at 23 °C.

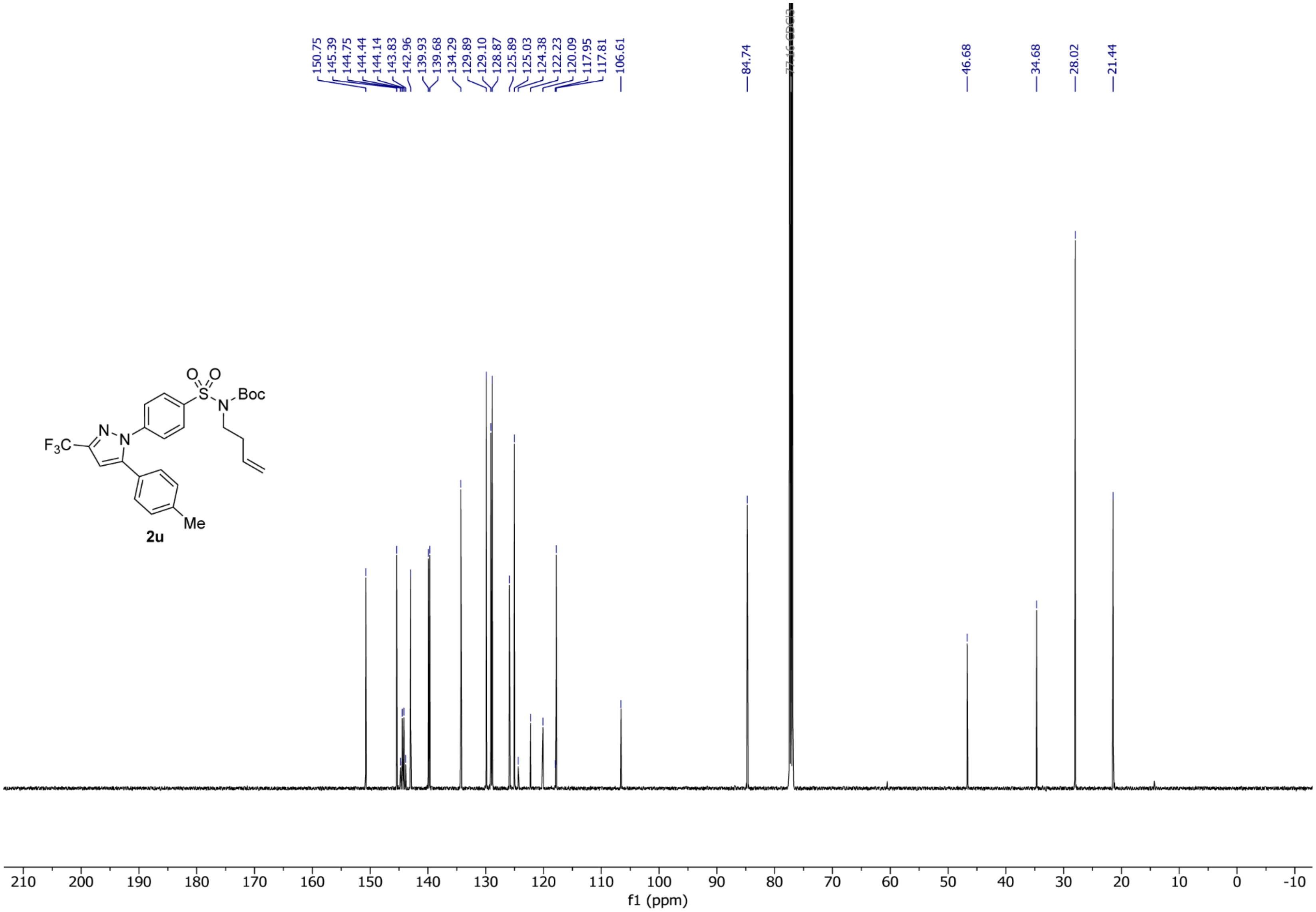

Figure S36. ^{13}C NMR spectrum of *tert*-butyl but-3-en-1-yl((4-(5-(*p*-tolyl)-3-(trifluoromethyl)-1*H*-pyrazol-1-yl)phenyl)sulfonyl) carbamate (**2u**) in $CDCl_3$ (126 MHz) at 23 °C.

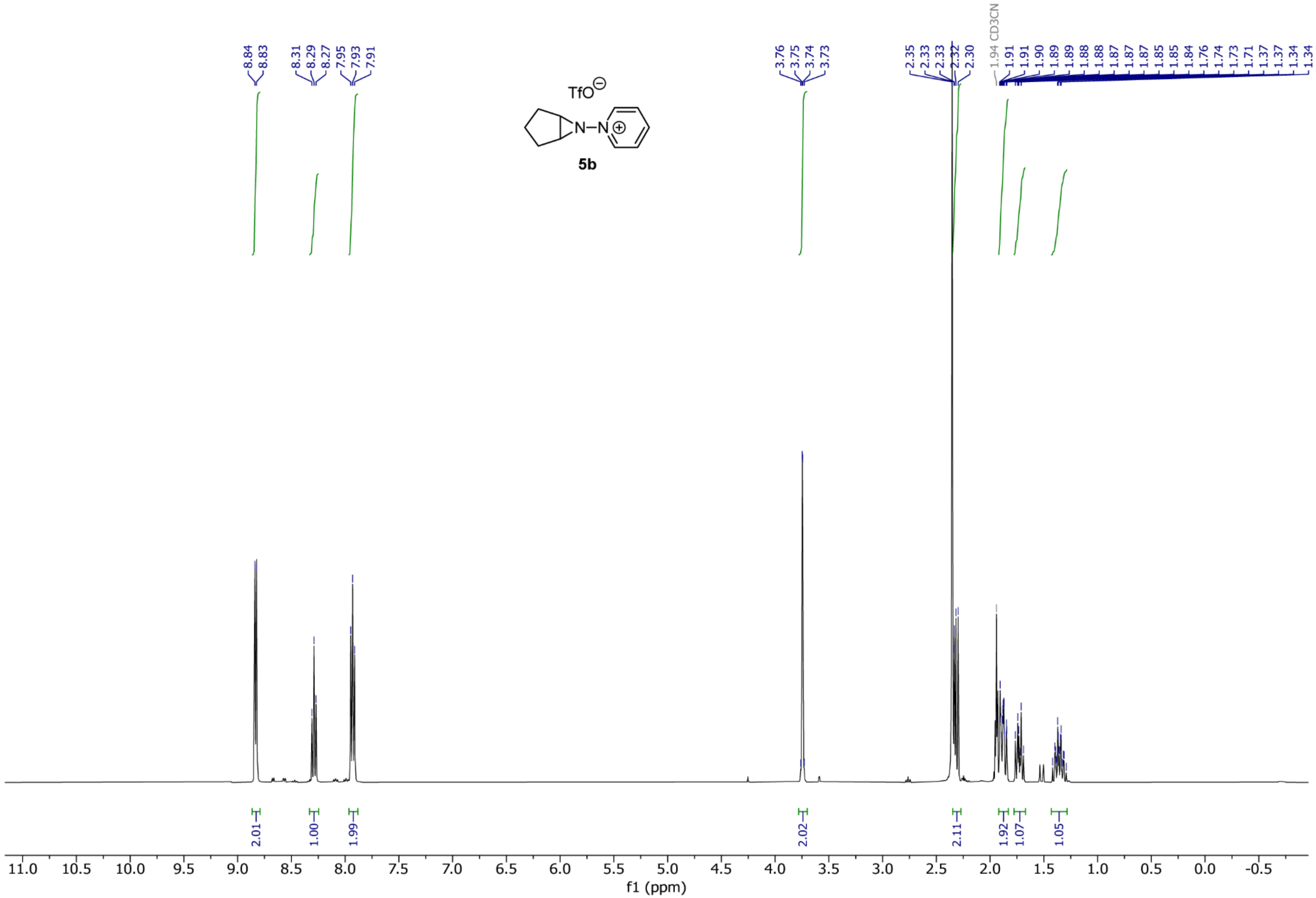

Figure S37. ^{1}H NMR spectrum of 1-(6-azabicyclo[3.1.0]hexan-6-yl)pyridin-1-ium trifluoromethanesulfonate (**5b**) in CD_3CN (400 MHz) at 23 °C.

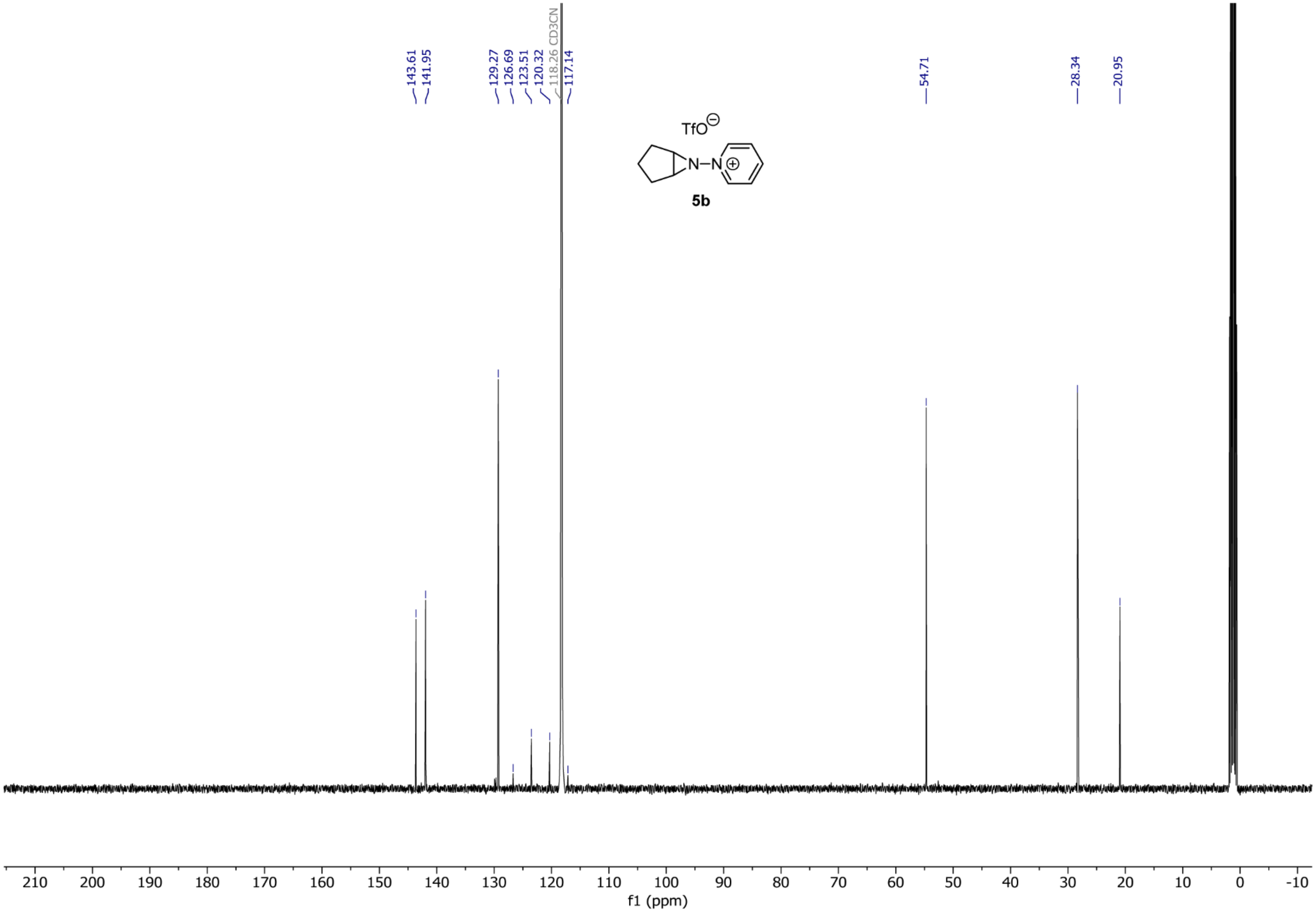

Figure S38. ^{13}C NMR spectrum of 1-(6-azabicyclo[3.1.0]hexan-6-yl)pyridin-1-ium trifluoromethanesulfonate (**5b**) in CD_3CN (101 MHz) at 23 °C.

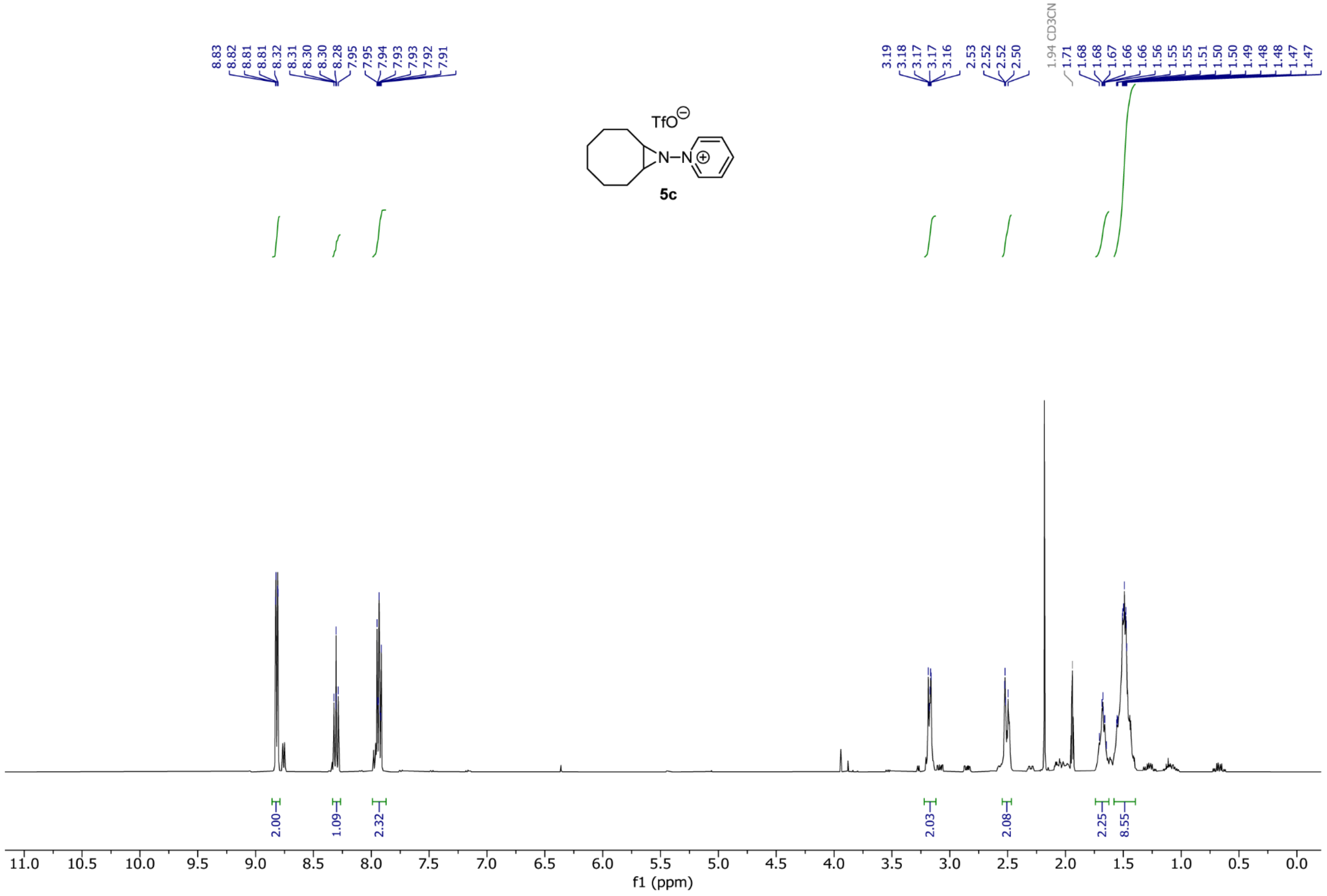

Figure S39. ^{1}H NMR spectrum of 1-(9-azabicyclo[6.1.0]nonan-9-yl)pyridine-1-ium trifluoromethanesulfonate (**5c**) in CD_3CN (400 MHz) at 23 °C. Extra peaks are attributed to isomer *t*-**5c**.

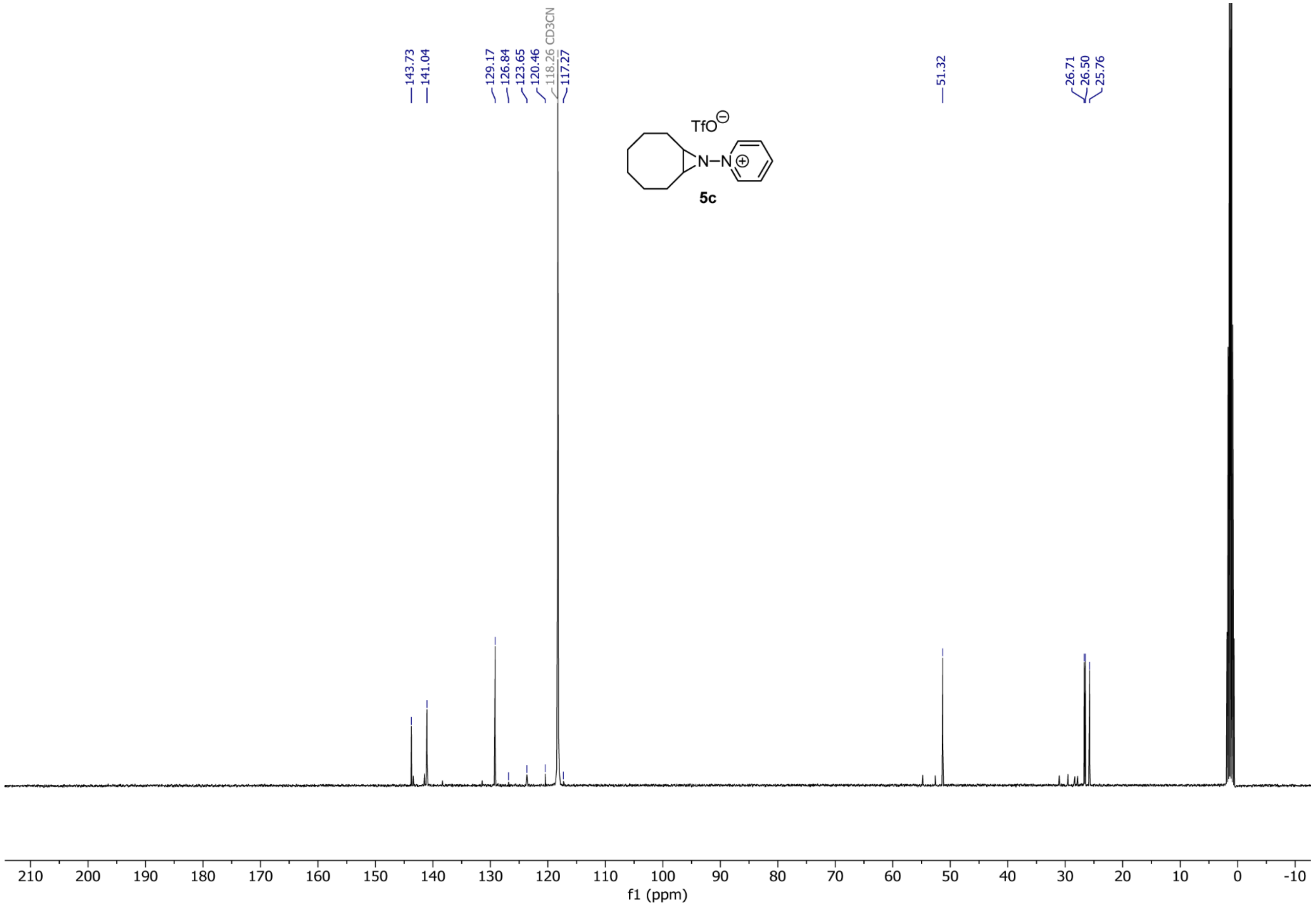

Figure S40. ${}^{13}C$ NMR spectrum of 1-(9-azabicyclo[6.1.0]nonan-9-yl)pyridine-1-ium trifluoromethanesulfonate (**5c**) in CD_3CN (101 MHz) at 23 °C.

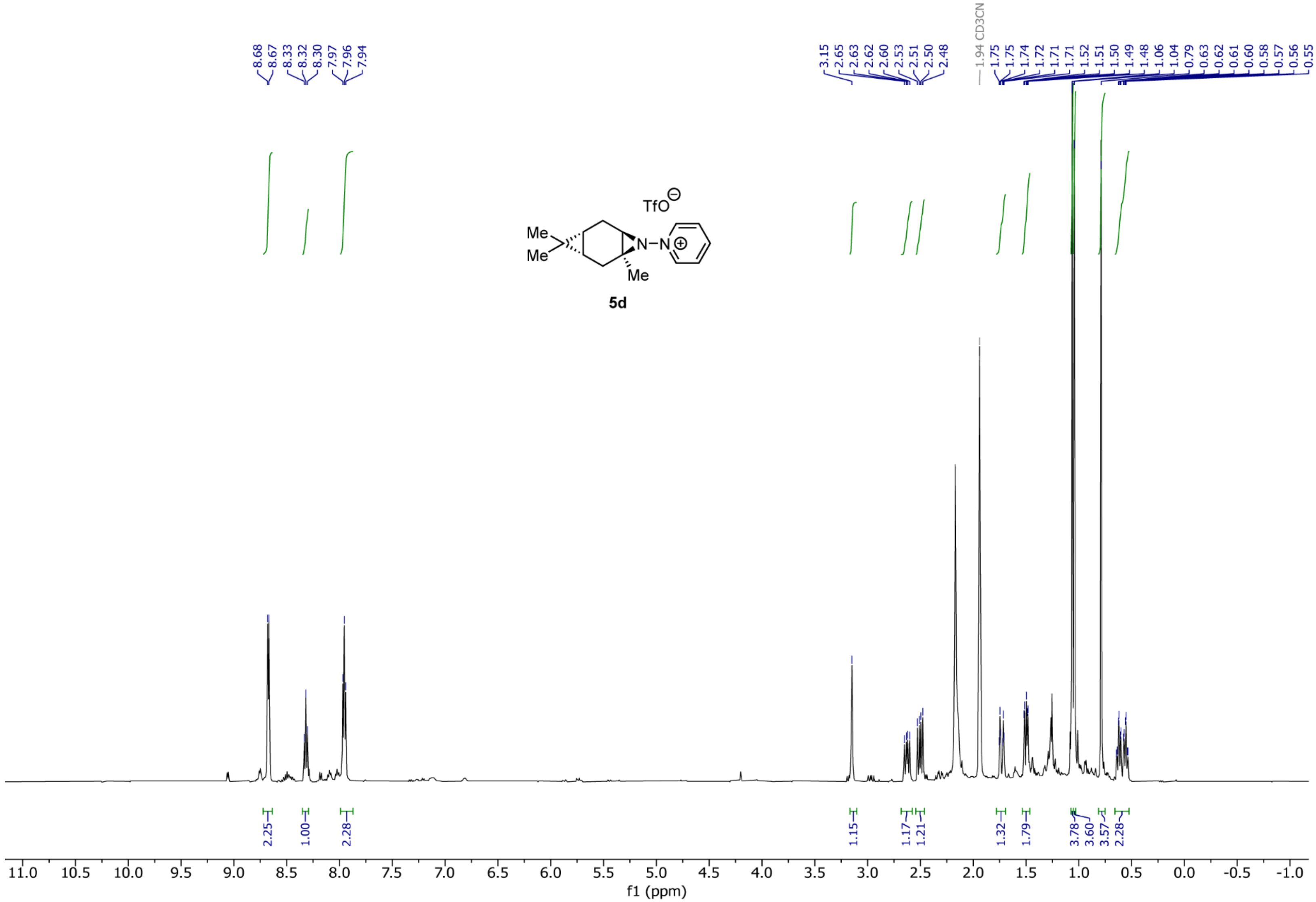

Figure S41. ^{1}H NMR spectrum of 1-((1*S*,3*S*,5*R*,7*R*)-3,8,8-trimethyl-4-azatricyclo[5.1.0.0^{3,5}]octan-4-yl)pyridin-1-ium trifluoromethanesulfonate (**5d**) in CD_3CN (500 MHz) at 23 °C.

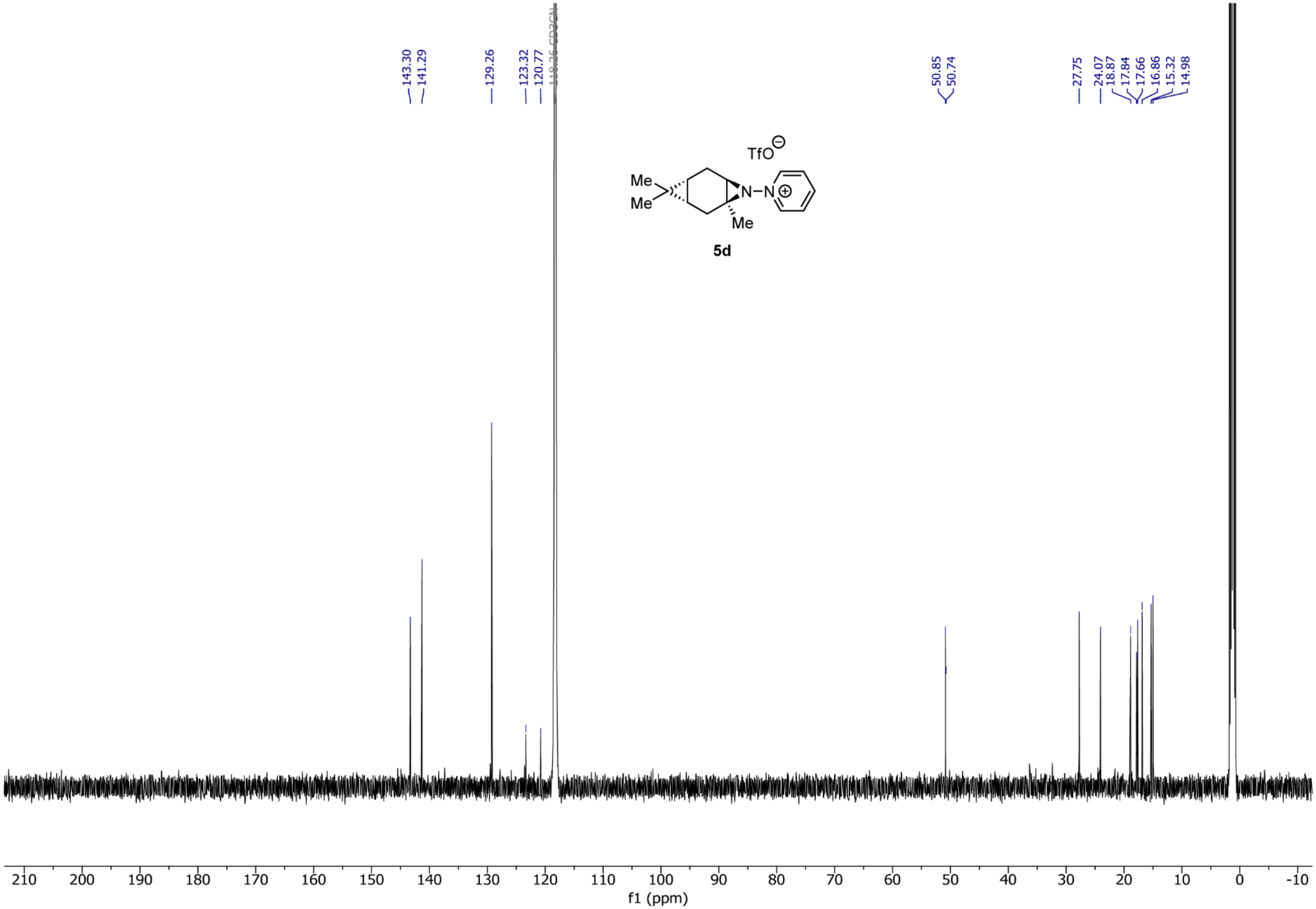

Figure S42. ^{13}C NMR spectrum of 1-((1*S*,3*S*,5*R*,7*R*)-3,8,8-trimethyl-4-azatricyclo[5.1.0.0^{3,5}]octan-4-yl)pyridin-1-ium trifluoromethanesulfonate (**5d**) in CD_3CN (126 MHz) at 23 °C.

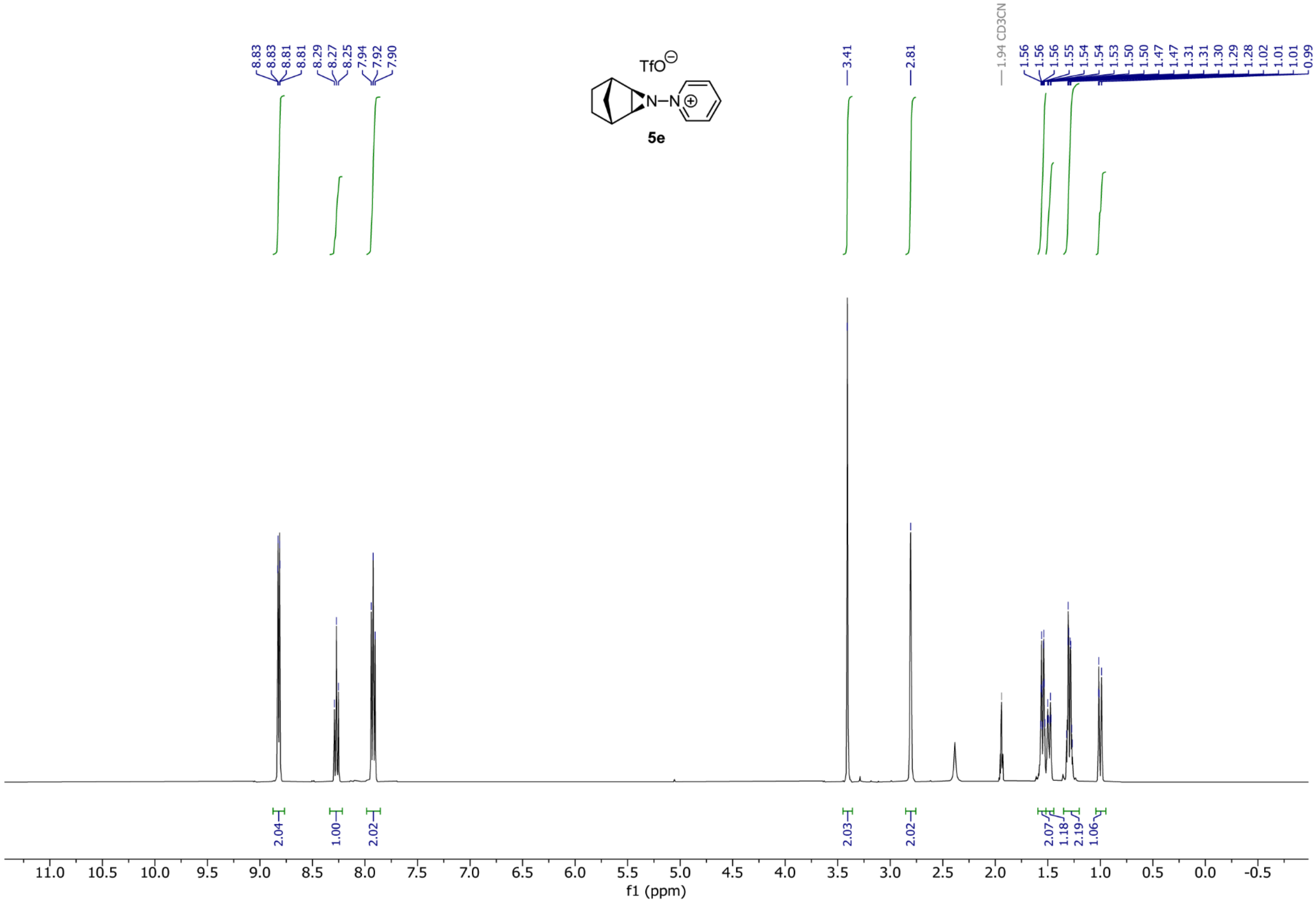

Figure S43. ^{1}H NMR spectrum of 1-(3-azatricyclo[3.2.1.0^{2,4}]octan-3-yl)pyridine-1-ium trifluoromethanesulfonate (**5e**) in CD_3CN (400 MHz) at 23 °C.

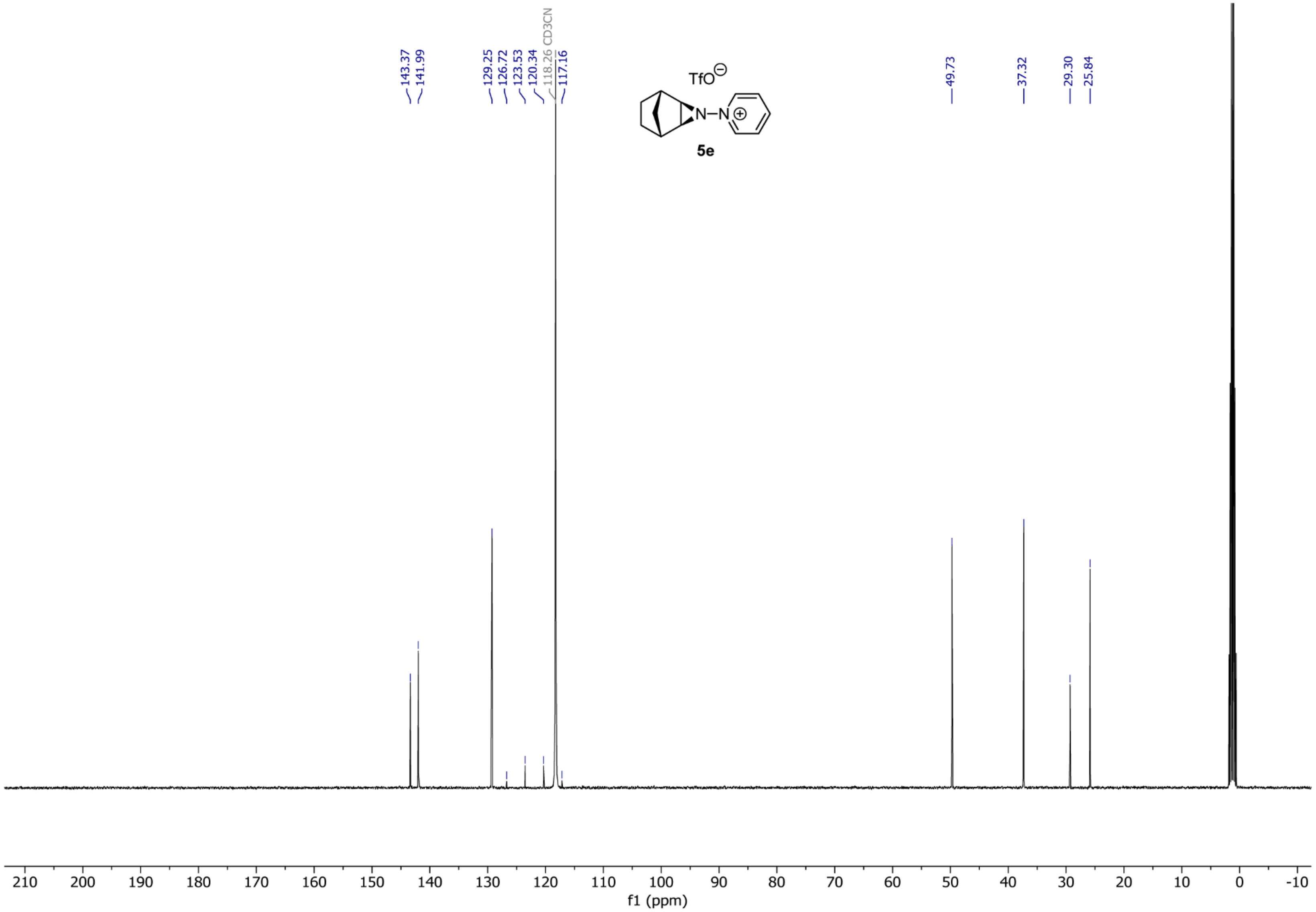

Figure S44. ^{13}C NMR spectrum of 1-(3-azatricyclo[3.2.1.0^{2,4}]octan-3-yl)pyridin-1-ium trifluoromethanesulfonate (**5e**) in CD_3CN (101 MHz) at 23 °C.

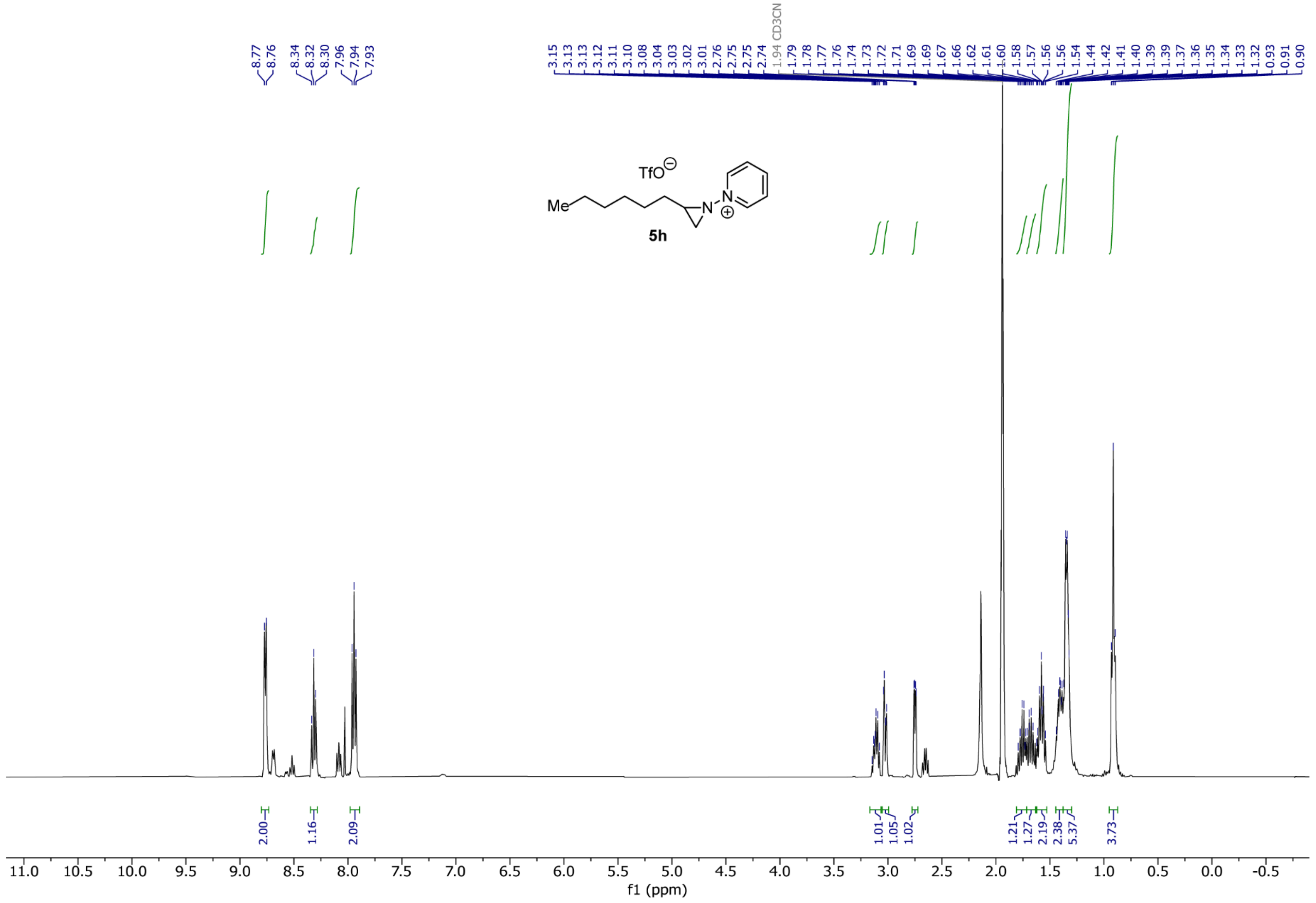

Figure S45. ^{1}H NMR spectrum of 1-(2-hexylaziridin-1-yl)pyridin-1-ium trifluoromethanesulfonate (**5h**) in CD_3CN (400 MHz) at 23 °C (extra peaks attributed to the imine byproduct which co-elutes with **5h**).

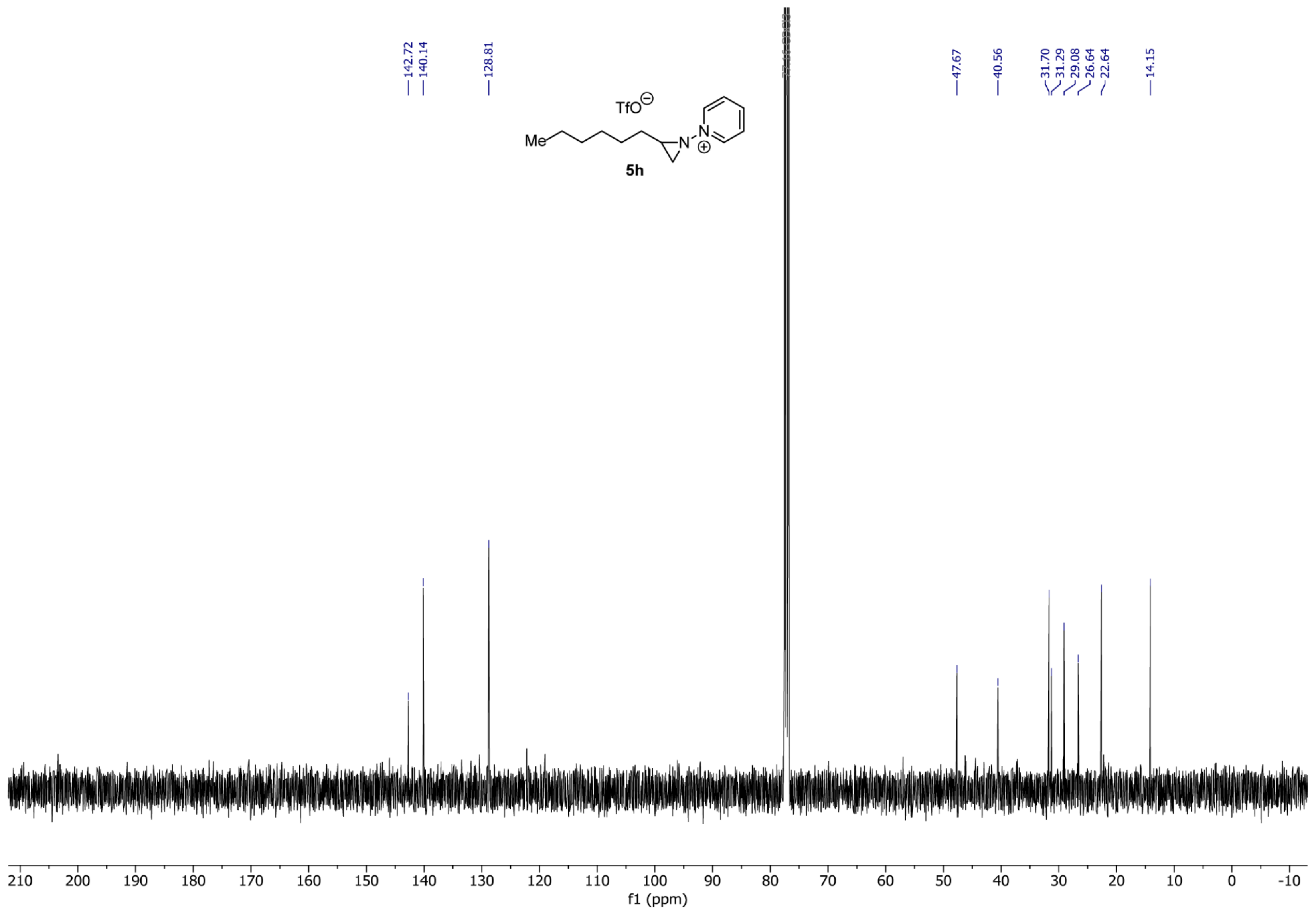

Figure S46. ^{13}C NMR spectrum of 1-(2-hexylaziridin-1-yl)pyridin-1-ium trifluoromethanesulfonate (**5h**) in CD_3CN (101 MHz) at 23 °C.

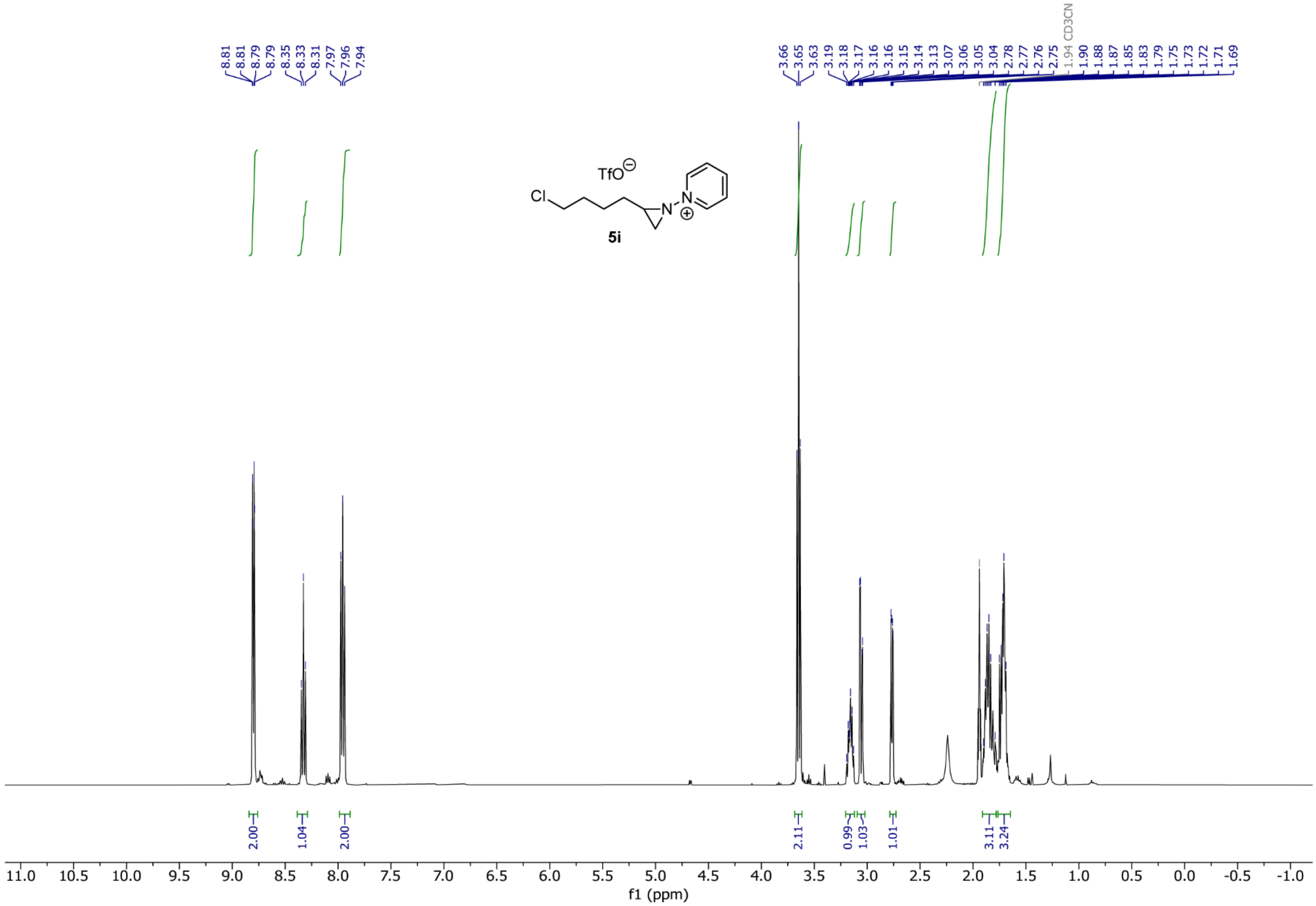

Figure S47. ^{1}H NMR spectrum of 1-(2-(4-chlorobutyl)aziridin-1-yl)pyridin-1-ium trifluoromethanesulfonate (**5i**) in CD_3CN (400 MHz) at 23 °C.

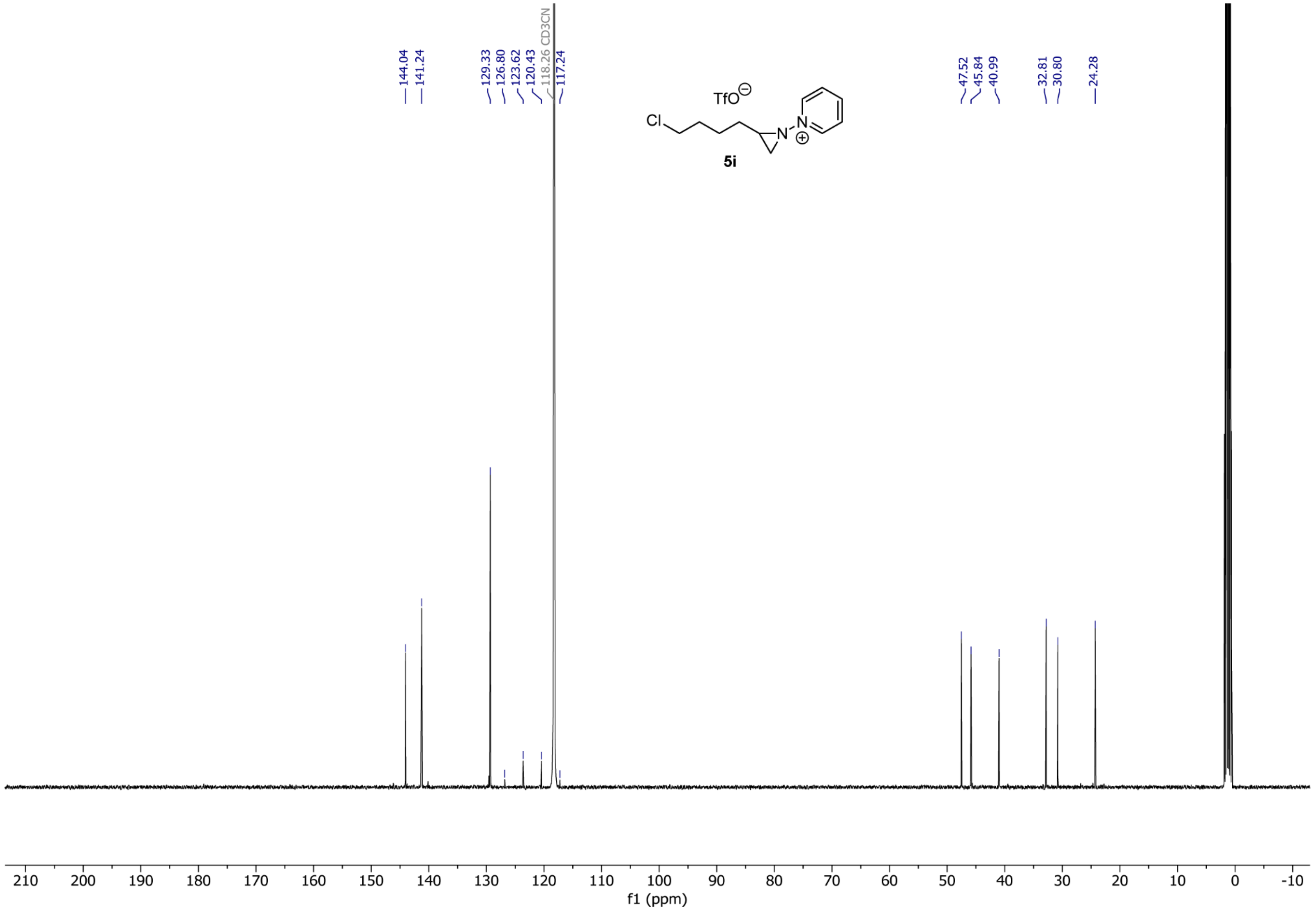

Figure S48. ^{13}C NMR spectrum of 1-(2-(4-chlorobutyl)aziridin-1-yl)pyridin-1-ium trifluoromethanesulfonate (**5i**) in CD_3CN (101 MHz) at 23 °C.

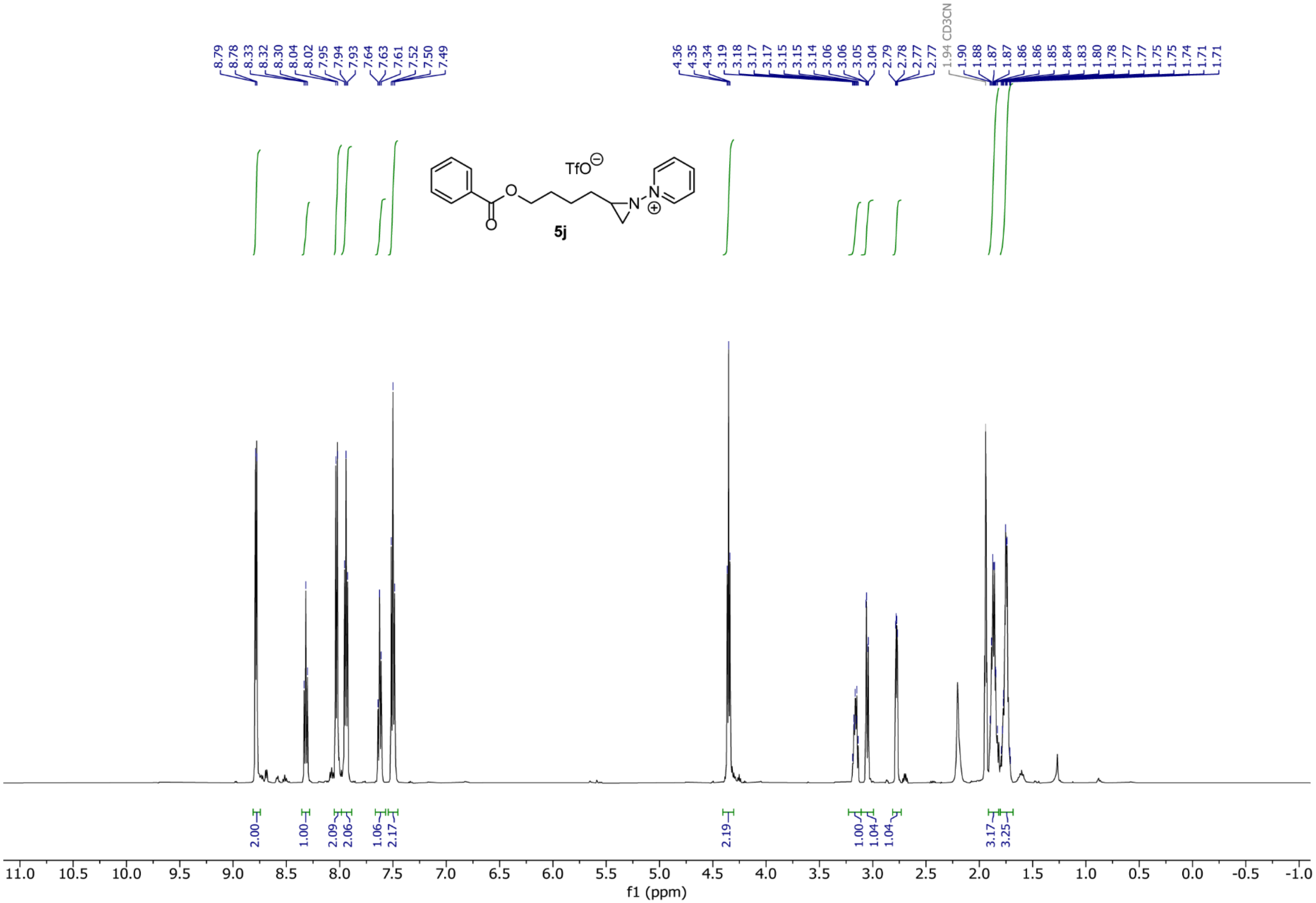

Figure S49. ^{1}H NMR spectrum of 1-(2-(4-(benzoyloxy)butyl)aziridin-1-yl)pyridin-1-ium trifluoromethanesulfonate (**5j**) in CD_3CN (500 MHz) at 23 °C.

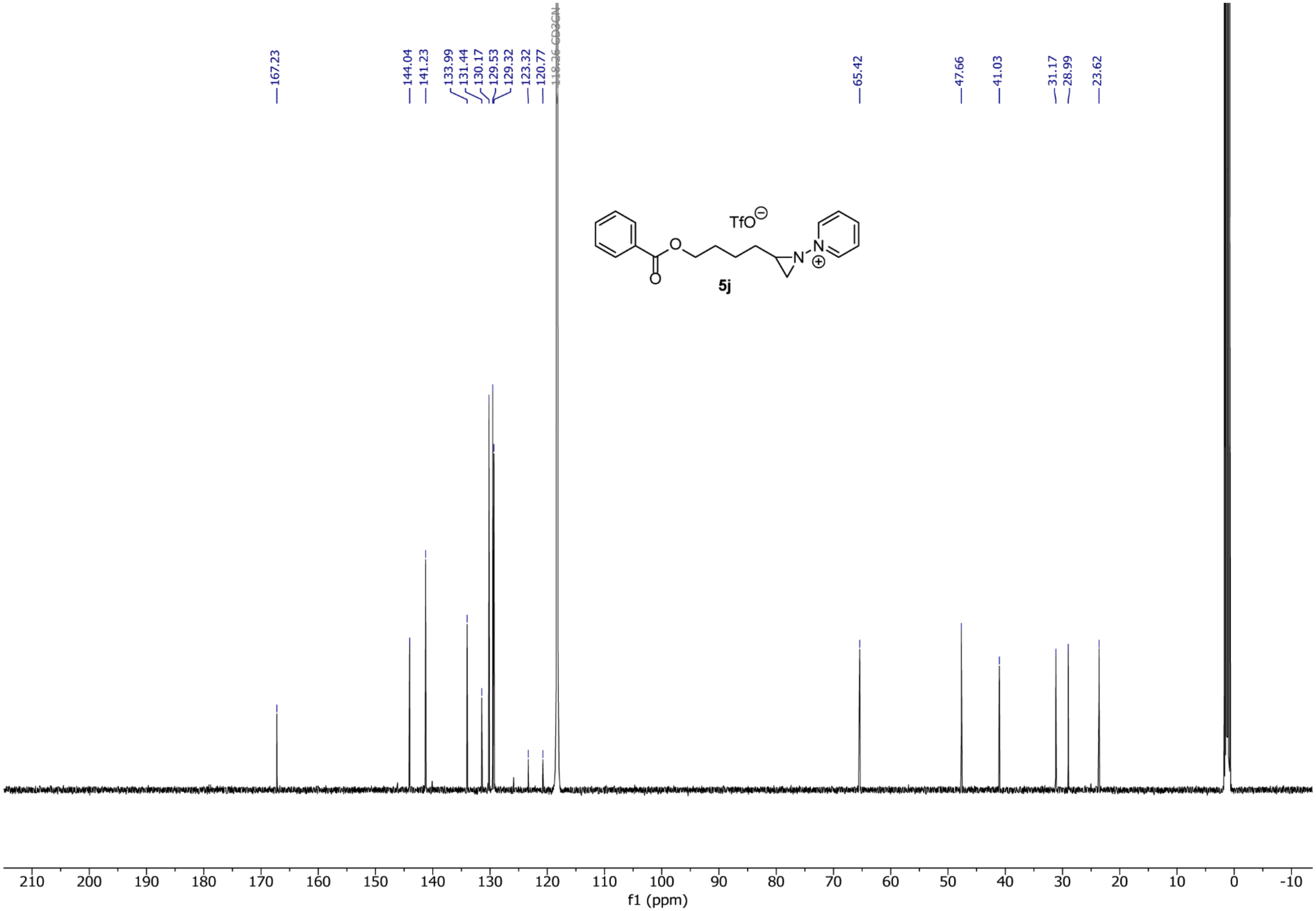

Figure S50. ^{13}C NMR spectrum of 1-(2-(4-(benzoyloxy)butyl)aziridin-1-yl)pyridin-1-ium trifluoromethanesulfonate (**5j**) in CD_3CN (126 MHz) at 23 °C.

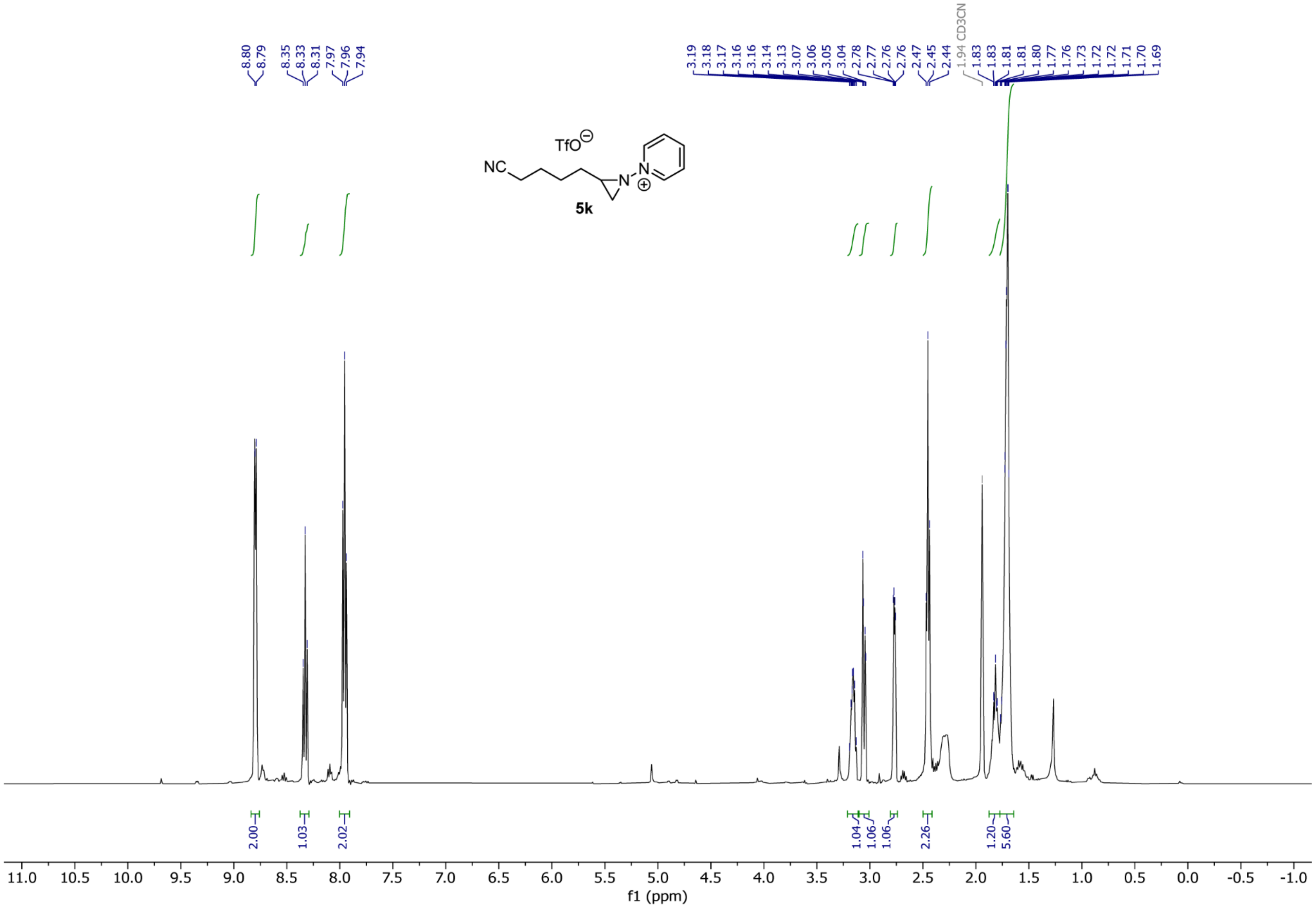

Figure S51. ^{1}H NMR spectrum of 1-(2-(4-cyanobutyl)aziridin-1-yl)pyridin-1-ium trifluoromethanesulfonate (**5k**) in CD_3CN (400 MHz) at 23 °C.

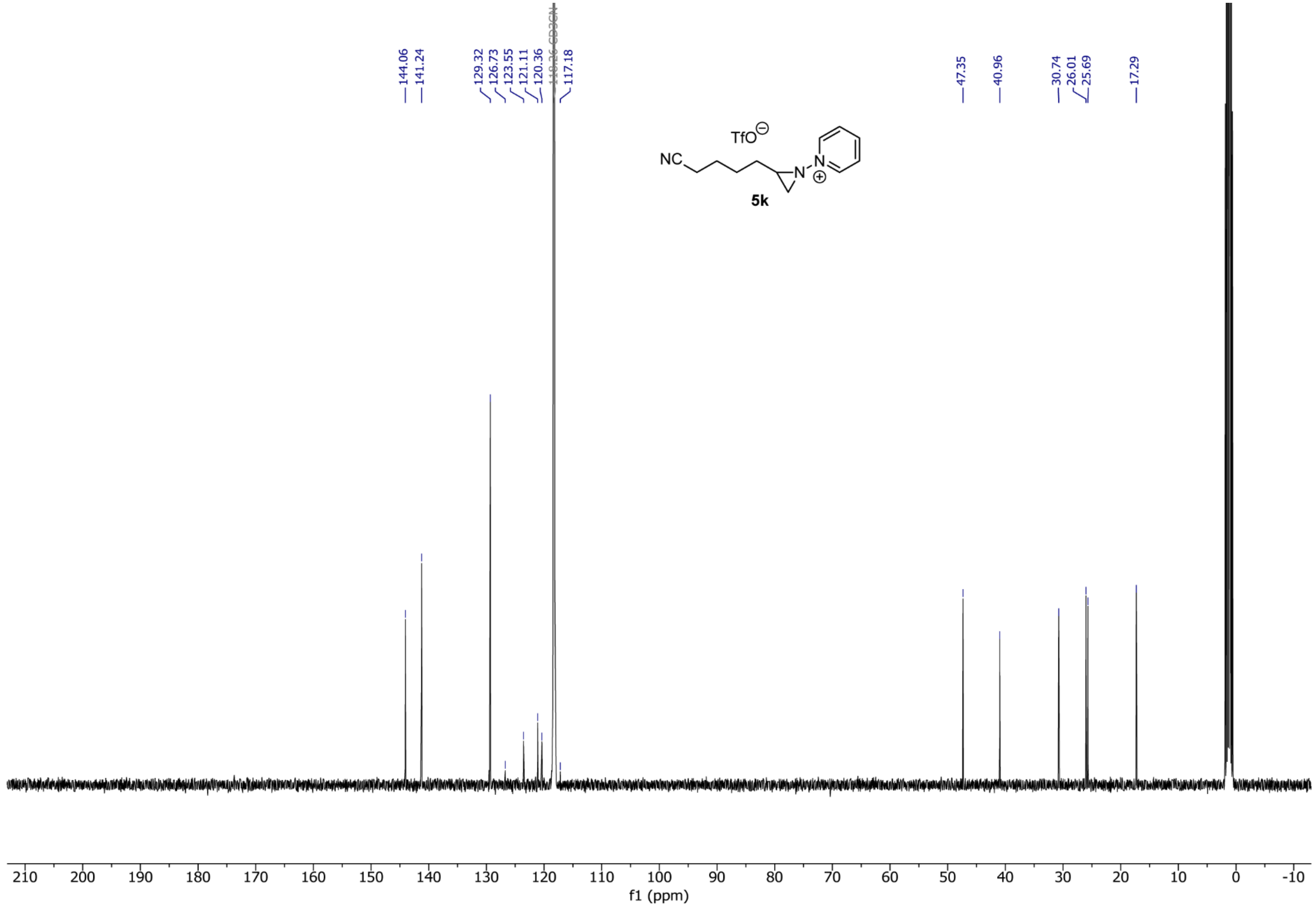

Figure S52. ^{13}C NMR spectrum of 1-(2-(4-cyanobutyl)aziridin-1-yl)pyridin-1-ium trifluoromethanesulfonate (**5k**) in CD_3CN (101 MHz) at 23 °C.

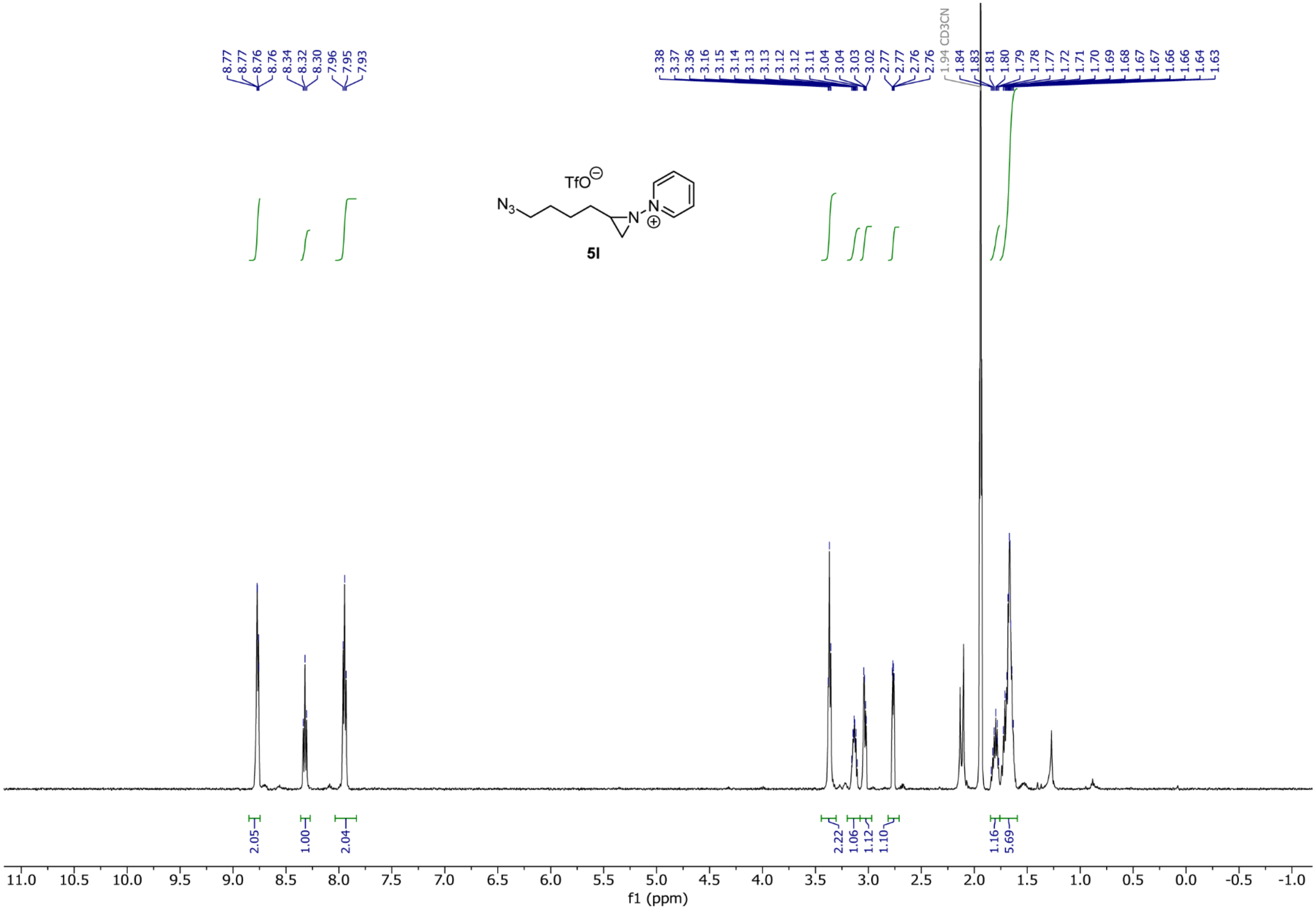

Figure S53. ^{1}H NMR spectrum of 1-(2-(4-azidobutyl)aziridin-1-yl)pyridin-1-ium trifluoromethanesulfonate (**5l**) in CD_3CN (500 MHz) at 23 °C.

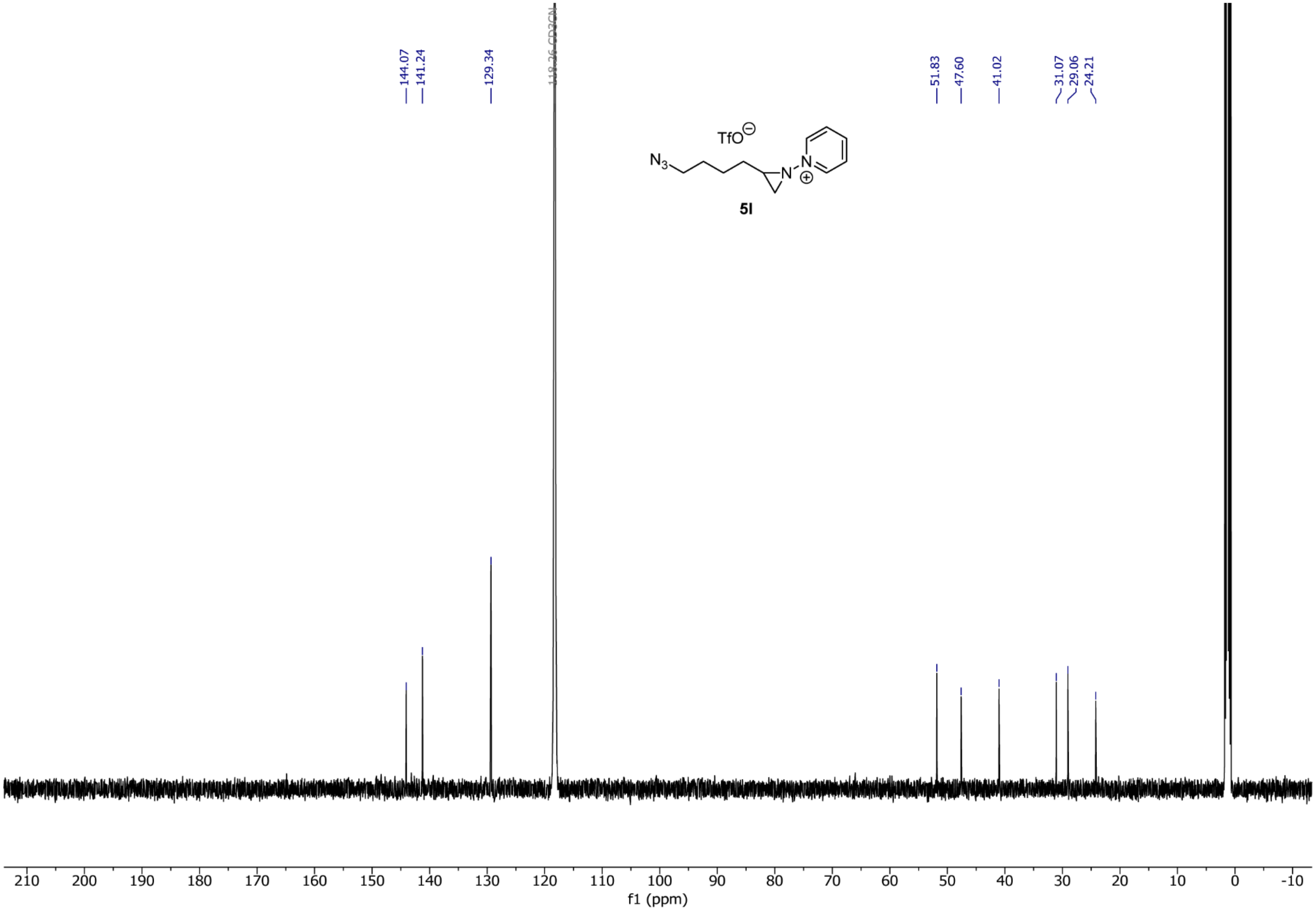

Figure S54. ^{13}C NMR spectrum of 1-(2-(4-azidobutyl)aziridin-1-yl)pyridin-1-ium trifluoromethanesulfonate (**5l**) in CD_3CN (126 MHz) at 23 °C.

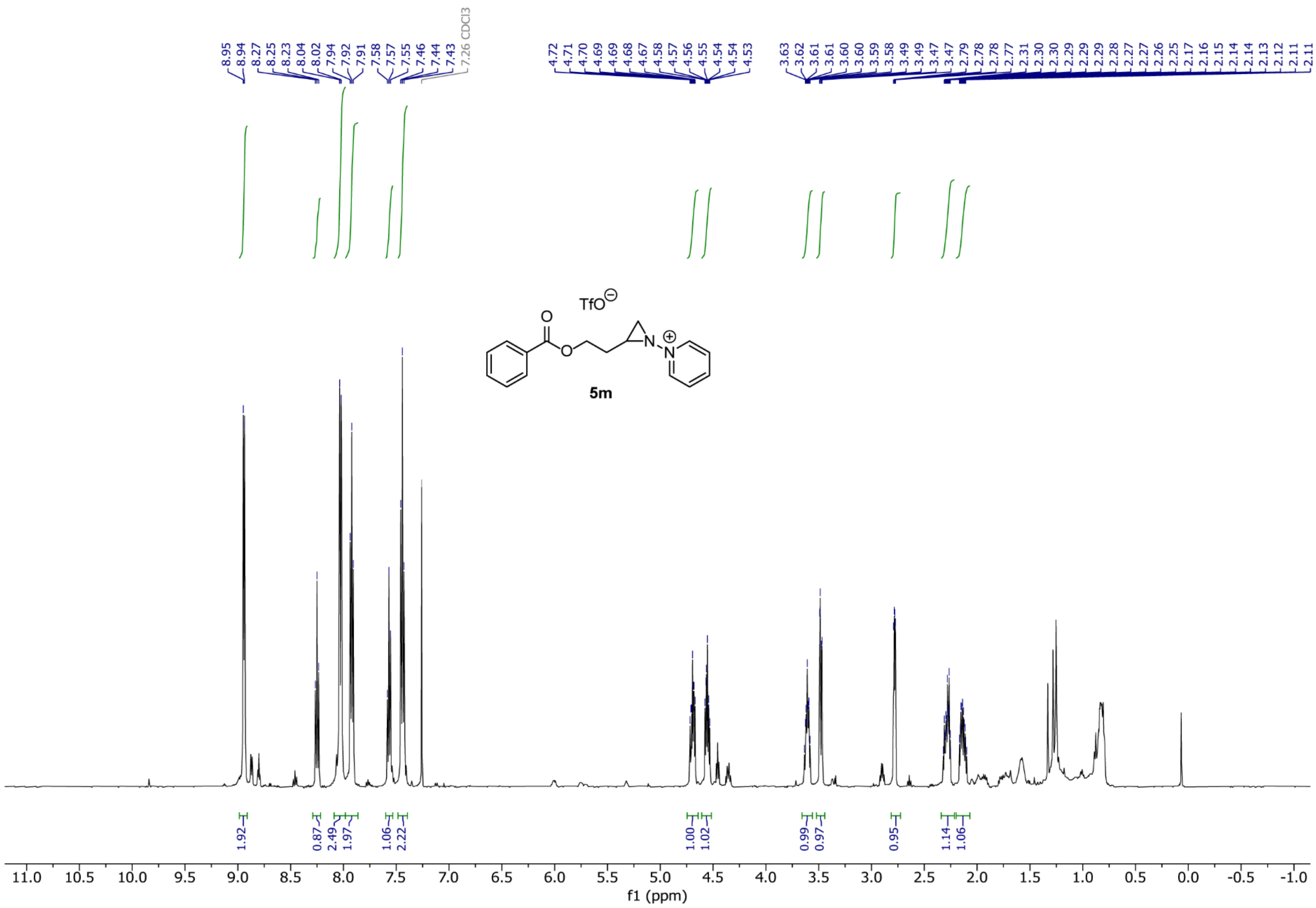

Figure S55. ^1H NMR spectrum of 1-(2-(2-(benzoyloxy)ethyl)aziridin-1-yl)pyridin-1-ium trifluoromethanesulfonate (**5m**) in $CDCl_3$ (500 MHz) at 23 °C. Further purification (HPLC or flash column) failed to improve the purity of **5m**.

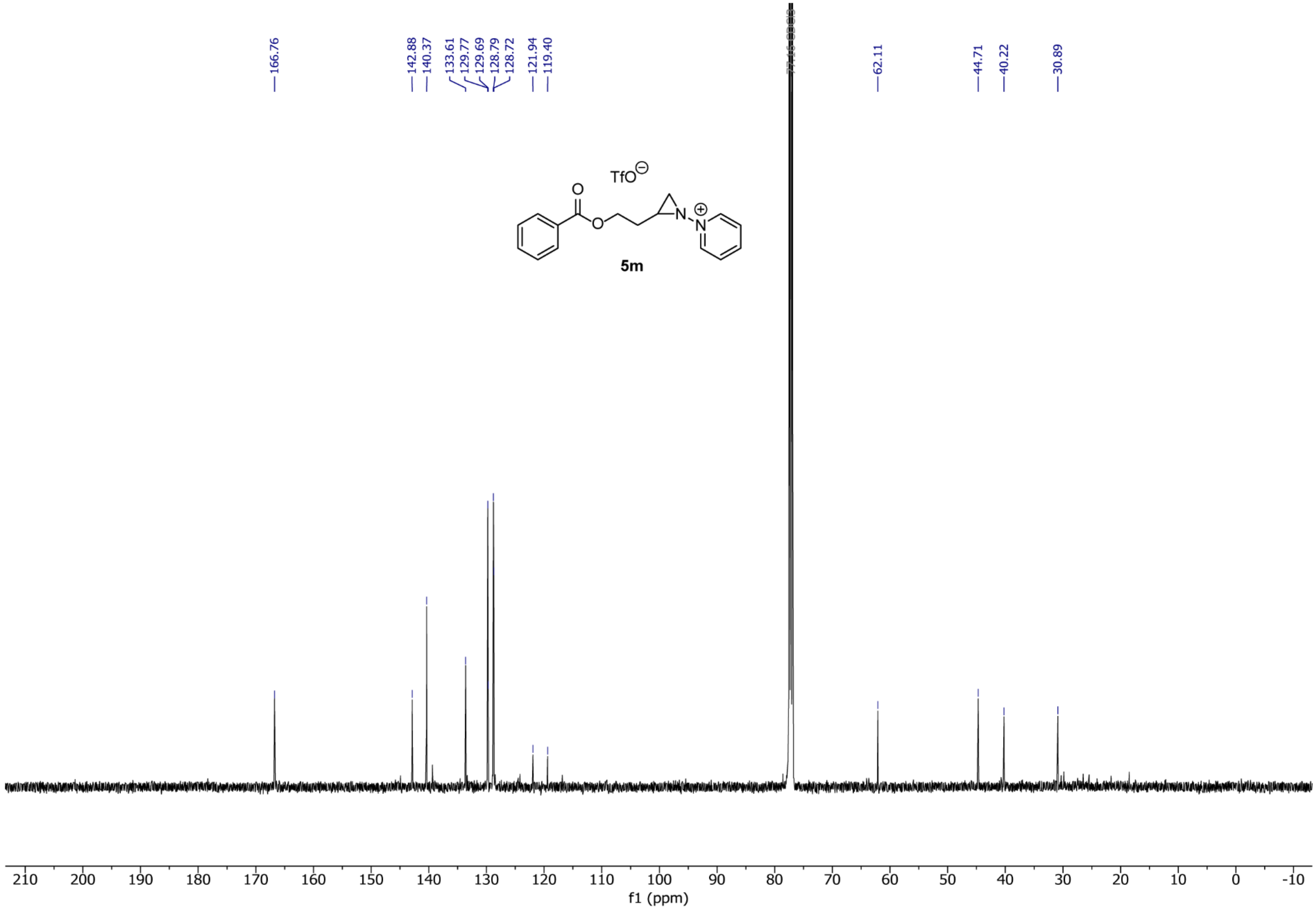

Figure S56. ^{1}H NMR spectrum of 1-(2-(2-(benzoyloxy)ethyl)aziridin-1-yl)pyridin-1-ium trifluoromethanesulfonate (**5m**) in $CDCl_3$ (126 MHz) at 23 °C.

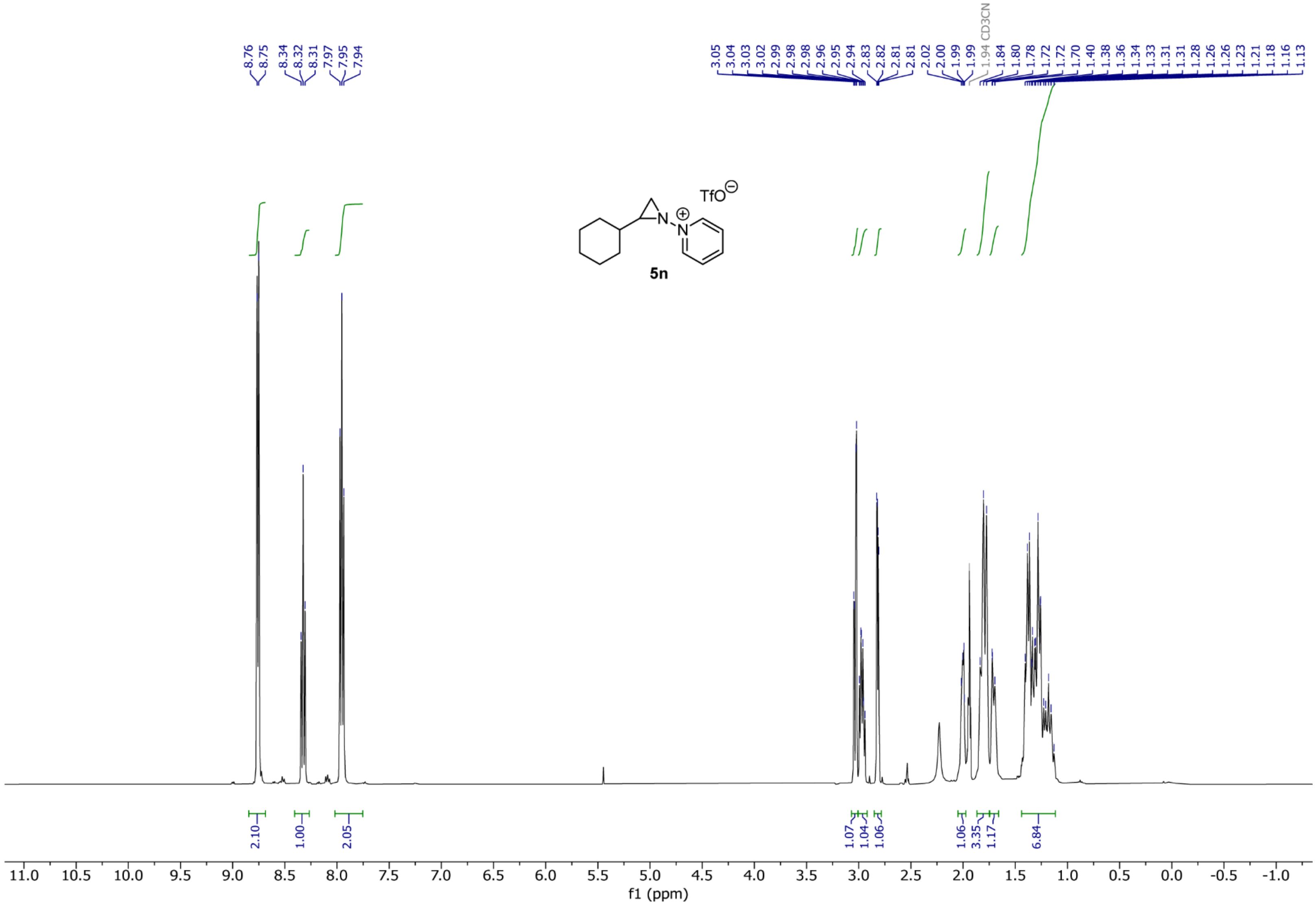

Figure S57. ^{1}H NMR spectrum of 1-(2-cyclohexylaziridin-1-yl)pyridin-1-ium trifluoromethanesulfonate (**5n**) in CD_3CN (400 MHz) at 23 °C.

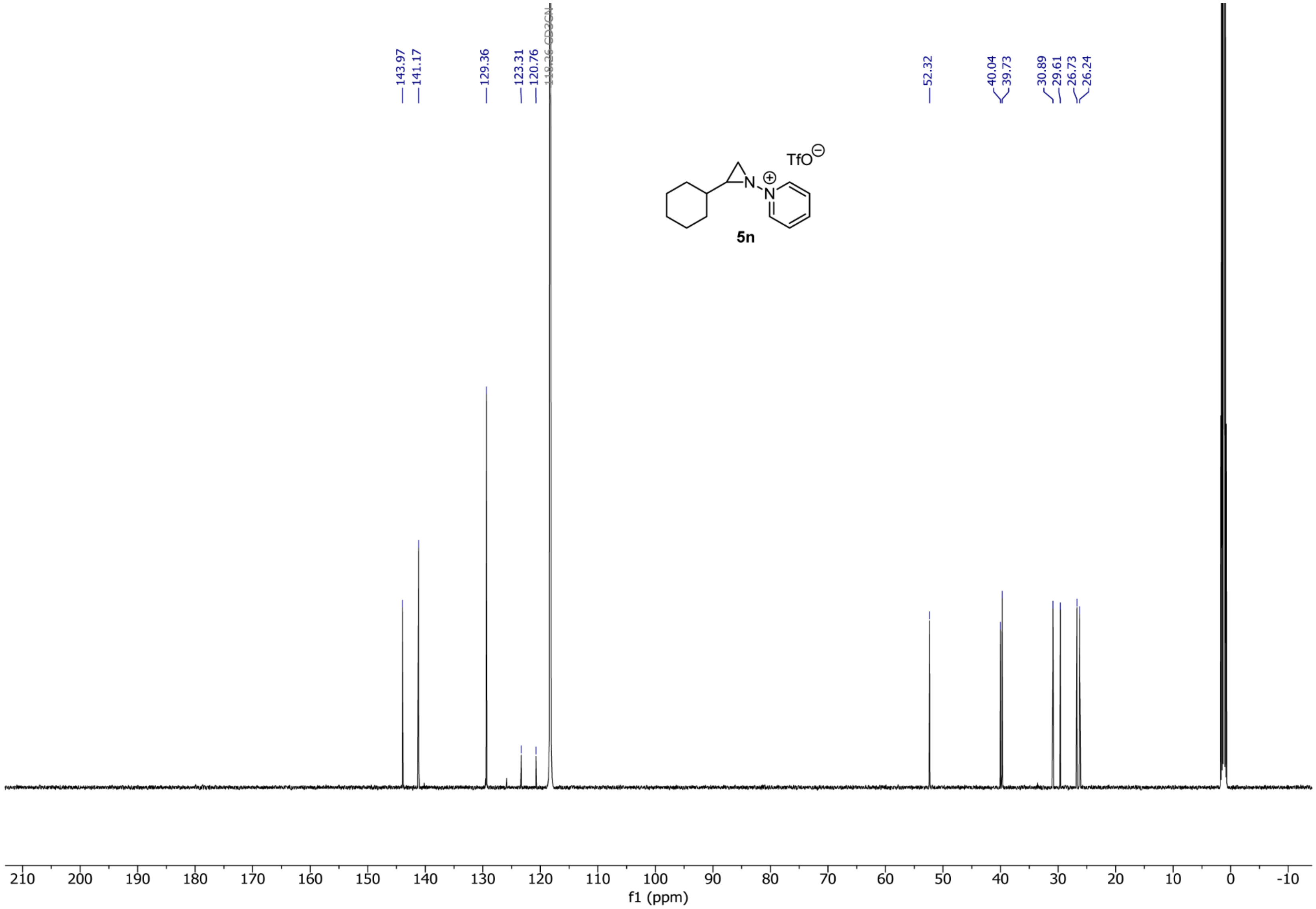

Figure S58. ^{13}C NMR spectrum of 1-(2-cyclohexylaziridin-1-yl)pyridin-1-ium trifluoromethanesulfonate (**5n**) in CD_3CN (126 MHz) at 23 °C.

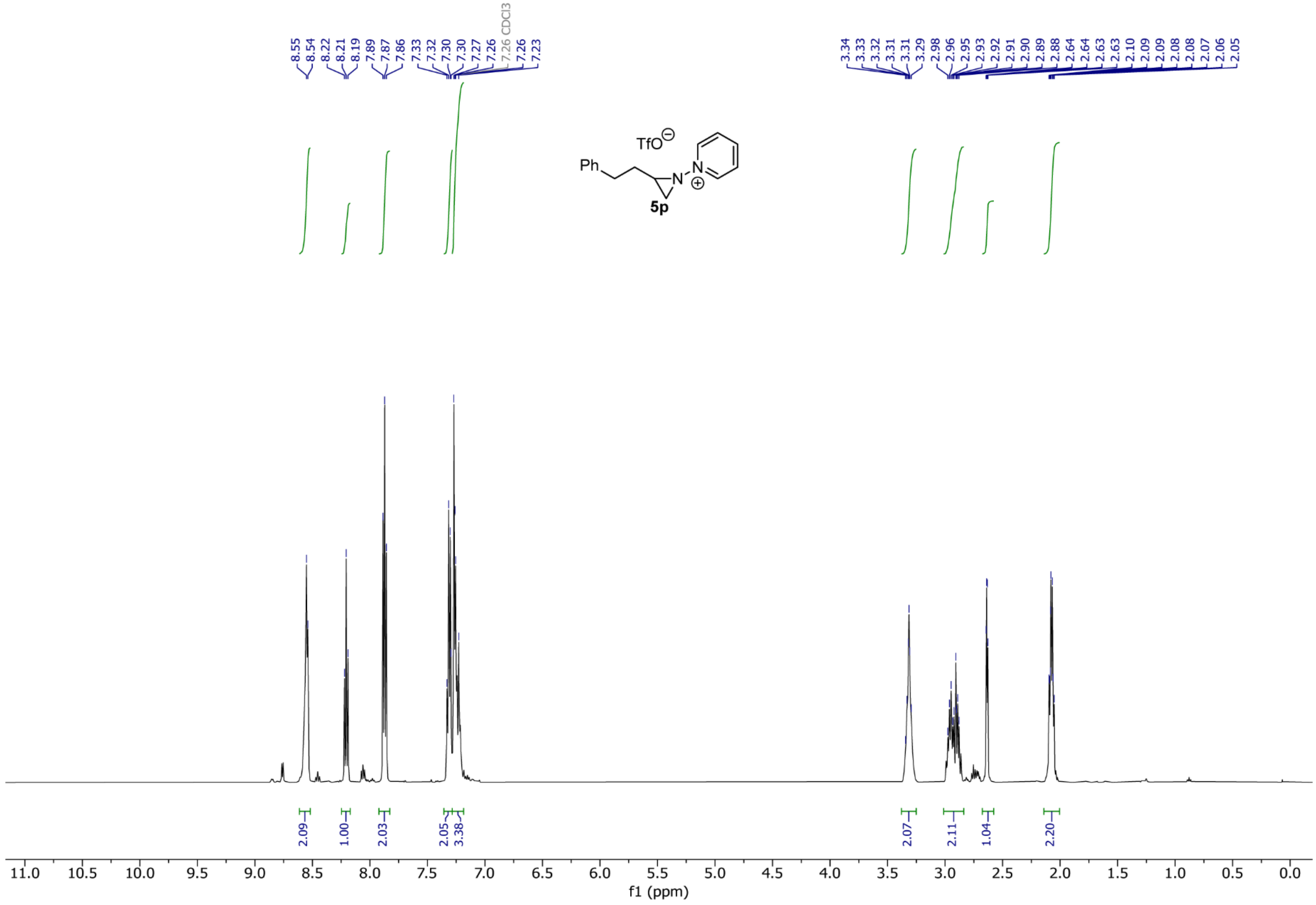

Figure S59. ^{1}H NMR spectrum of 1-(2-phenethylaziridin-1-yl)pyridin-1-ium trifluoromethanesulfonate (**5p**) in $CDCl_3$ (500 MHz) at 23 °C.

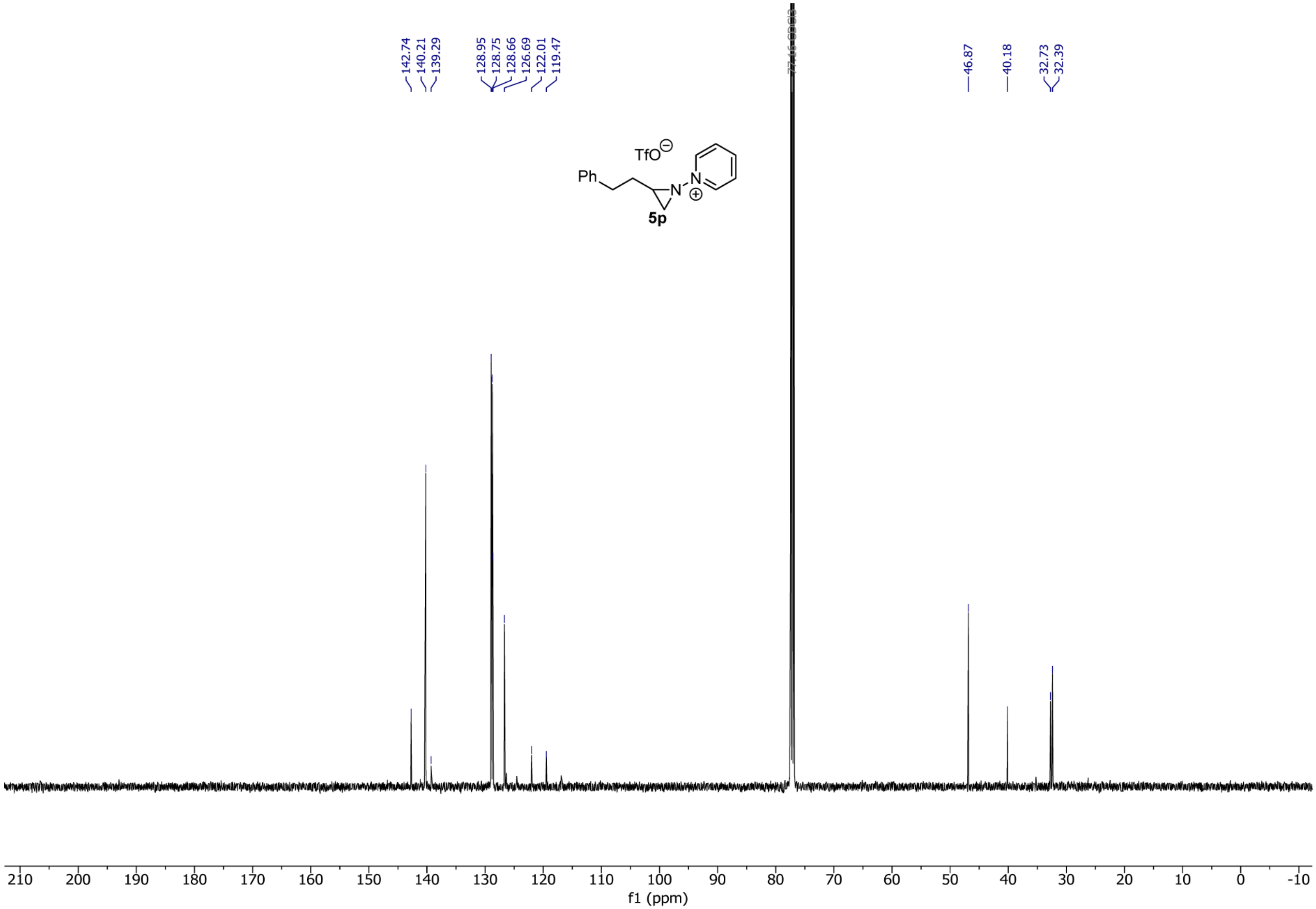

Figure S60. ^{13}C NMR spectrum of 1-(2-phenethylaziridin-1-yl)pyridin-1-ium trifluoromethanesulfonate (**5p**) in $CDCl_3$ (126 MHz) at 23 °C.

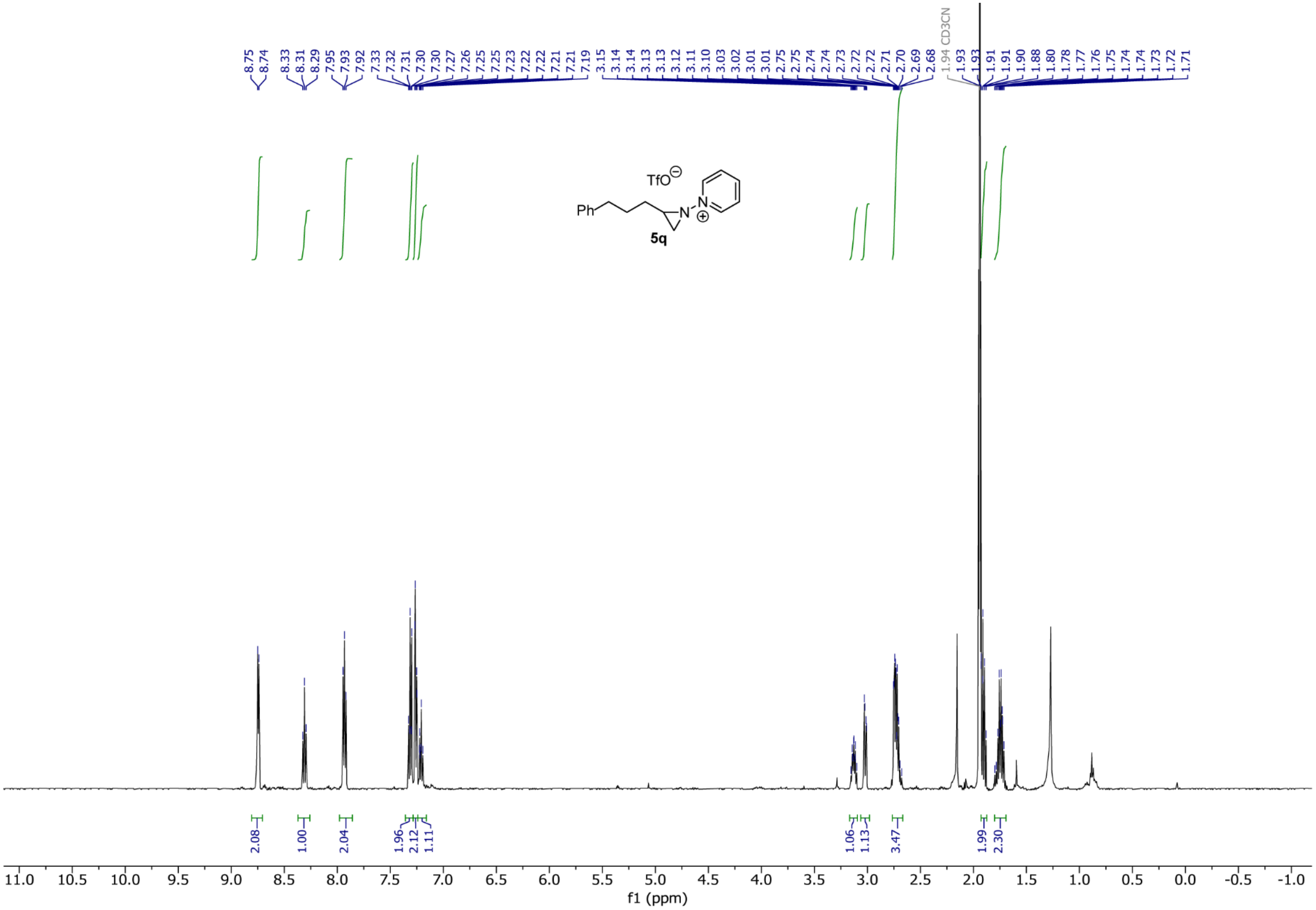

Figure S61. ^{1}H NMR spectrum of 1-(2-(3-phenylpropyl)aziridin-1-yl)pyridin-1-ium trifluoromethanesulfonate (**5q**) in CD_3CN (500 MHz) at 23 °C.

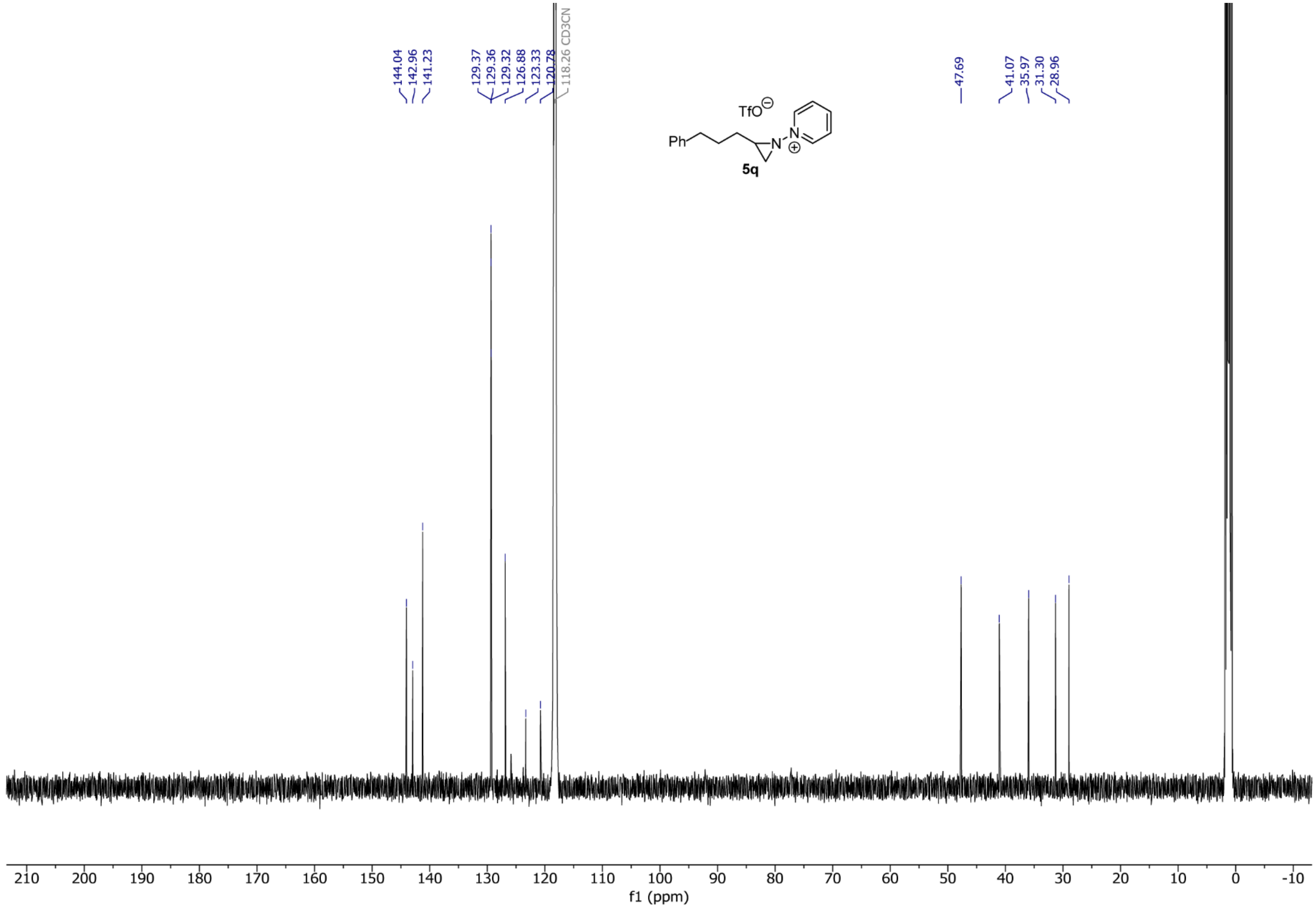

Figure S62. ^{13}C NMR spectrum of 1-(2-(3-phenylpropyl)aziridin-1-yl)pyridin-1-ium trifluoromethanesulfonate (**5q**) in CD_3CN (126 MHz) at 23 °C.

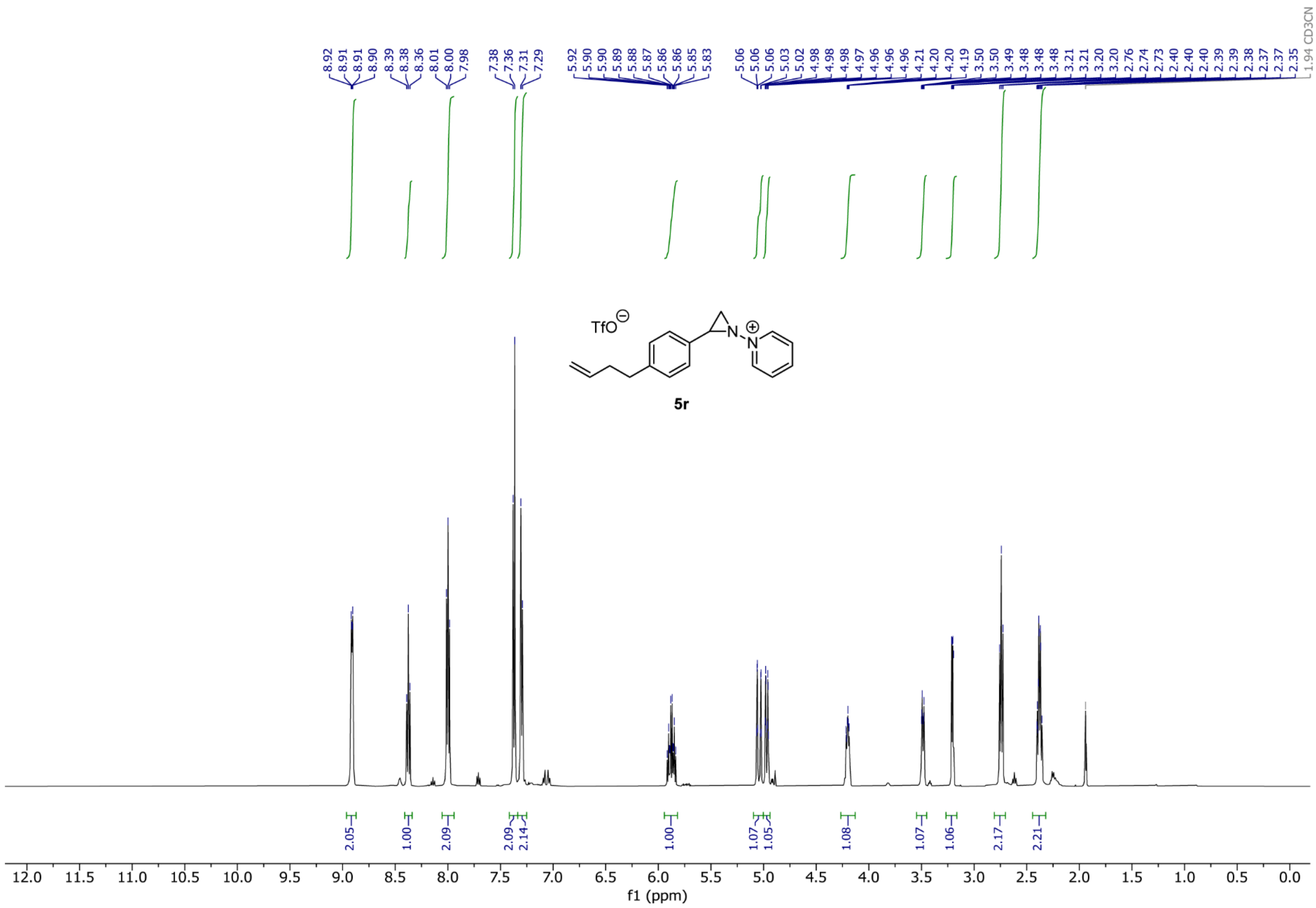

Figure S63. ^{1}H NMR spectrum of 1-(2-(4-(but-3-en-1-yl)phenyl)aziridin-1-yl)pyridin-1-ium trifluoromethanesulfonate (**5r**) in CD_3CN (500 MHz) at 23 °C.

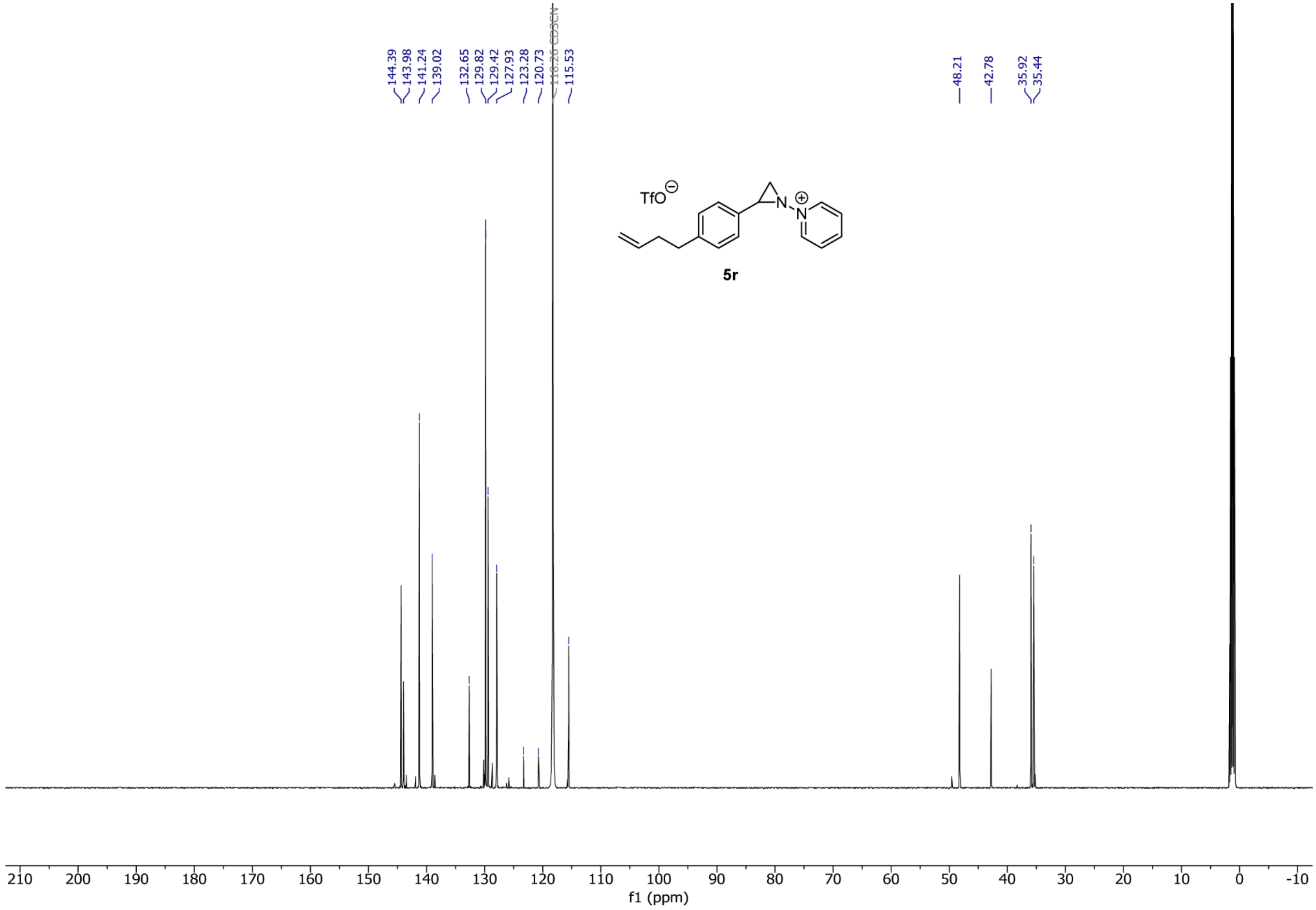

Figure S64. ^{13}C NMR spectrum of 1-(2-(4-(but-3-en-1-yl)phenyl)aziridin-1-yl)pyridin-1-ium trifluoromethanesulfonate (**5r**) in CD_3CN (126 MHz) at 23 °C.

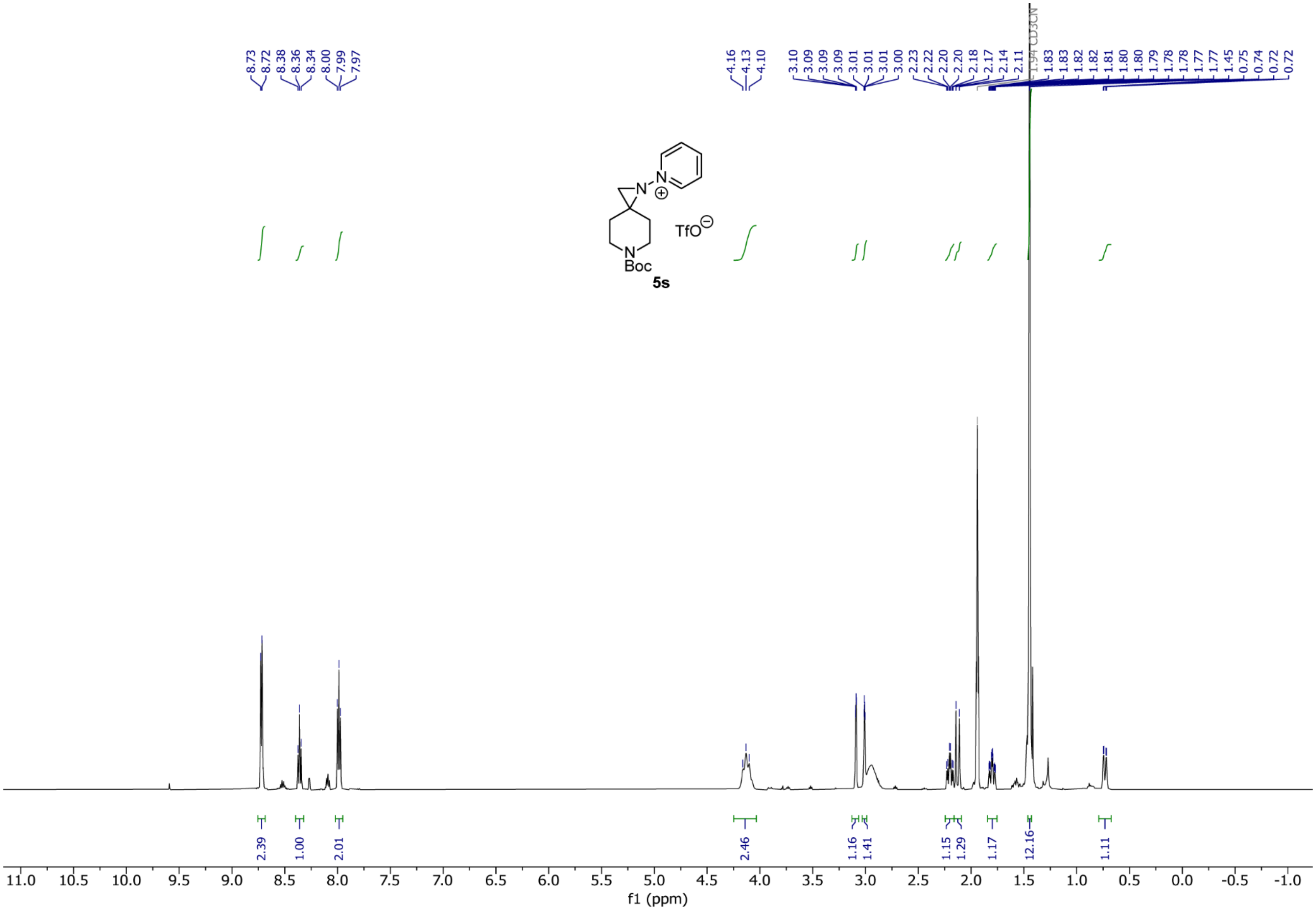

Figure S65. ^{1}H NMR spectrum of 1-(6-(*tert*-butoxycarbonyl)-1,6-diazaspiro[2.5]octan-1-yl)pyridin-1-ium trifluoromethanesulfonate (**5s**) in CD_3CN (500 MHz) at 23 °C. Further purification (HPLC or flash column) failed to improve the purity of **5s**, but **5s** could be carried to subsequent reactions without issue.

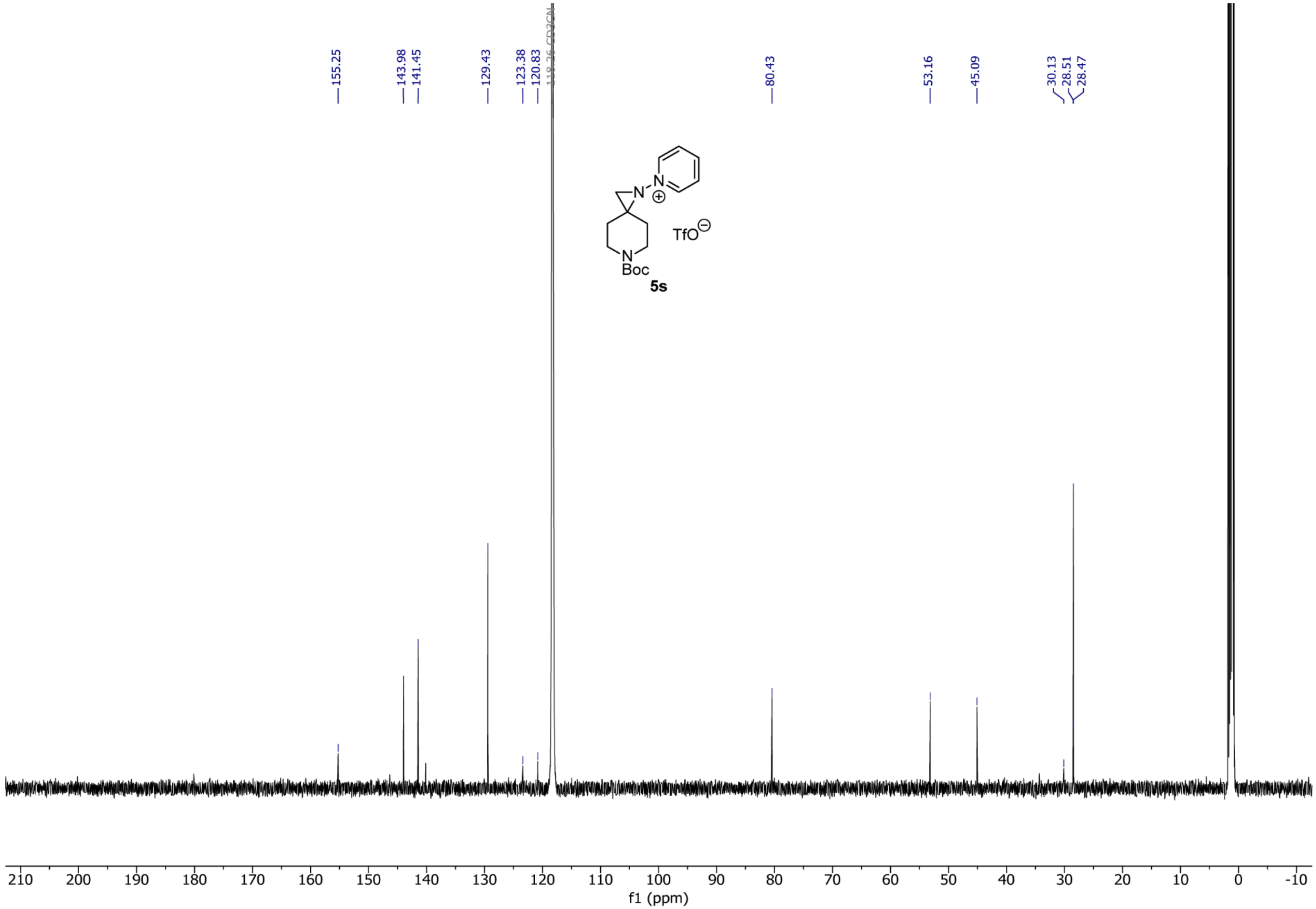

Figure S66. ^{13}C NMR spectrum of 1-(6-(*tert*-butoxycarbonyl)-1,6-diazaspiro[2.5]octan-1-yl)pyridin-1-ium trifluoromethanesulfonate (**5s**) in CD_3CN (126 MHz) at 23 °C.

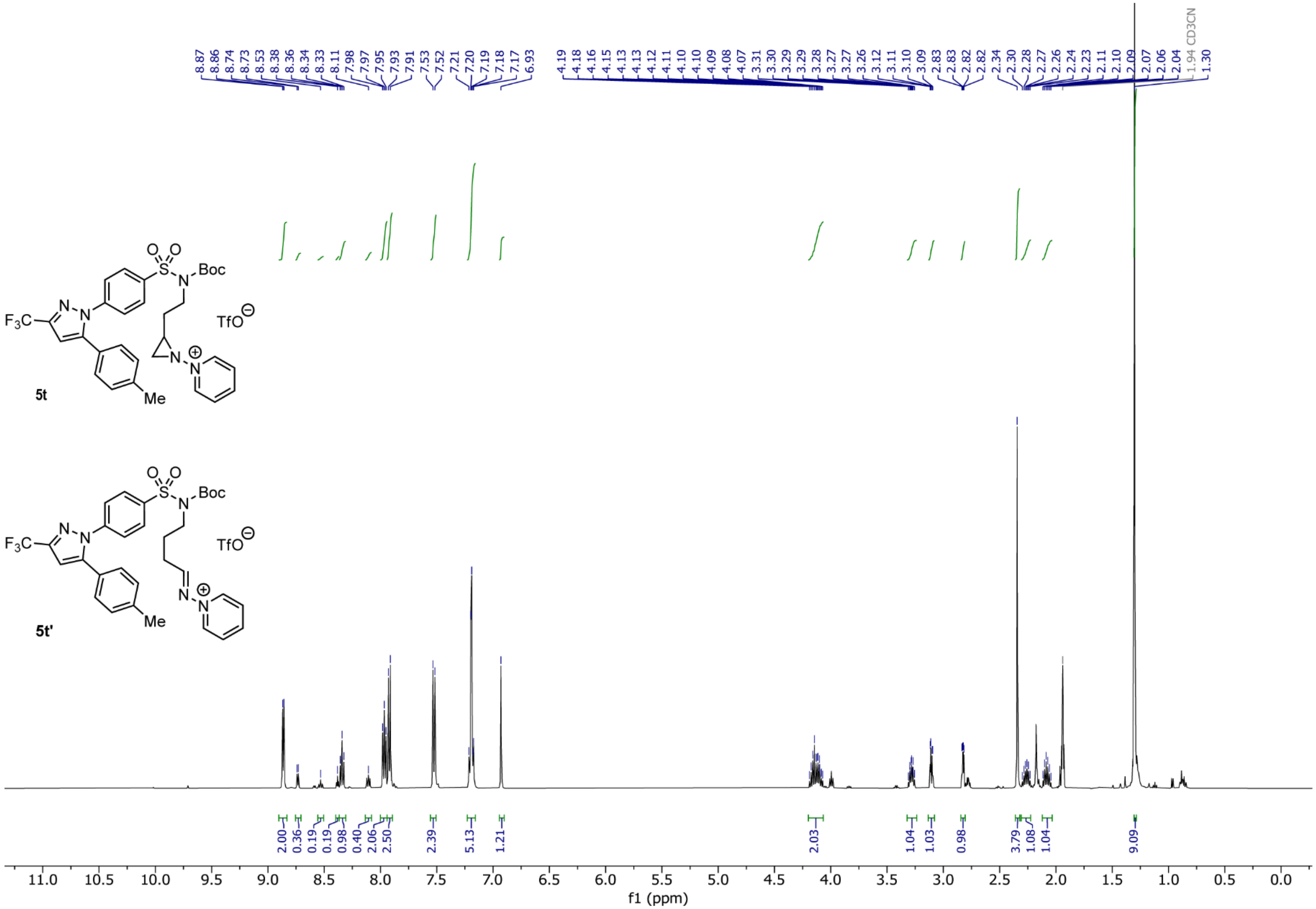

Figure S67. ^{1}H NMR spectrum of 1-(2-(2-((*N*-(*tert*-butoxycarbonyl)-4-(5-(*p*-tolyl)-3-(trifluoromethyl)-1*H*-pyrazol-1-yl)phenyl)sulfonamido)ethyl)aziridin-1-yl)pyridin-1-ium trifluoromethanesulfonate (**5t**) in CD_3CN (500 MHz) at 23 °C. This sample contains 15% of the imine byproduct **5t'**, indicated by peaks at 8.74, 8.53, 8.36, and 8.11 ppm.

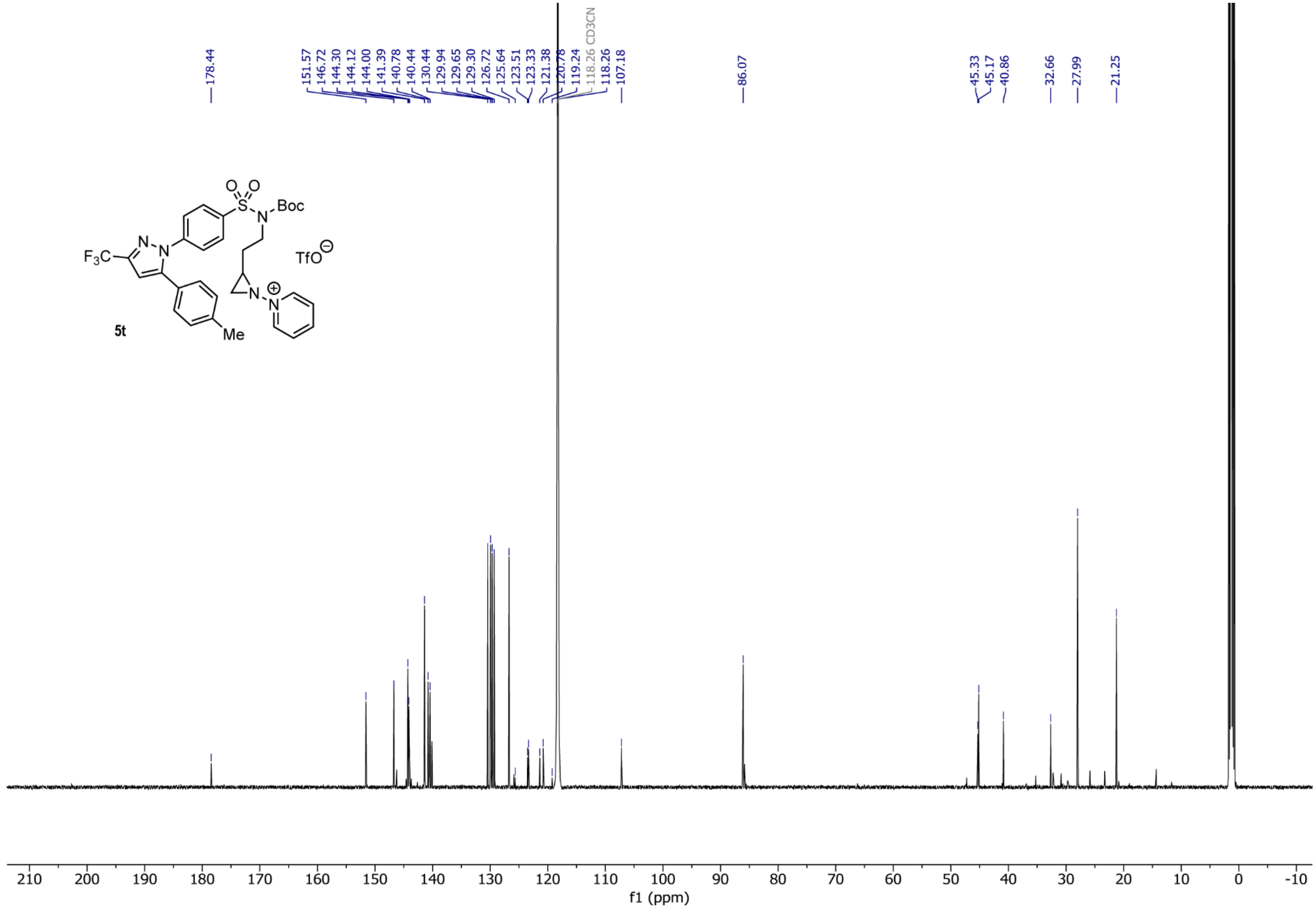

Figure S68. ^{13}C NMR spectrum of 1-(2-(2-((*N*-(*tert*-butoxycarbonyl)-4-(5-(*p*-tolyl)-3-(trifluoromethyl)-1*H*-pyrazol-1-yl)phenyl)sulfonamido)ethyl)aziridin-1-yl)pyridin-1-ium trifluoromethanesulfonate (**5t**) in CD_3CN (126 MHz) at 23 °C.

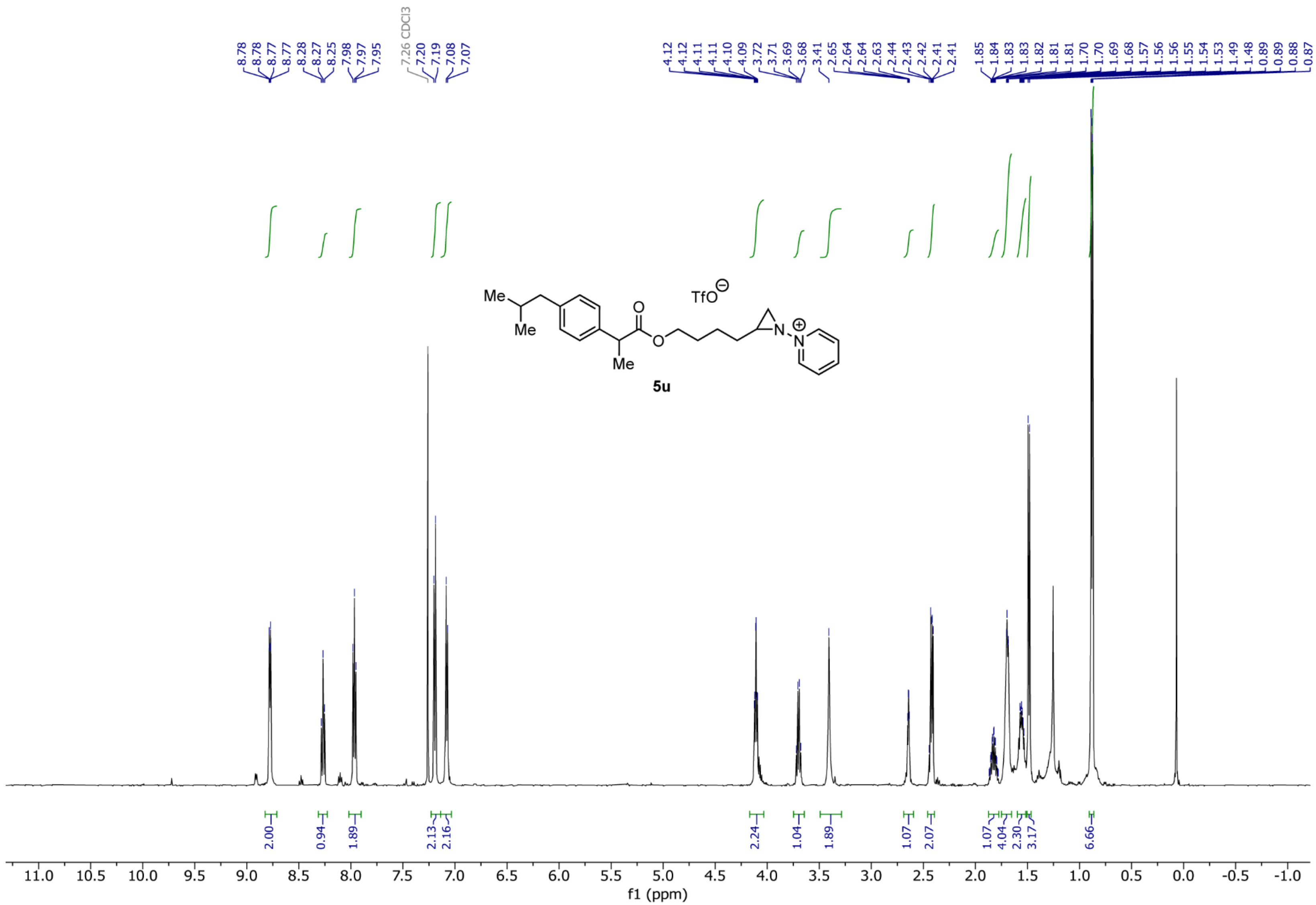

Figure S69. ^{1}H NMR spectrum of 1-(2-(4-((2-(4-isobutylphenyl)propanoyl)oxy)butyl)aziridin-1-yl)pyridin-1-ium trifluoromethanesulfonate (**5u**) in $CDCl_3$ (500 MHz) at 23 °C.

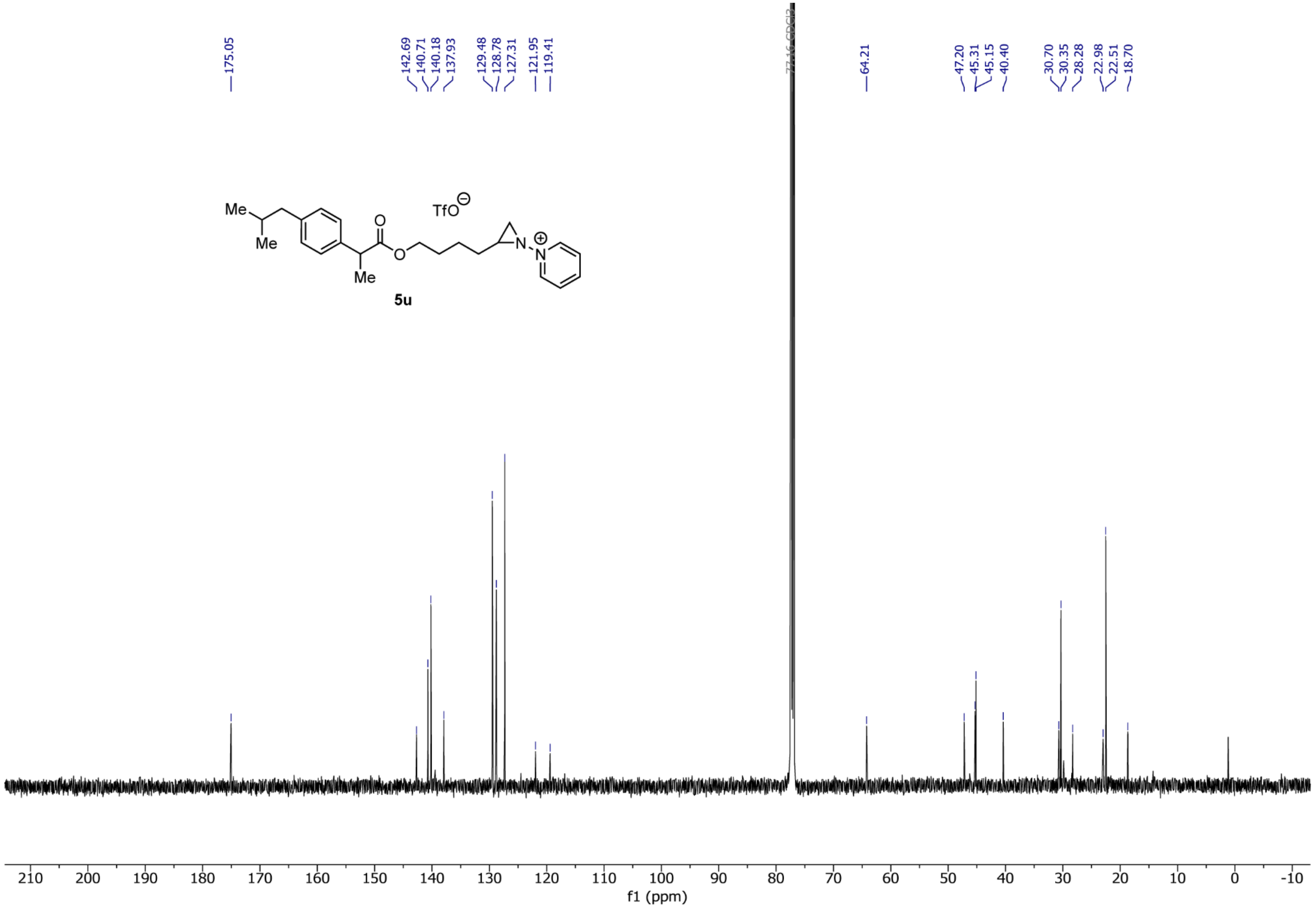

Figure S70. ^{13}C NMR spectrum of 1-(2-(4-((2-(4-isobutylphenyl)propanoyl)oxy)butyl)aziridin-1-yl)pyridin-1-ium trifluoromethanesulfonate (**5u**) in $CDCl_3$ (126 MHz) at 23 °C.

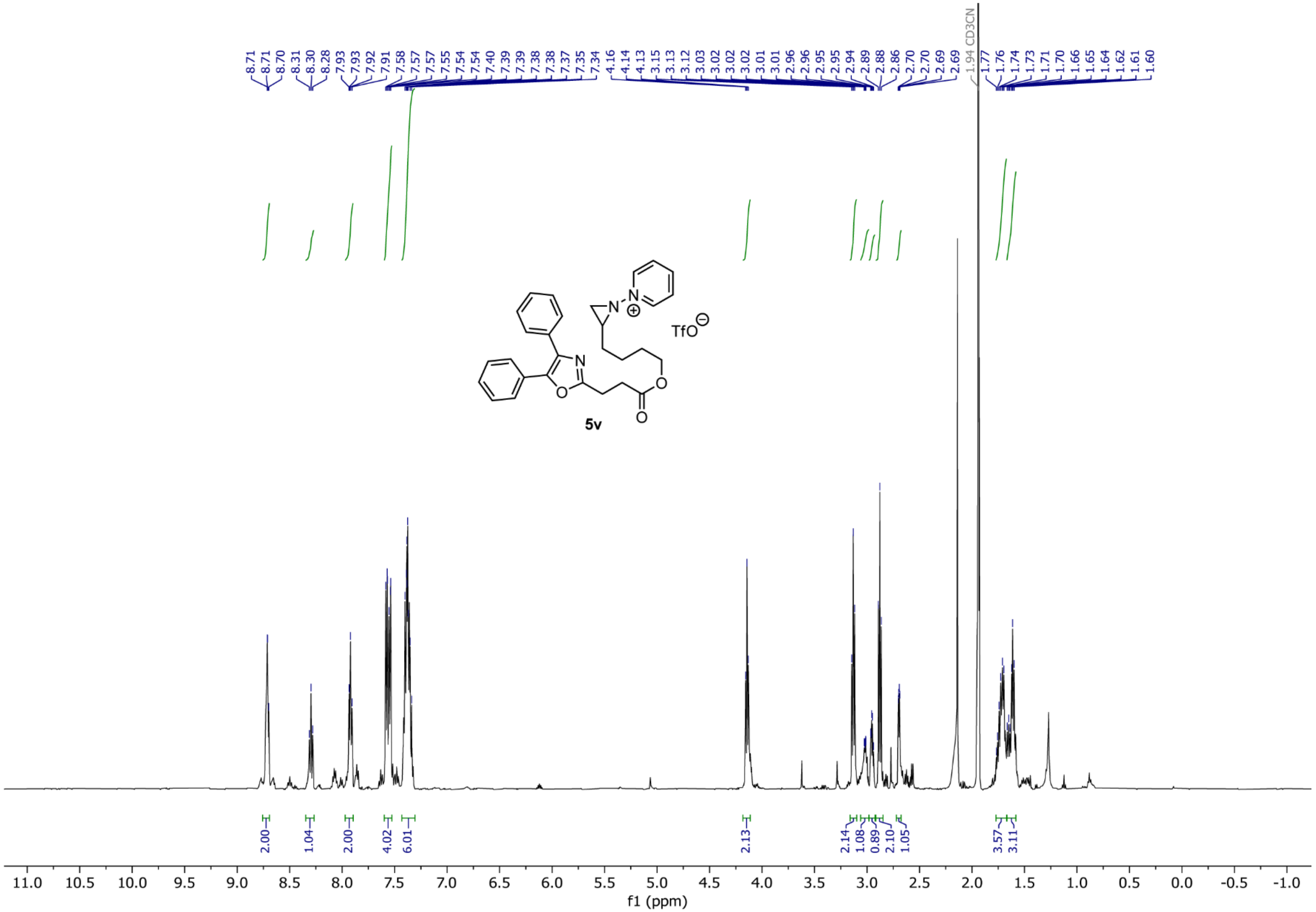

Figure S71. ^{1}H NMR spectrum of 1-(2-(4-((3-(4,5-diphenyloxazol-2-yl)propanoyl)oxy)butyl)aziridin-1-yl)pyridin-1-ium trifluoromethanesulfonate (**5v**) in CD_3CN (500 MHz) at 23 °C. Further purification (HPLC or flash column) failed to improve the purity of **5v.**

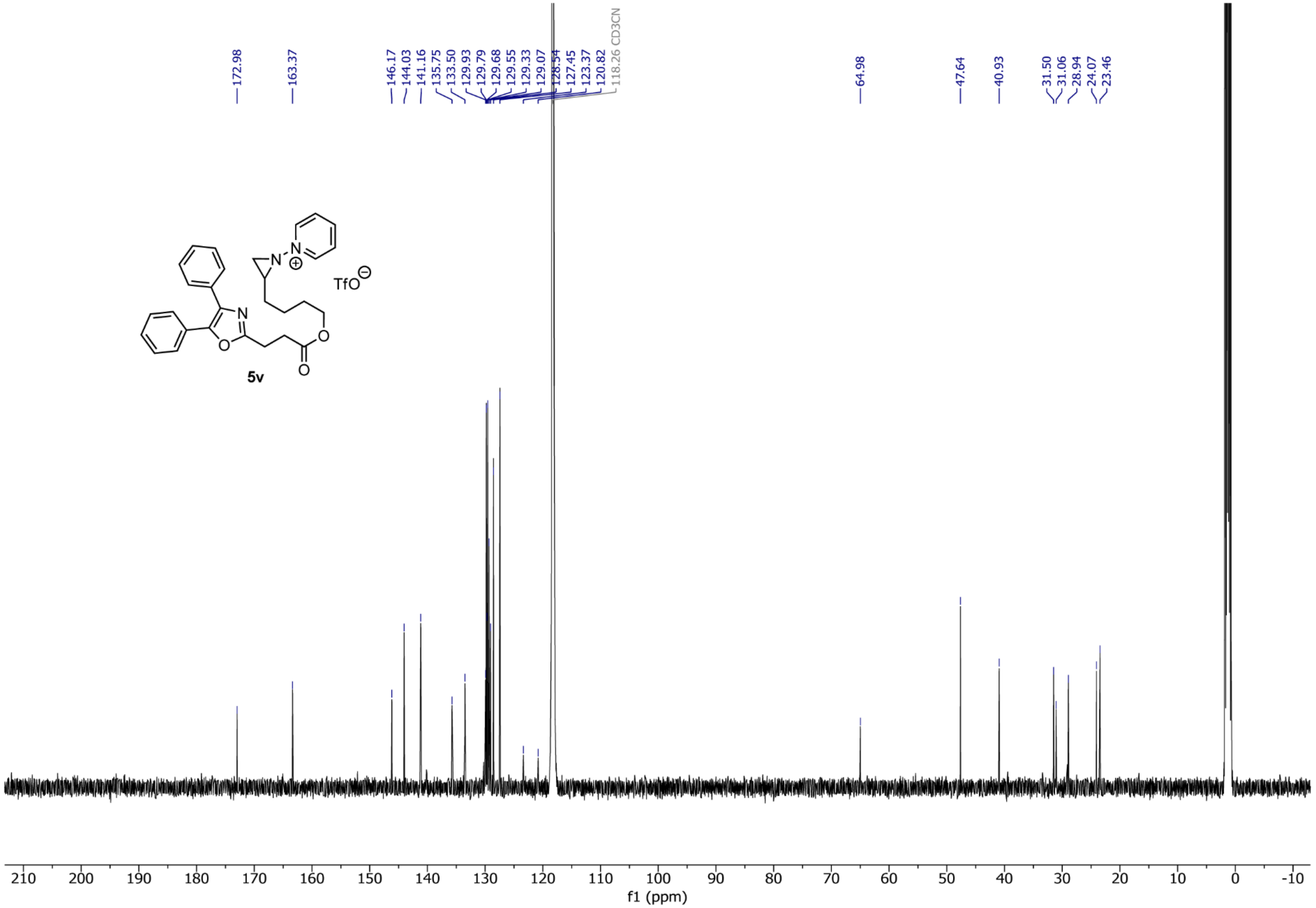

Figure S72. ^{13}C NMR spectrum of 1-(2-(4-((3-(4,5-diphenyloxazol-2-yl)propanoyl)oxy)butyl)aziridin-1-yl)pyridin-1-ium trifluoromethanesulfonate (**5v**) in CD_3CN (126 MHz) at 23 °C.

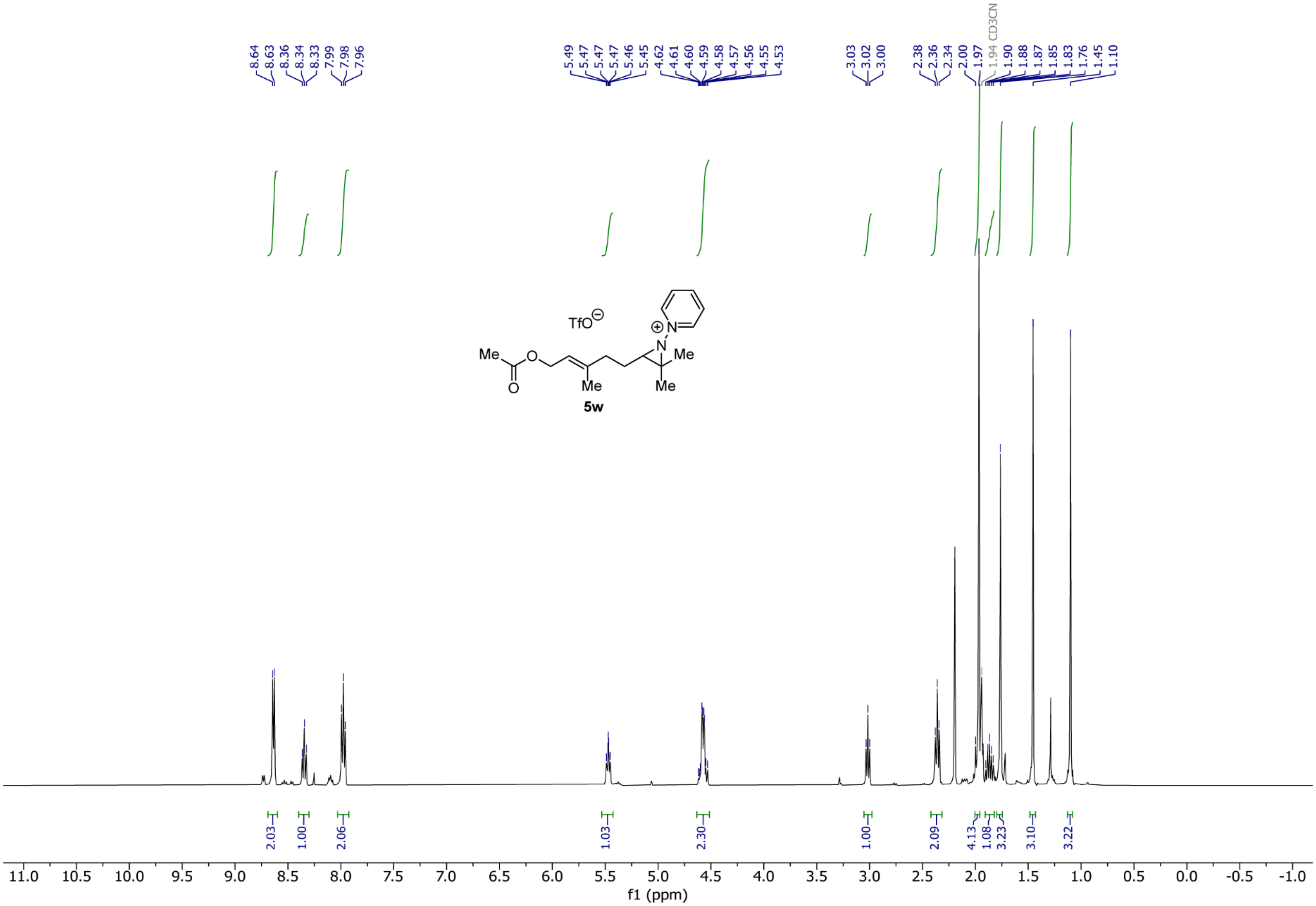

Figure S73. ^{1}H NMR spectrum of (*E*)-1-(3-(5-acetoxy-3-methylpent-3-en-1-yl)-2,2-dimethylaziridin-1-yl)pyridin-1-ium trifluoromethanesulfonate (**5w**) in CD_3CN (400 MHz) at 23 °C. Extra peaks are attributed to the imine byproduct.

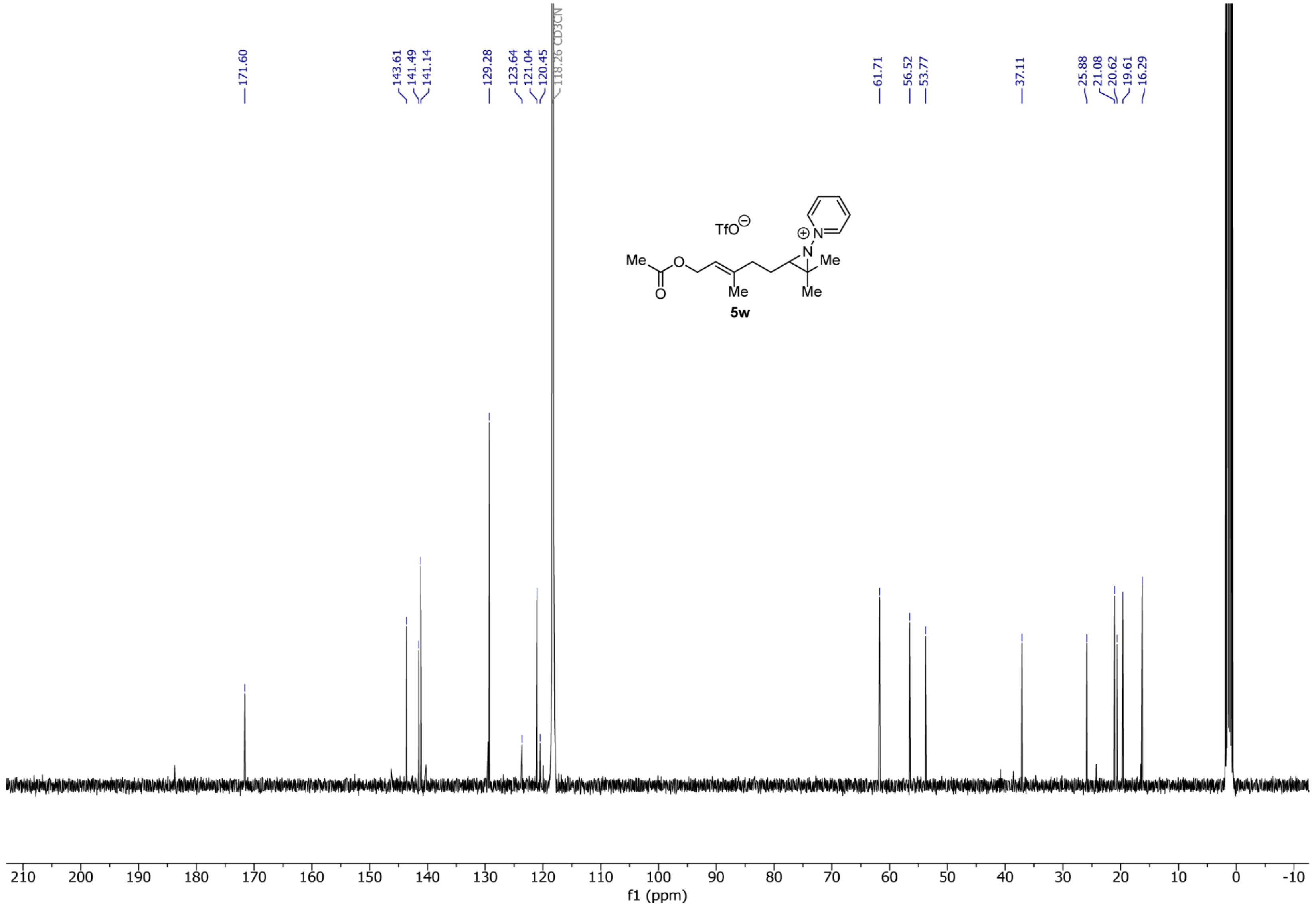

Figure S74. ^{13}C NMR spectrum of (*E*)-1-(3-(5-acetoxy-3-methylpent-3-en-1-yl)-2,2-dimethylaziridin-1-yl)pyridin-1-ium trifluoromethanesulfonate (**5w**) in CD_3CN (101 MHz) at 23 °C.

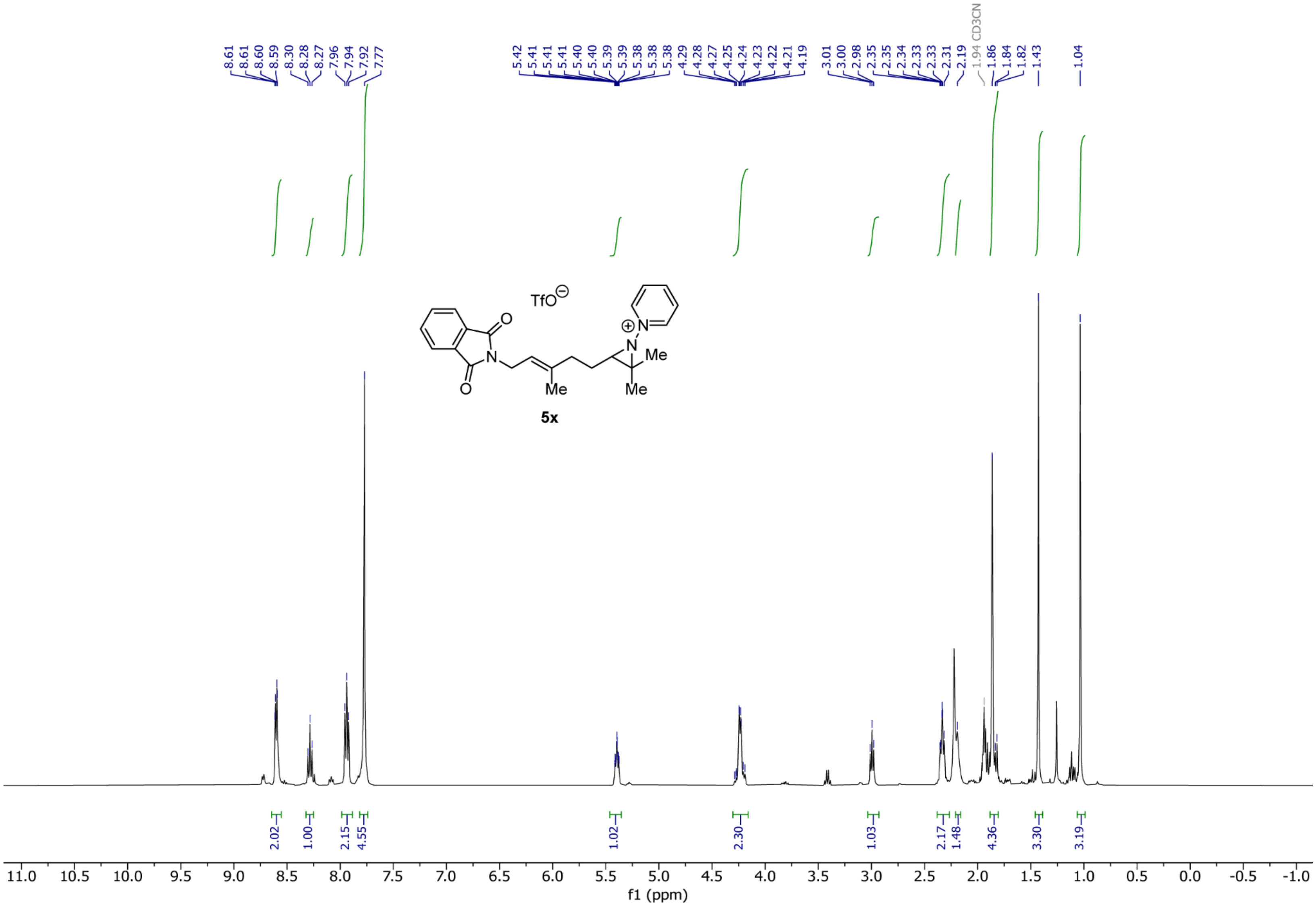

Figure S75. ^{1}H NMR spectrum of (E)-1-(3-(5-(1,3-dioxoisoindolin-2-yl)-3-methylpent-3-en-1-yl)-2,2-dimethylaziridin-1-yl)pyridin-1-ium trifluoromethanesulfonate (**5x**) in CD_3CN (400 MHz) at 23 °C. Extra peaks are attributed to the imine byproduct.

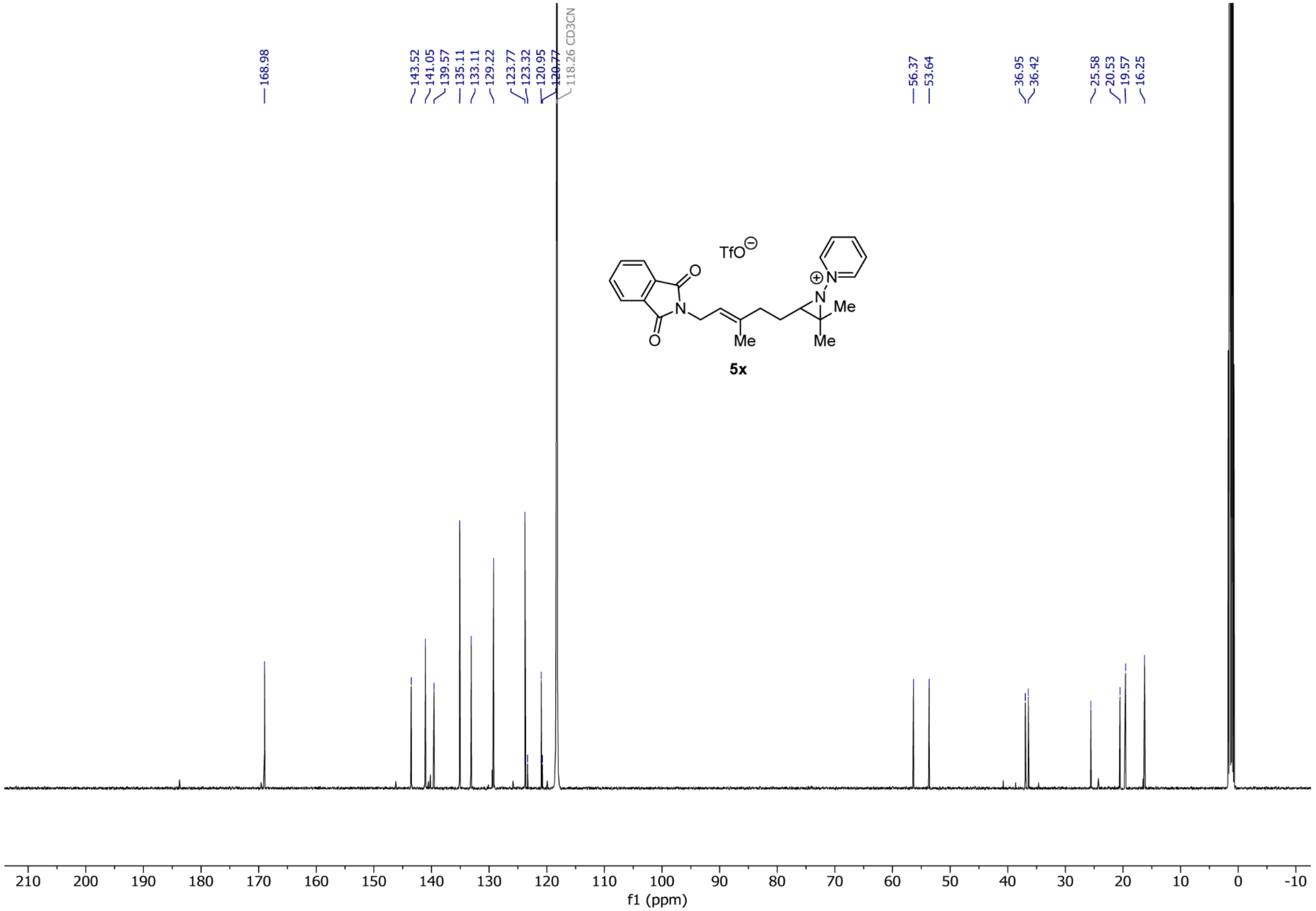

Figure S76. ^{13}C NMR spectrum of (*E*)-1-(3-(5-(1,3-dioxoisoindolin-2-yl)-3-methylpent-3-en-1-yl)-2,2-dimethylaziridin-1-yl)pyridin-1-ium trifluoromethanesulfonate (**5x**) in CD_3CN (126 MHz) at 23 °C.

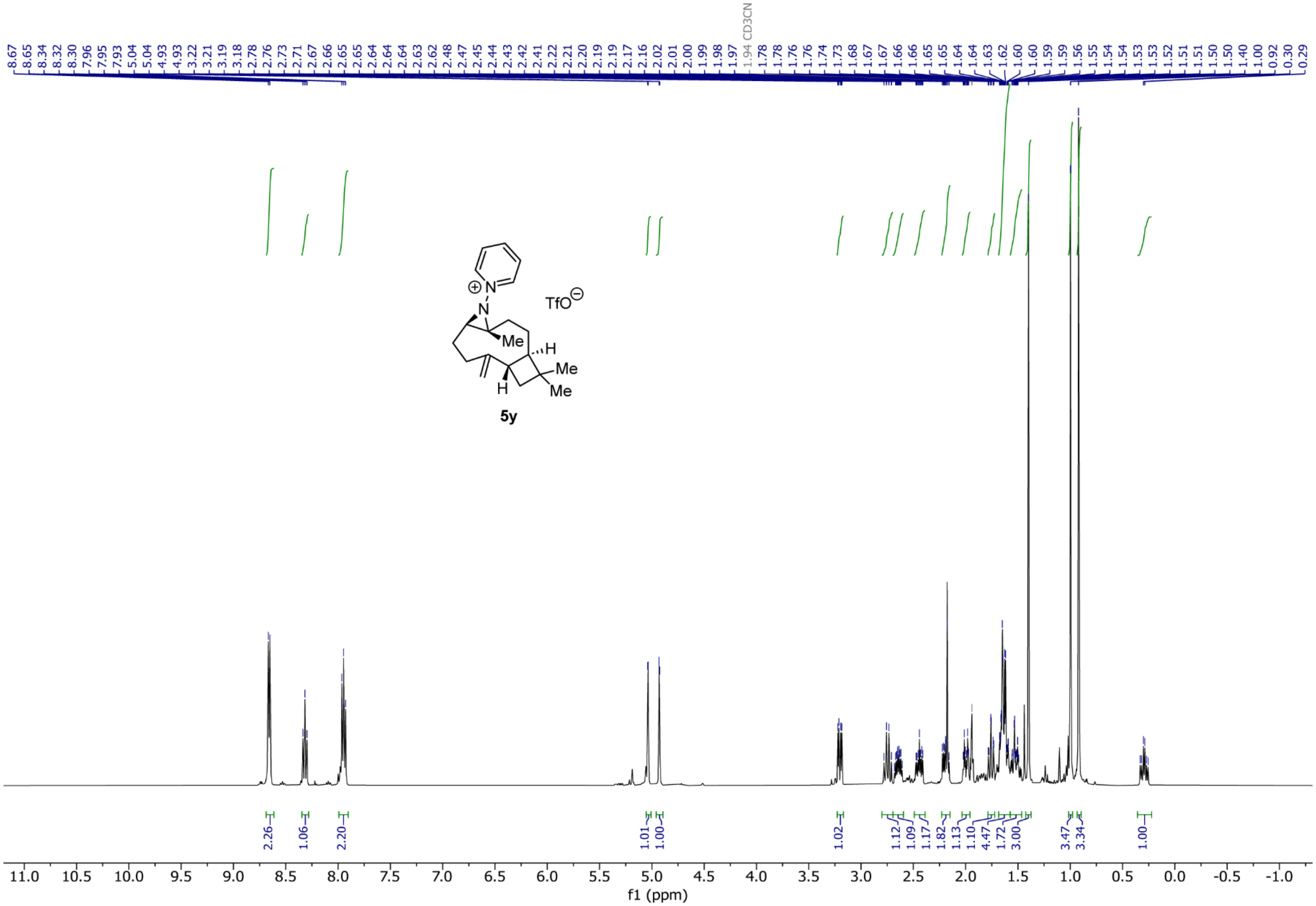

Figure S77. ^{1}H NMR spectrum of 1-((1*R*,4*R*,6*R*,10*S*)-4,12,12-trimethyl-9-methylene-5-azatricyclo[8.2.0.0^{4,6}]dodecan-5-yl)pyridin-1-ium trifluoromethanesulfonate (**5y**) in CD_3CN (400 MHz) at 23 °C.

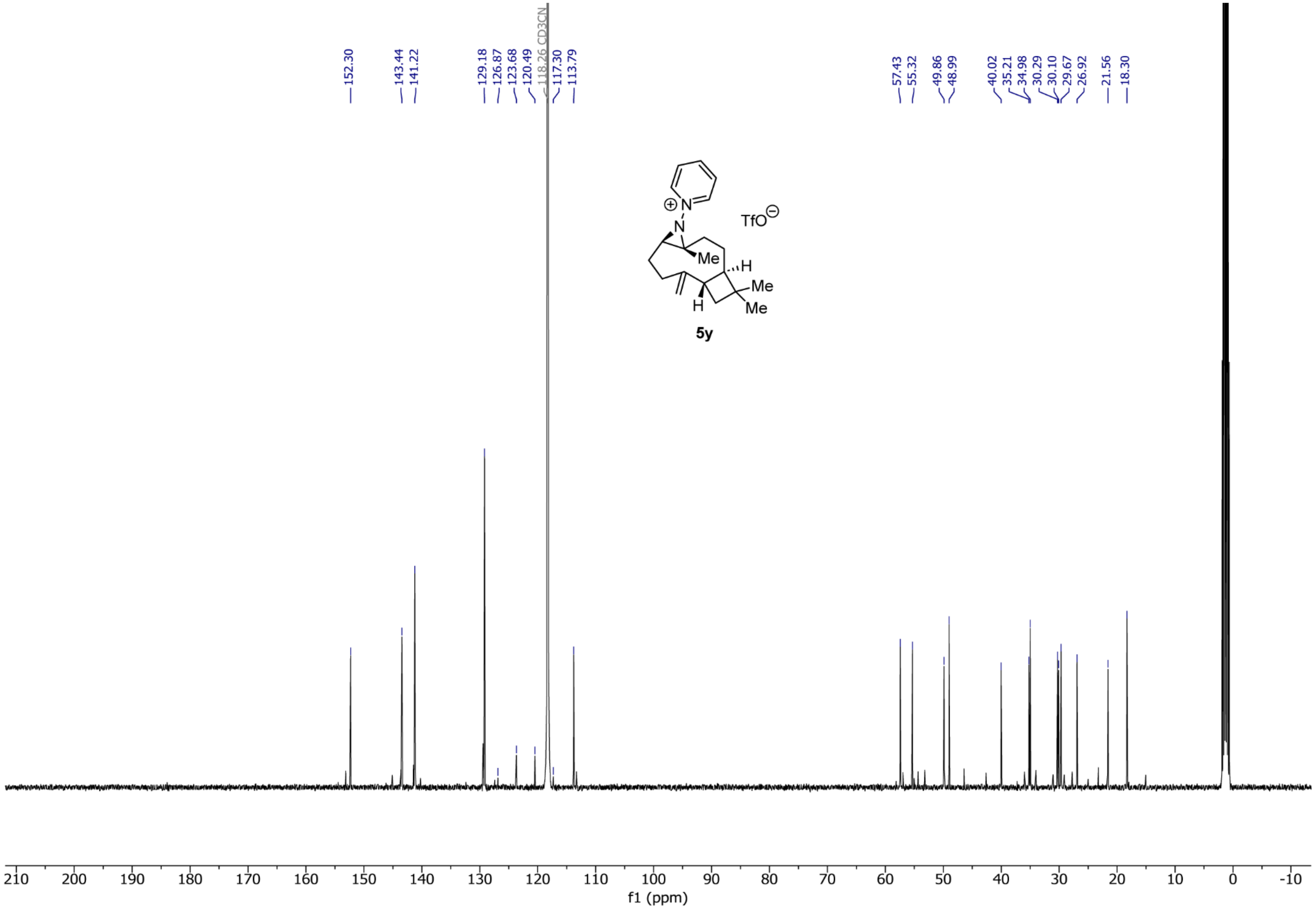

Figure S78. ^{13}C NMR spectrum of 1-((1*R*,10*S*)-4,12,12-trimethyl-9-methylene-5-azatricyclo[8.2.0.0^{4,6}]dodecan-5-yl)pyridin-1-ium trifluoromethanesulfonate (**5y**) in CD_3CN (101 MHz) at 23 °C. Extra peaks are attributed to the diastereomer.

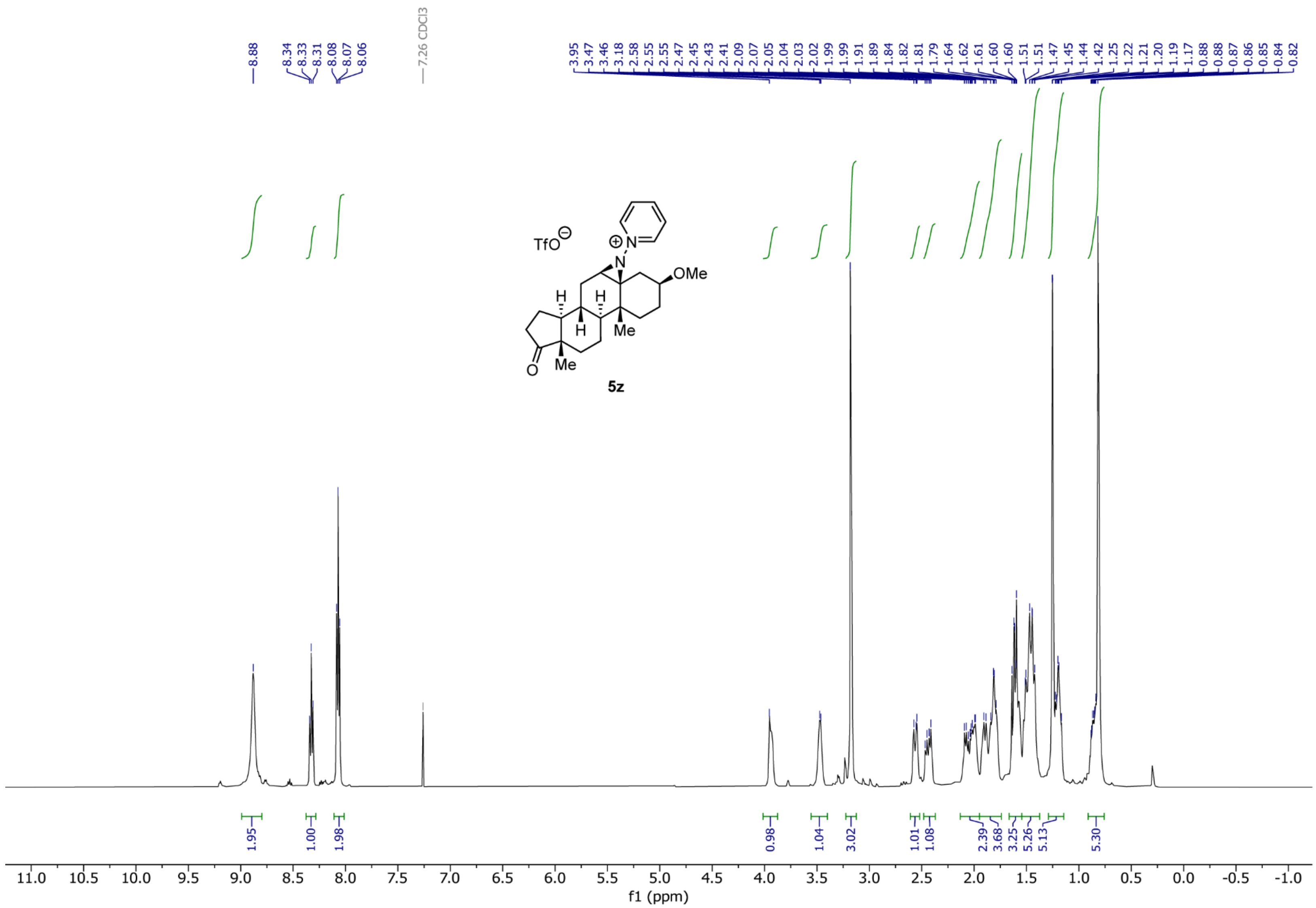

Figure S79. ^{1}H NMR spectrum of 1-((3*S*,4a*S*,5a*R*,6a*R*,6b*S*,9a*S*,11a*S*,11b*R*)-3-methoxy-9a,11b-dimethyl-9-oxohexadecahydro-5*H*-cyclopenta[1,2]phenanthro[8a,9-*b*]azirin-5-yl)pyridin-1-ium trifluoromethanesulfonate (**5z**) in $CDCl_3$ (500 MHz) at 23 °C.

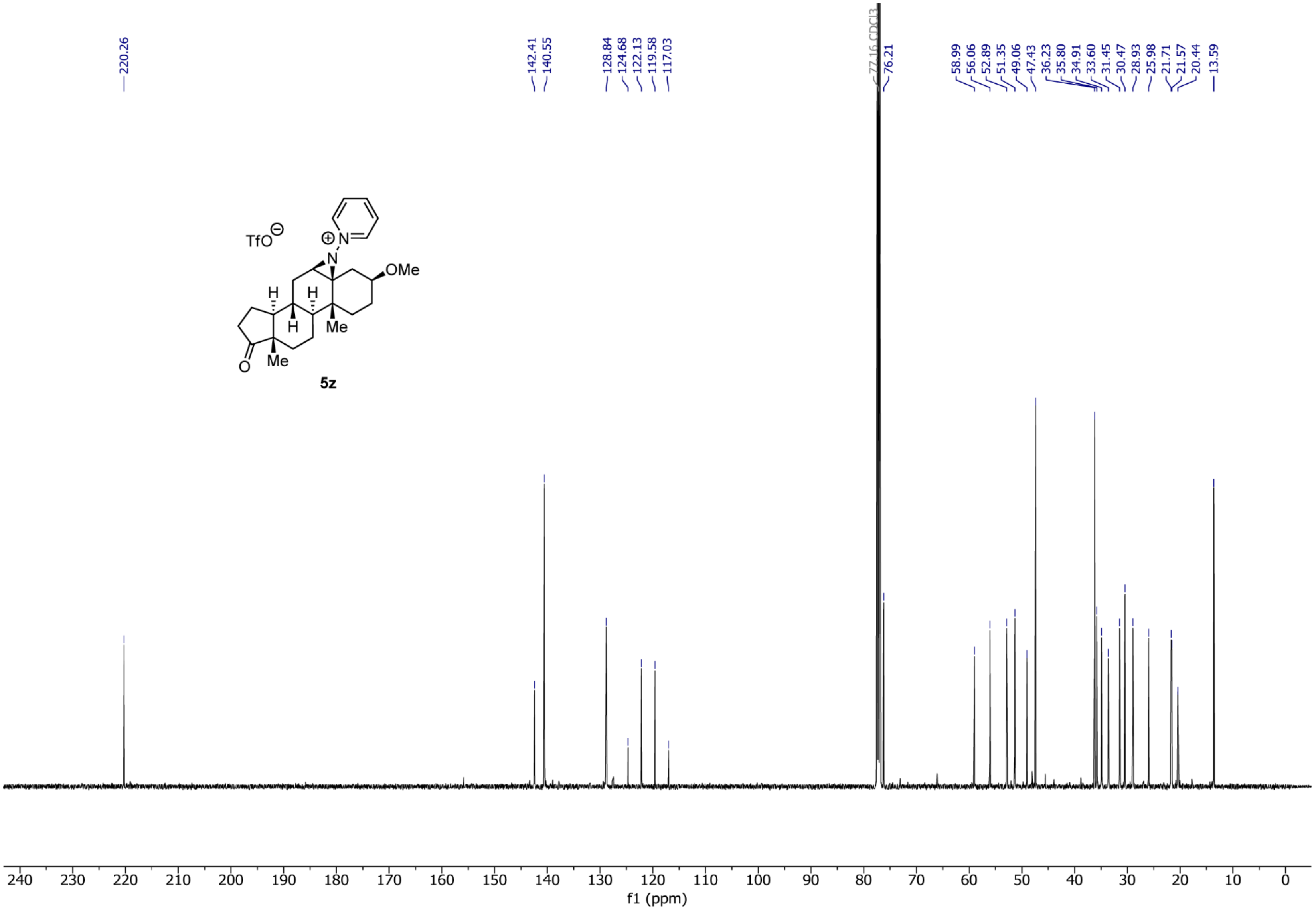

Figure S80. ^{13}C NMR spectrum of 1-((3*S*,4a*S*,5a*R*,6a*R*,6b*S*,9a*S*,11a*S*,11b*R*)-3-methoxy-9a,11b-dimethyl-9-oxohexadecahydro-5*H*-cyclopenta[1,2]phenanthro[8a,9-*b*]azirin-5-yl)pyridin-1-ium trifluoromethanesulfonate (**5z**) in $CDCl_3$ (126 MHz) at 23 °C.

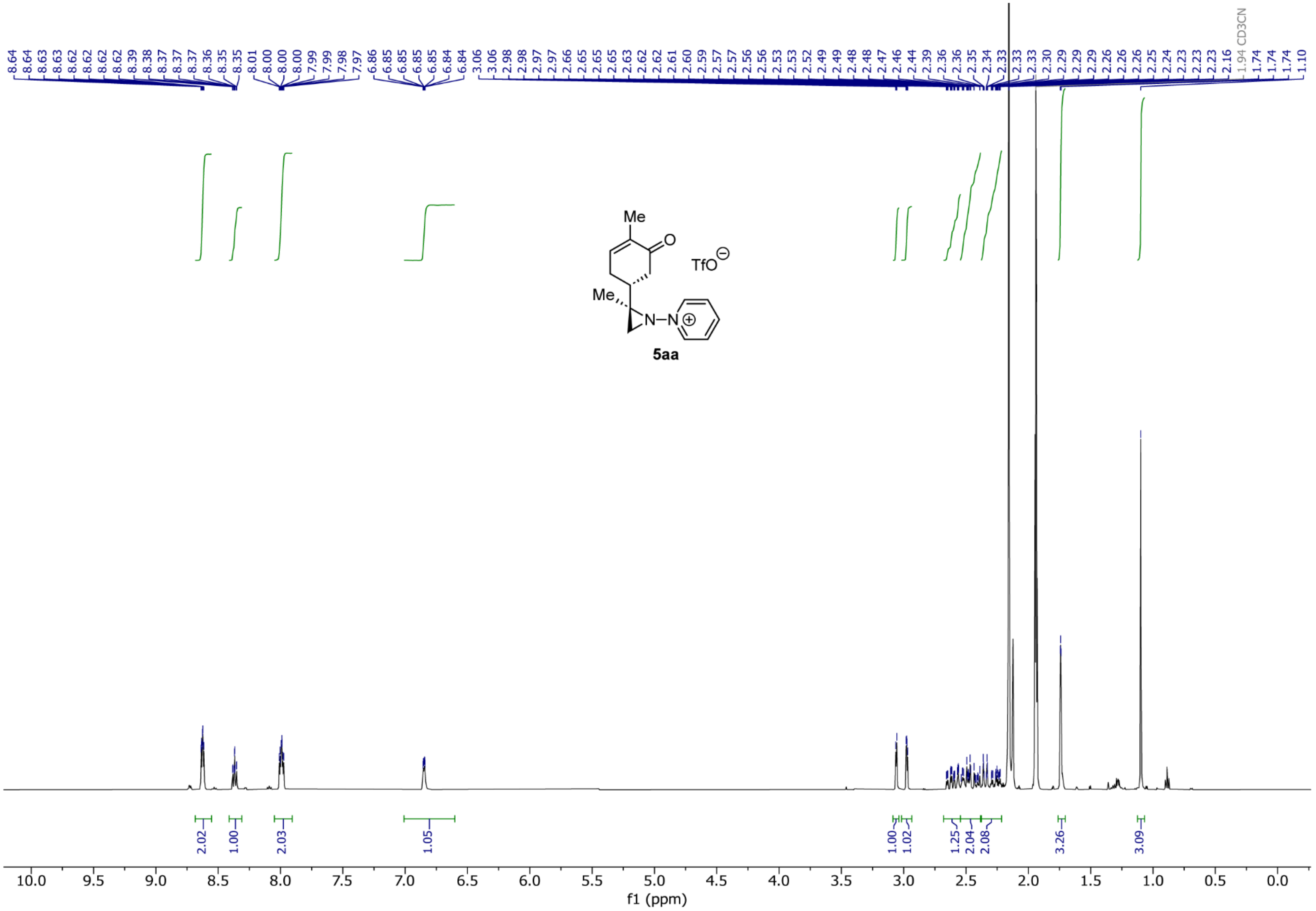

Figure S81. ^{1}H NMR spectrum of 1-((R)-2-methyl-2-((R)-4-methyl-5-oxocyclohex-3-en-1-yl)aziridin-1-yl)pyridin-1-ium trifluoromethanesulfonate (**5aa**) in CD_3CN (500 MHz) at 23 °C.

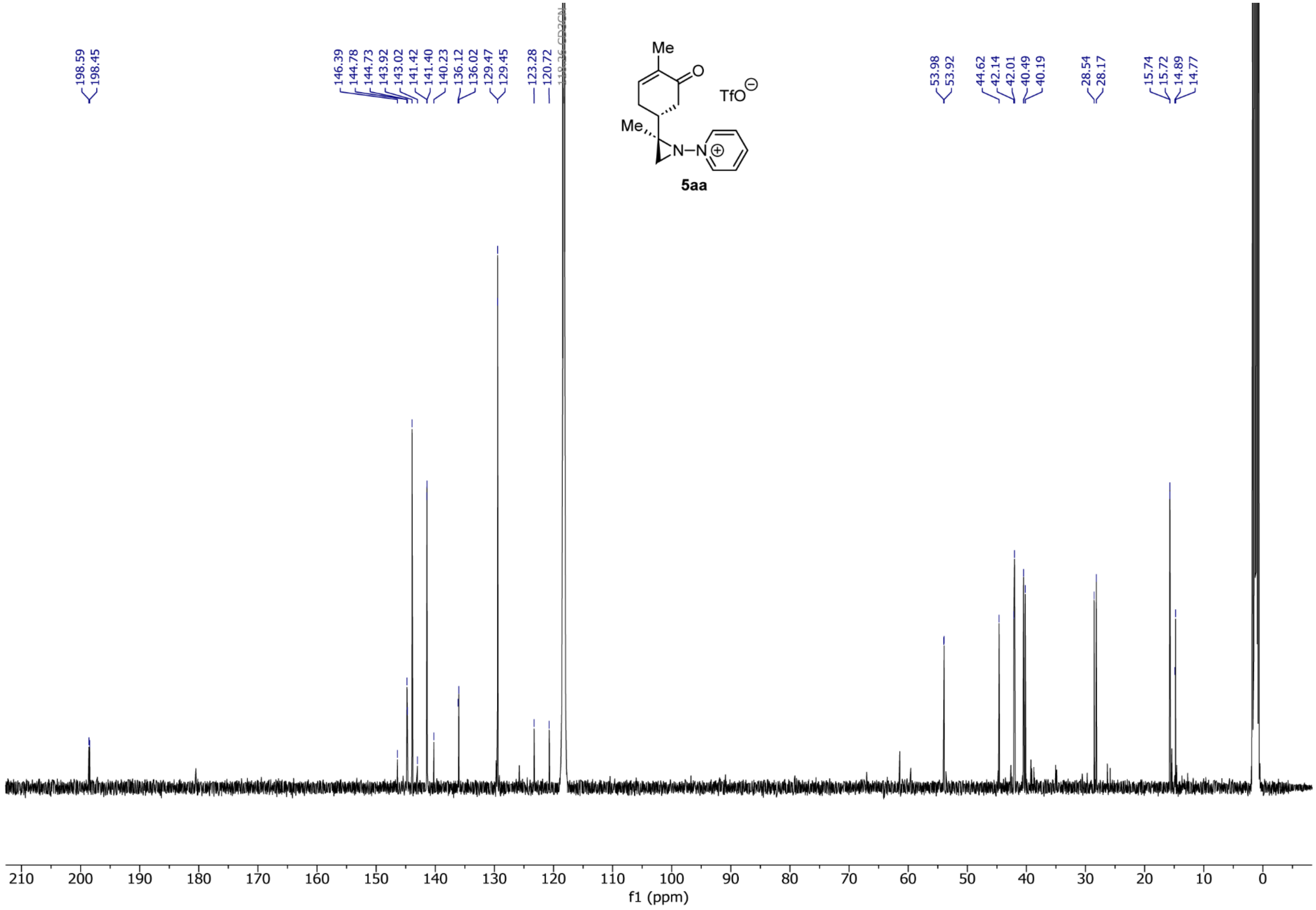

Figure S82. ^{13}C NMR spectrum of 1-((R)-2-methyl-2-((R)-4-methyl-5-oxocyclohex-3-en-1-yl)aziridin-1-yl)pyridin-1-ium trifluoromethanesulfonate (**5z**) in CD_3CN (126 MHz) at 23 °C.

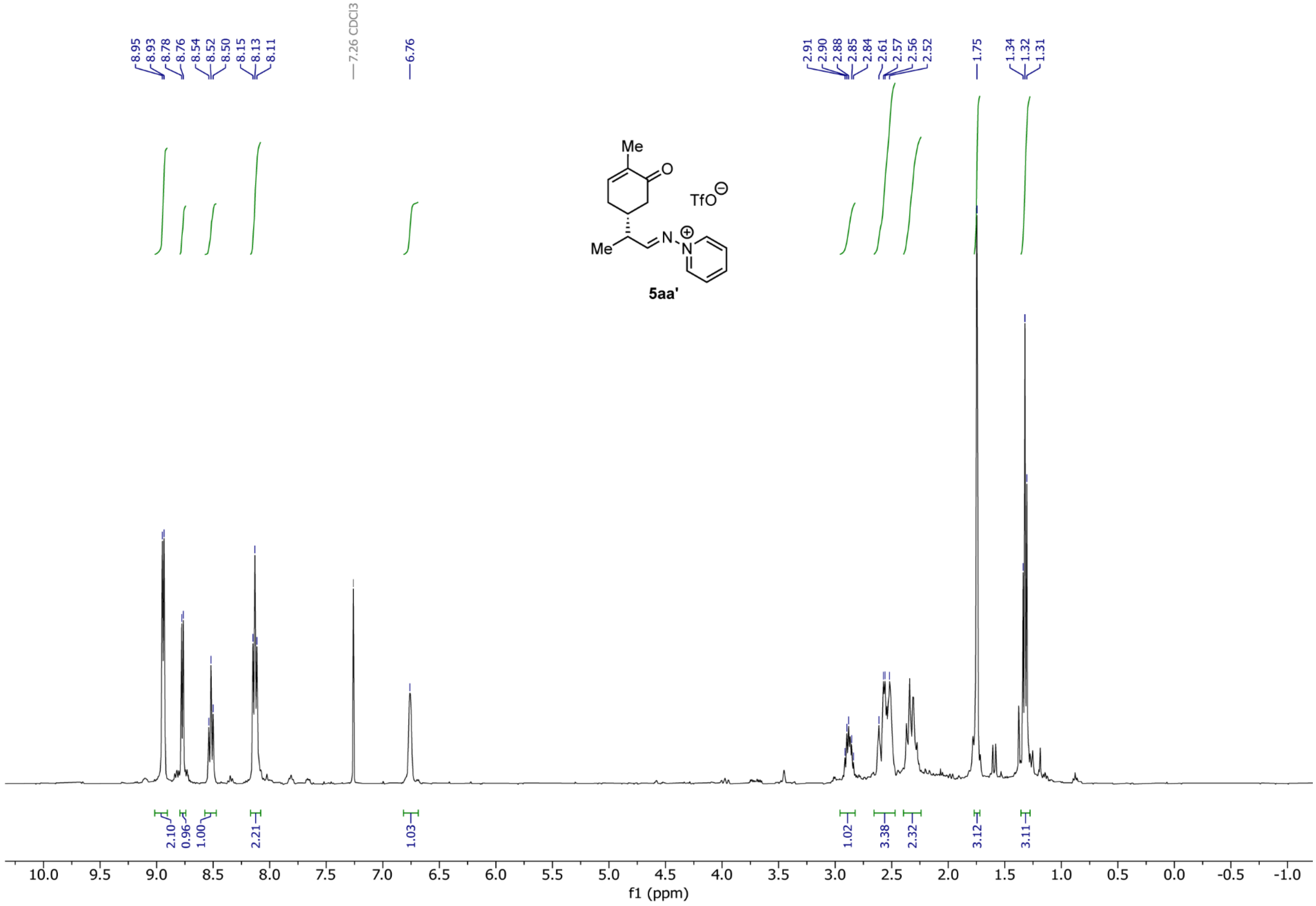

Figure S83. ^{1}H NMR spectrum of (*R*)-1-((2-(4-methyl-5-oxocyclohex-3-en-1-yl)propylidene)amino)pyridin-1-ium trifluoromethanesulfonate (**5aa'**) in $CDCl_3$ (400 MHz) at 23 °C.

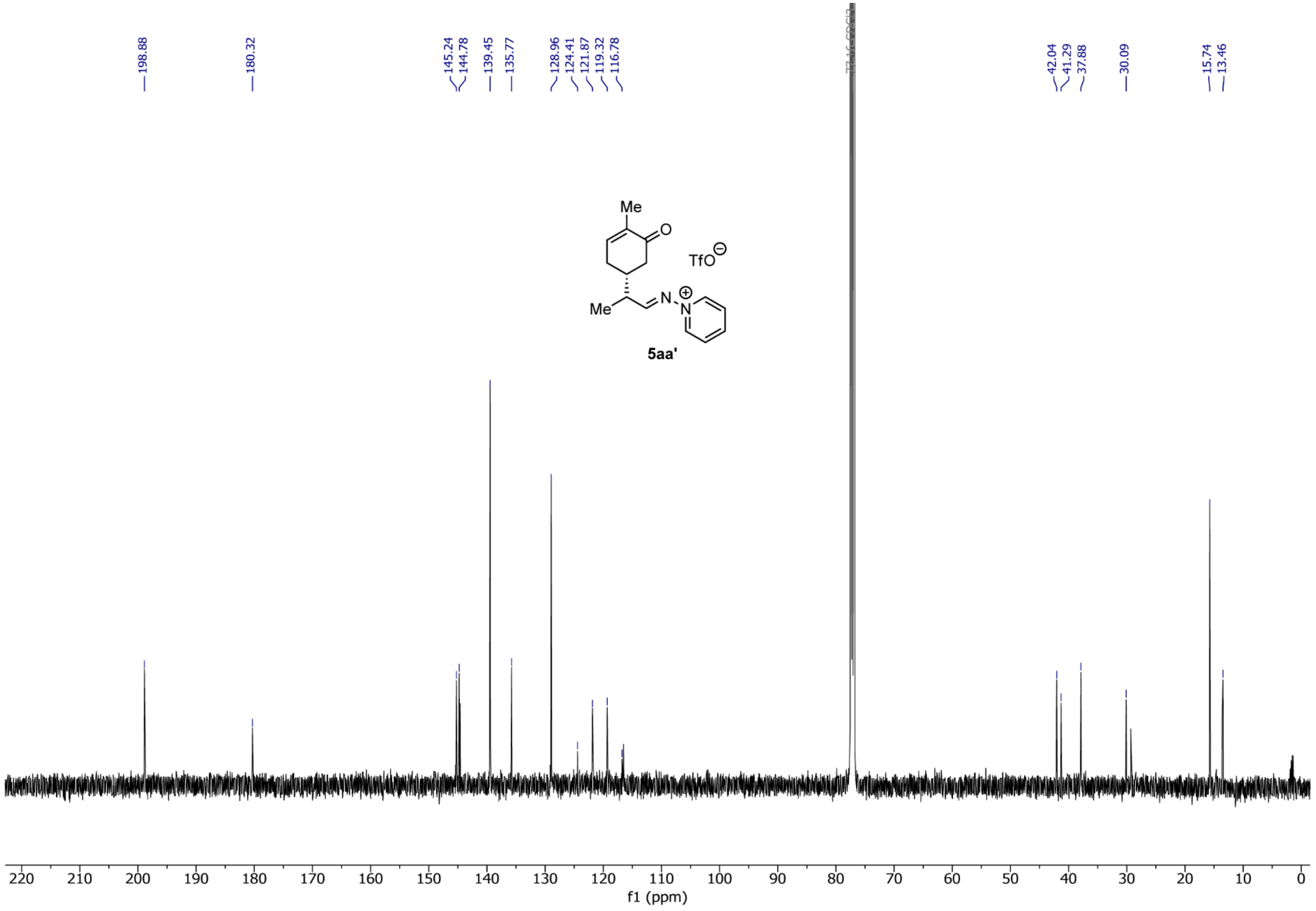

Figure S84. ^{13}C NMR spectrum of (*R*)-1-((2-(4-methyl-5-oxocyclohex-3-en-1-yl)propylidene)amino)pyridin-1-ium trifluoromethanesulfonate (**5aa'**) in $CDCl_3$ (126 MHz) at 23 °C.

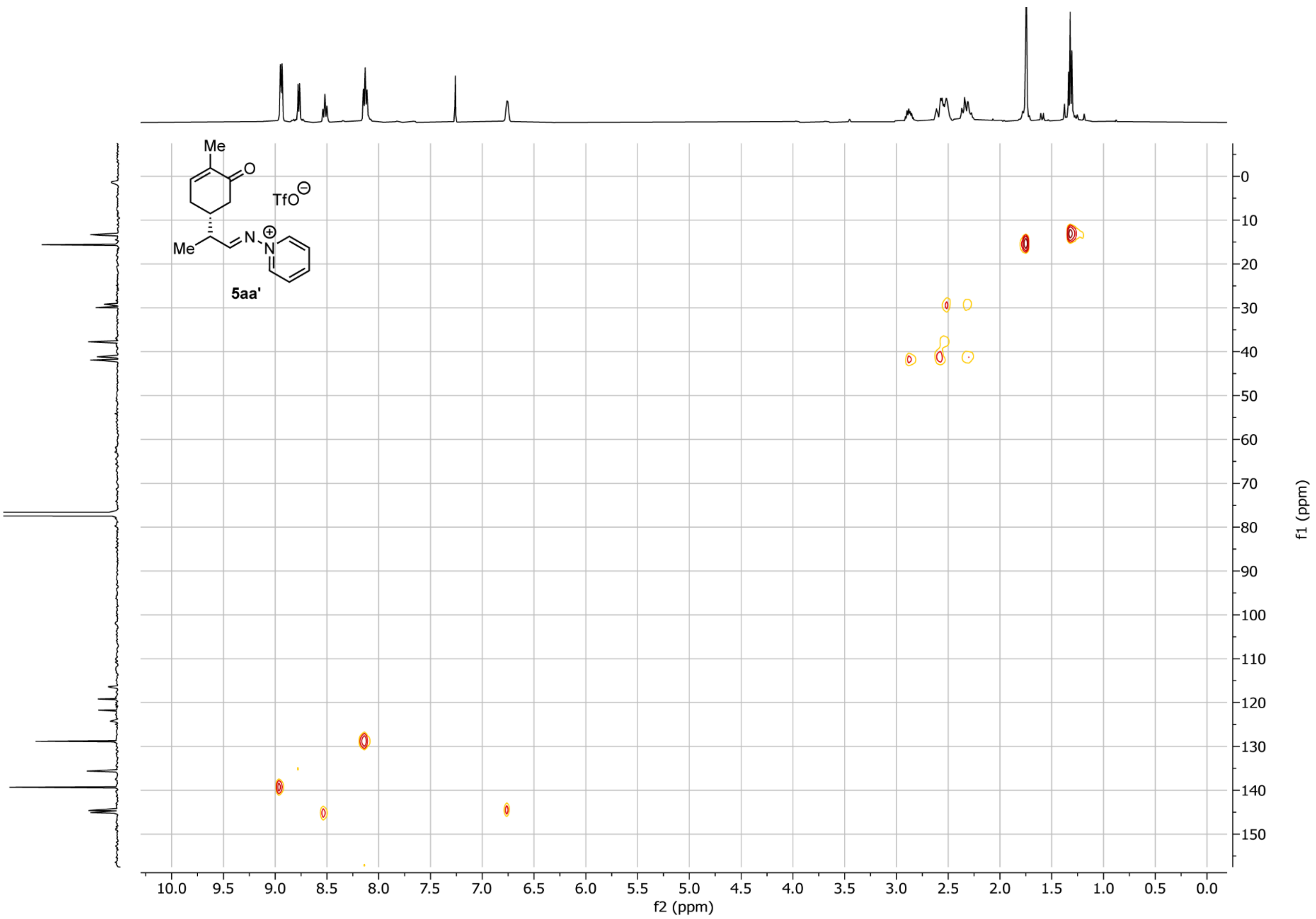

Figure S85. HSQC NMR spectrum of (*R*)-1-((2-(4-methyl-5-oxocyclohex-3-en-1-yl)propylidene)amino)pyridin-1-ium trifluoromethanesulfonate (**5aa'**) in $CDCl_3$ at 23 °C.

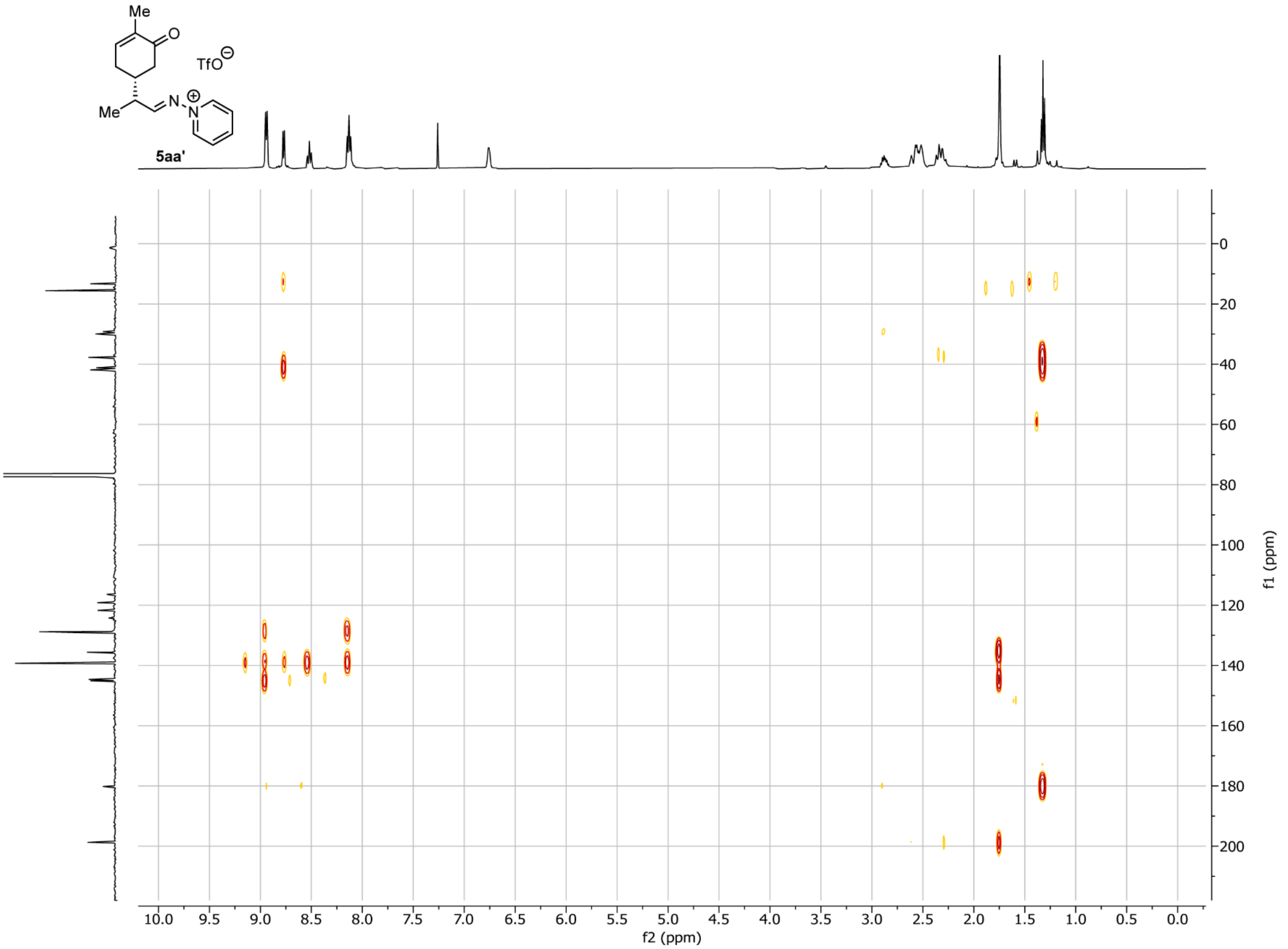

Figure S86. HMBC NMR spectrum of (*R*)-1-((2-(4-methyl-5-oxocyclohex-3-en-1-yl)propylidene)amino)pyridin-1-ium trifluoromethanesulfonate (**5aa'**) in $CDCl_3$ at 23 °C.

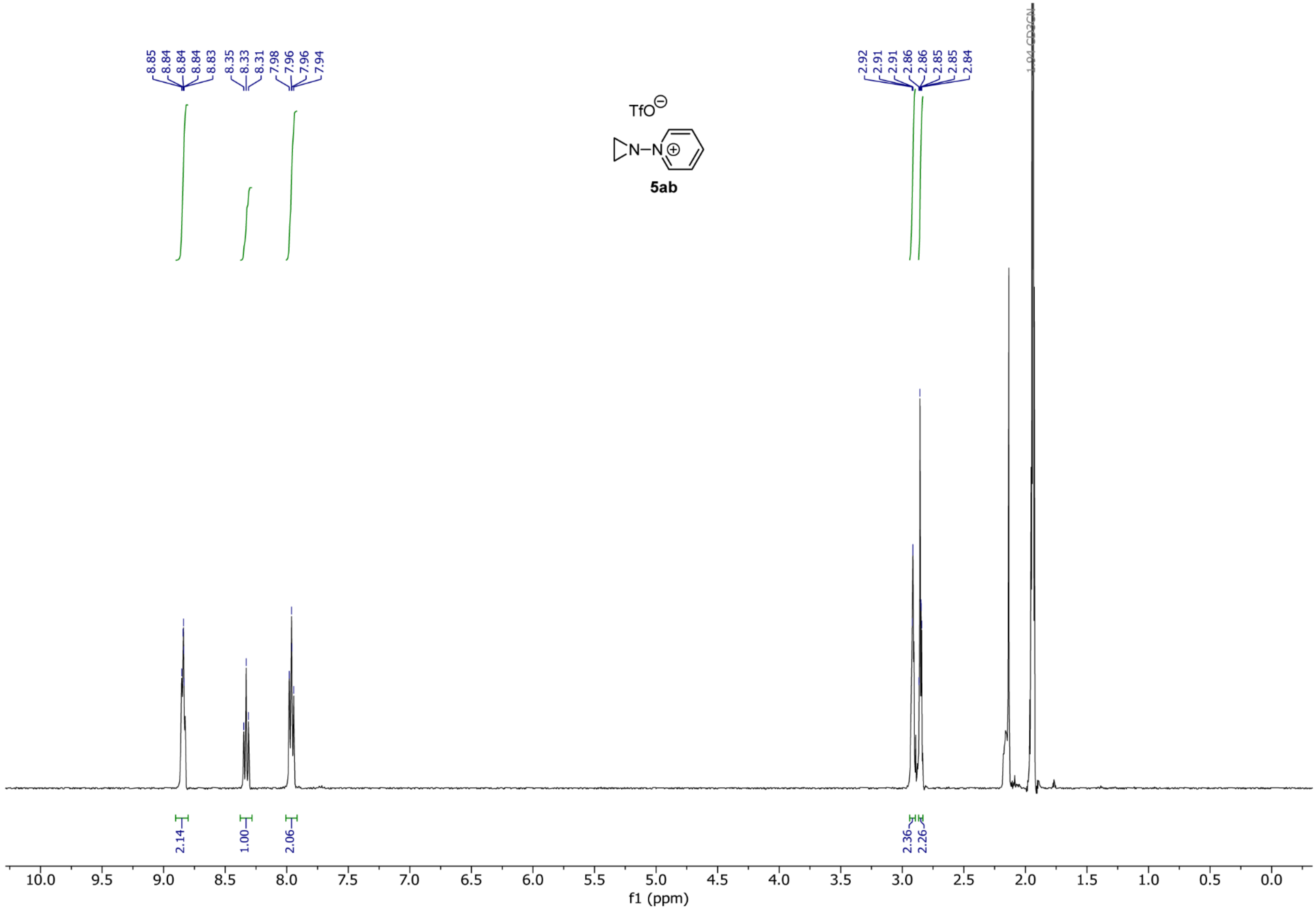

Figure S87. ^{1}H NMR spectrum of 1-(aziridin-1-yl)pyridin-1-ium trifluoromethanesulfonate (**5ab**) in CD_3CN (400 MHz) at 23 °C.

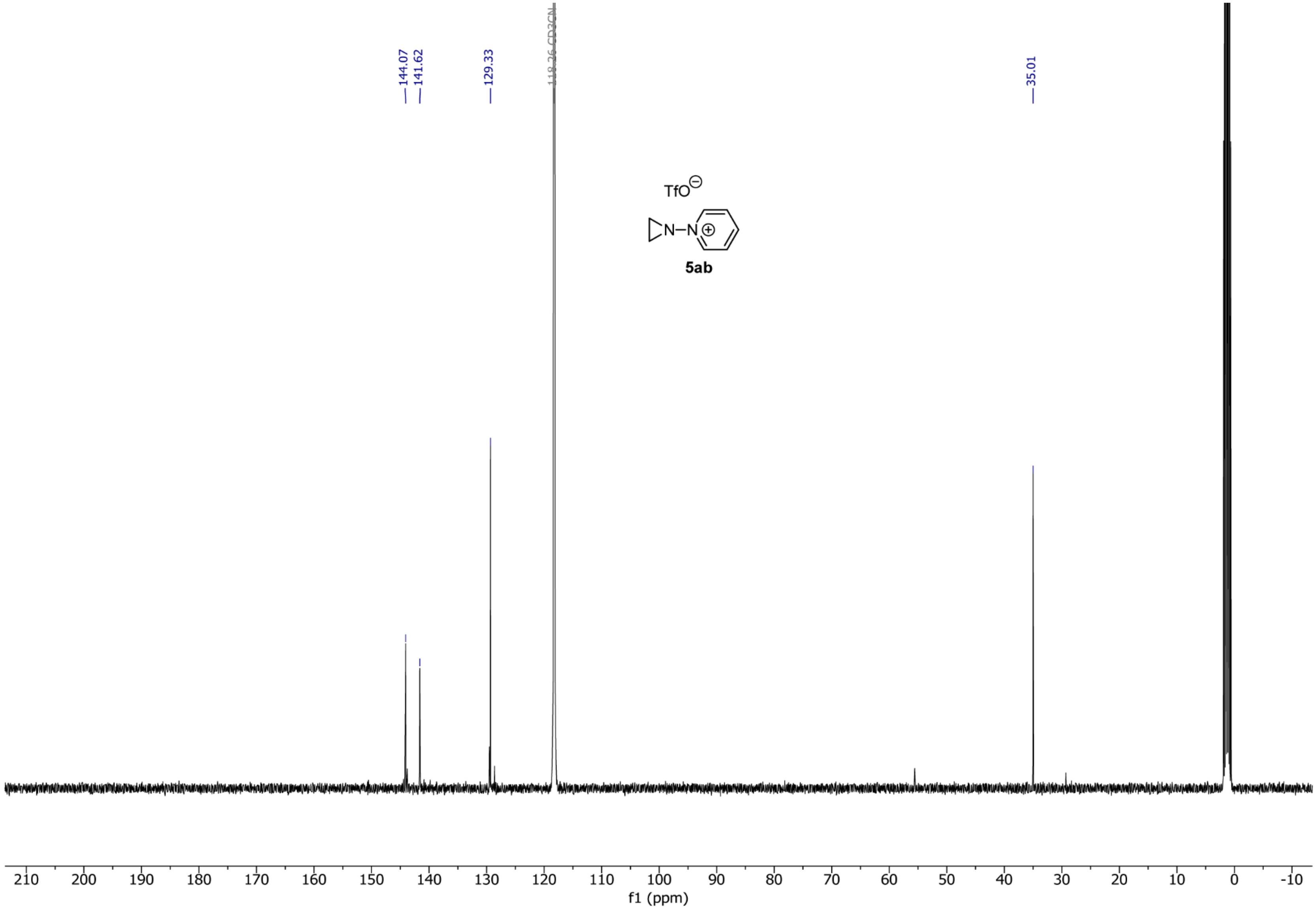

Figure S88. ^{13}C NMR spectrum of 1-(aziridin-1-yl)pyridin-1-ium trifluoromethanesulfonate (**5ab**) in CD_3CN (101 MHz) at 23 °C.

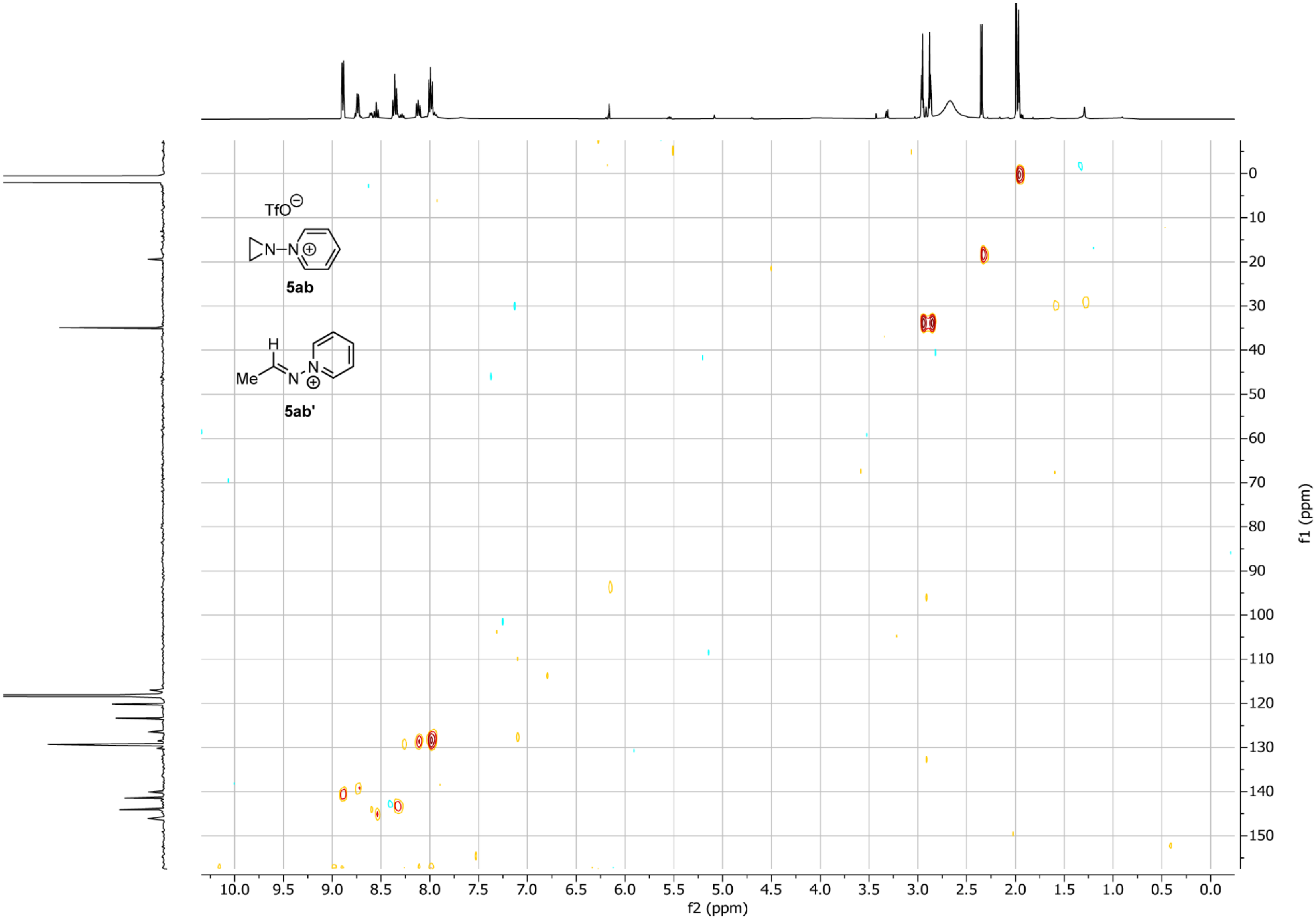

Figure S89. HSQC NMR spectrum of 1-(aziridin-1-yl)pyridin-1-ium trifluoromethanesulfonate (**5ab**) in CD_3CN at 23 °C.

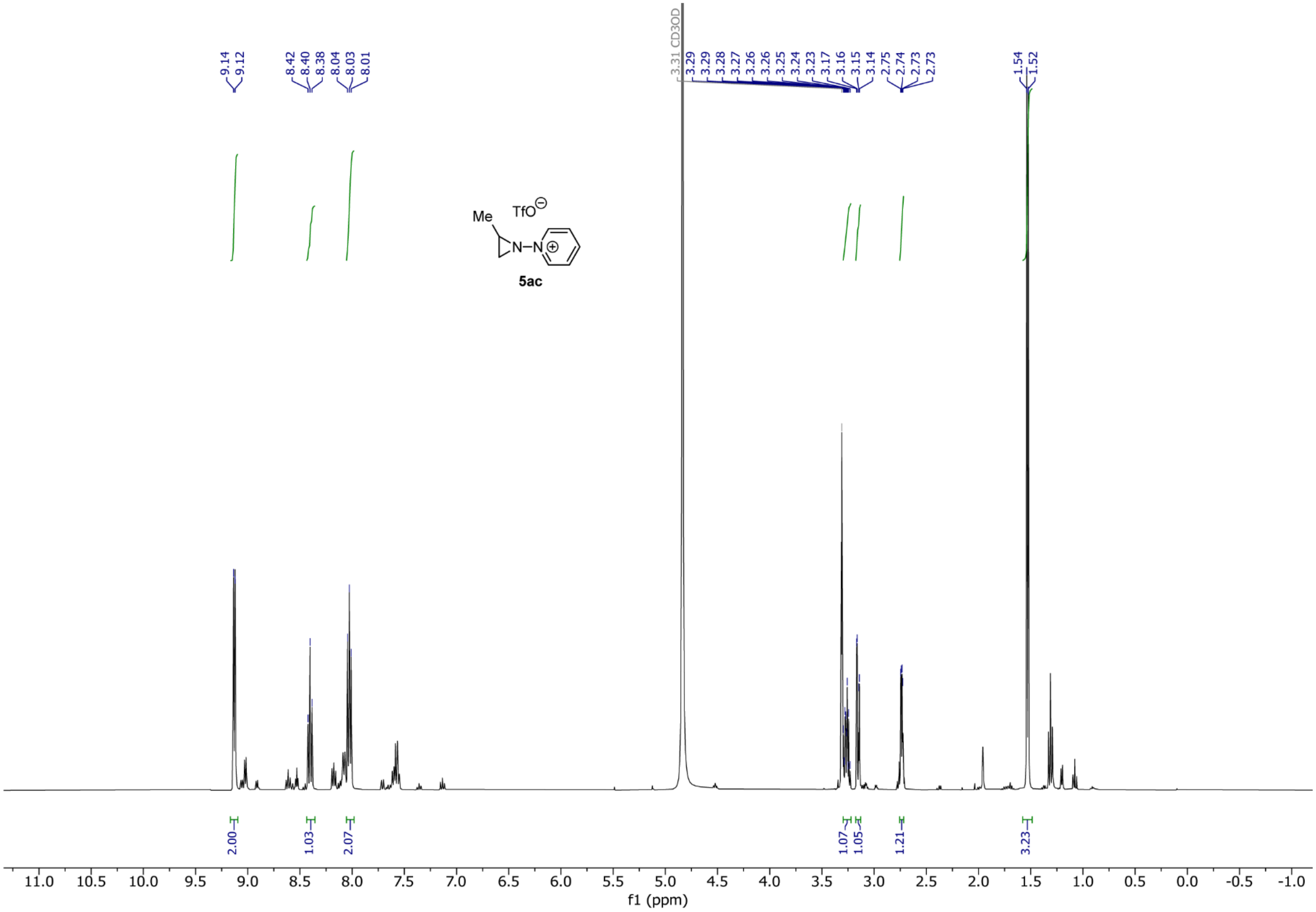

Figure S90. ^{1}H NMR spectrum of 1-(2-methylaziridin-1-yl)pyridin-1-ium trifluoromethanesulfonate (**5ac**) in CD_3OD (400 MHz) at 23 °C. Extra peaks are due to coelution of the imine byproduct.

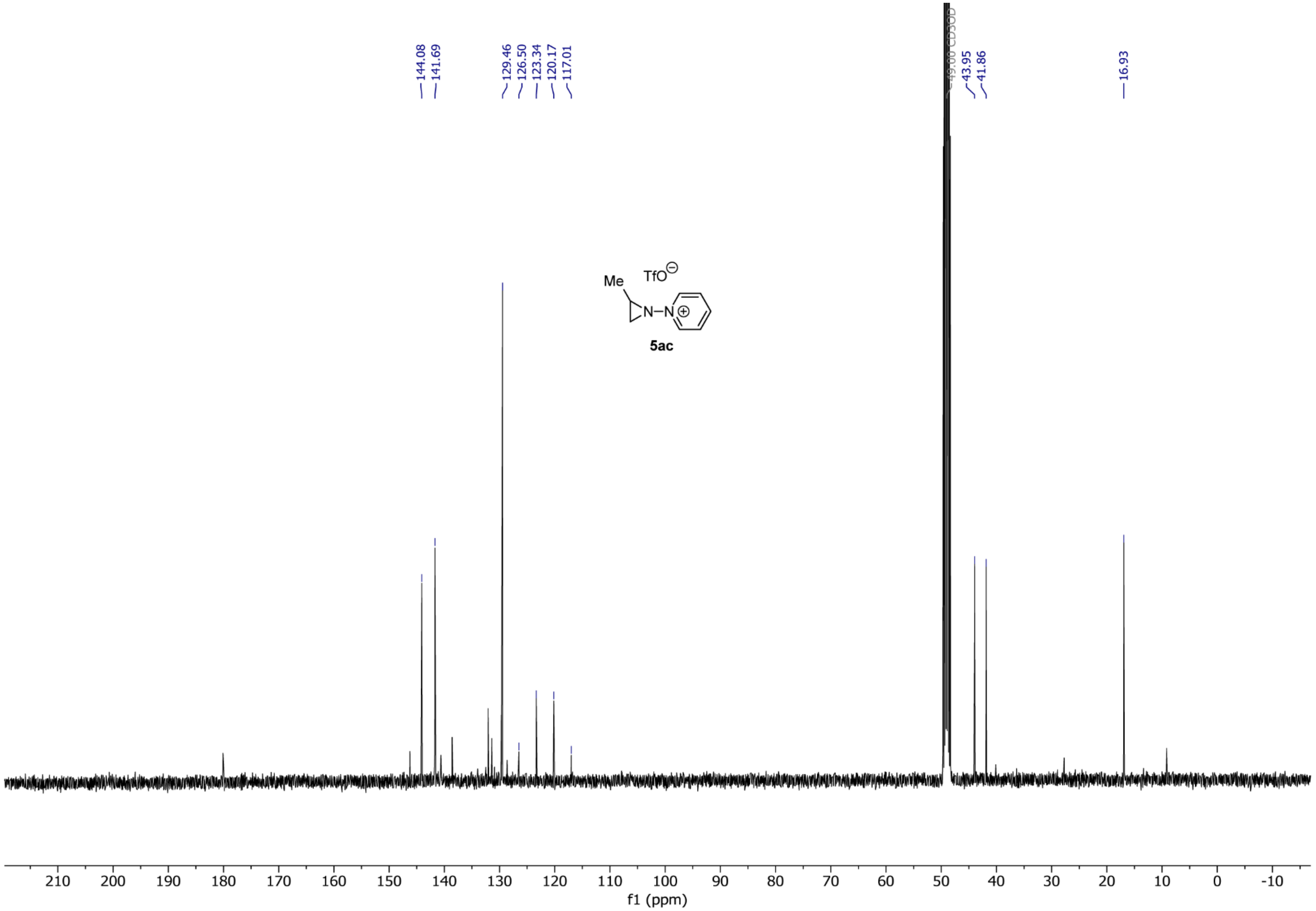

Figure S91. ^{13}C NMR spectrum of 1-(2-methylaziridin-1-yl)pyridin-1-ium trifluoromethanesulfonate (**5ac**) in CD_3OD (101 MHz) at 23 °C.

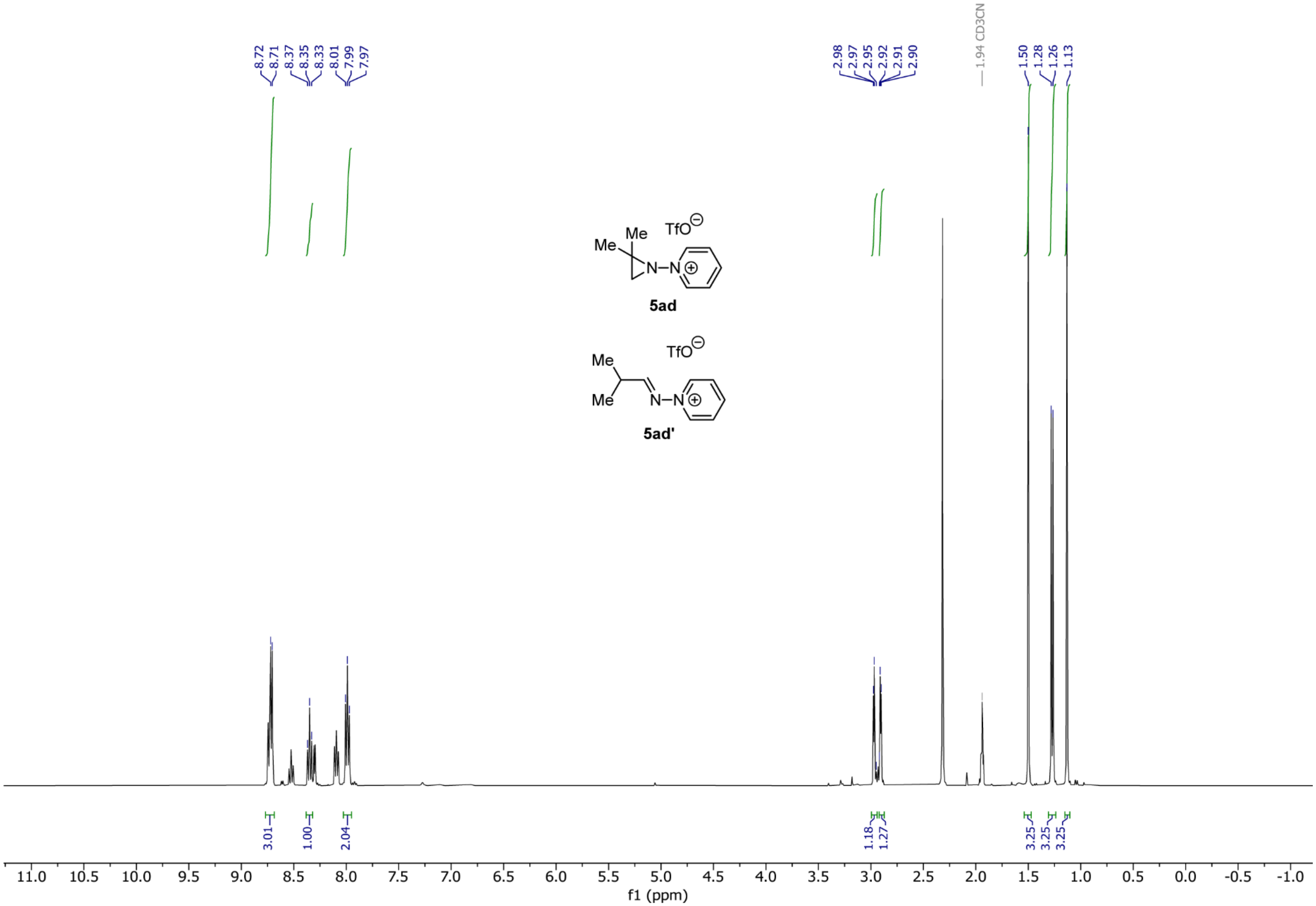

Figure S92. ^{1}H NMR spectrum of 1-(2,2-dimethylaziridin-1-yl)pyridin-1-ium trifluoromethanesulfonate (**5ad**) in CD_3CN (400 MHz) at 23 °C. Extra peaks are attributed to imine byproduct **5ad'**.

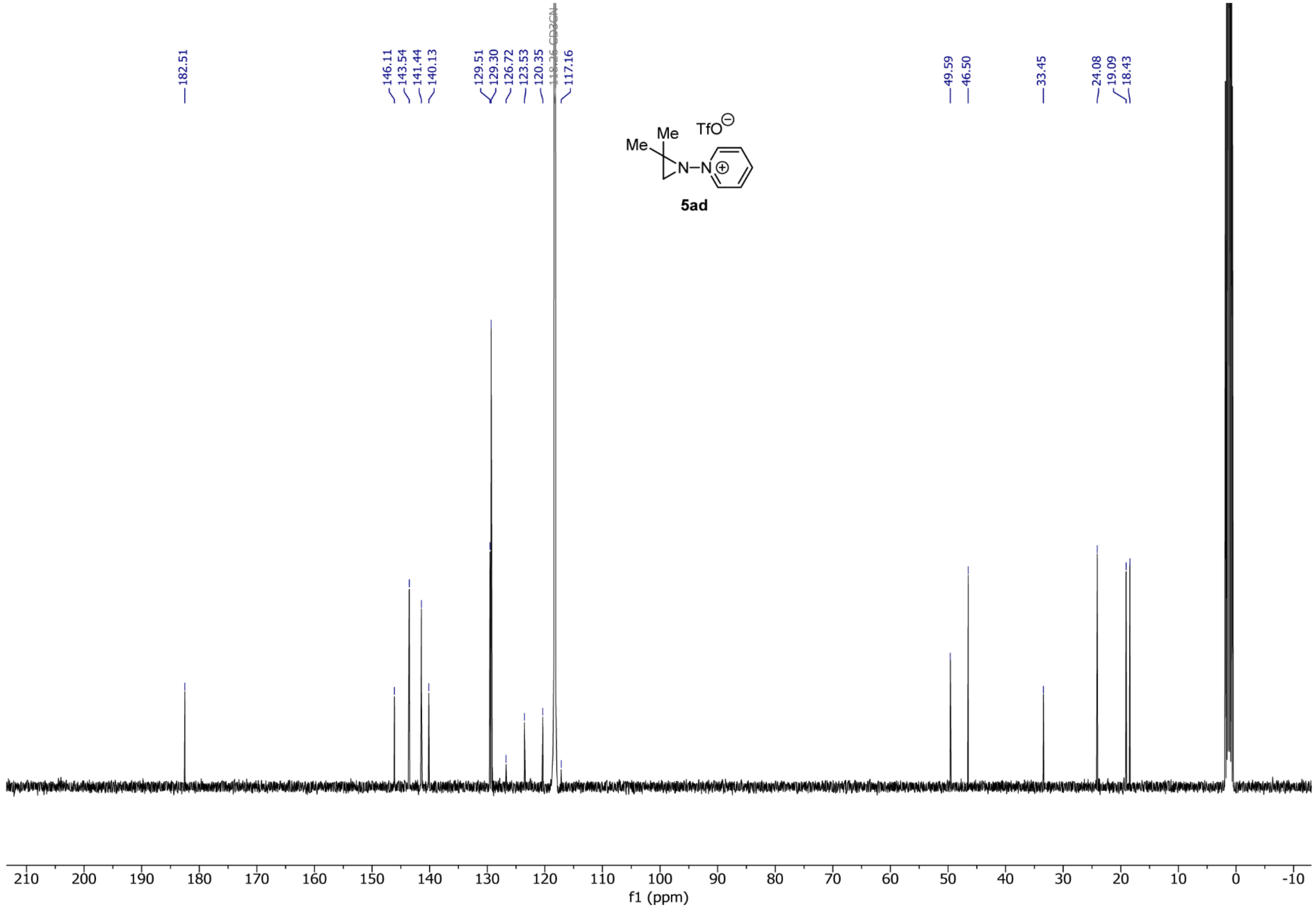

Figure S93. ^{13}C NMR spectrum of 1-(2,2-dimethylaziridin-1-yl)pyridin-1-ium trifluoromethanesulfonate (**5ad**) in CD_3CN (101 MHz) at 23 °C.

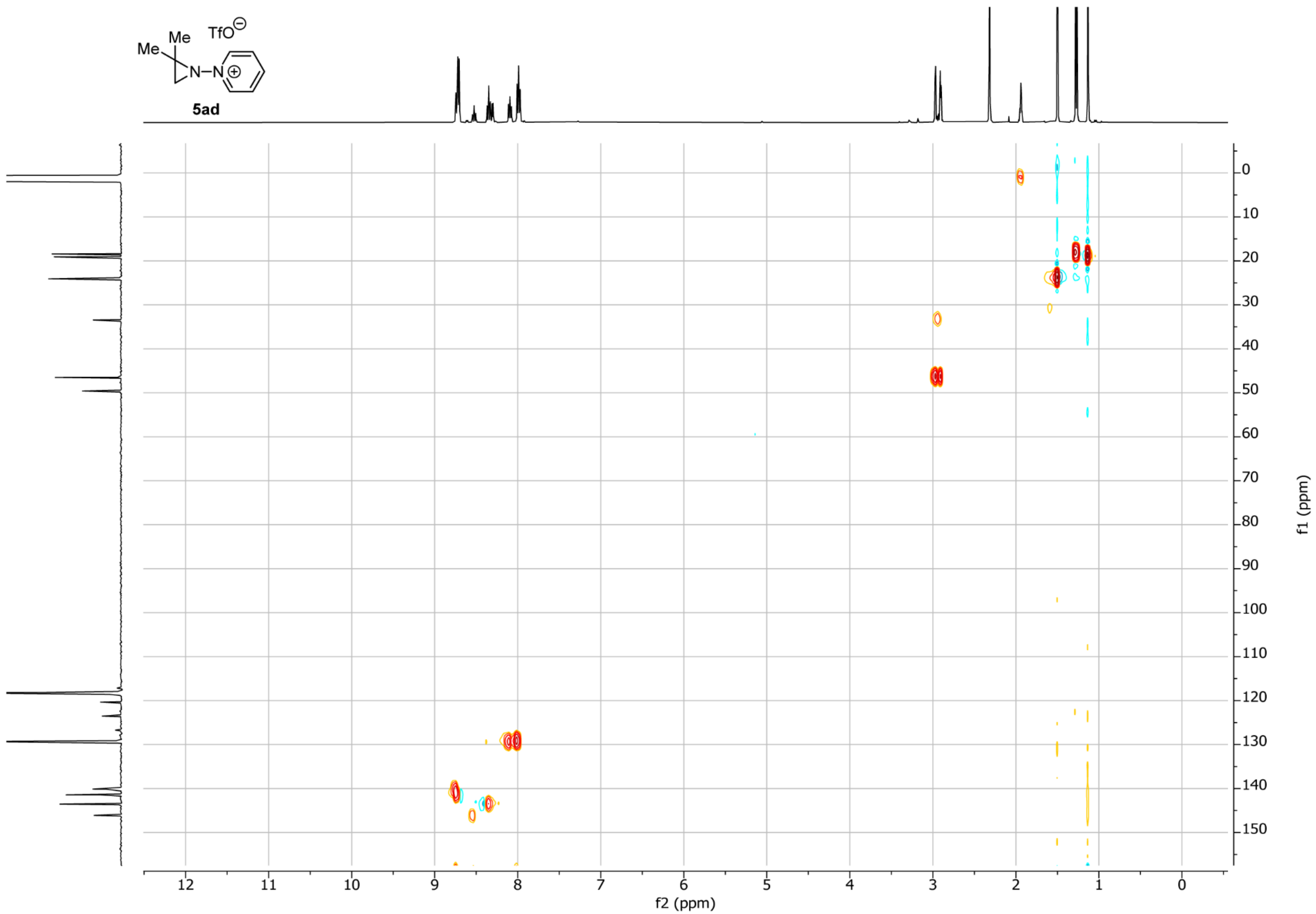

Figure S94. HSQC NMR spectrum of 1-(2,2-dimethylaziridin-1-yl)pyridin-1-ium trifluoromethanesulfonate (**5ad**) in CD_3CN at 23 °C.

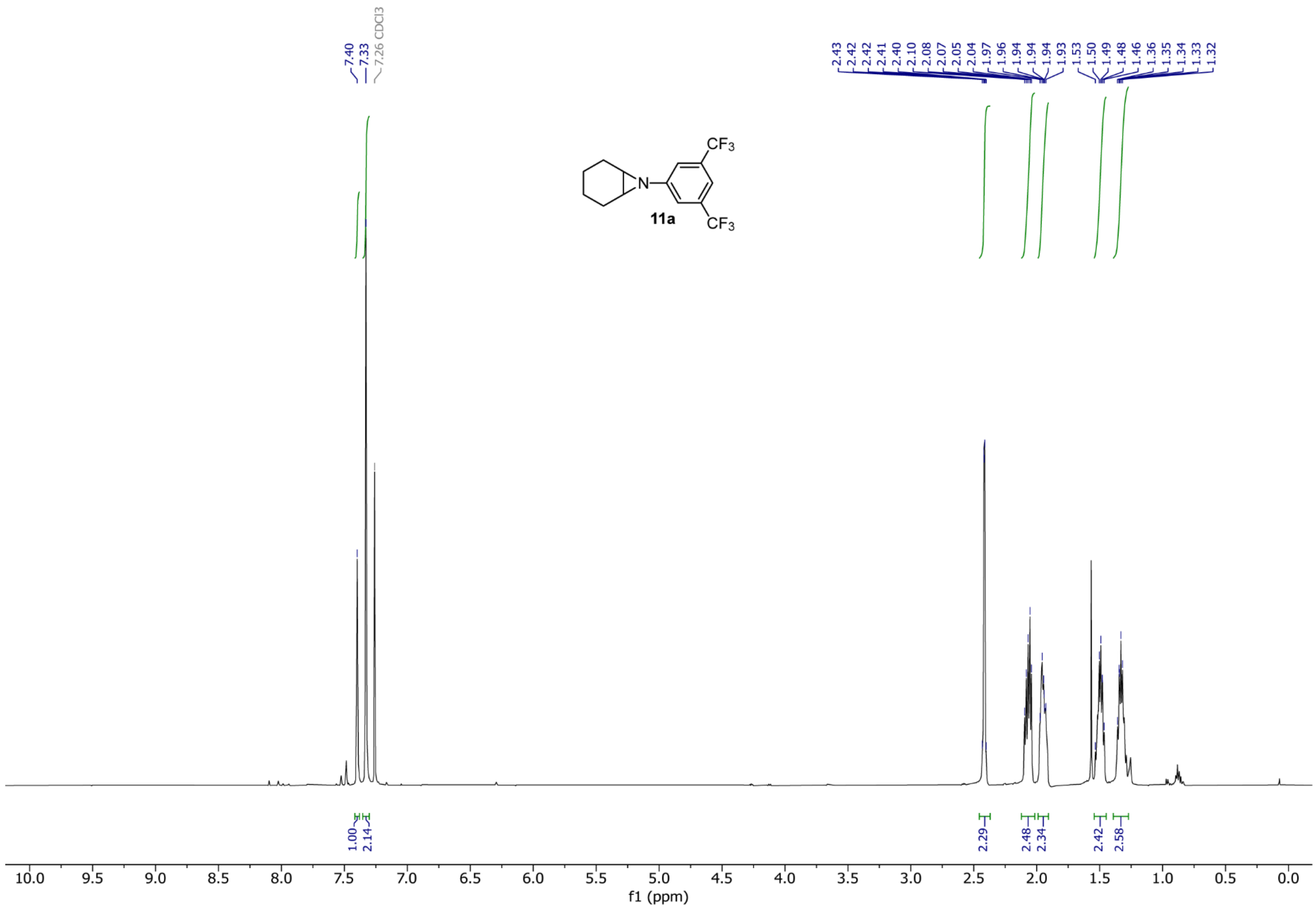

Figure S95. ^{1}H NMR spectrum of 7-(3,5-bis(trifluoromethyl)phenyl)-7-azabicyclo[4.1.0]heptane (**11a**) in $CDCl_3$ (499 MHz) at 23 °C.

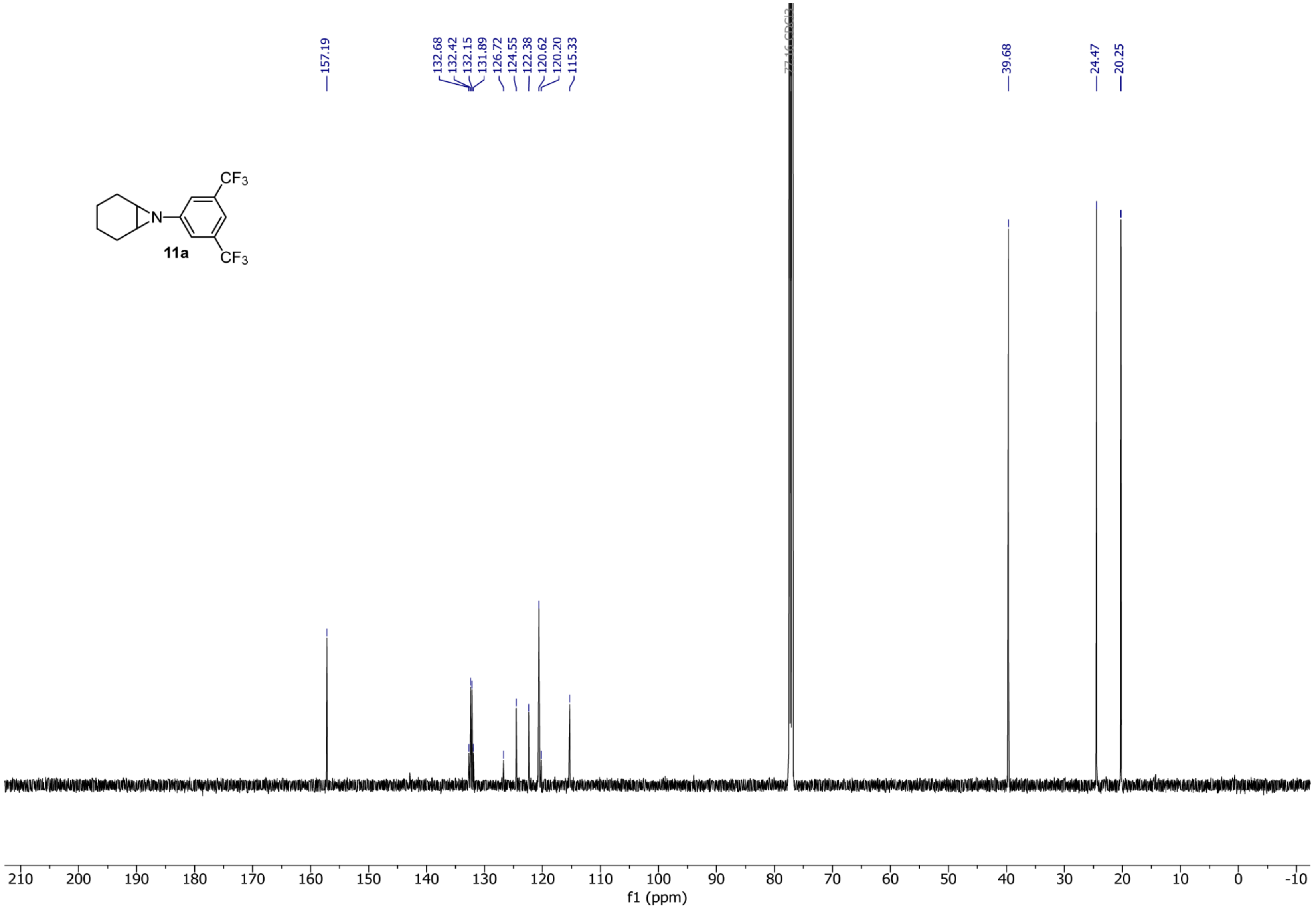

Figure S96. ^{13}C NMR spectrum of 7-(3,5-bis(trifluoromethyl)phenyl)-7-azabicyclo[4.1.0]heptane (**11a**) in $CDCl_3$ (126 MHz) at 23 °C.

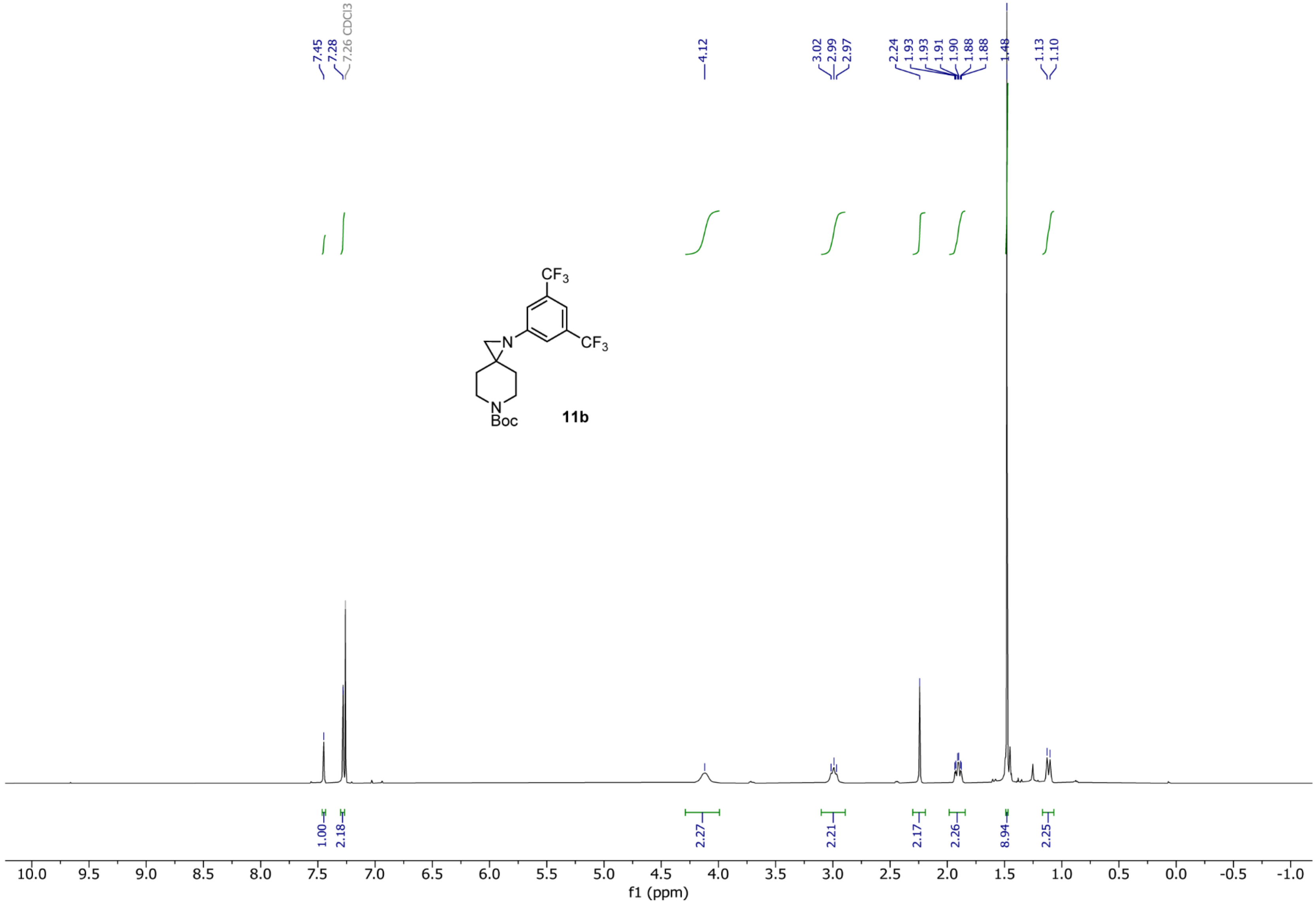

Figure S97. ^{1}H NMR spectrum of *tert*-butyl 1-(3,5-bis(trifluoromethyl)phenyl)-1,6-diazaspiro[2.5]octane-6-carboxylate (**11b**) in $CDCl_3$ (500 MHz) at 23 °C.

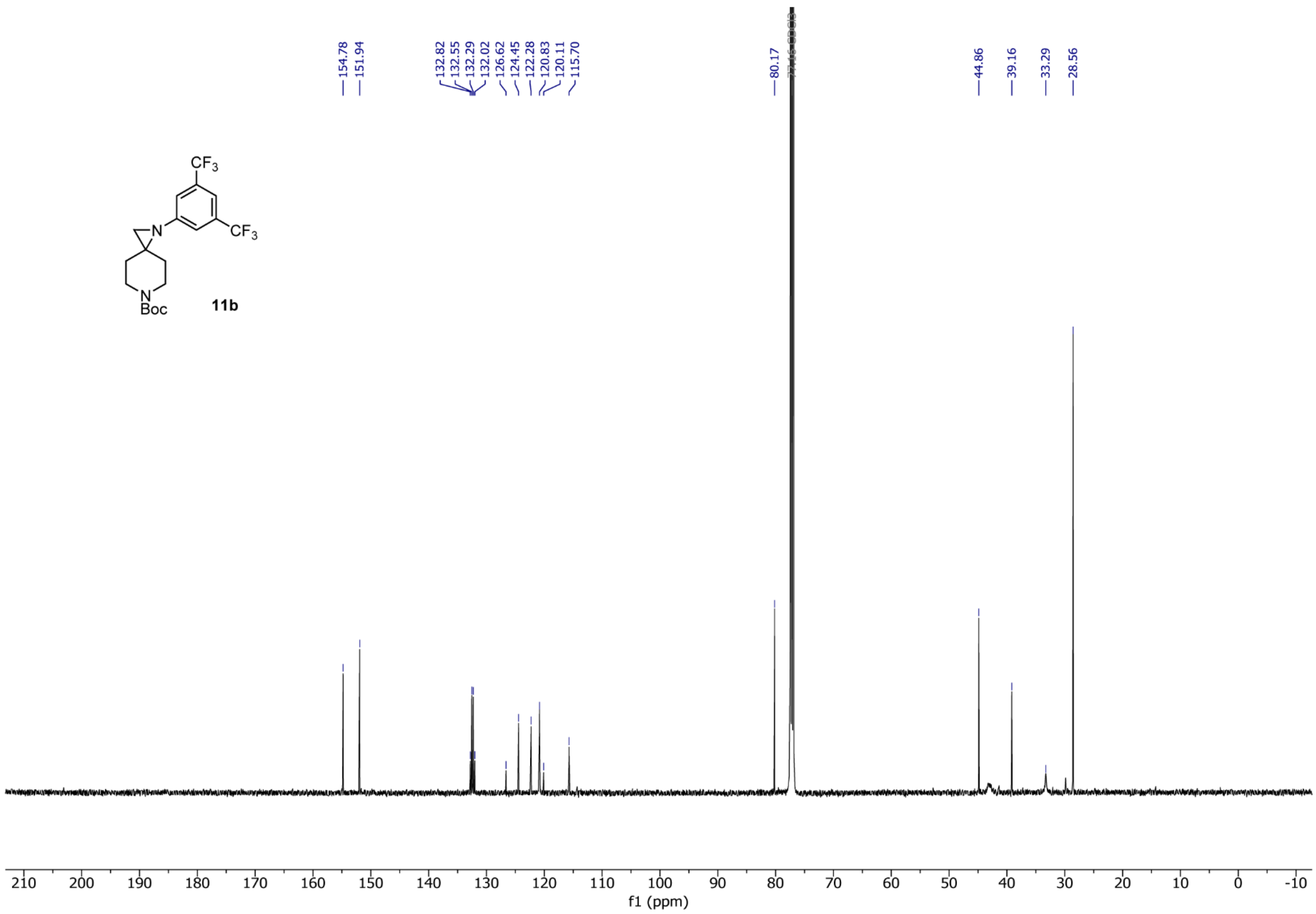

Figure S98. ^{13}C NMR spectrum of *tert*-butyl 1-(3,5-bis(trifluoromethyl)phenyl)-1,6-diazaspiro[2.5]octane-6-carboxylate (**11b**) in $CDCl_3$ (126 MHz) at 23 °C.

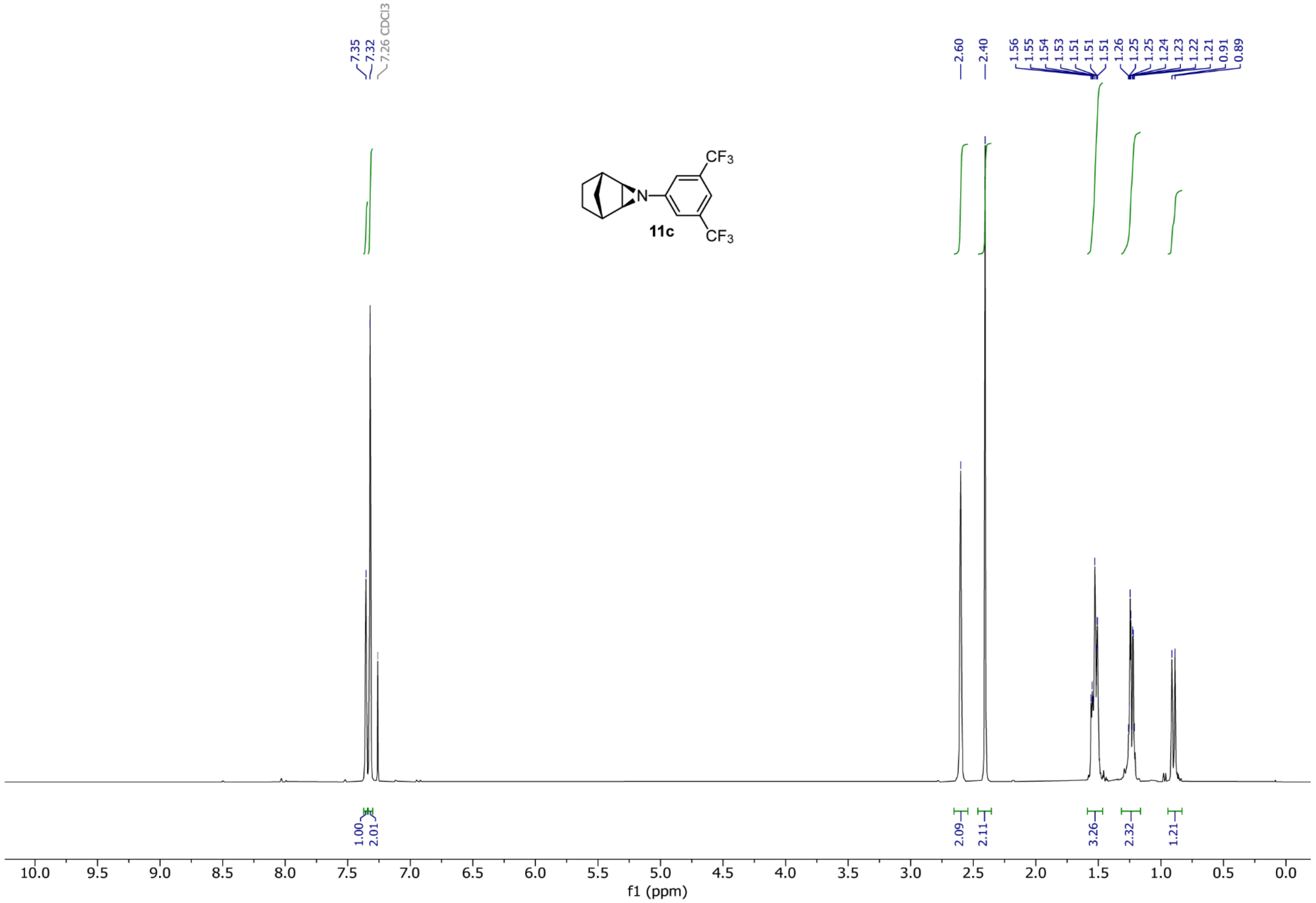

Figure S99. ^{1}H NMR spectrum of *exo*-3-(3,5-bis(trifluoromethyl)phenyl)-3-azatricyclo[3.2.1.0^{2,4}]octane (**11c**) in $CDCl_3$ (400 MHz) at 23 °C.

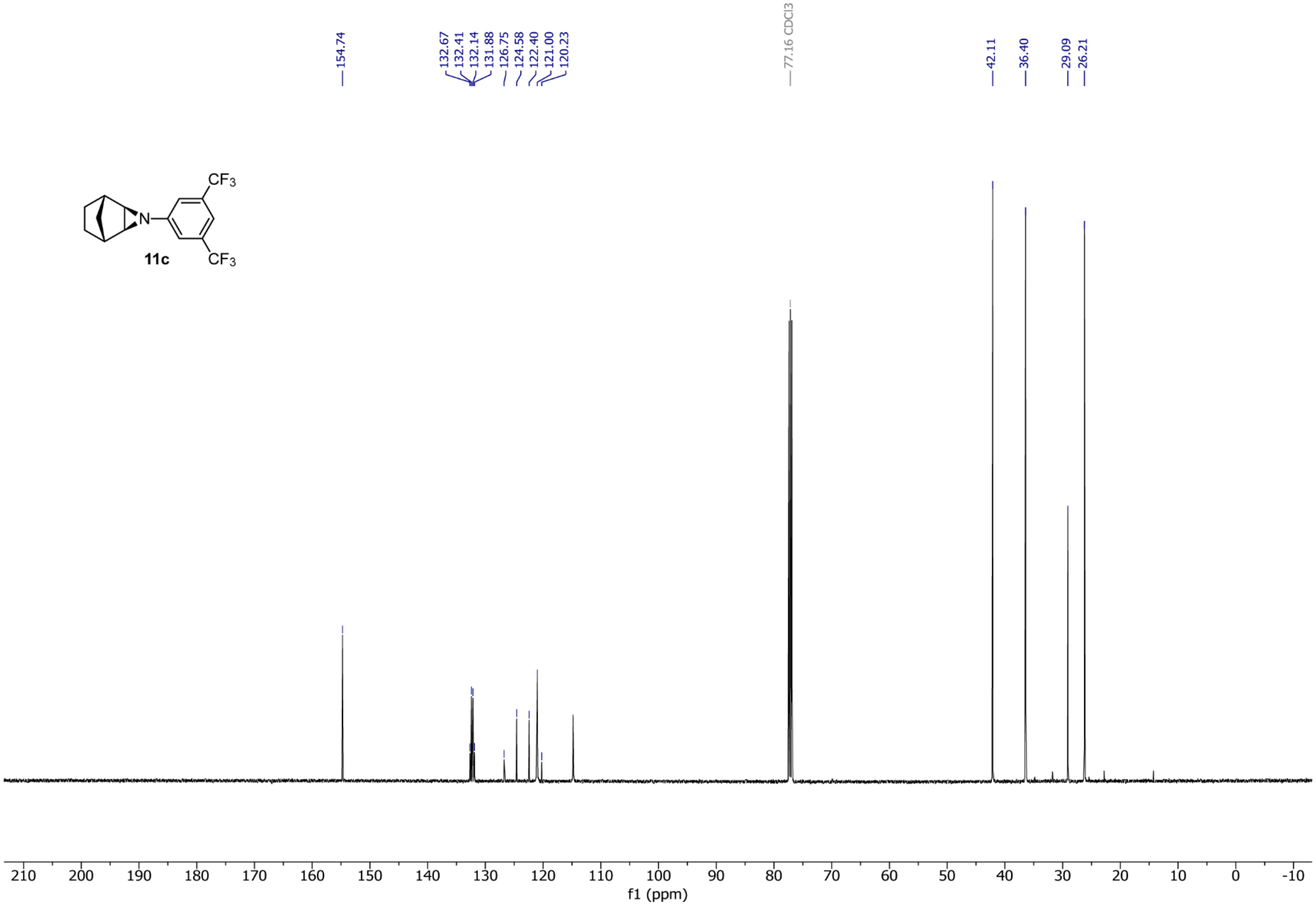

Figure S100. ^{13}C NMR spectrum of *exo*-3-(3,5-bis(trifluoromethyl)phenyl)-3-azatricyclo[3.2.1.0^{2,4}]octane (**11c**) in $CDCl_3$ (101 MHz) at 23 °C.

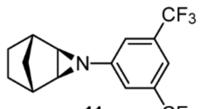

11c

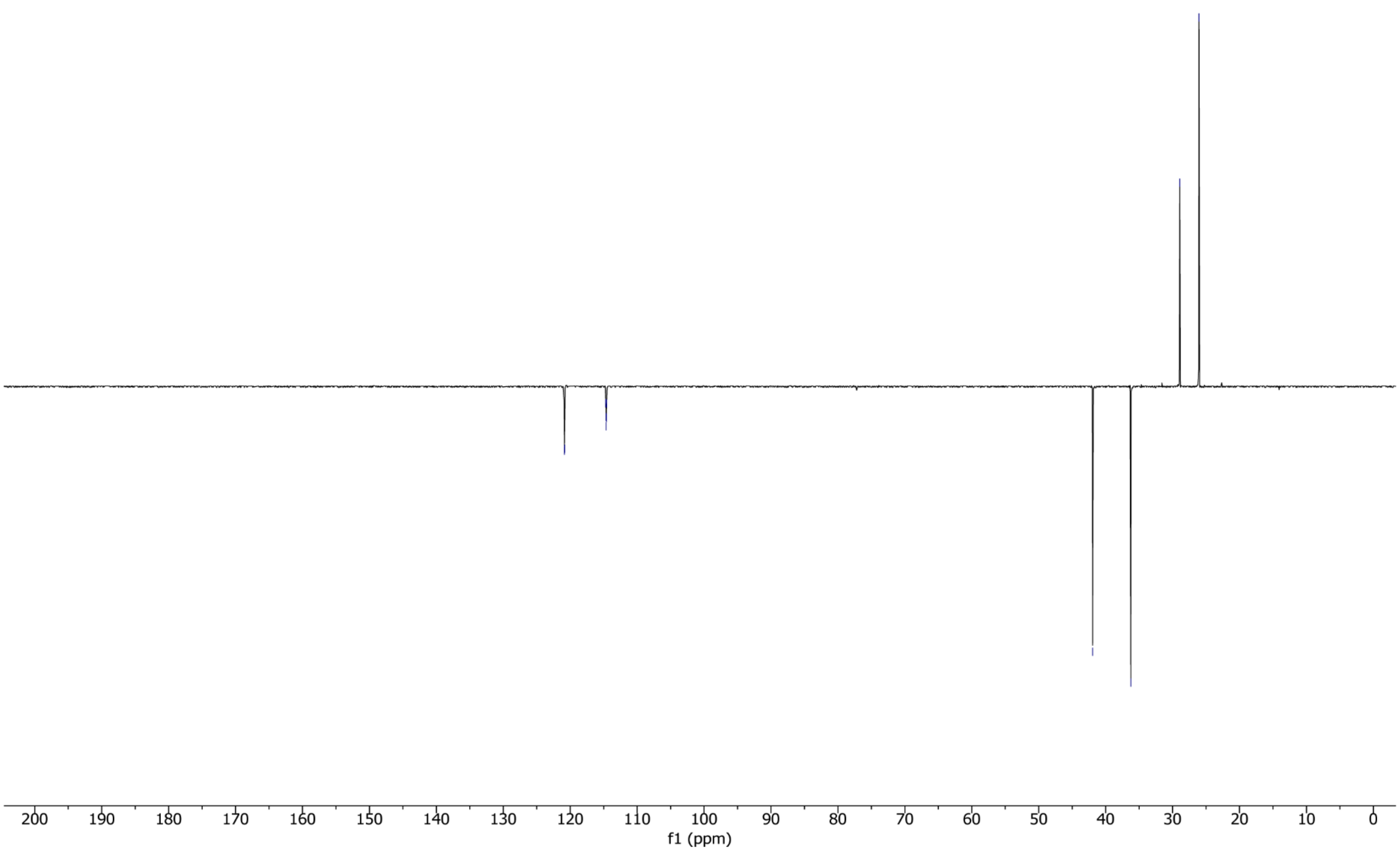

Figure S101. ^{13}C DEPT 135° NMR spectrum of *exo*-3-(3,5-bis(trifluoromethyl)phenyl)-3-azatricyclo[3.2.1.0^{2,4}]octane (**11c**) in $CDCl_3$ (101 MHz) at 23 °C.

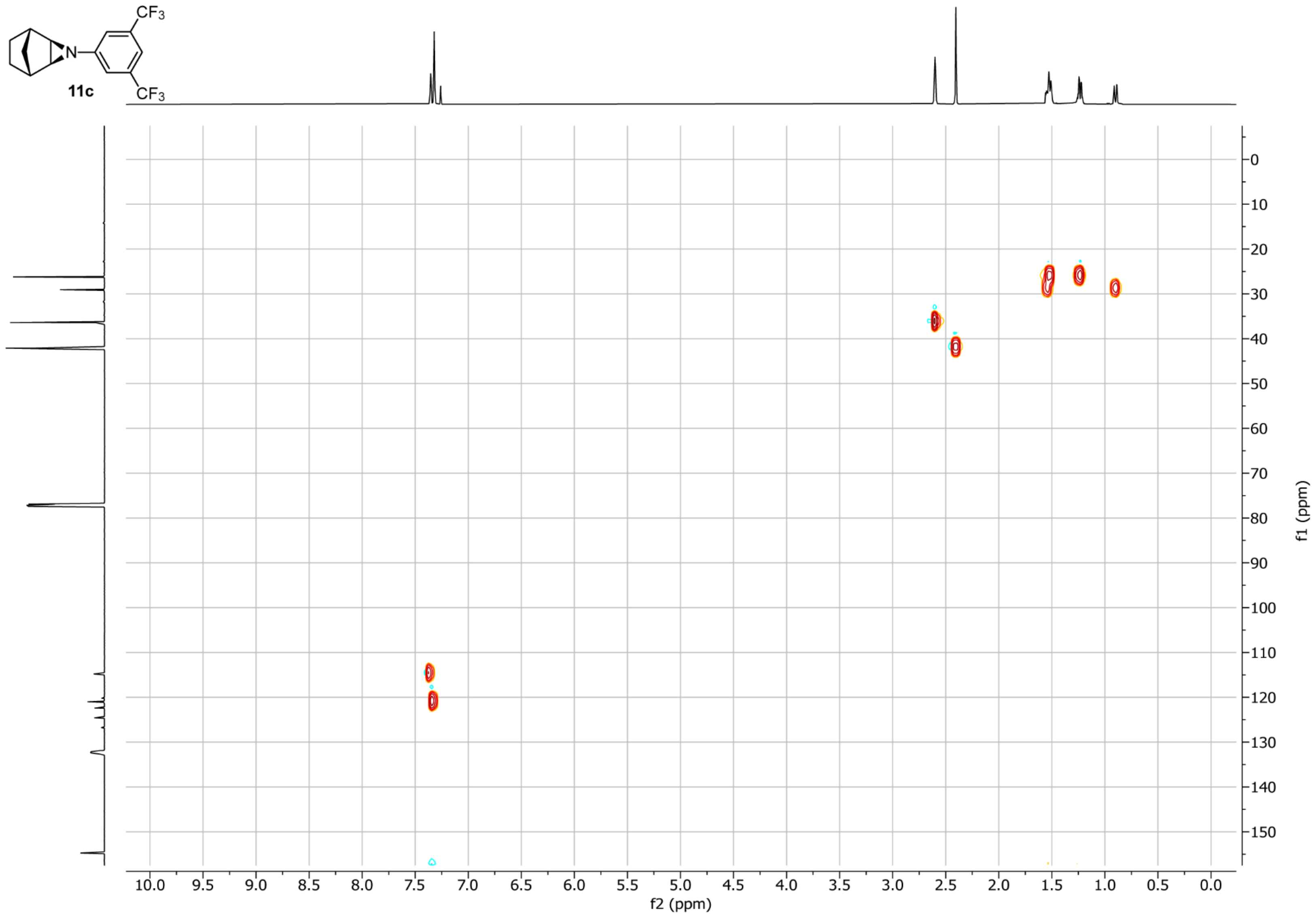

Figure S102. HSQC NMR spectrum of *exo*-3-(3,5-bis(trifluoromethyl)phenyl)-3-azatricyclo[3.2.1.0^{2,4}]octane (**11c**) in $CDCl_3$ at 23 °C.

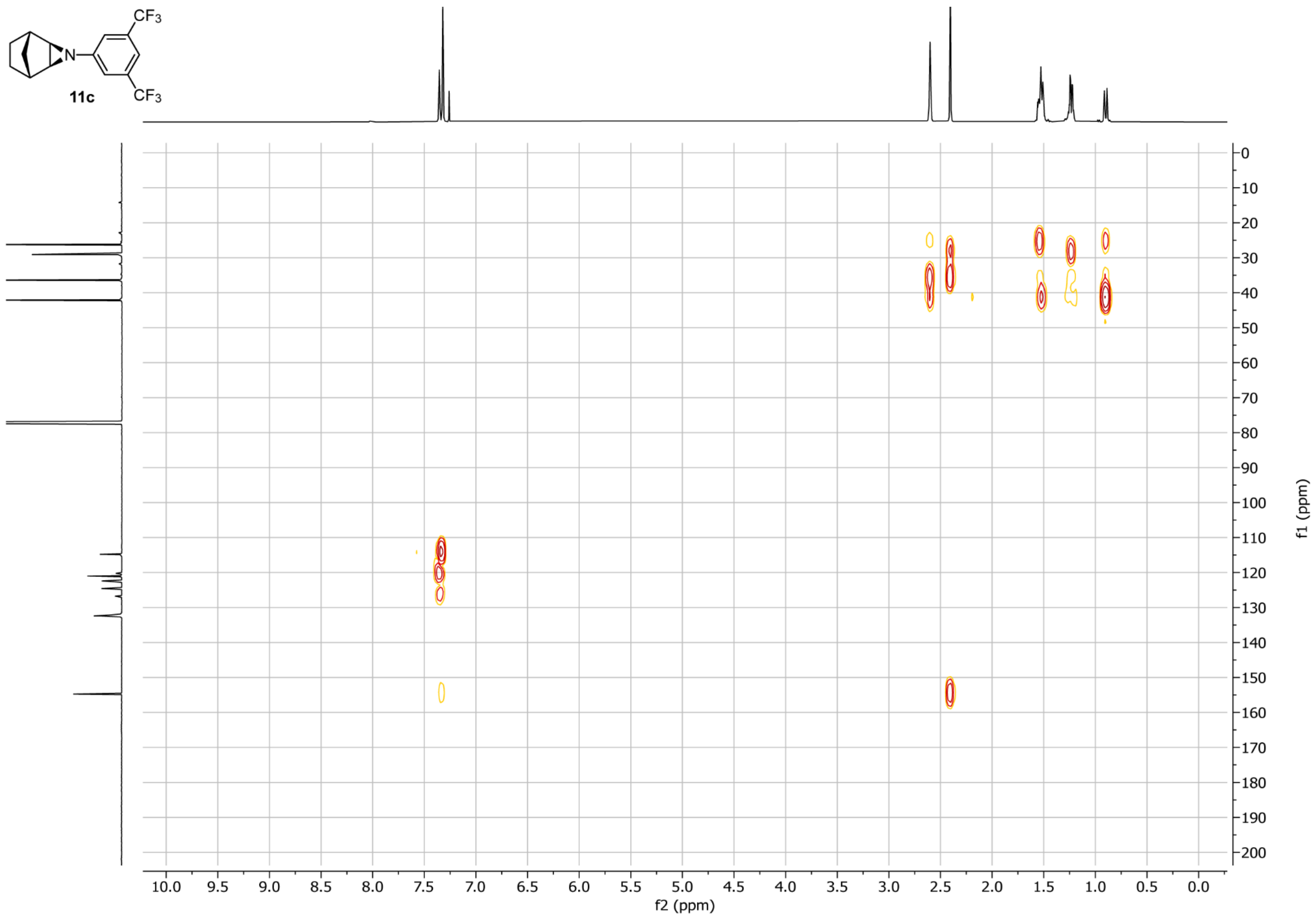

Figure S103. HMBC NMR spectrum of *exo*-3-(3,5-bis(trifluoromethyl)phenyl)-3-azatricyclo[3.2.1.0^{2,4}]octane (**11c**) in $CDCl_3$ at 23 °C.

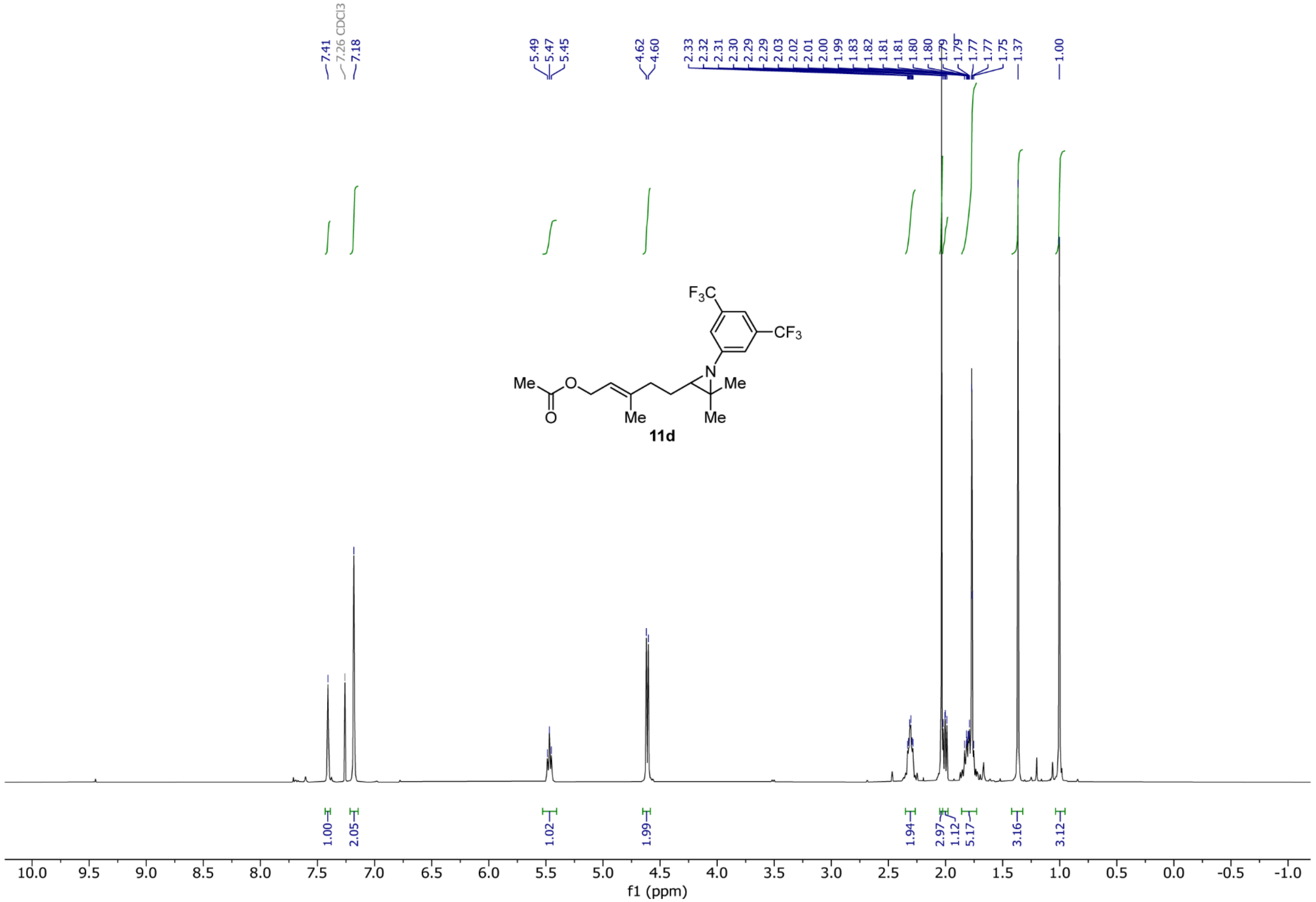

Figure S104. ^{1}H NMR spectrum of (*E*)-5-(1-(3,5-bis(trifluoromethyl)phenyl)-3,3-dimethylaziridin-2-yl)-3-methylpent-2-en-1-yl acetate (**11d**) in $CDCl_3$ (400 MHz) at 23 °C.

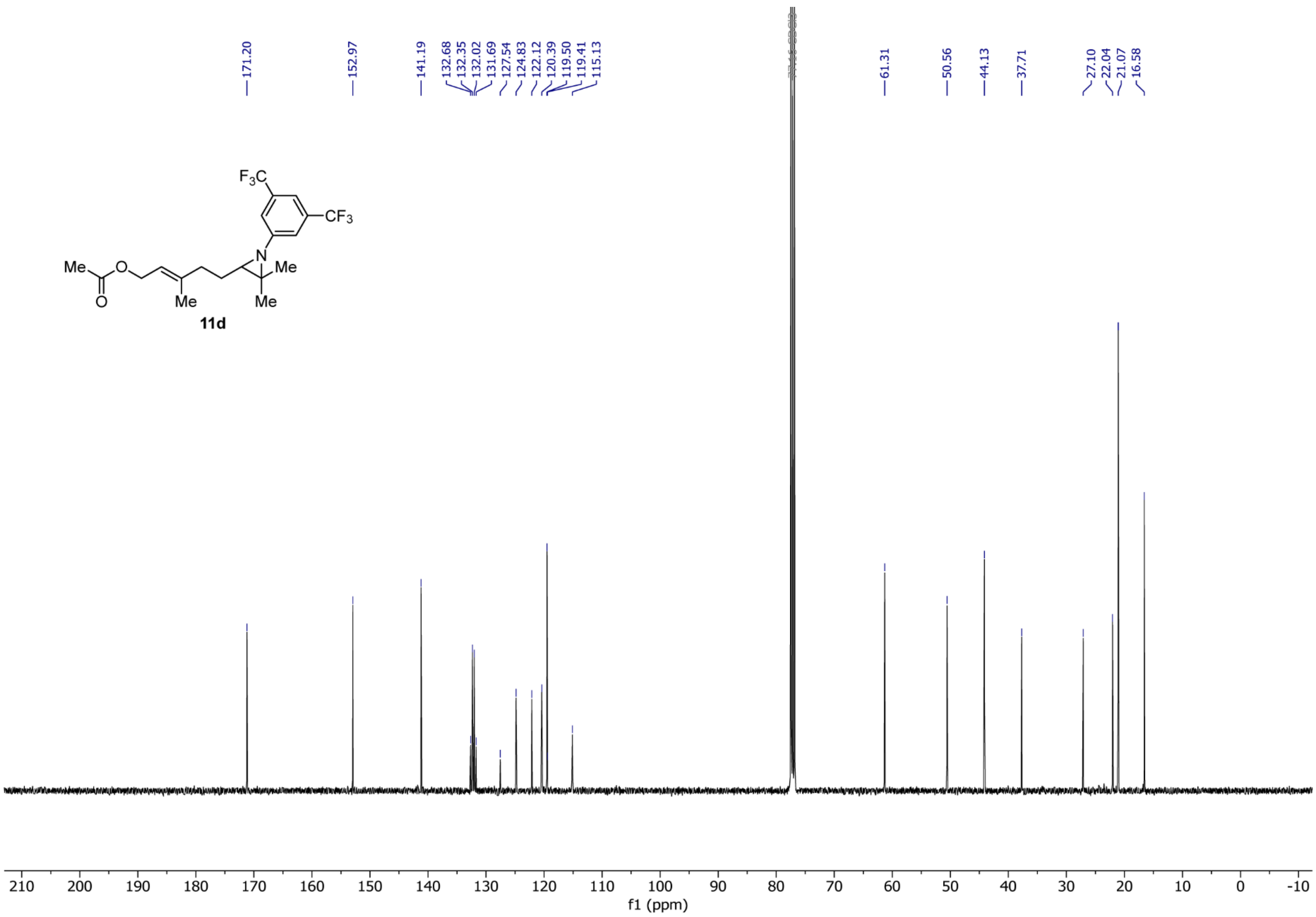

Figure S105. ^{13}C NMR spectrum of (*E*)-5-(1-(3,5-bis(trifluoromethyl)phenyl)-3,3-dimethylaziridin-2-yl)-3-methylpent-2-en-1-yl acetate (**11d**) in $CDCl_3$ (101 MHz) at 23 °C.

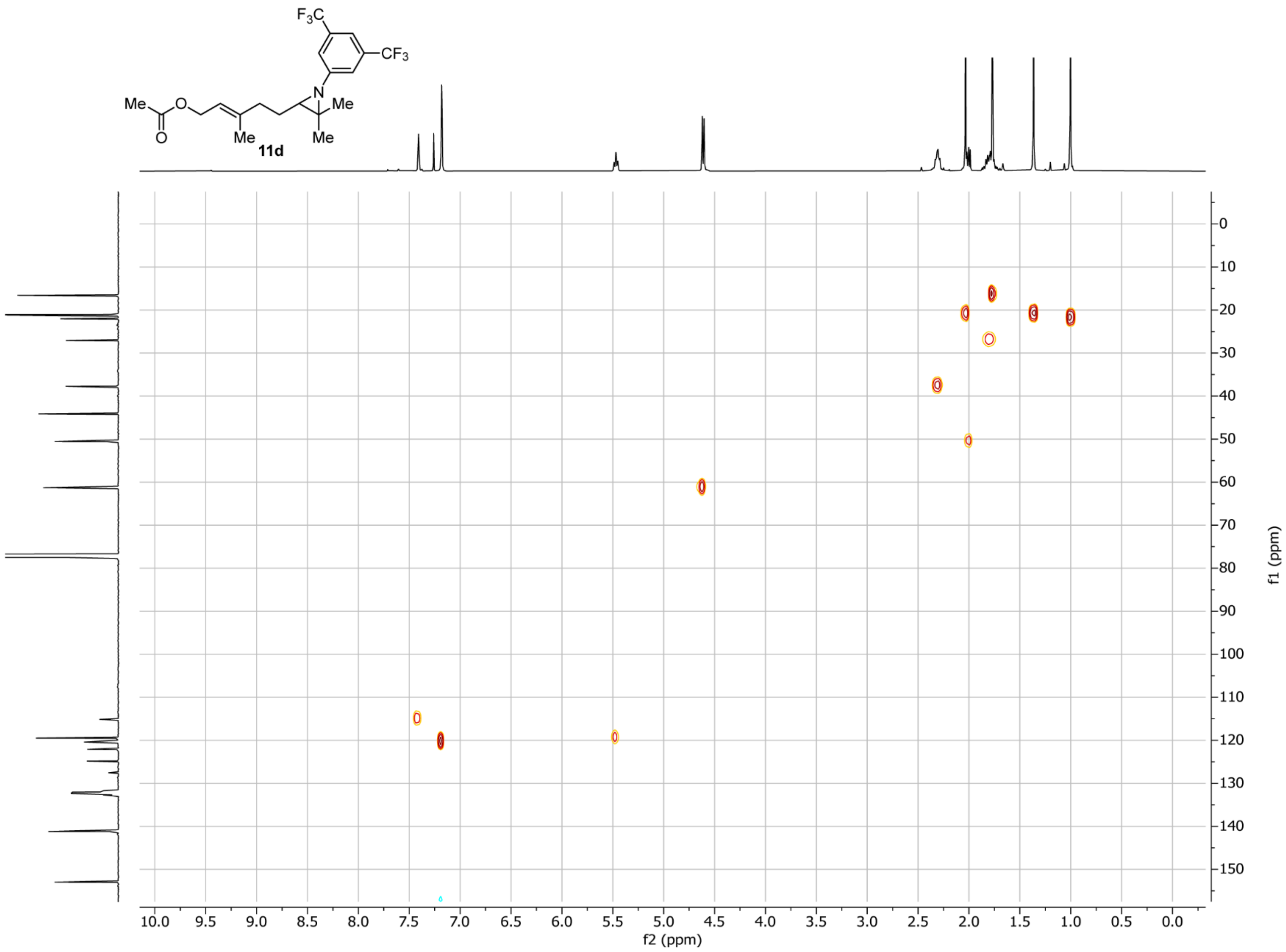

Figure S106. HSQC NMR spectrum of (*E*)-5-(1-(3,5-bis(trifluoromethyl)phenyl)-3,3-dimethylaziridin-2-yl)-3-methylpent-2-en-1-yl acetate (**11d**) in $CDCl_3$ at 23 °C.

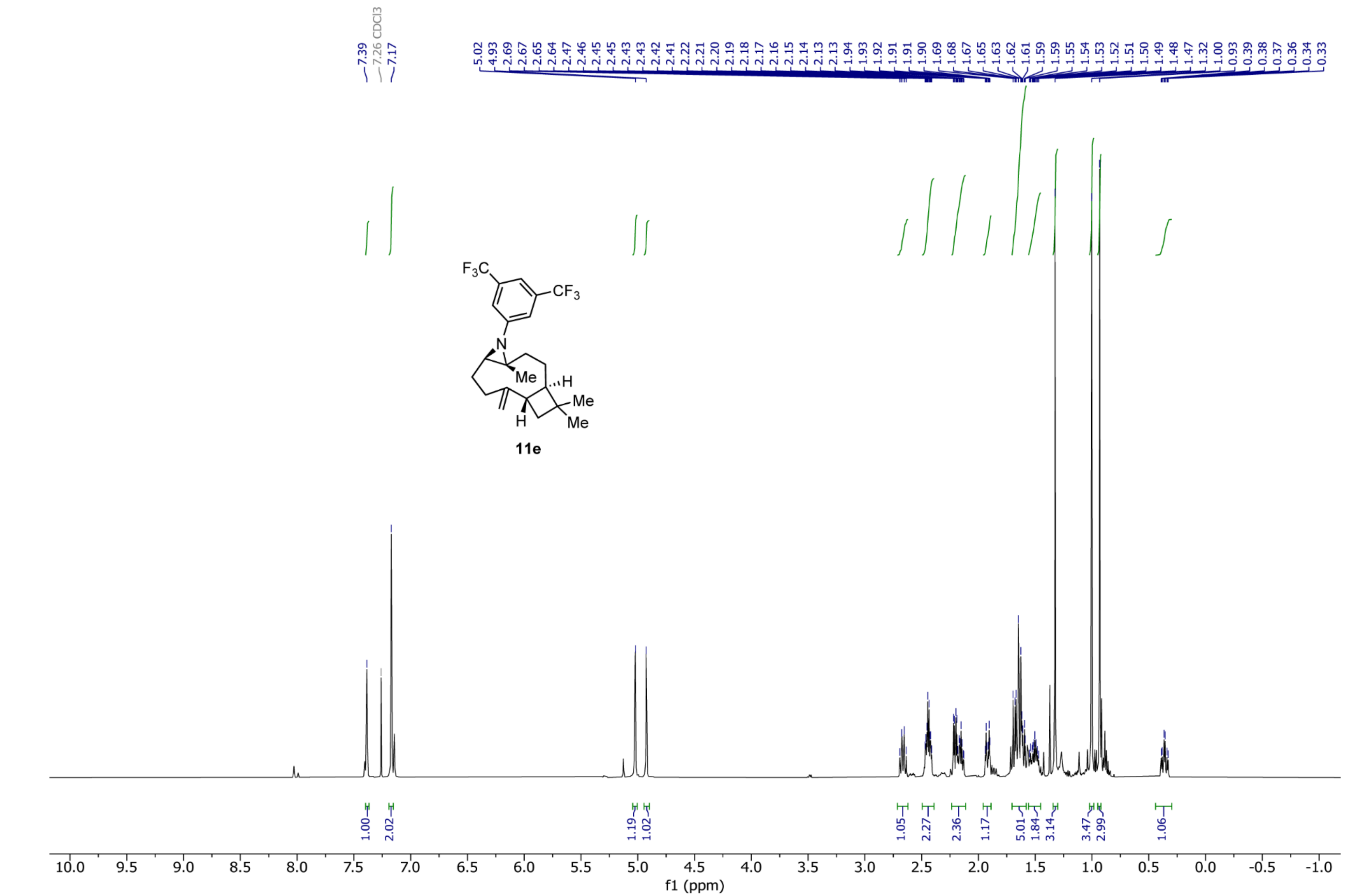

Figure S107. ^{1}H NMR spectrum of (1*R*,4*R*,6*R*,10*S*)-5-(3,5-bis(trifluoromethyl)phenyl)-4,12,12-trimethyl-9-methylene-5-azatricyclo[8.2.0.0^{4,6}]dodecane (**11e**) in $CDCl_3$ (500 MHz) at 23 °C.

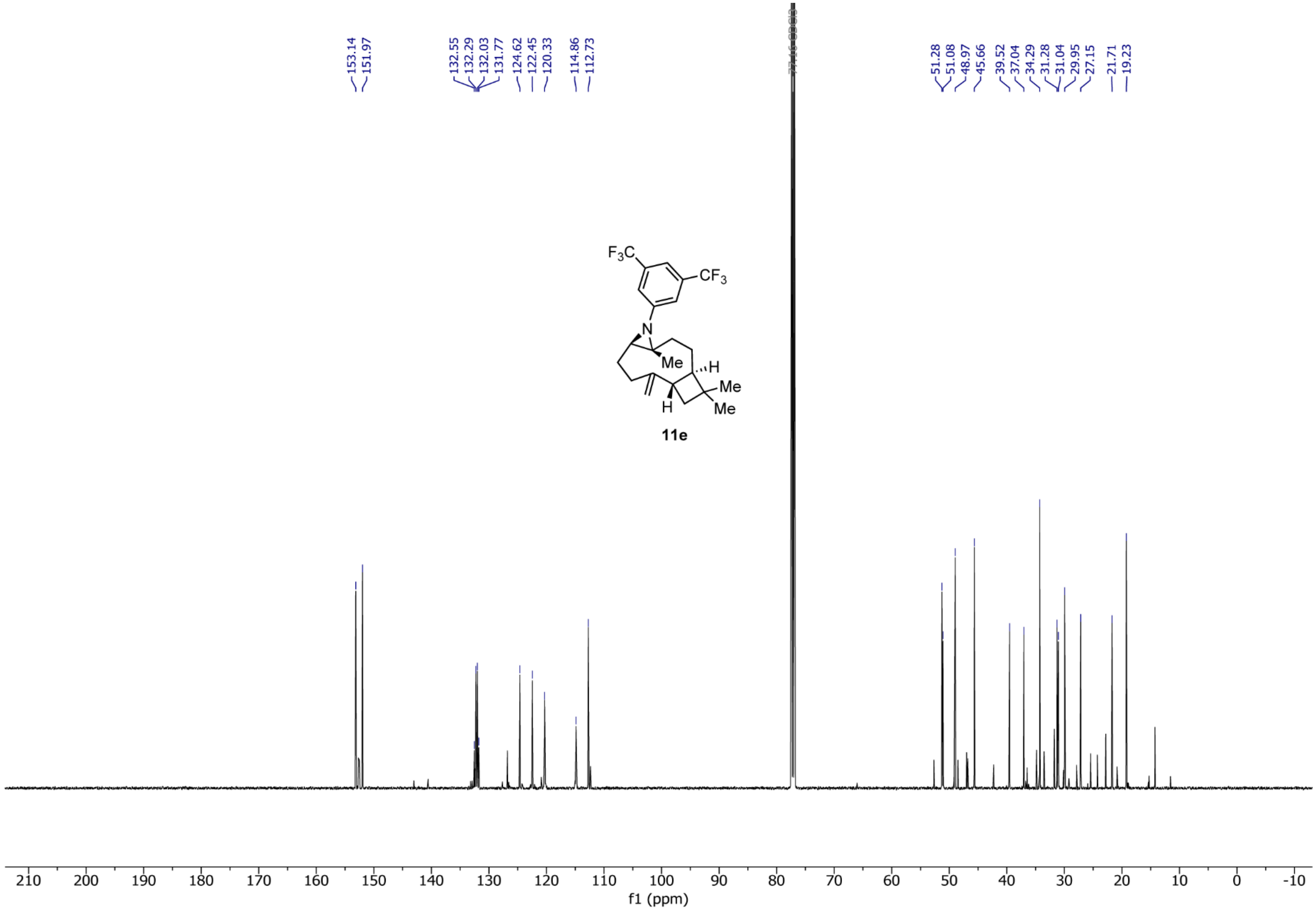

Figure S108. ^{13}C NMR spectrum of (1*R*,4*R*,6*R*,10*S*)-5-(3,5-bis(trifluoromethyl)phenyl)-4,12,12-trimethyl-9-methylene-5-azatricyclo[8.2.0.0^{4,6}]dodecane (**11e**) in $CDCl_3$ (126 MHz) at 23 °C. Extra peaks are attributed to a co-eluting diastereomer.

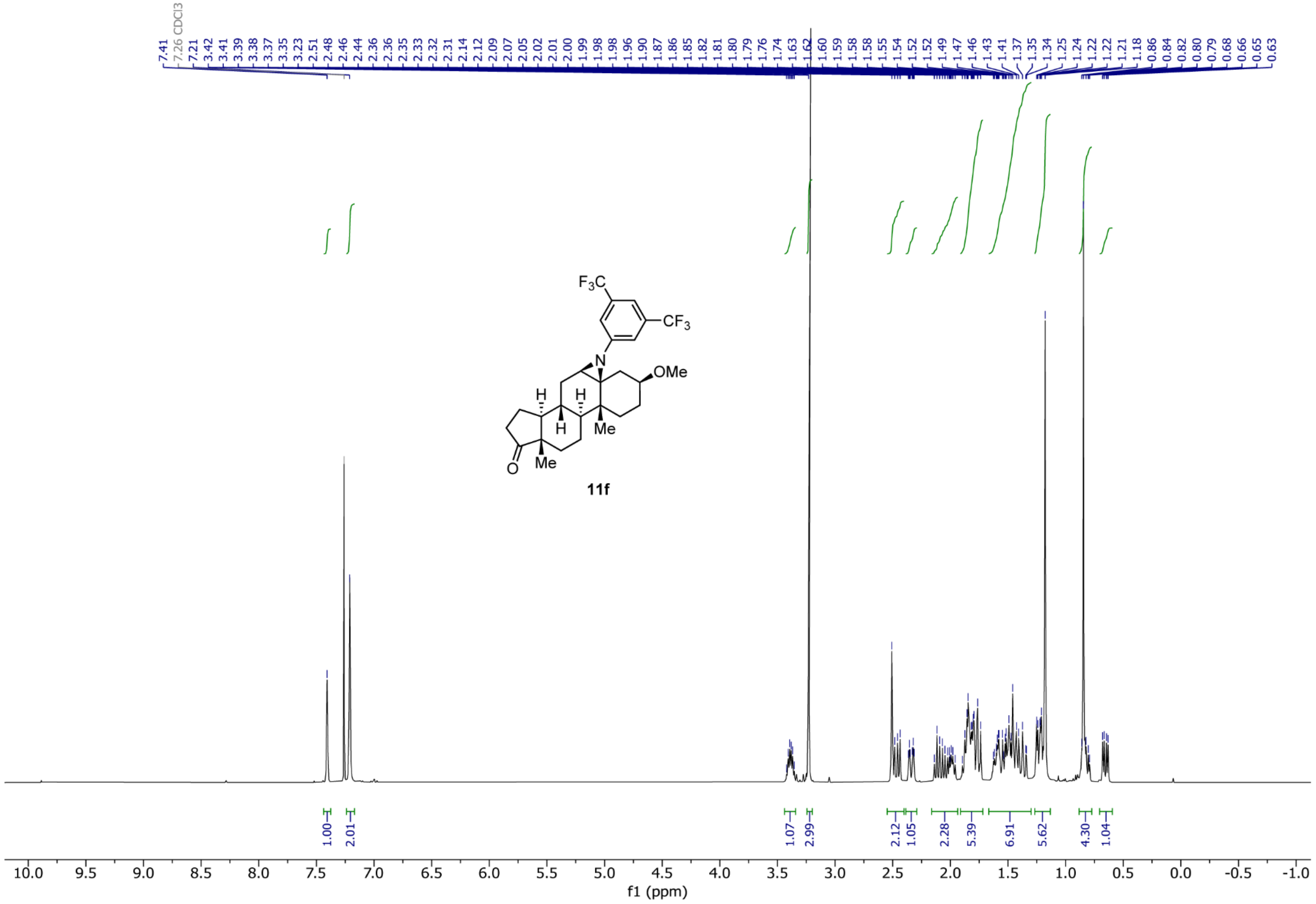

Figure S109. ^{1}H NMR spectrum of (3*S*,4a*S*,5a*R*,6a*R*,6b*S*,9a*S*,11a*S*,11b*R*)-5-(3,5-bis(trifluoromethyl)phenyl)-3-methoxy-9a,11b-dimethylhexadecahydro-9*H*-cyclopenta[1,2]phenanthro[8a,9-*b*]azirin-9-one (**11f**) in $CDCl_3$ (400 MHz) at 23 °C.

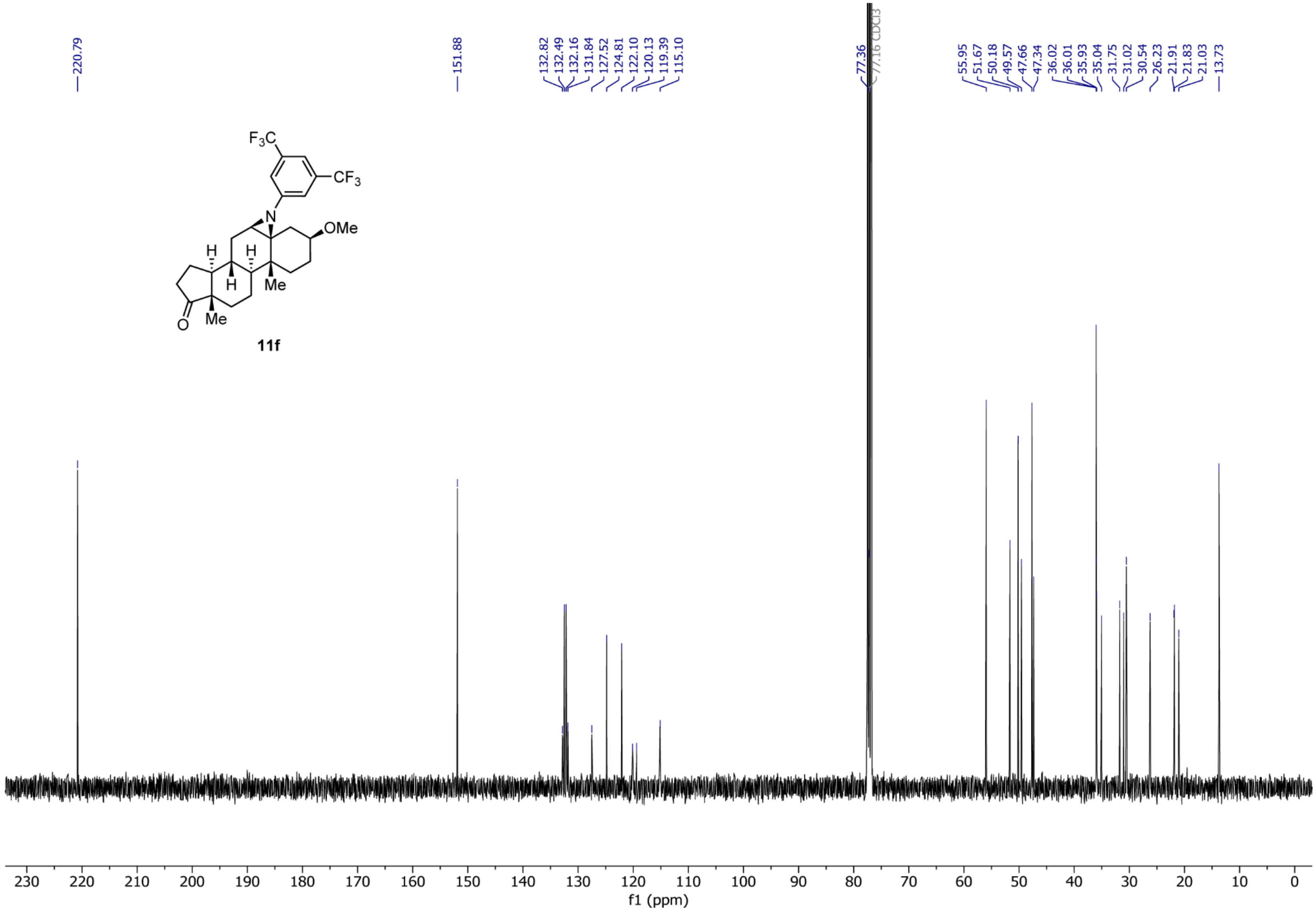

Figure S110. ^{13}C NMR spectrum of (3*S*,4a*S*,5a*R*,6a*R*,6b*S*,9a*S*,11a*S*,11b*R*)-5-(3,5-bis(trifluoromethyl)phenyl)-3-methoxy-9a,11b-dimethylhexadecahydro-9*H*-cyclopenta[1,2]phenanthro[8a,9-*b*]azirin-9-one (**11f**) in $CDCl_3$ (101 MHz) at 23 °C.

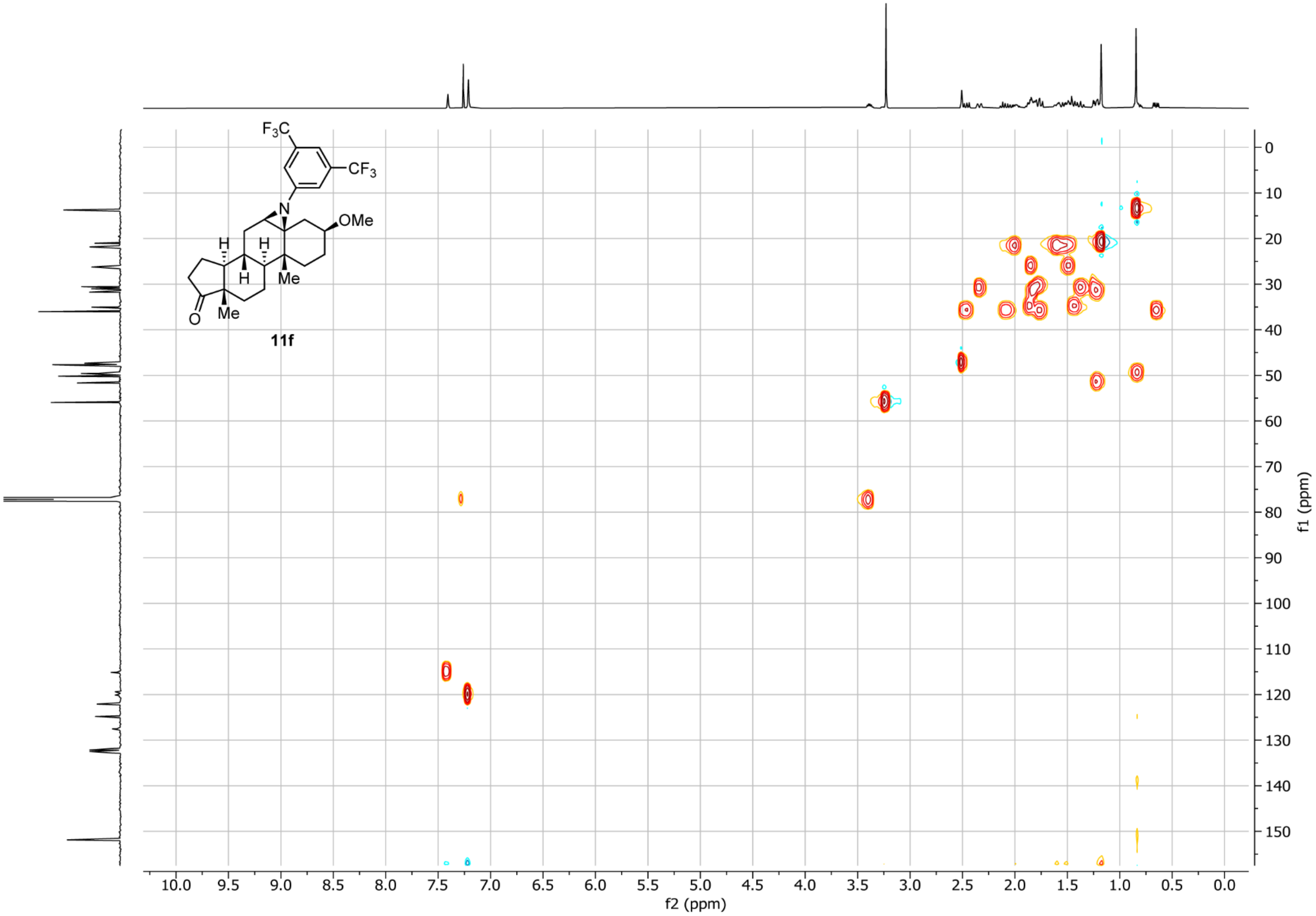

Figure S111. HSQC NMR spectrum of (3*S*,4a*S*,5a*R*,6a*R*,6b*S*,9a*S*,11a*S*,11b*R*)-5-(3,5-bis(trifluoromethyl)phenyl)-3-methoxy-9a,11b-dimethylhexadecahydro-9*H*-cyclopenta[1,2]phenanthro[8a,9-*b*]azirin-9-one (**11f**) in $CDCl_3$ at 23 °C.

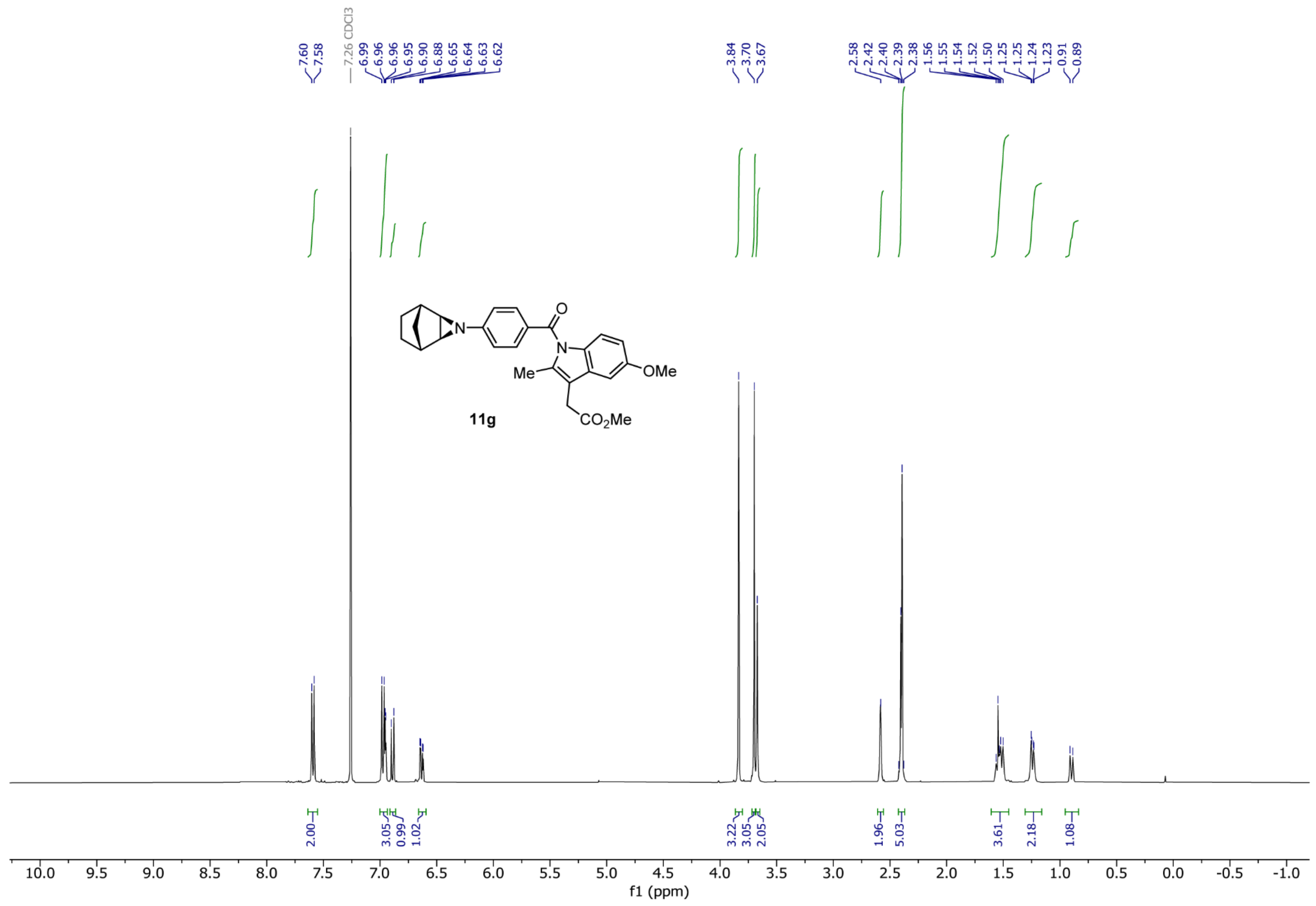

Figure S112. ^{1}H NMR spectrum of methyl *exo*-2-(1-(4-(3-azatricyclo[3.2.1.0^{2,4}]octan-3-yl)benzoyl)-5-methoxy-2-methyl-1*H*-indol-3-yl)acetate (**11g**) in $CDCl_3$ (400 MHz) at 23 °C.

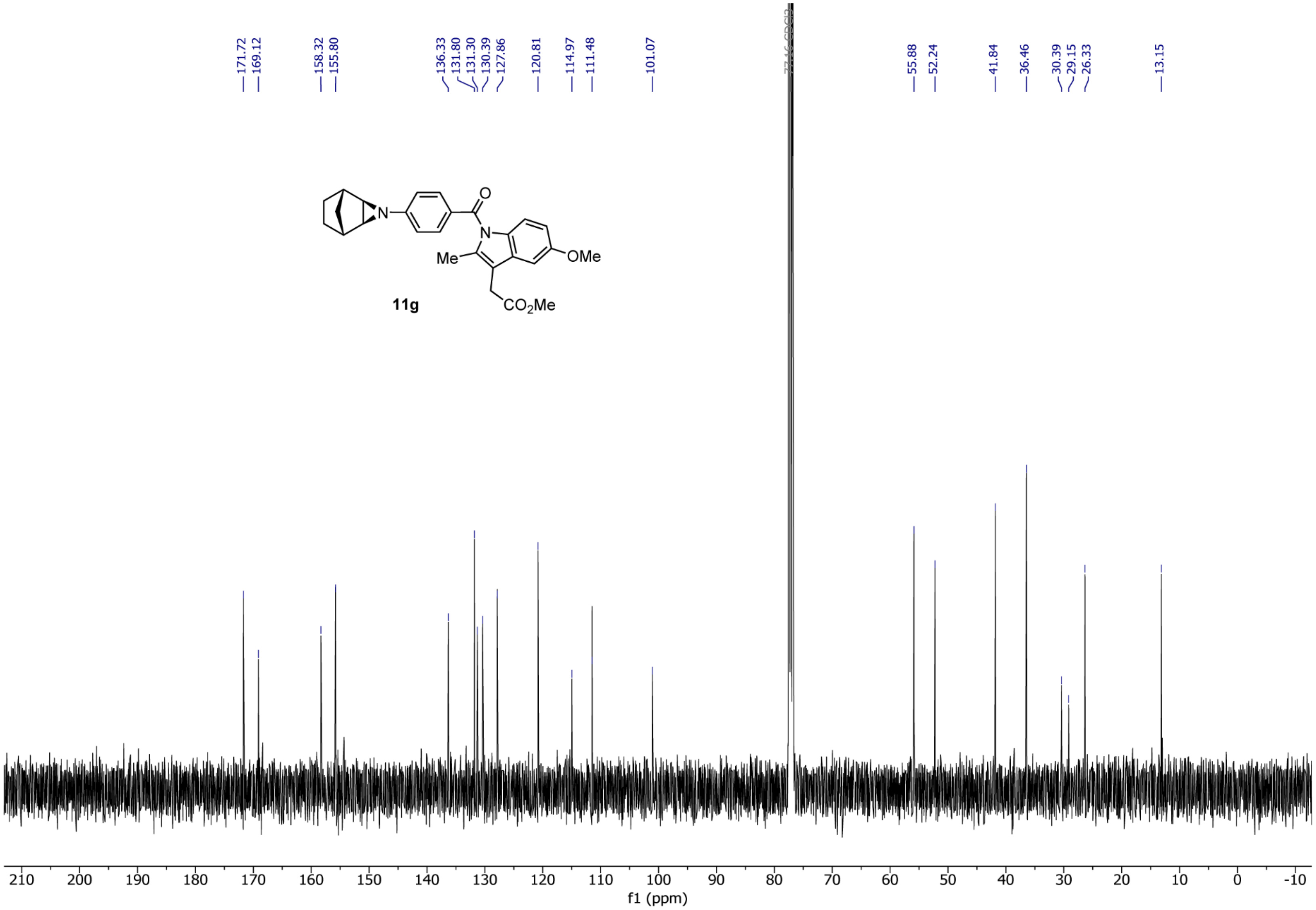

Figure S113. ^{13}C NMR spectrum of methyl *exo*-2-(1-(4-(3-azatricyclo[3.2.1.0^{2,4}]octan-3-yl)benzoyl)-5-methoxy-2-methyl-1*H*-indol-3-yl)acetate (**11g**) in $CDCl_3$ (101 MHz) at 23 °C.

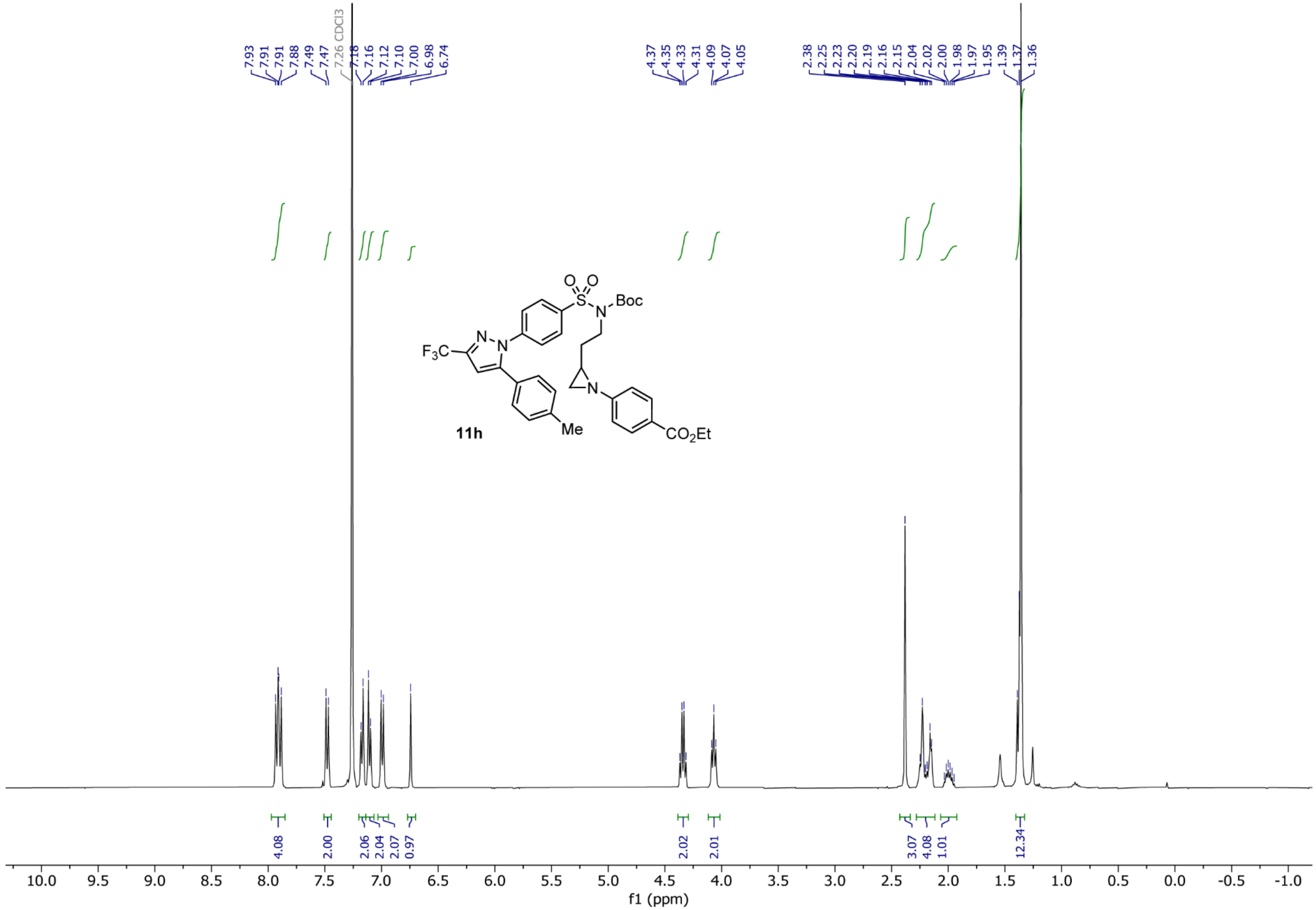

Figure S114. ^{1}H NMR spectrum of ethyl 4-(2-(2-((*N*-(*tert*-butoxycarbonyl)-4-(5-(*p*-tolyl)-3-(trifluoromethyl)-1*H*-pyrazol-1-yl)phenyl)sulfonamido)ethyl)aziridin-1-yl)benzoate (**11h**) in $CDCl_3$ (400 MHz) at 23 °C.

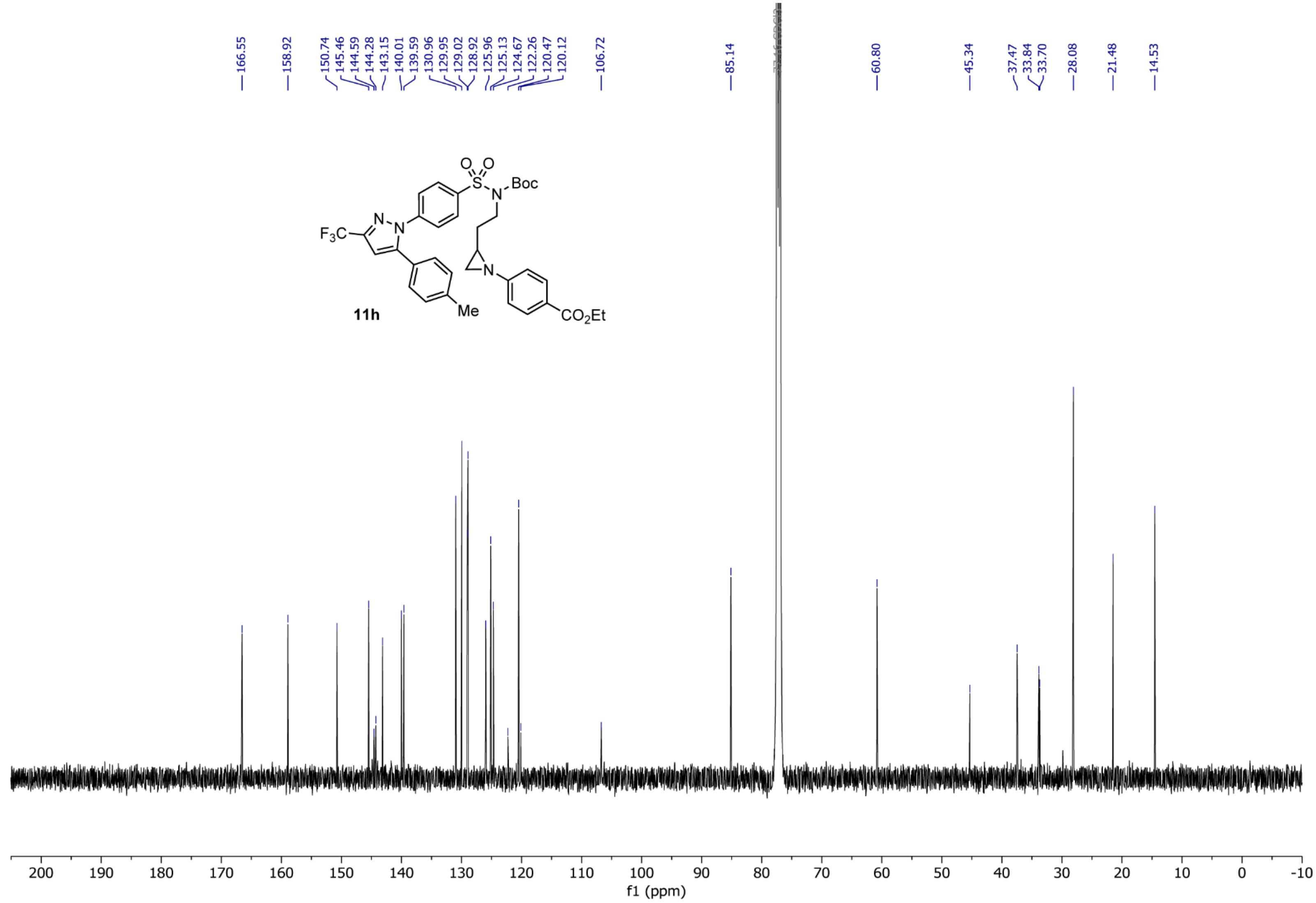

Figure S115. ^{13}C NMR spectrum of ethyl 4-(2-(2-((*N*-(*tert*-butoxycarbonyl)-4-(5-(*p*-tolyl)-3-(trifluoromethyl)-1*H*-pyrazol-1-yl)phenyl)sulfonamido)ethyl)aziridin-1-yl)benzoate (**11h**) in $CDCl_3$ (126 MHz) at 23 °C.

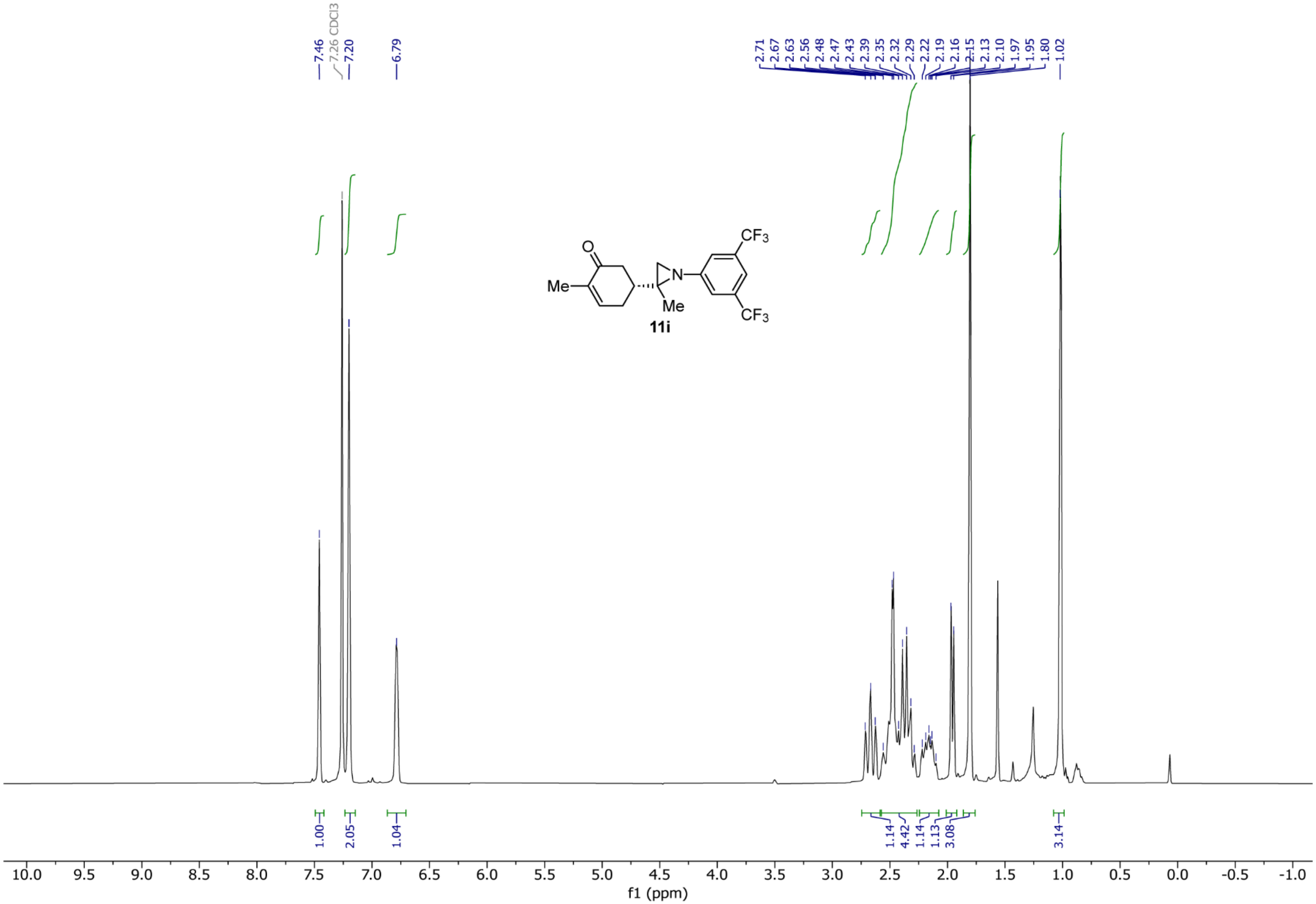

Figure S116. ^{1}H NMR spectrum of (*R*)-5-(1-(3,5-bis(trifluoromethyl)phenyl)-2-methylaziridin-2-yl)-2-methylcyclohex-2-en-1-one (**11i**) in $CDCl_3$ (400 MHz) at 23 °C.

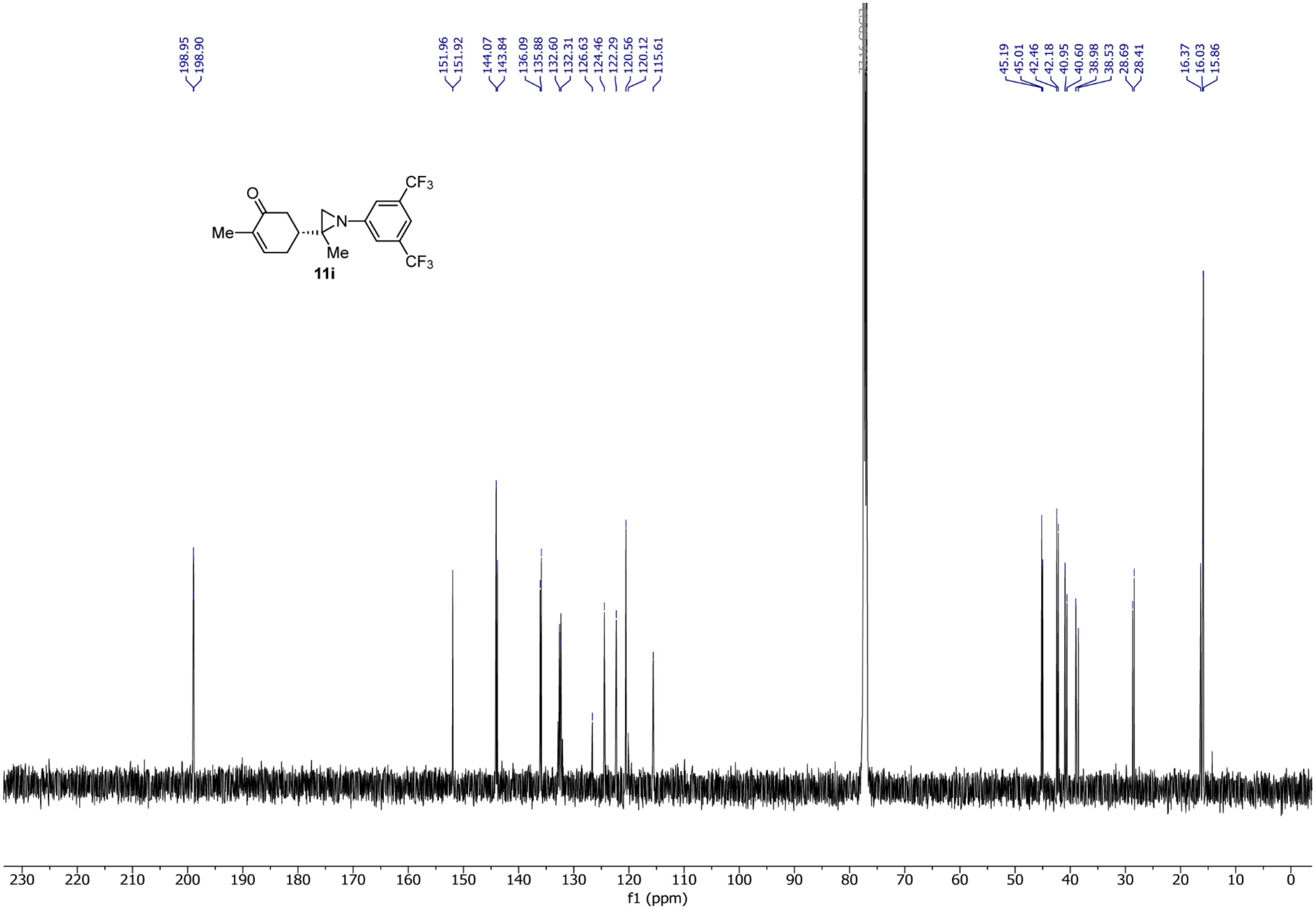

Figure S117. ^{13}C NMR spectrum of (*R*)-5-(1-(3,5-bis(trifluoromethyl)phenyl)-2-methylaziridin-2-yl)-2-methylcyclohex-2-en-1-one (**11i**) in $CDCl_3$ (101 MHz) at 23 °C.

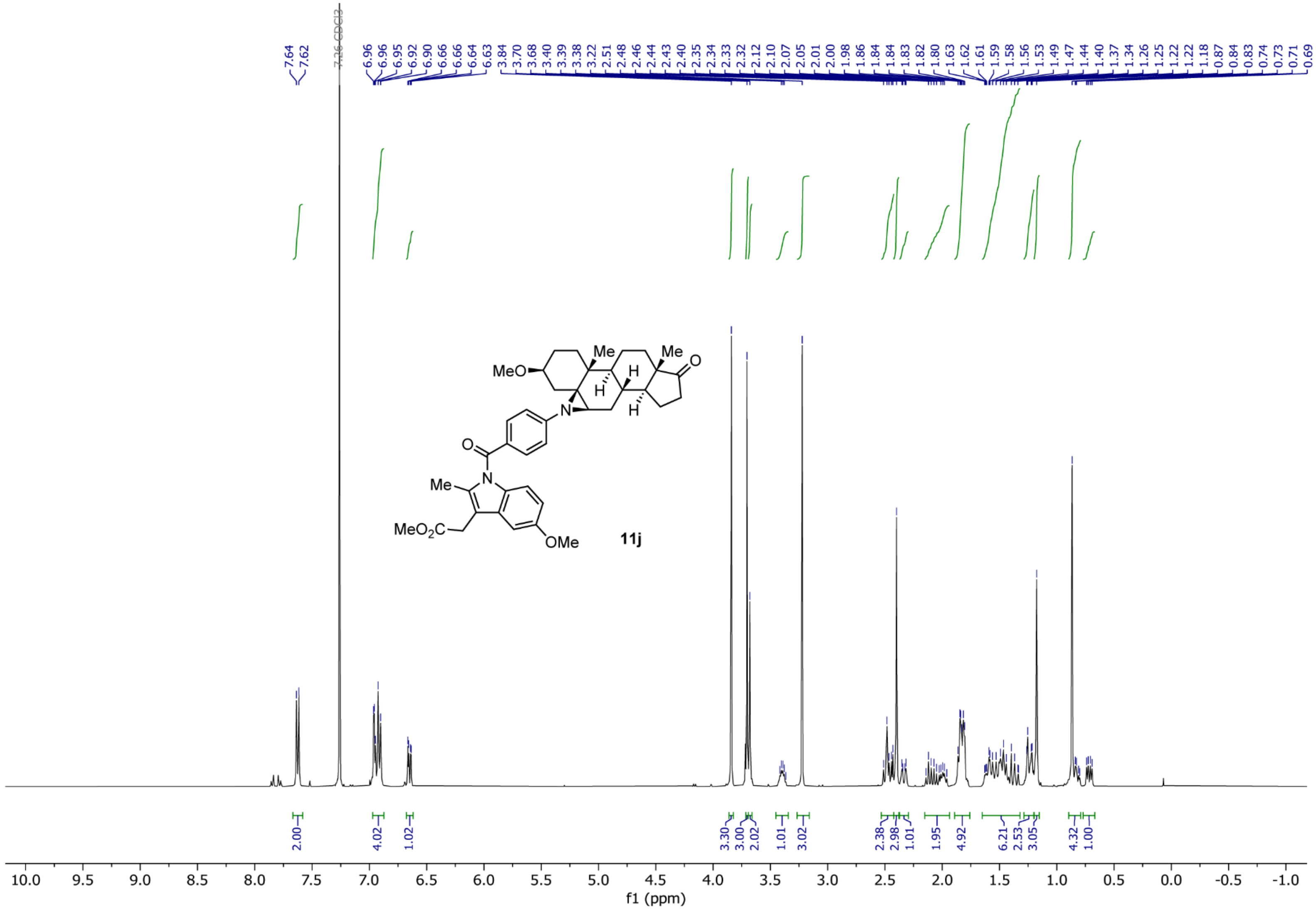

Figure S118. ^{1}H NMR spectrum of methyl 2-(5-methoxy-1-(4-((3*S*,4a*S*,5a*R*,6a*R*,6b*S*,9a*S*,11a*S*,11b*R*)-3-methoxy-9a,11b-dimethyl-9-oxohexadecahydro-5*H*-cyclopenta[1,2]phenanthro[8a,9-*b*]azirin-5-yl)benzoyl)-2-methyl-1*H*-indol-3-yl)acetate (**11j**) in $CDCl_3$ (400 MHz) at 23 °C.

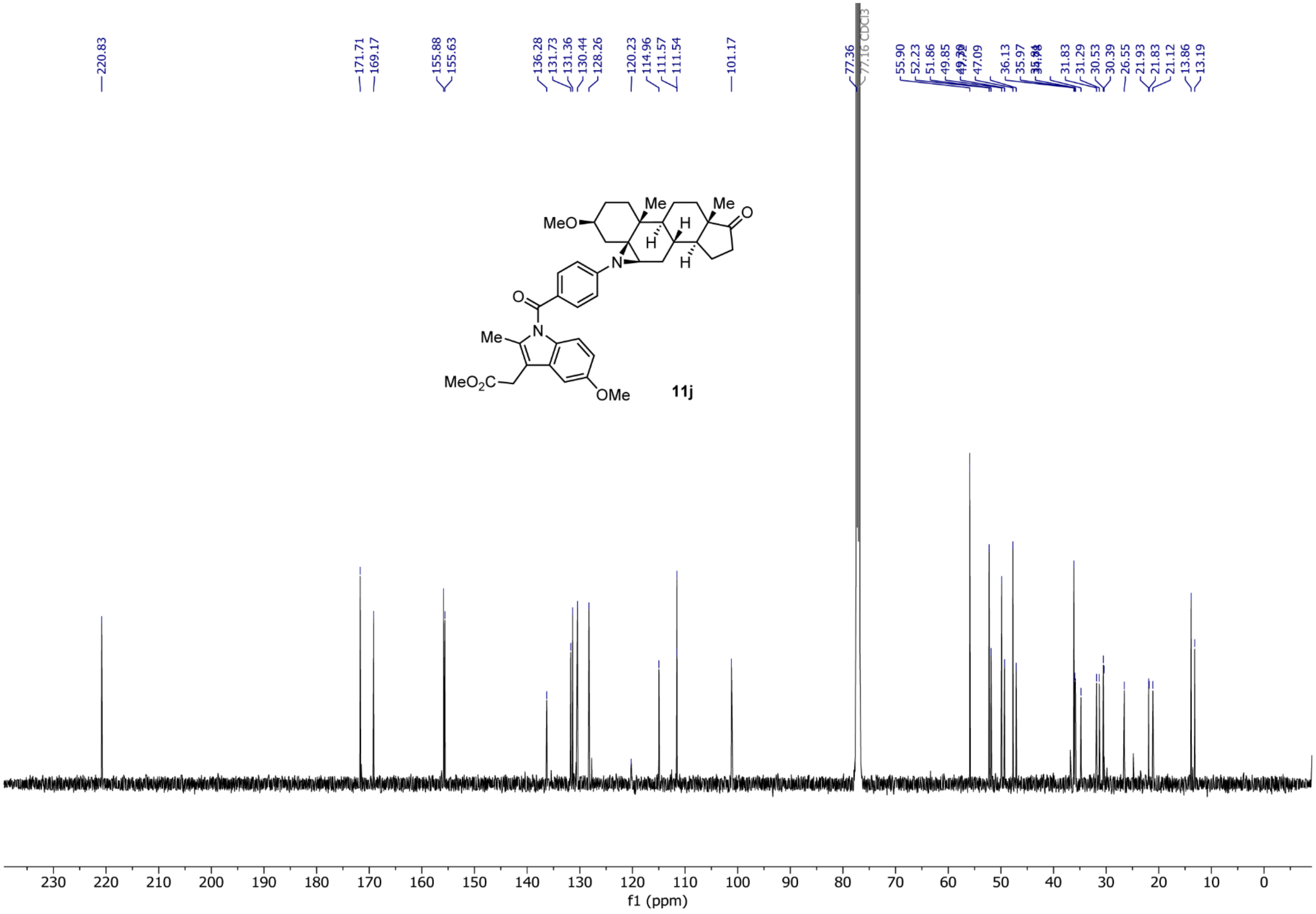

Figure S119. ^{13}C NMR spectrum of methyl 2-(5-methoxy-1-(4-((3*S*,4a*S*,5a*R*,6a*R*,6b*S*,9a*S*,11a*S*,11b*R*)-3-methoxy-9a,11b-dimethyl-9-oxohexadecahydro-5*H*-cyclopenta[1,2]phenanthro[8a,9-*b*]azirin-5-yl)benzoyl)-2-methyl-1*H*-indol-3-yl)acetate (**11j**) in $CDCl_3$ (126 MHz) at 23 °C.

I. Supplementary References

1. Armarego, W. L. F.; Chai, C. L. L. Chapter 4 - Purification of Organic Chemicals. In *Purification of Laboratory Chemicals (Sixth Edition)*, Armarego, W. L. F.; Chai, C. L. L., Eds. Butterworth-Heinemann: Oxford, 2009; pp 88–444.
2. Pangborn, A. B.; Giardello, M. A.; Grubbs, R. H.; Rosen, R. K.; Timmers, F. J. Safe and Convenient Precedure for Solvent Purification. *Organometallics* **1996**, *15*, 1518–1520.
3. Li, X.; He, S.; Song, Q. Diethylzinc-Mediated Radical 1,2-Addition of Alkenes and Alkynes *Org. Lett.* **2021**, *23*, 2994–2999.
4. Guérin, C.; Jean-Gérard, L.; Octobre, G.; Pascal, S.; Maury, O.; Pilet, G.; Ledoux, A.; Andrioletti, B. Bis-triazolyl BODIPYs: a simple dye with strong red-light emission *RSC Adv.* **2015**, *5*, 76342–76345.
5. Youn, S. W.; Pastine, S. J.; Sames, D. Ru(III)-Catalyzed Cyclization of Arene-Alkene Substrates via Intramolecular Electrophilic Hydroarylation. *Org. Lett.* **2004**, *6*, 581–584.
6. Dong, J. Y.; Manias, E.; Chung, T. C. Functionalized Syndiotactic Polystyrene Polymers Prepared by the Combination of Metallocene Catalyst and Borane Comonomer. *Macromolecules* **2002**, *35*, 3439–3447.
7. Oberhauser, C.; Harms, V.; Seidel, K.; Schröder, B.; Ekramzadeh, K.; Beutel, S.; Winkler, S.; Lauterbach, L.; Dickschat, J. S.; Kirschning, A. Exploiting the Synthetic Potential of Sesquiterpene Cyclases for Generating Unnatural Terpenoids. *Angew. Chem. Int. Ed.* **2018**, *57*, 11802–11806.
8. Imanishi, Y.; Awai, N.; Hirai, M.; Hosaka, T.; Kono, R. Large Conductance Calcium-Activated K Channel Opener. WO2005037271, 2004.
9. Iinuma, M.; Moriyama, K.; Togo, H. Simple and Practical Method for Preparation of [(Diacetoxy)iodo]arenes with Iodoarenes and *m*-Chloroperoxybenzoic Acid. *Synlett* **2012**, *23*, 2663–2666.
10. Maity, A.; Hyun, S.-M.; Powers, D. C. Oxidase catalysis via aerobically generated hypervalent iodine intermediates. *Nat. Chem.* **2018**, *10*, 200–204.
11. Kazmierczak, P.; Skulski, L.; Kraszkiewicz, L. Syntheses of (Diacetoxyiodo)arenes or Iodylarenes from Iodoarenes, with Sodium Periodate as the Oxidant. *Molecules* **2001**, *6*, 881–891.
12. Fulmer, G. R.; Miller, A. J. M.; Sherden, N. H.; Gottlieb, H. E.; Nudelman, A.; Stoltz, B. M.; Bercaw, J. E.; Goldberg, K. I. NMR Chemical Shifts of Trace Impurities: Common Laboratory Solvents, Organics, and Gases in Deuterated Solvents Relevant to the Organometallic Chemist. *Organometallics* **2010**, *29*, 2176–2179.
13. Sheldrick, G. A short history of SHELX. *Acta Crystallogr. Sect. A* **2008**, *64*, 112–122.
14. Dolomanov, O. V.; Bourhis, L. J.; Gildea, R. J.; Howard, J. A. K.; Puschmann, H. LEX2: a complete structure solution, refinement and analysis program. *J. Appl. Crystallogr.* **2009**, *42*, 339–341.
15. Sheldrick, G. Crystal structure refinement with SHELXL. *Acta Crystallogr. Sect. C* **2015**, *71*, 3–8.
16. Maity, A.; Roychowdhury, P.; Herrera, R. G.; Powers, D. C. Diversification of Amidyl Radical Intermediates Derived from C–H Aminopyridylation. *Org. Lett.* **2022**, *24*, 2762–2766.

17. Mendiola, J.; Rincón, J. A.; Mateos, C.; Soriano, J. F.; de Frutos, Ó.; Niemeier, J. K.; Davis, E. M. Preparation, Use, and Safety of O-Mesitylenesulfonylhydroxylamine. *Org. Process Res. Dev.* **2009**, *13*, 263–267.
18. Tamura, Y.; Minamikawa, J.; Miki, Y.; Matsugashita, S.; Ikeda, M. A novel method for heteroaromatic N-imines. *Tetrahedron Lett.* **1972**, *13*, 4133–4135.
19. Wu, W.-Q.; Qin, H.-L. Synthesis of Pyrazolo[1,5-a]pyridinyl, Pyrazolo[1,5-a]quinolinyl, and Pyrazolo[5,1-a]isoquinolinyl Sulfonyl Fluorides via a [3 + 2] Annulation. *J. Org. Chem.* **2023**, *88*, 3266–3276.
20. Takeuchi, H.; Hayakawa, S.; Tanahashi, T.; Kobayashi, A.; Adachi, T.; Higuchi, D. Novel generation of parent, alkyl, dialkyl and alicyclic nitrenium ions in photolyses of pyridinium, quinolinium, bipyridinium and phenanthrolinium salts and aromatic N-substitution by nitrenium ions. *J. Chem. Soc. Perk. Trans. 2* **1991**, 847–855.
21. Wu, X.; Gannett, C. N.; Liu, J.; Zeng, R.; Novaes, L. F. T.; Wang, H.; Abruña, H. D.; Lin, S. Intercepting Hydrogen Evolution with Hydrogen-Atom Transfer: Electron-Initiated Hydrofunctionalization of Alkenes. *J. Am. Chem. Soc.* **2022**, *144*, 17783–17791.
22. Xu, C.; Huang, W.; Zhang, R.; Gao, C.; Li, Y.; Wang, M. Trifluoromethylations of Alkenes Using $PhICF_3Cl$ as Bifunctional Reagent. *J. Org. Chem.* **2019**, *84*, 14209–14216.
23. Qian, X.; Auffrant, A.; Felouat, A.; Gosmini, C. Cobalt-Catalyzed Reductive Allylation of Alkyl Halides with Allylic Acetates or Carbonates. *Angew. Chem. Int. Ed.* **2011**, *50*, 10402–10405.
24. White, S. K.; Ge, Y.; Huang, Y. Methods for Preparing 17-Alkynyl-7-Hydroxy Steroids and Related Compounds. US9163059B2, 2009.
25. Kiran, I.; Hanson, J.; Hunter, A. The microbiological hydroxylation of some methoxysteroids by Cephalosporium aphidicola. *J. Chem. Res.* **2004**, 362–363.
26. Tan, H.; Samanta, S.; Maity, A.; Roychowdhury, P.; Powers, D. C. *N*-Aminopyridinium reagents as traceless activating groups in the synthesis of *N*-Aryl aziridines. *Nat. Commun.* **2022**, *13*, 3341.
27. Cheng, Q.-Q.; Zhou, Z.; Jiang, H.; Siitonen, J. H.; Ess, D. H.; Zhang, X.; Kürti, L. Organocatalytic nitrogen transfer to unactivated olefins via transient oxaziridines. *Nat. Catal.* **2020**, *3*, 386–392.
28. Fleming, I.; Frackenpohl, J.; Ila, H. Cleavage of sulfonamides with phenyldimethylsilyllithium. *J. Chem. Soc., Perkin Trans. 1* **1998**, 1229–1236.
29. Jat, J. L.; Chandra, D.; Kumar, P.; Singh, V.; Tiwari, B. Metal- and Additive-Free Intermolecular Aziridination of Olefins Using *N*-Boc-*O*-tosylhydroxylamine. *Synthesis* **2022**, *54*, 4513–4520.
30. Zu, B.; Ke, J.; Guo, Y.; He, C. Synthesis of Diverse Aryliodine(III) Reagents by Anodic Oxidation *Chin. J. Chem .* **2021**, *39*, 627–632.
31. Hiller, A.; Patt, J. T.; Steinbach, J. NMR study on the structure and stability of 4-substituted aromatic iodosyl compounds. *Magn. Reson. Chem.* **2006**, *44*, 955–958.
32. Burés, J. Variable Time Normalization Analysis: General Graphical Elucidation of Reaction Orders from Concentration Profiles. *Angew. Chem. Int. Ed.* **2016**, *55*, 16084–16087.
33. C. Hansch; A. Leo; R. W. Taft. A survey of Hammett substituent constants and resonance and field parameters *Chem. Rev.* **1991**, *91*, 165–195.
34. Bagchi, V.; Kalra, A.; Das, P.; Paraskevopoulou, P.; Gorla, S.; Ai, L.; Wang, Q.; Mohapatra, S.; Choudhury, A.; Sun, Z.; Cundari, T. R.; Stavropoulos, P. Comparative Nitrene-Transfer

Chemistry to Olefinic Substrates Mediated by a Library of Anionic Mn(II) Triphenylamido-Amine Reagents and M(II) Congeners (M = Fe, Co, Ni) Favoring Aromatic over Aliphatic Alkenes. *ACS Catal.* **2018**, *8*, 9183–9206.
